# High glutamine suppresses osteogenesis through mTORC1-mediated inhibition of the mTORC2/AKT-473/RUNX2 axis

**DOI:** 10.1038/s41420-022-01077-3

**Published:** 2022-06-07

**Authors:** Meher Bolisetti Gayatri, Navya Naidu Gajula, Suresh Chava, Aramati B. M. Reddy

**Affiliations:** 1 Department of Animal Biology, School of Life Sciences, University of Hyderabad, Hyderabad, India; 2 Present Address: Department of Biochemistry and Molecular Genetics, University of Alabama at Birmingham, Birmingham, Alabama USA

**Keywords:** TOR signalling, Diabetes complications

## Abstract

Activation of the key nutrient cellular sensors mTORC1 and mTORC2 directs the fate of mesenchymal stromal cells (MSCs). Here, we report that glutamine regulates crosstalk between mTOR complexes and lineage commitment of MSCs independent of glucose concentration. High glutamine-induced mTORC1 hyperactivation resulted in the suppression of mTORC2, which otherwise stabilizes RUNX2 via GSK3β inhibition through pAKT-473. Activation of GSK3β resulted in the ubiquitination of RUNX2, a key transcription factor for the osteogenic commitment of MSCs. However, low glutamine conditions inhibit mTORC1 hyperactivation followed by increased mTORC2 activation and RUNX2 stabilization. Under diabetic/high-glucose conditions, glutamine-triggered hyperactivation of mTORC1 resulted in mTORC2 suppression, and active GSK3β led to suppression of RUNX2. Activation of p-AMPK by metformin inhibits high glutamine-induced mTORC1 hyperactivation and rescues RUNX2 through the mTORC2/AKT-473 axis. Collectively, our study indicates the role of glutamine in modulating MSC fate through cross-talk between mTOR complexes by identifying a critical switch in signaling. It also shows the importance of glutamine in modulating molecular cues (mTORC1/p-70S6K/mTORC2/RUNX2) that are involved in driving diabetes-induced bone adipogenesis and other secondary complications.

## Introduction

High glucose-induced bone fragility [1] and adipogenesis [2] are the leading secondary complications associated with diabetes, characterized by the loss of bone mineralization [3] and an increase in the adipogenic commitment of precursor mesenchymal stromal cells (MSCs) [2]. Nutrients, especially glucose and glutamine, influence the insulin secretion of pancreatic-β cells, and their imbalance is associated with diabetes and diabetes-related complications [4, 5]. Despite being a major disorder, the molecular mechanisms underlying the biochemistry of nutrient metabolism in diabetes remain elusive, and recent studies are directed towards new dimensions of physiology. It was hypothesized that nutrient regulation can improve diabetes management, but there are many niches to be explored to understand the nutrient control of cell fate and the role of nutrients in diabetes management [6]. mTOR complexes (mTORC1 and mTORC2) are key players in integrating external cues, such as metabolic and nutrient signals, into downstream pathways [7]. mTOR is present as two different multiprotein subunit complexes, namely, mTORC1 (characterized by raptor, PRAS40, deptor, mLST8, and mTOR) and mTORC2 (characterized by rictor, mSIN1, and protor). Growth factor signaling and nutrients activate mTORC1, although little is known about mTORC2 activation [8].

Several studies have revealed that mTORC1 is critical in glucose metabolism, insulin secretion, and energy homeostasis [9]. Hyperactivation of mTORC1 due to chronic conditions, such as chronic high glucose exposure or diabetes, or overexpression of any one of the components of the mTORC1 complex leads to the destruction of β-islet cells, insulin resistance, loss of glucose homeostasis, and obesity [10]. Under similar conditions, mTORC1 hyperactivation is also linked to decreased mTORC2 function and impaired AKT signaling, leading to loss of mitogenic signaling [11].

Glutamine is a potent activator of mTORC1, and its function in promoting adipogenesis was thought to be carried out through hyperactivation of mTORC1 [12]. There are conflicting reports on the involvement of glucose or glutamine in mTORC1 activation since the levels of both seem to be high in diabetes; however, studies by Moloughney et al. and others demonstrated that under glucose-depleted conditions, the presence of glutamine promotes mTORC1 activation, whereas other amino acids did not show much significance. Furthermore, these studies also revealed that mTORC2 was activated under reduced glutamine levels [13, 14].

Diabetes is primarily characterized by the presence of high blood glucose levels, but the plasma glutamine levels of diabetic patients, though ambiguous, tend to be higher than those of their matched controls [15, 16]. High levels of glucose were shown to inhibit osteogenesis mainly through the suppression of RUNX2 levels [17]. Our earlier studies demonstrated that p-AMPK drives MSCs to become osteocytes, whereas under diabetes conditions, the loss of AMPK activity correlates with ubiquitination of RUNX2 and favors adipogenesis. We also demonstrated that metformin attenuates diabetes-induced bone adipogenesis and bone loss in a mouse model, which correlates with epidemiological data indicating that diabetes patients who are on metformin have healthier bones than those on nonmetformin drugs [18]. Recent reports have also shown that mTORC1 is indispensable for insulin-mediated adipogenesis [19–21], whereas mTORC2 seems to be crucial for osteogenesis [22, 23] and high levels of glucose increase the adipogenic commitment of MSCs, hitherto the critical molecular players involved in this cross-talk are unclear [24]. Taking all these factors into consideration, the current study aims to decipher the molecular mechanisms involved in the differential regulation of MSC fate by glucose and glutamine and their roles in the regulation of mTORC1 and mTORC2 signaling and their cross-talk under diabetes conditions.

## Results

### mTORC1, but not mTORC2, is indispensable for adipogenesis and vice versa

**Fig. 1:**
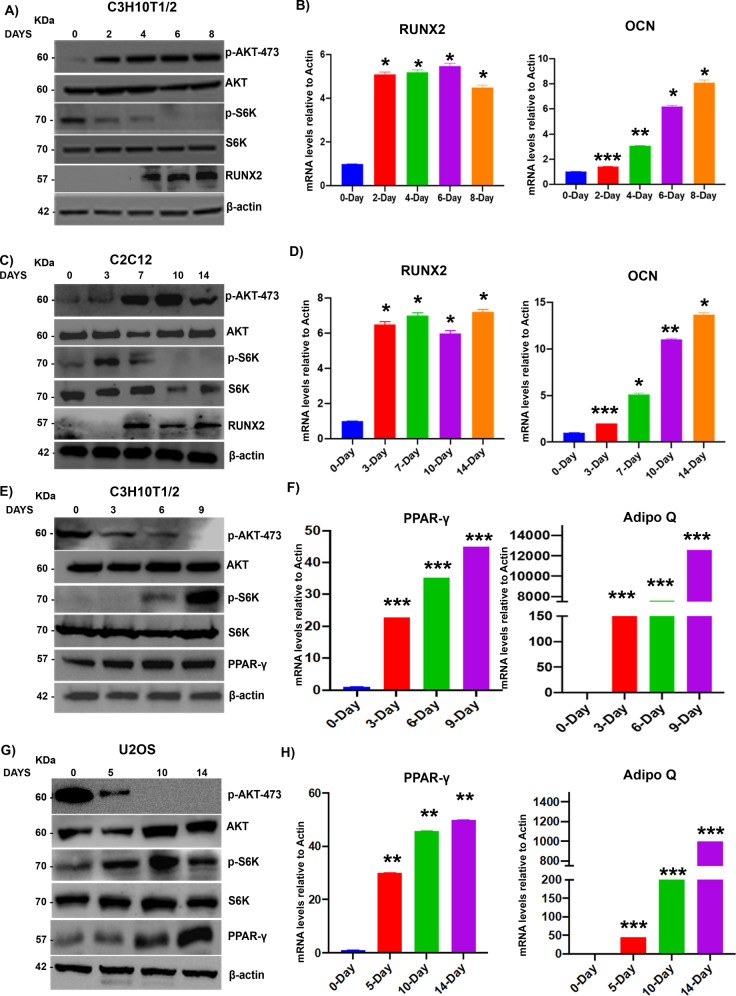
mTORC1, but not mTORC2, is indispensable for adipogenesis and vice versa. Murine MSCs (C3H10T1/2) were subjected to osteogenic differentiation, and lysates were collected at the indicated time points and subjected to **A** Western blot analysis using the indicated antibodies and **B** RT-PCR. **C** C2C12 cells (skeletal myocytes) were subjected to osteogenic transdifferentiation, and cell lysates were subjected to Western blotting and **D** RT-PCR. Murine MSCs were subjected to adipogenic differentiation, and cell lysates were collected at the indicated time points and **E** subjected to Western blotting and **F** RT-PCR as indicated. U2OS cells were subjected to adipogenic transdifferentiation, and cell lysates were collected and **G** subjected to Western blot analysis and **H** RT-PCR analysis as indicated. Mean ± S.E.M.; *N* = 3, *p < 0.1 versus control, **p < 0.01 versus control; ***p < 0.001 versus control. RUNX2 runt-related transcription factor 2, OCN Osteocalcin, Adipo Q adiponectin Q, PPAR-γ peroxisome proliferator-activated receptor gamma.

MSCs have the capacity to give rise to a repertoire of lineages, including osteocytes and adipocytes. Several molecular players, such as RUNX2 (for osteogenesis) and PPAR-γ (adipogenesis), are known to play a critical role in lineage commitment [18, 25]. Here, we first analyzed the activation of mTORC1 and mTORC2 during osteogenic and adipogenic differentiation and transdifferentiation. Our data show that the mTORC2/AKT-473 pathway was activated during C3H10T1/2 (MSC) osteogenic differentiation along with an increase in the levels of RUNX2, whereas mTORC1 activity (as measured by p-70S6K levels) was at the basal level (Fig. 1A) during osteogenic commitment. Osteogenic differentiation was further confirmed by analysis of key regulators, such as RUNX2 and OCN, at the mRNA level (Fig. 1B). Similar results were established in the C2C12 (myoblast) transdifferentiation to osteocyte model (Fig. 1C, D). However, the mTORC1/p-70S6K pathway was activated under C3H10T1/2 to adipogenic differentiation along with an increase in PPAR-γ levels and reduced mTORC2 activity (as measured by p-AKT473 levels) (Fig. 1E). To further confirm the adipogenic differentiation of PPAR-γ, Adipo Q mRNA levels were measured (Fig. 1F). Similar results were established in U2OS transdifferentiation (osteocyte to adipocyte differentiation) (Fig. 1G, H).

### mTORC1 promotes the adipogenic lineage through inhibition of the mTORC2/AKT-473/RUNX2 axis

**Fig. 2:**
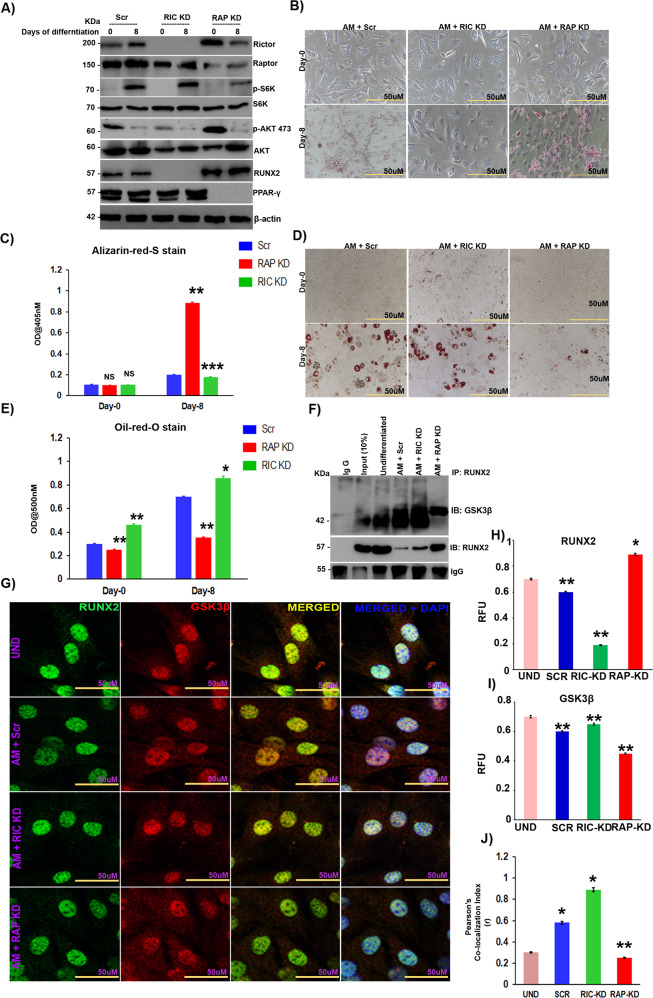
mTORC1 promotes adipogenic lineage through inhibition of the mTORC2/AKT-473/RUNX2 axis. Murine MSCs (C3H10T1/2) were transfected with siRNAs against RICTOR and RAPTOR, 48 hours post-transfection, MSCs were induced to adipogenic differentiation and subjected to **A** Western blot analysis. **B)** Alizarin red S-stained calcium deposition of ECM in murine MSCs (C3H10T1/2) induced adipogenic differentiation post-transfection at the indicated time points and **C** quantification of alizarin red S-stain using Image J. **D** Oil red O-stained murine MSCs that were induced to adipogenic differentiation post-transfection at the indicated time points and **E** quantification of Oil red O-stain using Image J. **F** Immunoprecipitation analysis of transfected murine MSCs (C3H10T1/2) with or without induction of adipogenic differentiation. Complexes with anti-RUNX2 were pulled down and immunoblotted with anti-GSK3β and anti-RUNX2 antibodies. **G** Confocal images of transfected murine MSCs (C3H10T1/2) induced to adipogenic differentiation for 8 days, co-stained with anti-RUNX2 (Alexa 488) and anti-GSK3β (Alexa 594) and counterstained with DAPI (400). Quantification of confocal staining by ImageJ analysis for **H** RUNX2, **I** GSK3β, and **J** Pearson’s correlation index. Mean ± S.E.M.; *N* = 3, *p < 0.1 versus control, **p < 0.01 versus control, ***p < 0.001 versus control. AM adipogenic medium, Scr scrambled siRNA, RAP KD raptor knockdown with siRNA, RIC KD rictor knockdown with siRNA, UND undifferentiated, RFU relative florescence units.

MSC commitment to adipocytes is accompanied by suppression of osteogenic differentiation with distinct diversification processes [26]. Since RUNX2 is the master regulator of osteogenesis, we first analyzed the effects of raptor (for mTORC1 KD) and rictor (for mTORC2 KD) knockdown on RUNX2 expression levels in undifferentiated MSCs. Raptor, but not rictor, knockdown resulted in increased RUNX2 expression, whereas rictor knockdown resulted in a reduction in RUNX2 levels (SF 1A), indicating that mTORC1 negatively regulates RUNX2, whereas mTORC2 seems to positively regulate RUNX2. Next, we checked the importance of raptor and rictor knockdown of mTOR complexes in MSC differentiation. These results suggested that knockdown of raptor resulted in loss of adipogenesis, whereas the loss of rictor enhanced adipogenesis, as shown by Western blot (Fig. 2A), RT-PCR (SF1B-F-E), Alizarin red S staining (Fig. 2B, C) and oil red O (Fig. 2D, E) analyses. Our results demonstrated that mTORC1 knockdown (through anti-raptor siRNA) not only resulted in the suppression of adipogenic differentiation (Fig. 2D) but also increased the levels of RUNX2 (Fig. 2A & SF 1A) protein and RUNX2 downstream signaling targets, such as OCN and ALP (SF 1B & C) [27]; however, there was no effect on RUNX2 transcript levels (SF 1F), indicating posttranslational control of RUNX2 by mTORC1. These effects were accompanied by a gain of osteocyte-like features by MSCs, as shown by RT-PCR (SF1B, C) and Alizarin red staining of differentiated MSCs (Fig. 2B, C), and a reduction in adipogenesis (Fig. 2D, E). Hitherto, mTORC2 knockdown (through siRNA against rictor) resulted in increased adipogenesis, as shown by oil red O staining (Fig. 2D, E), along with loss of RUNX2 (Fig. 2A & SF 1A) and osteocyte-like features by MSCs (SF1B & C) with no effect on RUNX2 mRNA levels (SF1F), indicating possible posttranslational control of RUNX2 by mTORC2. Based on these results, we hypothesized that mTORC1 was involved in the suppression of osteogenesis of MSCs through RUNX2 suppression. Since GSK3β is a well-known regulator of the ubiquitination of several key proteins involved in osteogenesis and acts downstream of the mTORC2/AKT-473 axis and involved in adipogenesis [28], we checked the involvement of GSK3β in the posttranslational regulation of RUNX2 in the differentiation of MSCs. Our studies on the interaction between RUNX2 and GSK3β, by immunoprecipitation of RUNX2 in MSCs followed by mTORC1 and C2 knockdown, demonstrated that the interaction between RUNX2 and GSK3β was reduced upon knockdown of mTORC1, whereas the same was enhanced upon mTORC2 knockdown (Fig. 2F). Similar results were confirmed by confocal analysis (Fig. 2G–J).

### mTORC2 stabilizes RUNX2 through the AKT-473/ GSK3β axis

**Fig. 3:**
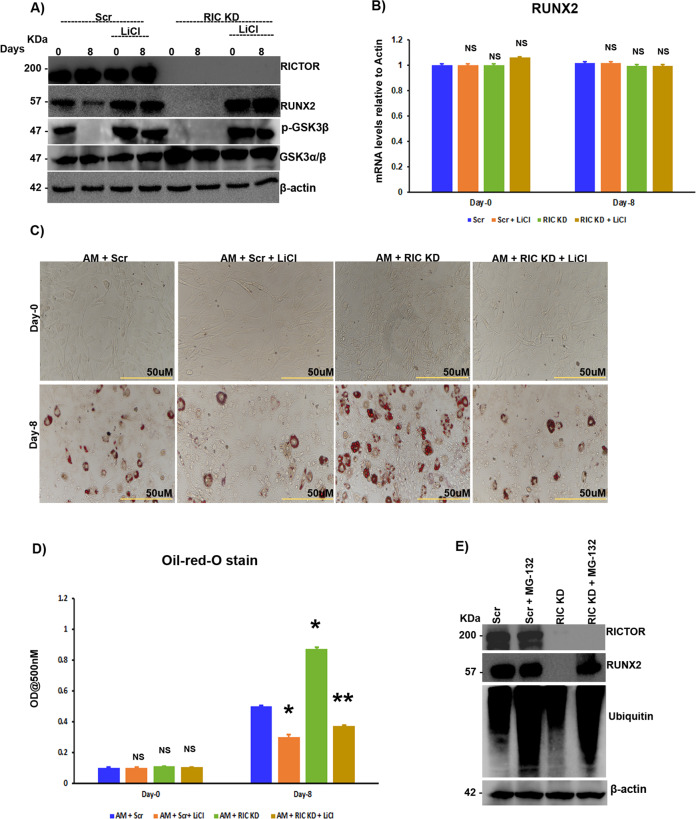
mTORC2 stabilizes RUNX2 through the AKT-473/GSK3β axis. Murine MSCs (C3H10T1/2) were treated with LiCl (0.5 mM) with or without siRNA against rictor, along with adipogenic induction and **A** subjected to Western blot analysis and **B** RT-PCR as indicated. **C** Oil red O-stained images of murine MSCs (C3H10T1/2) treated with LiCl (0.5 mM) with or without siRNA against rictor, along with adipogenic induction as indicated and **D** quantification of oil red O staining by spectrophotometry. Murine MSCs (C3H10T1/2) were transfected with siRNA against rictor in the presence or absence of MG-132 for 48 h after transfection, and **E** Western blot analysis was performed after 48 h with the indicated antibodies. Mean ± S.E.M.; *N* = 3, *p < 0.1 versus control, **p < 0.01 versus control, ^NS^*p* > 0.1 versus control. AM adipogenic medium, Scr scrambled siRNA, RIC KD rictor knockdown with siRNA, LiCl lithium chloride, NS not significant.

As mTOR complexes appear to regulate RUNX2 stabilization through GSK3β, we investigated the role of GSK3β in RUNX2 regulation during adipogenic differentiation. MSCs were subjected to mTORC2 knockdown in the presence or absence of LiCl (a GSK3β inhibitor) and primed for adipogenesis. LiCl treatment rescued the mTORC2 knockdown-mediated loss of RUNX2 expression (Fig. 3A) without altering RUNX2 mRNA levels (Fig. 3B). Since mTORC2 knockdown resulted in increased adipogenesis, we analyzed whether this increase in adipogenesis was indeed mediated through GSK3β. Our differentiation model in MSCs confirmed that upon treatment with LiCl, there was a decrease in adipogenesis due to the suppression of GSK3β (Fig. 3C, D). Since posttranslational regulation of RUNX2 was obvious and it is known that GSK3β regulates several key signaling molecules by ubiquitination, we analyzed the role of GSK3β in the proteasomal degradation of RUNX2. Our experiments in MSCs with or without MG-132 treatment (an inhibitor of proteasomal degradation) and with or without mTORC2 knockdown demonstrated that the loss of RUNX2 protein levels upon mTORC2 knockdown was reduced upon exposure to MG-132. These results, along with the interaction of RUNX2 and GSK3β, indicate that GSK3β is involved in RUNX2 ubiquitination (Fig. 3E).

### Diabetes-induced RUNX2 loss is mediated through the mTORC1/p-70S6K/GSK3β axis

**Fig. 4:**
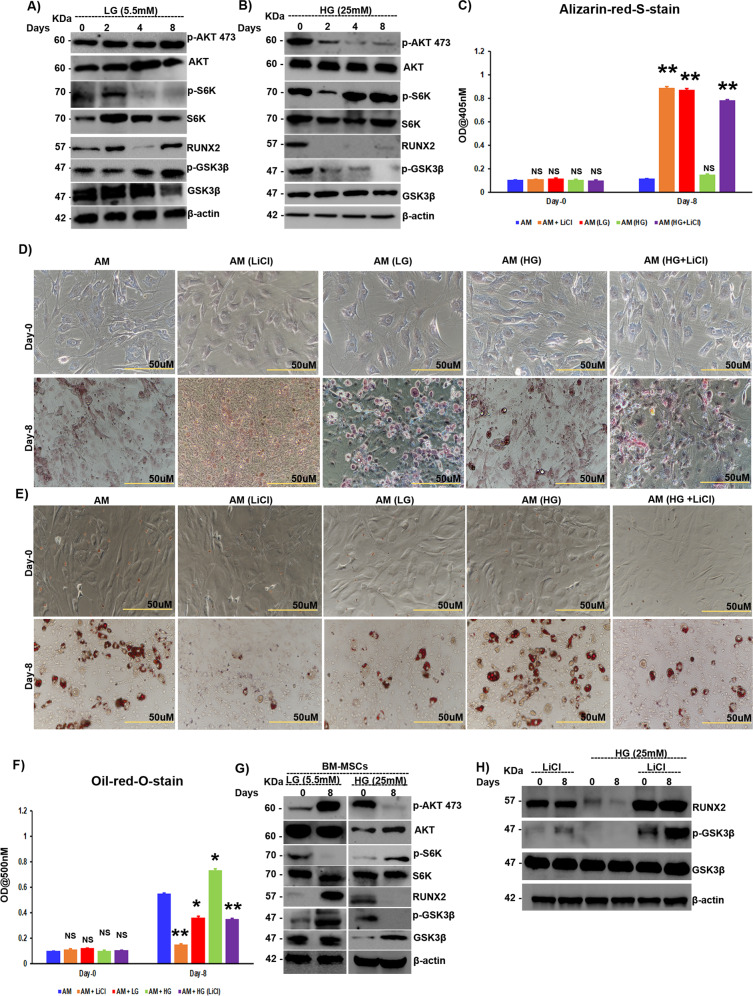
Diabetes-induced RUNX2 loss is mediated through the mTORC1/p-70S6K/GSK3β axis. Murine MSCs (C3H10T1/2) were subjected to adipogenic differentiation in the presence of either low (5.5 mM) or high (25 mM) levels of glucose in the medium and subjected to **A**, **B** Western blot analysis as indicated. **C** Quantification of alizarin red-S staining by spectrophotometry and **D** Alizarin red-S-stained images of murine MSCs (C3H10T1/2), which were induced to adipogenic differentiation with either low (5.5 mM) or high (25 mM) levels of glucose and with or without LiCl (0.5 mM) treatment, as indicated. **E** Oil red O-stained murine MSCs (C3H10T1/2), which were subjected to adipogenic differentiation in the presence of either low (5.5 mM) or high (25 mM) levels of glucose and with or without LiCl (0.5 mM) treatment, as indicated, and **F** quantification of oil red O staining by spectrophotometry. **G** Bone marrow-derived MSCs (BM-MSCs) were subjected to adipogenic differentiation in the presence of either low (5.5 mM) or high (25 mM) levels of glucose, and cell lysates were collected and subjected to Western blot analysis as indicated. Murine MSCs (C3H10T1/2) treated with LiCl (0.5 mM) with or without treatment with high glucose (25 mM) and **H** Western blot analysis, as indicated. Mean ± S.E.M.; *N* = 3, *p < 0.1 versus control, **p < 0.01 versus control, ^NS^*p* > 0.1 versus control. AM adipogenic medium, LG low glucose, HG high glucose, LiCl lithium chloride, BM-MSCs bone marrow-derived mesenchymal stromal cells freshly isolated from mice, NS not significant.

Since our data suggest that mTORC1 and C2 regulate RUNX2 ubiquitination through GSK3β, we then asked whether RUNX2 is ubiquitinated by the mTOR-GSK3β axis under diabetic conditions. To test this hypothesis, C3H10T1/2 cells were grown in high- and low-glucose conditions, and the activity of mTORC1 (measured through S6K phosphorylation status) and mTORC2 (measured through AKT-473 phosphorylation) was analyzed. Under low-glucose conditions (which mimic starved physiological conditions), mTORC1 activity is reduced, while mTORC2 activity is increased, resulting in inhibition of GSK3β through Ser9 phosphorylation, thus stabilizing RUNX2 levels (Fig. 4A). However, under high-glucose conditions (which correlates with physiological diabetic conditions), inhibition of mTORC2 activity was observed. Low levels of pAKT-473 led to GSK3β activation, resulting in low levels of RUNX2 (Fig. 4B). Similar to RUNX2 protein levels under high-glucose conditions, there was a decrease in the ECM calcification of MSCs, which was increased under low-glucose and GSK3β-inhibited conditions (Fig. 4C, D). Owing to the high mTORC1 activity under high-glucose conditions, there was an increase in the adipogenesis of MSCs, whereas under low-glucose conditions, mTORC1 activity was reduced, resulting in inhibition of GSK3β and stabilization of RUNX2, which resulted in decreased adipogenesis (Fig. 4E, F). Similar results were recapitulated in experiments with primary bone marrow-MSCs (BM-MSCs) (Fig. 4G). To delineate the role of GSK3β in high glucose-triggered RUNX2 loss, we next inhibited GSK3β in MSCs with LiCl and subjected MSCs to adipogenic differentiation under normal and high glucose conditions. The results showed that the loss of RUNX2 protein levels observed under high-glucose conditions was attenuated when MSCs were exposed to LiCl (Fig. 4H). Similarly, there was an increase in ECM calcification (Fig. 4C, D) and a decrease in adipogenesis (Fig. 4E, F) in the presence of LiCl.

### High glucose-induced glutamine sparing triggers RUNX2 loss under diabetic conditions

**Fig. 5:**
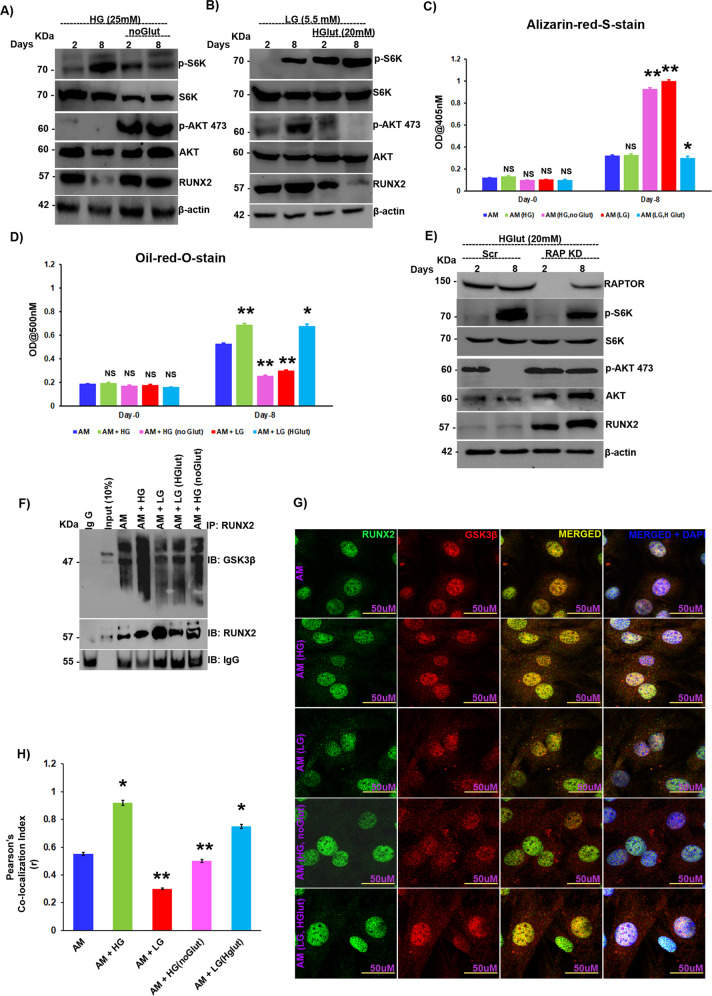
High glucose-induced glutamine sparing triggers RUNX2 loss under diabetic conditions. Murine MSCs (C3H10T1/2) were subjected to adipogenic differentiation **A** in the presence of high glucose (25 mM) with or without glutamine (4 mM) and **B** in the presence of low glucose (5.5 mM) with or without high glutamine (20 mM) followed by Western blot analysis at the indicated intervals. **C** Quantification of alizarin red S staining by spectrophotometry. **D** Quantification of oil red O staining by spectrophotometry. Murine MSCs (C3H10T1/2) were subjected to adipogenic differentiation in the presence of high glutamine (20 mM) with or without raptor siRNA, and cell lysates were subjected to **E** Western blot analysis as indicated. **F** Immunoprecipitation analysis of murine MSCs (C3H10T1/2) induced to adipogenic differentiation under varying glucose and glutamine concentrations. Anti-RUNX2 complexes were pulled and immunoblotted with anti-GSK3β and anti-RUNX2 antibodies. **G** Confocal images of murine MSCs (C3H10T1/2) induced to adipogenic differentiation under varying glucose and glutamine concentrations for 8 days, stained with anti-RUNX2 (Alexa 488) and anti-GSK3β (Alexa 594) and counterstained with DAPI (400). **H** Quantification of confocal images by ImageJ software. Mean ± S.E.M.; *N* = 3, *p < 0.1 versus control, **p < 0.01 versus control; ^NS^*p* > 0.1 versus control. Glut glutamine, HGlut high glutamine, AM adipogenic medium, LG low glucose, HG high glucose, IP immunoprecipitation, IB immunoblotting, NS not significant.

Under high-glucose conditions, mTORC1 is hyperactivated, which results in feedback inhibition of mTORC2 through p-70S6K and finally a loss of RUNX2 protein levels; however, the mechanism behind hyperactivation of mTORC1 with increasing glucose concentrations is unclear. Recent studies have shown that under high-glucose conditions, glutamine sparing from mitochondria increases mTORC1 activity [29–31]. Therefore, we hypothesized that a similar phenomenon could also occur in the context of diabetes. To test our hypothesis, we subjected MSCs to adipogenic differentiation under high-glucose conditions with or without glutamine. We found that upon withdrawal of glutamine, even in the presence of high glucose, mTORC1 hyperactivation was lost, as was inhibition of the mTORC2/AKT-473 axis, resulting in a resurrection of RUNX2 levels that were otherwise repressed (Fig. 5A). The role of glutamine in RUNX2 regulation was further established by treating MSCs with low levels of glucose along with high or normal levels of glutamine (high levels of glutamine were determined by measuring the phosphorylation status of p70S6K under varying levels of glutamine in adipogenic differentiation medium). mTORC2 activity was aborted upon the addition of high glutamine levels due to the hyperactivation of mTORC1 by glutamine and culminated in a loss of RUNX2 expression (Fig. 5B), along with a reduction in ECM calcification (Fig. 5C & SF2A) and an increase in adipogenesis (Fig. 5D & SF2B). The high glucose-primed increase in adipogenesis was also subdued upon withdrawal of glutamine (Fig. 5D & SF2B), and the reverse was seen in the case of ECM calcification (Fig. 5C & SF2A). To confirm that glutamine’s action indeed occurred through mTORC1, MSCs were subjected to mTORC1 knockdown and then exposed to high glutamine concentrations, which resulted in activation of the mTORC2/AKT-473 axis and subsequently stabilized RUNX2 levels (Fig. 5E), indicating that glutamine indeed acts through mTORC1. In our earlier section, we observed that RUNX2 loss was due to increased physical interaction between GSK3β and RUNX2, so the same was examined under varying glutamine and glucose concentrations. Immunoprecipitation by RUNX2 and immunoblotting with GSK3β in MSCs revealed elevated levels of interaction between RUNX2 and GSK3β under high-glutamate conditions (Fig. 5F). The same results were confirmed by immunofluorescence with confocal microscopy (Fig. 5G, H).

### Metformin rescues the mTORC2/RUNX2 axis by inhibiting the mTORC1/p-70S6K pathway

**Fig. 6:**
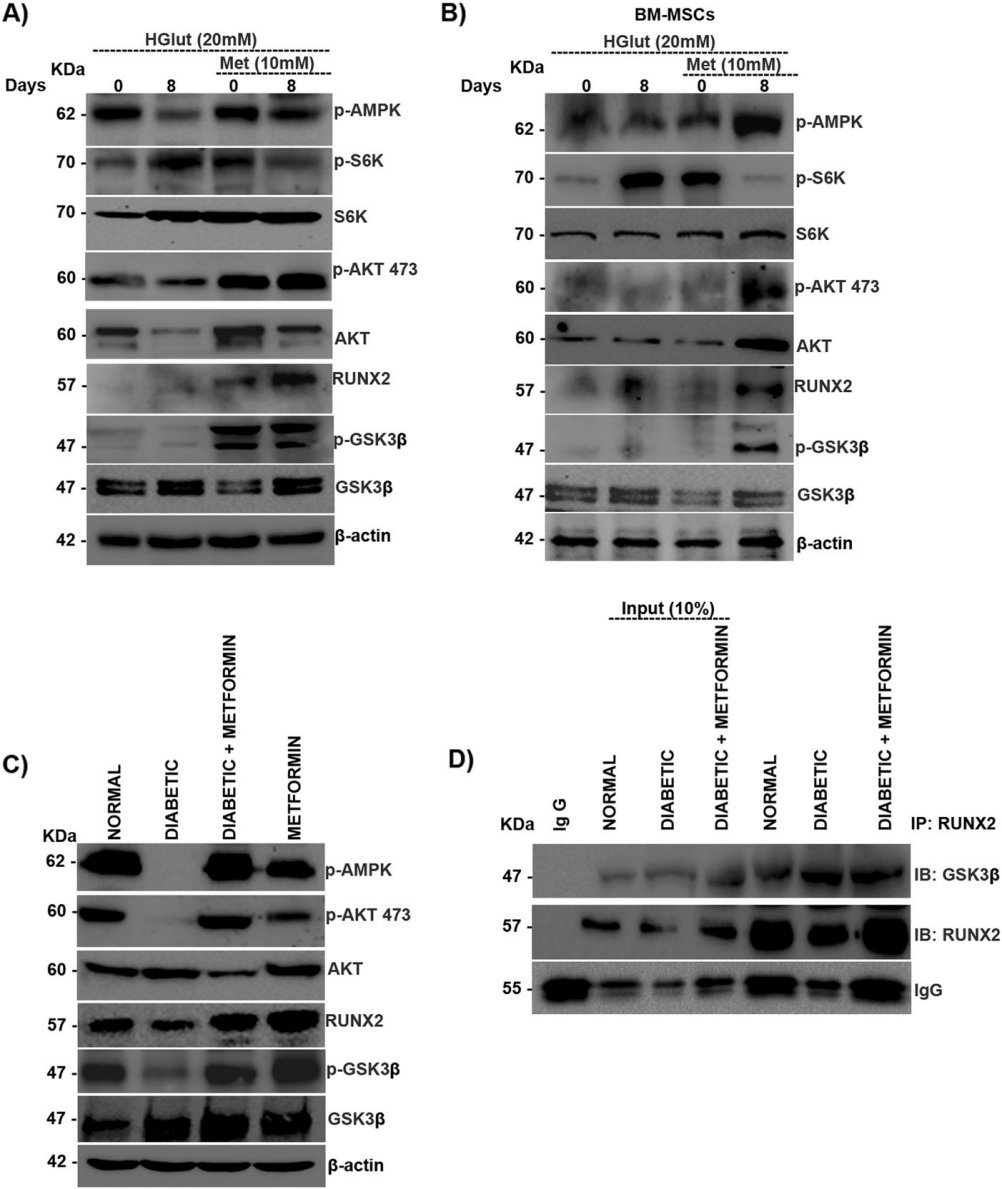
Metformin rescues the mTORC2/RUNX2 axis by inhibiting the mTORC1/p-70S6K pathway. **A** Murine MSCs (C3H10T1/2) and **B** BM-MSCs were subjected to adipogenic differentiation in the presence of high glutamine (20 mM) with or without metformin (10 mM) and subjected to Western blot analysis as indicated. Normal and diabetic BALB/c male mice, with and without metformin treatment, were sacrificed, and muscle tissue was excised from the hind limbs and subjected to **C** Western blot analysis with anti-GSK3-β and anti-RUNX2 and **D** immunoprecipitation with anti-RUNX2. Mean ± S.E.M.; *N* = 3. IP immunoprecipitation, IB immunoblotting, HGlut high glutamine, Met metformin, BM-MSCs bone marrow-derived mesenchymal stromal cells freshly isolated from mice.

Metformin is also known to have osteoprotective functions through the p-AMPK/RUNX2 axis [18]. Here, we examined the effects of metformin under high-glutamine conditions in MSCs. Metformin treatment abrogated glutamine-triggered mTORC1 activation, resulting in increased mTORC2 activity and stabilizing the AKT-473/RUNX2 axis (Fig. 6A). The inhibition of glutamine-induced mTORC1 activation by metformin was further validated in BM-MSCs, whose results were the same as those seen in MSCs (Fig. 6B). The role of the mTORC2/GSK3β/RUNX2 axis was further analyzed in streptozotocin-induced diabetic mice with or without metformin treatment. mTORC2 activity was downregulated under diabetic conditions and was rescued when treated with metformin. Under diabetic conditions, GSK3β was active due to mTORC2 inhibition, resulting in downregulation of RUNX2, and the situation was reversed upon metformin treatment (Fig. 6C). The interaction between RUNX2 and GSK3β was confirmed in protein lysates of diabetic muscle tissues, where the interaction was greater in diabetic samples than in controls and metformin-treated diabetic tissues. These results indicate that under diabetic conditions, the interaction between RUNX2 and GSK3β was enhanced due to a loss of mTORC2 regulation of GSK3β, which was reversed upon metformin treatment (Fig. 6D).

## Discussion

The lineage commitment of MSCs is a dynamic process carried out under the tight regulation of growth factors, hormones, and available nutrients [32]. Recently, the role of nutrients such as glucose and glutamine in MSC regulation has gained significance owing to the activation of mTORC1 and mTORC2 [23]. The current study reveals the role of nutrients in activating mTORC1/p-70S6K-induced adipogenesis while inhibiting osteogenesis, the role of mTORC2/AKT-473 in maintaining osteogenesis, and the impact of negative regulation of mTORC2 by mTORC1 levels and increased adipogenesis of MSCs. Both mTOR complexes exerted their effects in part through RUNX2 regulation, where mTORC2 stabilizes RUNX2 by inhibiting GSK3β, which would otherwise induce RUNX2 ubiquitination. On the other hand, mTORC1 hyperactivation by insulin or glutamine resulted in activation of GSK3β (due to the loss of inhibitory phosphorylation) by inhibiting mTORC2, which culminated in the repression of RUNX2 and osteogenic commitment of MSCs.

Diabetes-induced high glucose levels are known to downregulate RUNX2; however, the mechanisms involved are not clear [18]. From our studies, it was observed that mTORC2 was inhibited under diabetic conditions due to hyperactivation of mTORC1, and as a result, RUNX2 was suppressed. However, glucose as such has no direct role in activating mTORC1. Therefore, one of the possible mechanisms that can explain the above phenomenon is that at higher concentrations, glucose spares glutamine entry into the tricarboxylic acid (TCA) cycle, which can result in high glutamine levels inside the cell [30]. The crucial step for glutamine entry into the mitochondrial TCA cycle is the conversion of glutamine to glutamate, catalyzed by glutamine synthetase (GLS) [33]. Active GSK3β can inhibit GLS by inhibiting c-MYC, which otherwise would activate glutamine oxidation by upregulating GLS expression [34]. Inhibition of GLS by GSK3β results in increased intracellular glutamine levels and can potentiate the hyperactivation of mTORC1, which would result in adipogenesis. In parallel, GSK3β also ubiquitinates rictor, thus resulting in a loss of mTORC2 and inhibition of osteogenesis [35].

Active mTORC2 favors osteogenesis by inhibiting GSK3β through AKT-473, which results in the stabilization of RUNX2. Inactive GSK3β activates GLS and glutamine oxidation in cells, which could result in low intracellular glutamine levels, downregulating mTORC1 and adipogenesis. Active GSK3β can induce RUNX2 ubiquitination and downregulate osteogenesis. Notably, mTORC2 is also involved in the regulation of AKT ubiquitination through Ser473 phosphorylation [36]. Our data showed that low levels or the absence of glucose and glutamine, respectively, could activate mTORC2, which then paved the way for the osteocyte-like signature of MSCs, even in the absence of osteogenic-inducing medium (OM). This sheds light on the importance of cross-talk between mTOR complexes in MSC fate regulation.

In this study, metformin, through activation of p-AMPK, repressed mTORC1 hyperactivation under high glutamine conditions and thus activated the mTORC2/AKT-473/RUNX2 axis. p-AMPK inhibits mTORC1 activation by phosphorylating raptor, which interferes with mTORC1 complex assembly [37]. Activated AMPK can also alter glutamine oxidation through mitochondria [38], thus affecting intracellular glutamine levels and mTORC1 activation. Diabetic patients were shown to have reduced glutamine oxidation and low levels of GLS activity [39], which can induce high intracellular glutamine levels and result in hyperactivation of mTORC1 and inhibition of mTORC2, resulting in bone adipogenesis. Our work showed that upon treatment with metformin, glutamine-induced mTORC1 hyperactivation was subdued, resulting in rescue of the mTORC2/AKT-473 axis. Activation of mTORC2 by metformin through AMPK resulted in the activation of AKT, which inhibits GSK3β, due to which RUNX2 was rescued from ubiquitination. Metformin, by activating AMPK and inhibiting GSK3β, could modulate the intracellular levels of glutamine through GLS.

**Fig. 7:**
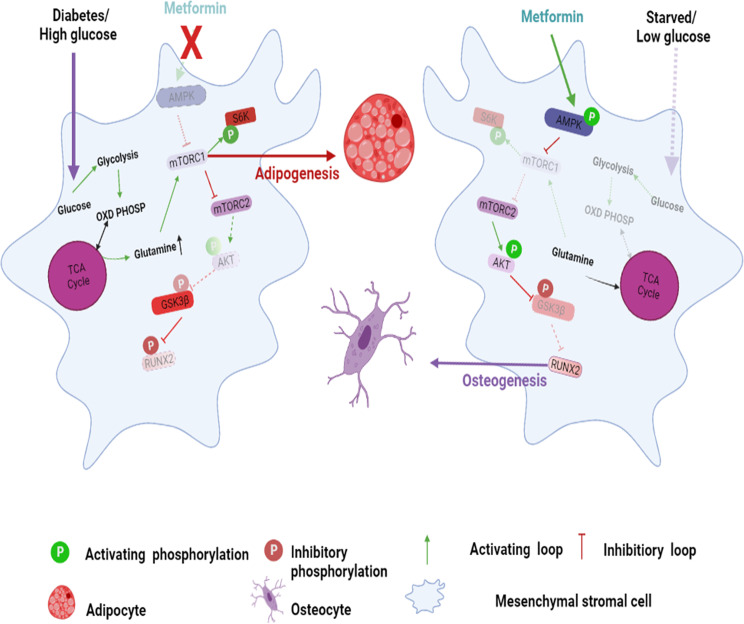
Schematic representation of mTOR crosstalk in RUNX2 regulation. This figure summarizes, along with the findings from the current study, where, at physiological levels, activation of mTORC2 by PIP2 resulted in activation of AKT by phosphorylation at Ser473. pSer473AKT inhibits GSK3β by phosphorylation at Ser9. Inhibitory/inactive Ser-9GSK3β fails to interact with RUNX2, and thus RUNX2 is stabilized from its degradation. Active RUNX2 can enhance ECM calcification and promote osteogenesis of MSCs. Increasing levels of glucose and glutamine or insulin can hyperactivate mTORC1 which through active p-70S6k inhibits mTORC2. Inhibition of mTORC2 results in destabilization of RUNX2 through the loss of pSer473AKT, which leads to activation of GSK3β. Active GSK3β interacts with RUNX2 and primes it for ubiquitination. mTORC1 also activates lipogenesis and helps to increase adipogenesis. Metformin activates AMPK by phosphorylation at Thr172, which inhibits mTORC1 and rescues mTORC2 under high-glucose and/or high-glutamine conditions.

Taken together, our data indicate that RUNX2 is stabilized directly by the mTORC2/AKT-473 axis by inhibiting GSK3β. Under an excess of nutrients such as glutamine and/or glucose, mTORC1 is hyperactivated, resulting in activation of GSK3β, which ubiquitinates RUNX2 and suppresses the osteogenic fate of MSCs, leading to bone loss and adipogenesis. The current work emphasizes the important cross-talk between mTOR complexes in directing MSC fate under normal and diabetic conditions (Fig. 7).

## Material and methods

### Cell culture and chemicals

C3H10T1/2 (Cat. No-CCL-226), hereafter called murine MSCs, C2C12 (Cat. No-CRL-1772) (murine skeletal muscle cells), and U2OS (Cat. No-HTB-96) (human bone osteosarcoma epithelial cells) were procured from ATCC (USA). All the cell lines, including BM-MSCs, were maintained in IMDM (Cat. No-12200069) (Gibco, USA) supplemented with 10% FBS (Cat. No-10082147) (Gibco, USA) and 1% pen-strep (Cat. No-1514022) (Gibco, USA) in 5% CO_2_ incubator at 37 ^o^C. For the glucose and glutamine treatments, media without glucose or glutamine (Cat. No-A1443001) (Gibco, USA), respectively was used. MG-132 (Cat. No-M8699), L-ascorbic acid (Cat. No-A92902), dexamethasone (Cat. No-D4902), 3-isobutyl-1- methylxanthine (IBMX) (Cat. No-I5879), rosiglitazone (Cat. No-R2408), metformin (Cat. No-1396309), glucose (Cat. No-1181302), oil-red-o (Cat. No-O0625), alizarin-red-s (Cat. No-A5533), β-glycerophosphate (Cat. No-G9422), and LiCl (Cat. No-L9650) were purchased from Sigma (USA). Insulin (Cat. No-12585014), L-glutamine (Cat. No-25030081) and Human BMP-2 (Cat. No-#PHC7141) were purchased from Gibco (USA). BM-MSCs from BALB/c male mice, 6-8 weeks of age, were isolated and characterized by florescence-assisted cell sorting using CD44^+^, CD90^+^ and CD45^−^ as markers. A more detailed information about BM-MSCs isolation and characterization was previously described in our earlier paper [18]. The cells were maintained in IMDM supplemented with 1% minimum essential amino acids (Cat. No-11140050) (Gibco, USA) acids for 5 days. The medium was changed every alternative day and subcultured using 0.25% trypsin-EDTA (Cat. No-25200056) (Gibco, USA).

### Ethics statement

All experiments involving animals were conducted according to the ethical policies and procedures approved by the ethics committee of the Institutional Animal Ethics Committee (IAEC)-University of Hyderabad, India (Approval no. IAEC/UH/151/2016/11/BMR/P3).

### Streptozotocin-induced diabetic model

Male BALB/c mice, 6-7 weeks of age, were used for the study as previously described by adhering to norms instituted by the Institutional Animal Ethics Committee-University of Hyderabad, governed by the CPCSEA-Govt of India. Diabetes was induced by low doses of streptozotocin (Cat. No-S0130) (Sigma, USA) in citrate buffer pH 4.5 given for 5 days and the control group were administered with citrate buffer alone. At the fifth day the blood glucose of mice was estimated using glucometer and mice having blood glucose greater than 300 mg/dL were considered diabetic [18]. Mice were sacrificed at 10 weeks of age after confirmation of diabetes. Metformin control and treated mice were given 60 mg/kg body weight metformin daily (intraperitoneal) for 10 weeks.

### Differentiation and transdifferentiation protocols

Murine-MSCs and BM-MSCs were differentiated into adipocytes by treating them with 0.5 mM IBMX, 20 nM insulin, and 0.1 µM dexamethasone, along with IMDM medium and 10% FBS, for 48 hours. After 48 hours, the cells were maintained for an additional 8 to 21 or 16 days in growth medium supplemented with 20 nM insulin. The medium was changed on alternate days. Murine-MSCs were differentiated into osteocytes using 0.5 mM IBMX, 0.1 µM dexamethasone, and 1 mM L-ascorbic acid, along with IMDM medium and 10% FBS, for 7 days or until they reached 80% confluence. On the 7^th^ day, 1 mM β-glycerophosphate was added and maintained for 21 days; the medium was changed on alternate days. C2C12 transdifferentiation to the osteogenic lineage was performed using BMP-2 at a concentration of 200 ng/mL in growth medium, along with 10% FBS, and maintained for 14 days with a change in medium every 48 hours. U2OS cells were transdifferentiated into adipocytes using 1 µM rosiglitazone in growth medium, along with charcoal-stripped FBS (Cat. No-#12676029) (Gibco, USA), for 14 days.

### Oil- red-o staining

Oil-red-o stock of 0.5% was prepared in isopropanol (Cat. No-91390LC250) (Finar, India), from which a working solution was made in distilled water at a 6:4 ratio. After differentiation, the medium was aspirated, and the cells were washed with 1X phosphate-buffered saline (PBS) and fixed in 10% formalin (Cat. No-10710LC500) (Finar, India) for 1 hour at room temperature, followed by another 1X PBS wash. After the wash, the cells were incubated in 60% isopropanol for five minutes, followed by staining with the oil red O working solution for five minutes. The excess stain was removed by washing with water 3-4 times and visualized under a microscope. Quantification was performed by eluting oil red O in isopropanol, and the absorbance was measured at 500 nM.

### Alizarin-red-s staining

Alizarin red S stain (2%) was prepared in water by adjusting the pH to 4.1 with an ammonium hydroxide solution, and then the stain solution was filtered. After differentiation, cells were washed twice with 1X PBS and fixed in 10% formalin (Cat. No-10710LC500) (Finar, India) for 1 hour at room temperature, followed by another 1X PBS wash. Afterward, the cells were stained with alizarin red S for 45 min, washed with water 3-4 times and visualized under a microscope. Quantification was performed by adding 10% acetic acid (Cat. No-96110LL025) (Finar, India) to each well and incubated for 30 minutes. Cells were then scraped and vortexed for 30 s followed by brief heating at 85 °C. Afterward, they were incubated on ice for 5 minutes, followed by centrifugation at 14,000 rpm for 20 min. For the absorbance measurement, 200 µL of 10% ammonium hydroxide (Cat. No-221228) (Sigma, USA) was added to 500 µL of supernatant, and the absorbance was measured at 405 nM.

### siRNA transfection

The siRNAs for rictor (SI05109048) and raptor (SI00698677) were purchased from Qiagen (Netherlands). All transfections were carried out using RNAifect (Cat. No- #301005) (Qiagen, Netherlands) following the manufacturer’s instructions. In brief, 1 µg of siRNA and 3 µL of RNAifect were diluted in 200 µL of plain DMEM (0.5% FBS) individually and incubated for 5 minutes; later, both were mixed and incubated for 30 minutes before adding the combined solution to cells. Cells were replaced with fresh regular medium after 6 hours of transfection.

### RNA isolation and real-time PCR (RT-PCR)

For the RT-PCR analysis, total RNA isolation was carried out using TRIzol (Cat. No- 15596026) (Thermo Fisher Scientific, USA) method. Equal amounts of RNA (1 µg) were taken, and cDNA synthesis and RT-PCR were carried out as described previously [40]. The sequences of the primer sets used in this manuscript were described previously [18]. The quantification of real-time data was carried out by the ΔΔCT method.

### Immunoprecipitation and Western blotting

Cells were lysed in 1X RIPA buffer (with protease (Cat. No- P8849) and phosphatase (Cat. No- P044) inhibitor cocktails (Sigma, USA)), and for immunoprecipitation, cells were lysed in Thermo IP lysis buffer (Cat. No- 87787) (Thermo Scientific, USA) supplemented with protease (Cat. No- P8849) and phosphatase (Cat. No- P044) inhibitor cocktails (Sigma, USA). Equal amounts of protein (500 µg) were incubated with α-RUNX2 (Cat. No- #8486) (Cell Signaling, USA) at 4 ^o^C overnight. Protein A/G Plus- agarose beads (2.5 mg/reaction) (Cat. No- Sc-2003) (Santa Cruz, USA) were used to pull down the immunoprecipitated protein complexes, and the latter were subjected to Western blotting. The antibodies used for Western blotting were p-P70S6K (Cat. No- #9205), P70S6K1/2 (Cat. No- #9202), α-RUNX2 (Cat. No- #8486), α-p-AMPK (Cat. No- #2523), raptor (Cat. No- #2280), rictor (Cat. No- #2114), p-AKT (473) (Cat. No- #9217) and AKT (Cat. No- #6703) were procured from Cell Signaling Technologies (USA); GSK3α/β (Cat. No- sc-7291) and β-actin (Cat. No- sc-47778) were procured from Santa Cruz Biotechnology Inc. (USA); p-GSK3β (Ser 9) (Cat. No- ab107166) and PPAR-γ (Cat. No- ab272718) were purchased from Abcam (USA). Corresponding HRP (horseradish peroxidase enzyme)-linked secondary antibodies were purchased from GeNeI labs (India) for western blotting and for immunoprecipitation TrueBlot secondary antibodies (Cat. No- #18-8817-33, #18-8816-33) from Rockland Immunochemicals (USA) were used. The signal was detected by Clarity Western ECL blotting substrates (Cat. No- 1705060, 1705062) (Bio-Rad, USA), and images were processed using a Bio-Rad Chemidoc MP system.

### Confocal microscopy

Cells were grown to confluence (80%) on coverslips, treated for the indicated time points, and washed in 1X PBS prior to fixation in 4% formalin for 10 min at room temperature. Cells were stained with α-RUNX2 and GSK3α/β, followed by the respective fluorescence-tagged secondary antibody for staining (Alexa Fluor 488 (Cat. No- #A-11094) and 594 (Cat. No- #A-11005), Invitrogen, USA). Cells were counterstained with 4’,6-diamino-2-phenylindole (DAPI) (Cat. No- P36935) (Thermo Scientific, USA) for nuclei, and images were captured using a laser scanning confocal microscope (LSM 780, Carl Zeiss, Germany).

### Statistical analysis

All data points are represented as mean ± SEM. Statistical analysis was performed with Student’s t-test by comparing the differences between mean values of controls with the experimental sets individually. P values less than 0.1 were considered statistically significant. A minimum set of two to three independent experiments was carried out for all the in vitro studies using cell lines except freshly isolated BM-MSCs and immunoprecipitation experiments due to limitations in the use of animals, whereas each mouse was considered one subject for the in vivo experiments.

## Supplementary information

Supplementary Figure-1

Supplementory Figure-2

Original Data File

## Data Availability

All the data is available in main text or in supplementary material as stated in the text.
