# Supplementary figures and images for: High glutamine suppresses osteogenesis through mTORC1-mediated inhibition of the mTORC2/AKT-473/RUNX2 axis
Source: Cell Death Discov. 2022 Jun 7;8:277. doi: 10.1038/s41420-022-01077-3 (PMC9174279; doi:10.1038/s41420-022-01077-3)

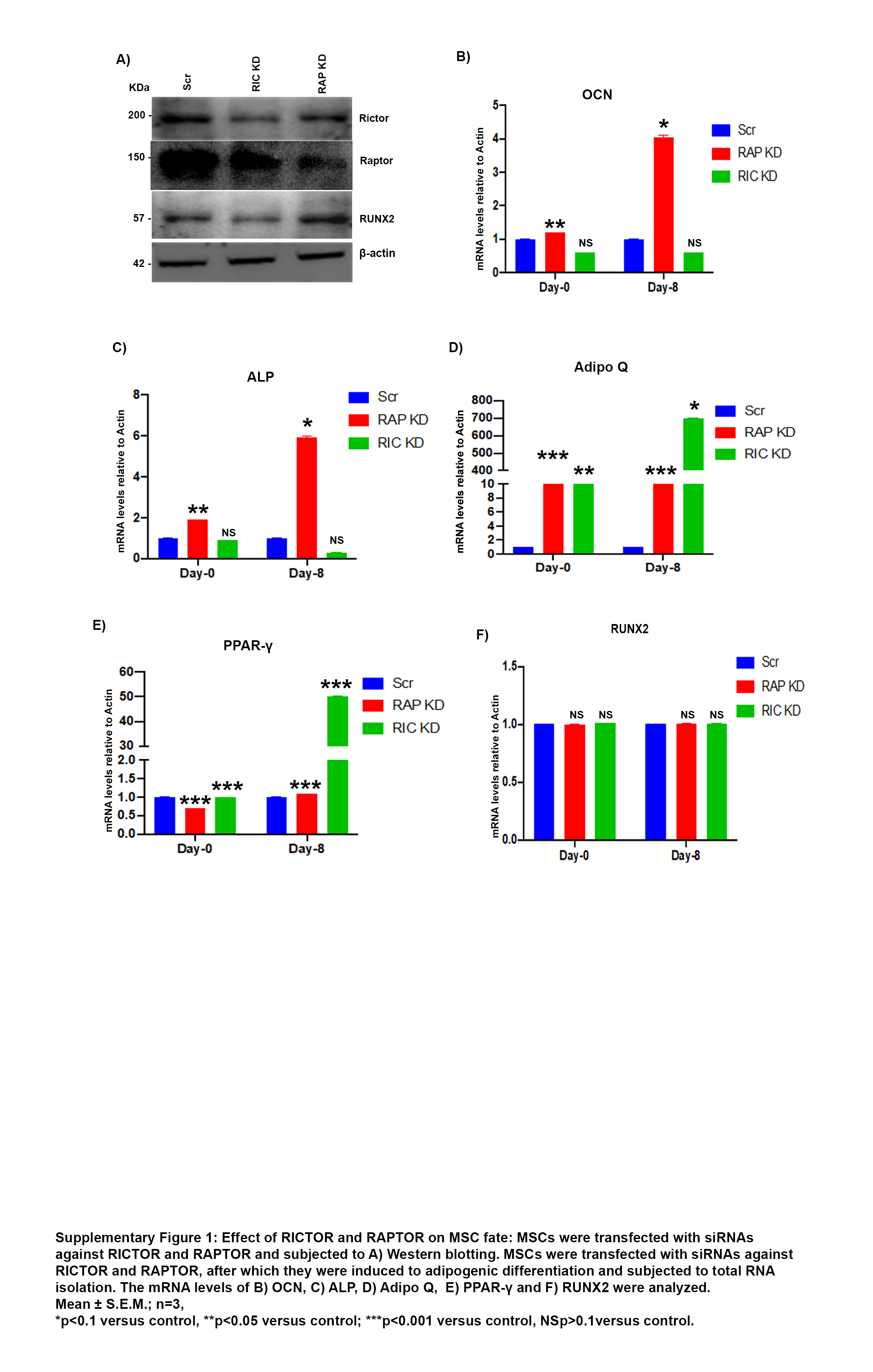

Supplement: Supplementary file 1 — Supplementary Figure-1 [file 41420_2022_1077_MOESM1_ESM.tif]

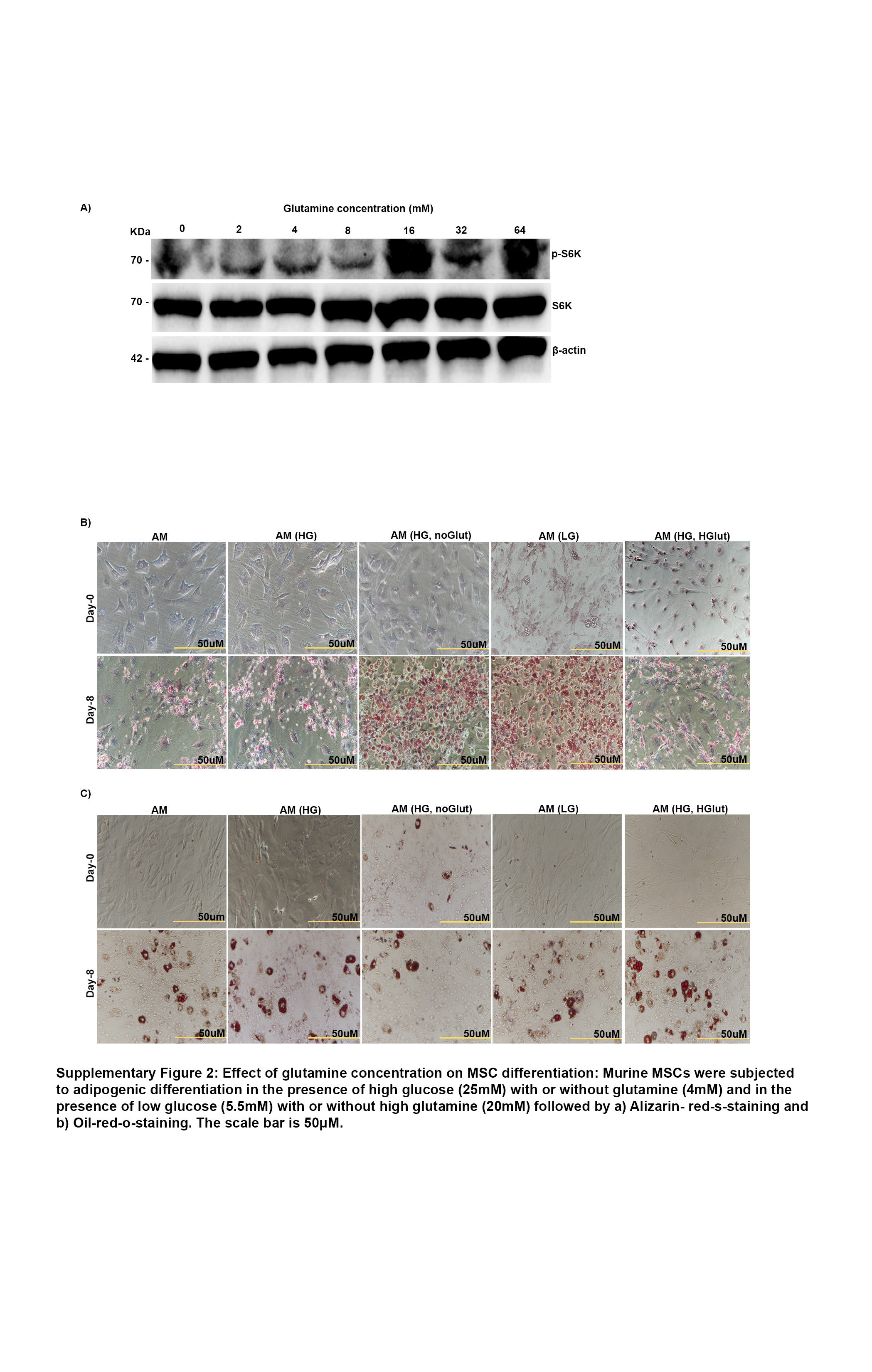

Supplement: Supplementary file 2 — Supplementory Figure-2 [file 41420_2022_1077_MOESM2_ESM.tif]
