# Supplementary material for: High glutamine suppresses osteogenesis through mTORC1-mediated inhibition of the mTORC2/AKT-473/RUNX2 axis
Source: Cell Death Discov. 2022 Jun 7;8:277. doi: 10.1038/s41420-022-01077-3 (PMC9174279; doi:10.1038/s41420-022-01077-3)

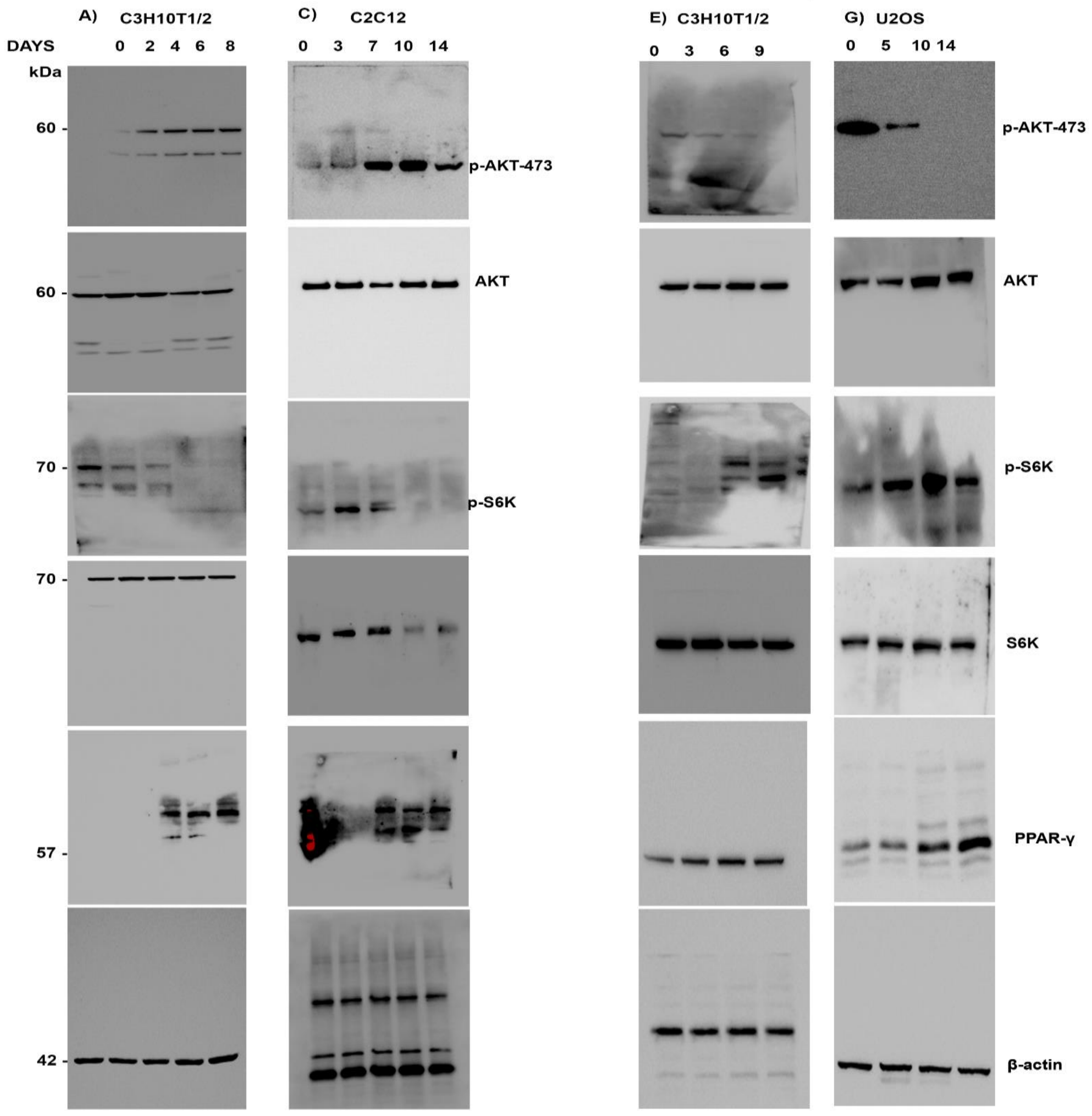
A) C3H10T1/2
DAYS 0 2 4 6 8
kDa
60
60
70
70
57
42
C) C2C12
0 3 7 10 14
p-AKT-473
AKT
p-S6K
E) C3H10T1/2
0 3 6 9
G) U2OS
0 5 10 14
p-AKT-473
AKT
p-S6K
S6K
PPAR-γ
β-actin

Figure 1

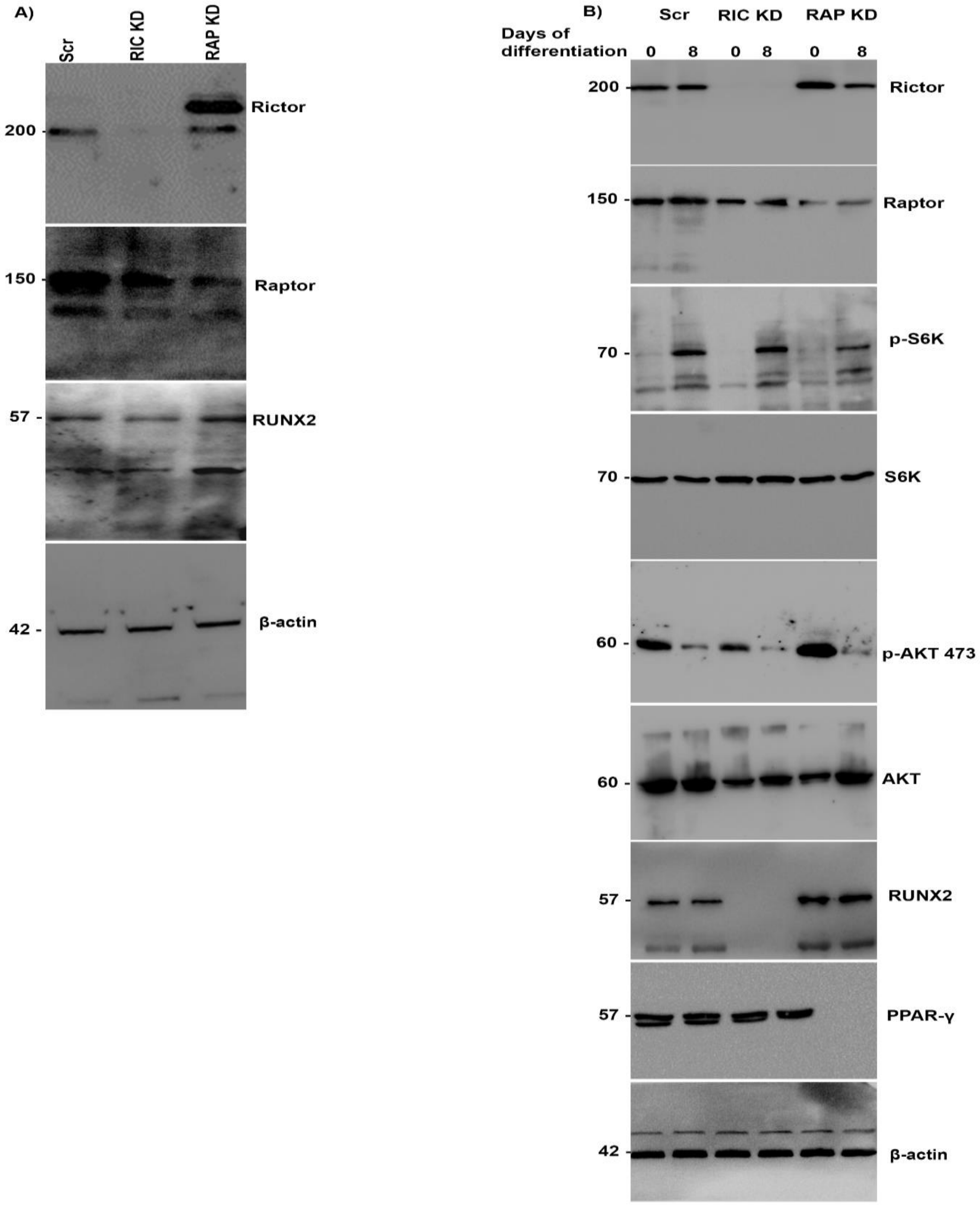
A)
Scr
RIC KD
RAP KD
200
Rictor
150
Raptor
57
RUNX2
42
β-actin
B)
Scr
RIC KD
RAP KD
Days of differentiation
0
8
0
8
0
8
200
Rictor
150
Raptor
70
p-S6K
70
S6K
60
p-AKT 473
60
AKT
57
RUNX2
57
PPAR-γ
42
β-actin

Figure 2

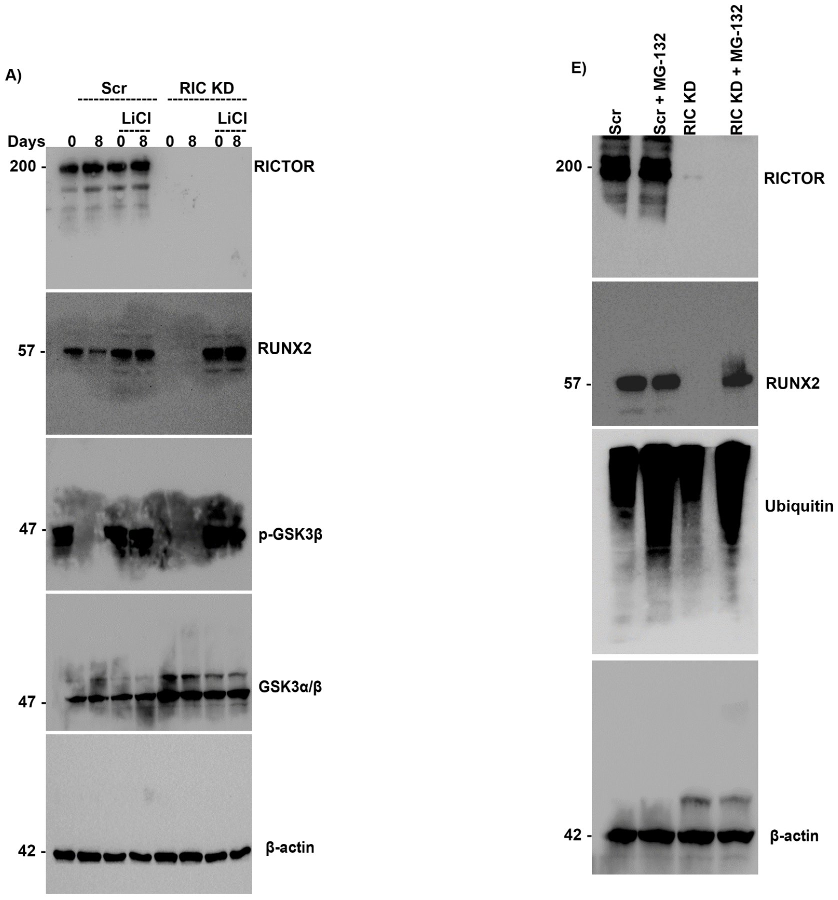
A)
Scr
RIC KD
LiCl
LiCl
Days 0 8 0 8 0 8 0 8
200 -
RICTOR
57 -
RUNX2
47 -
p-GSK3β
47 -
GSK3α/β
42 -
β-actin
E)
Scr
Scr + MG-132
RIC KD
RIC KD + MG-132
200 -
RICTOR
57 -
RUNX2
Ubiquitin
42 -
β-actin

Figure 3

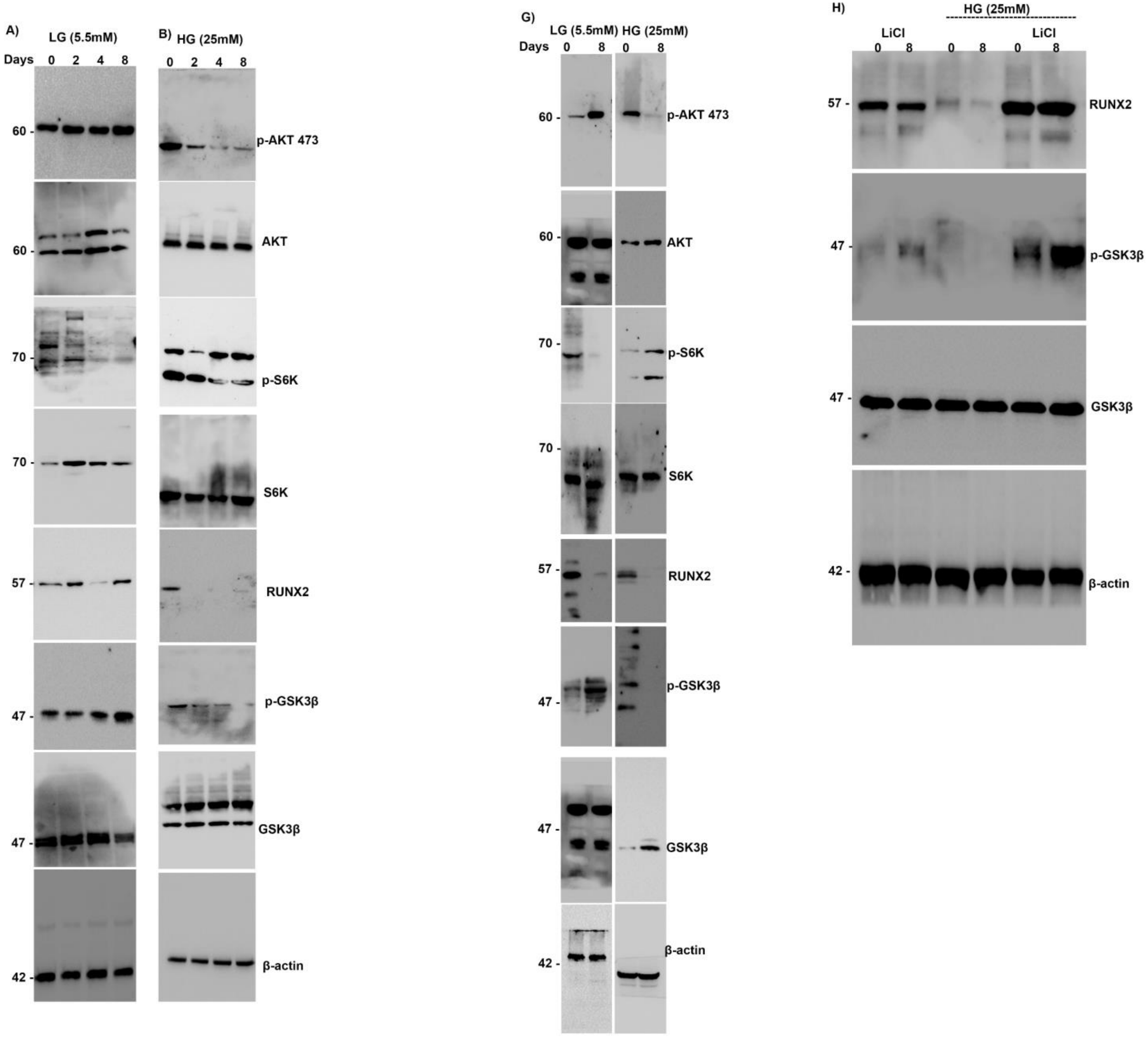
A)
LG (5.5mM)
Days 0 2 4 8
B) HG (25mM)
0 2 4 8
60
p-AKT 473
60
AKT
70
p-S6K
70
S6K
57
RUNX2
47
p-GSK3β
47
GSK3β
42
β-actin
G)
LG (5.5mM) HG (25mM)
Days 0 8 0 8
60
p-AKT 473
60
AKT
70
p-S6K
70
S6K
57
RUNX2
47
p-GSK3β
47
GSK3β
42
β-actin
H)
HG (25mM)
LiCl
LiCl
0 8 0 8 0 8
57
RUNX2
47
p-GSK3β
47
GSK3β
42
β-actin

Figure 4

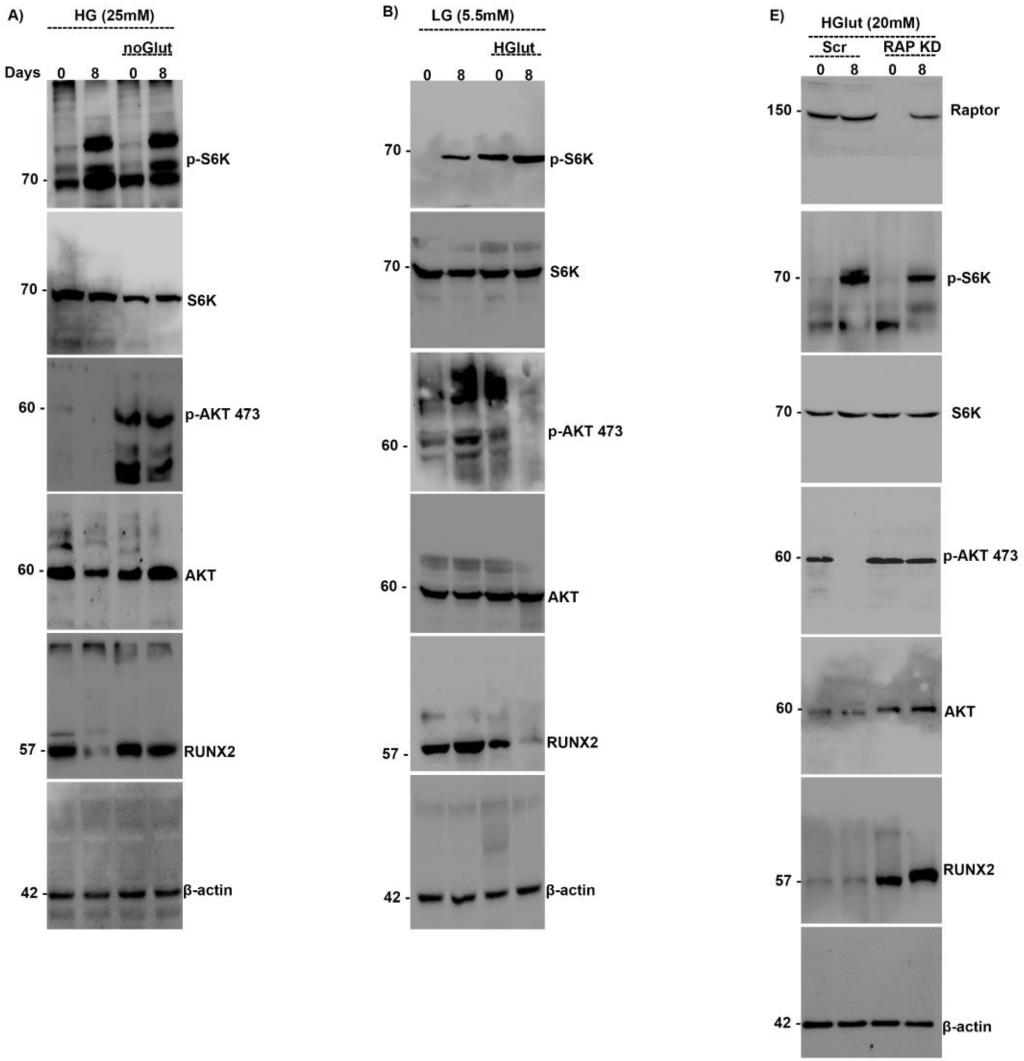
A)
HG (25mM)
noGlut
Days 0 8 0 8
p-S6K
70
S6K
70
p-AKT 473
60
AKT
60
RUNX2
57
β-actin
42
B)
LG (5.5mM)
HGlut
0 8 0 8
70
p-S6K
70
S6K
p-AKT 473
60
60
AKT
RUNX2
57
42
β-actin
E)
HGlut (20mM)
Scr
RAP KD
0 8 0 8
150
Raptor
70
p-S6K
70
S6K
60
p-AKT 473
60
AKT
57
RUNX2
42
β-actin

igure 5

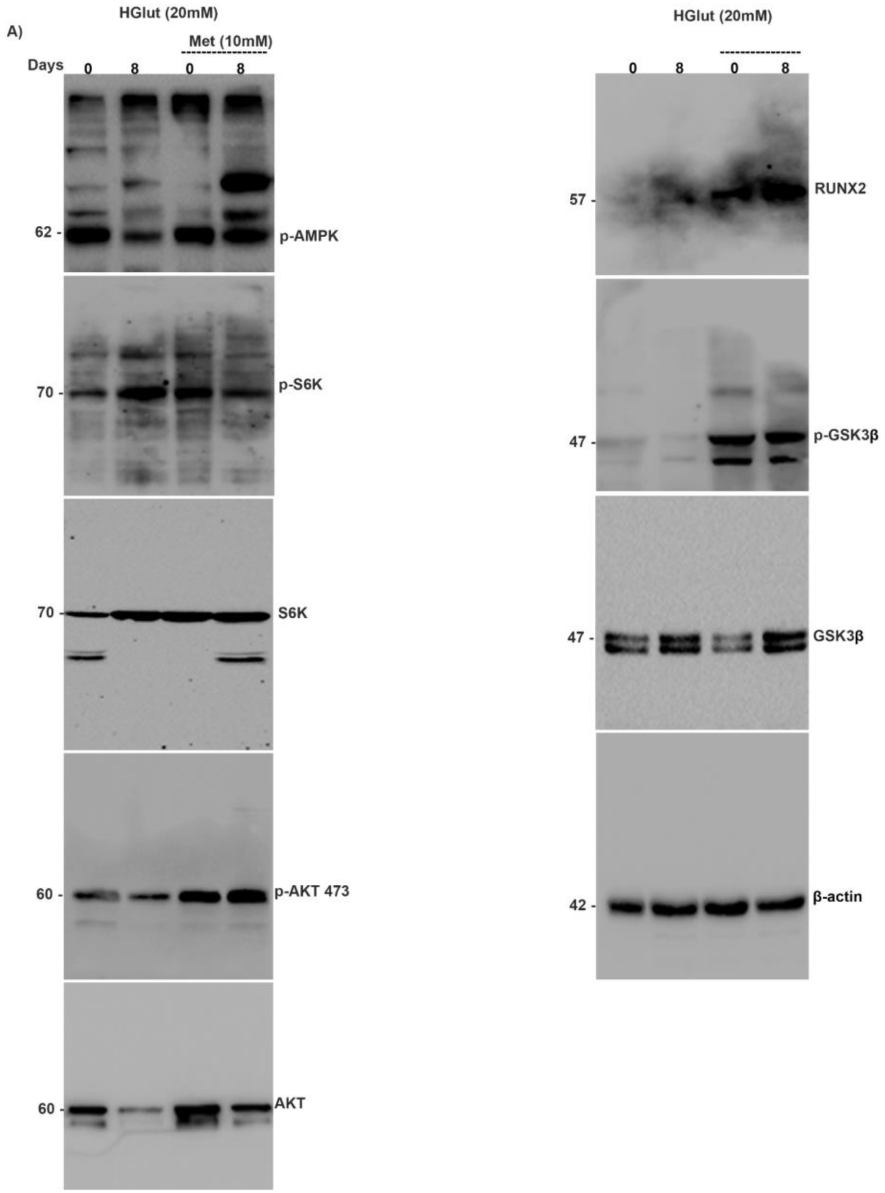
A)
HGlut (20mM)
Met (10mM)
Days
0
8
0
8
62 -
p-AMPK
70 -
p-S6K
70 -
S6K
60 -
p-AKT 473
60 -
AKT
HGlut (20mM)
0
8
0
8
57 -
RUNX2
47 -
p-GSK3β
47 -
GSK3β
42 -
β-actin

Figure 6

Fig.2 C)

D-0 AM + Scr

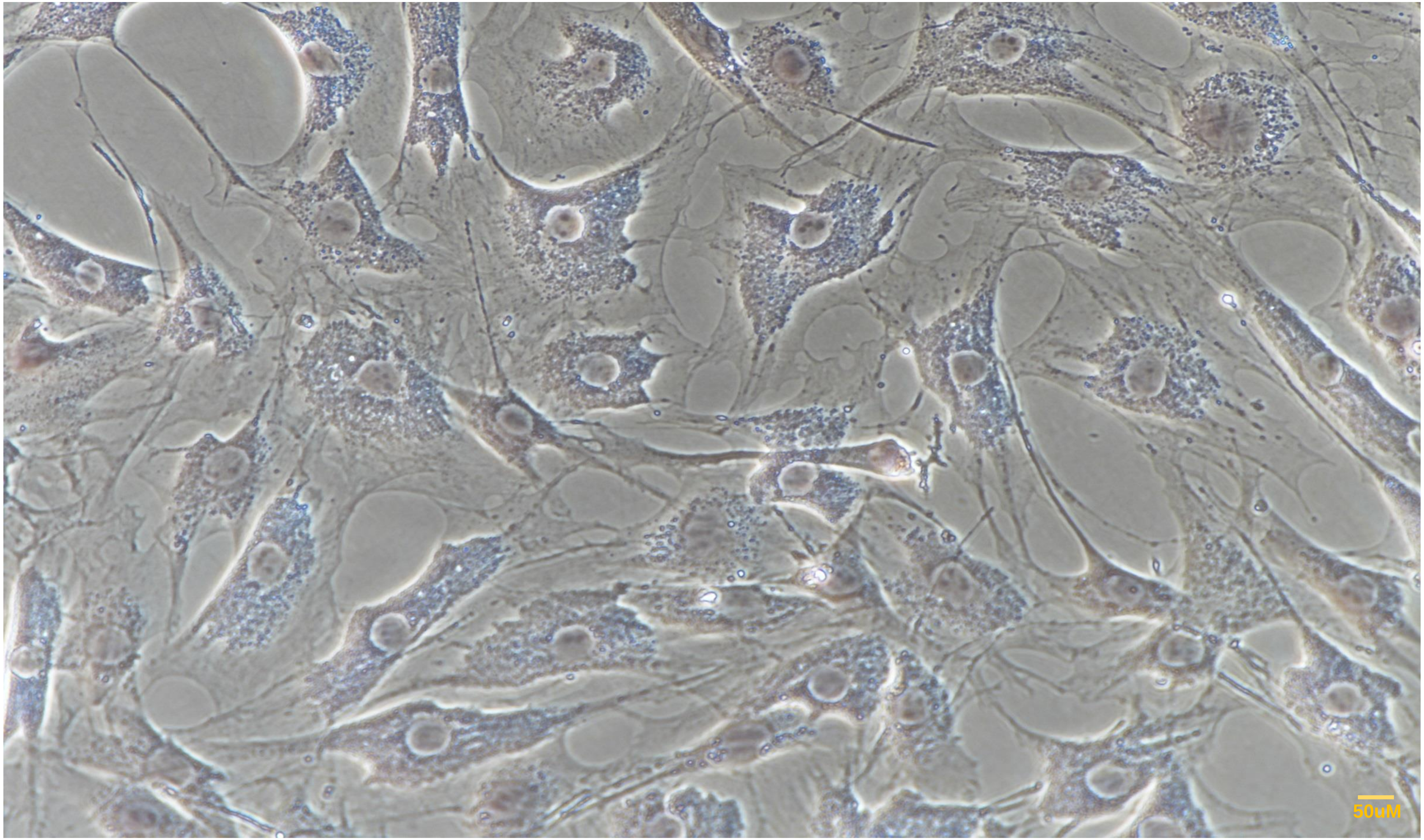

**D-8** **AM + Scr**

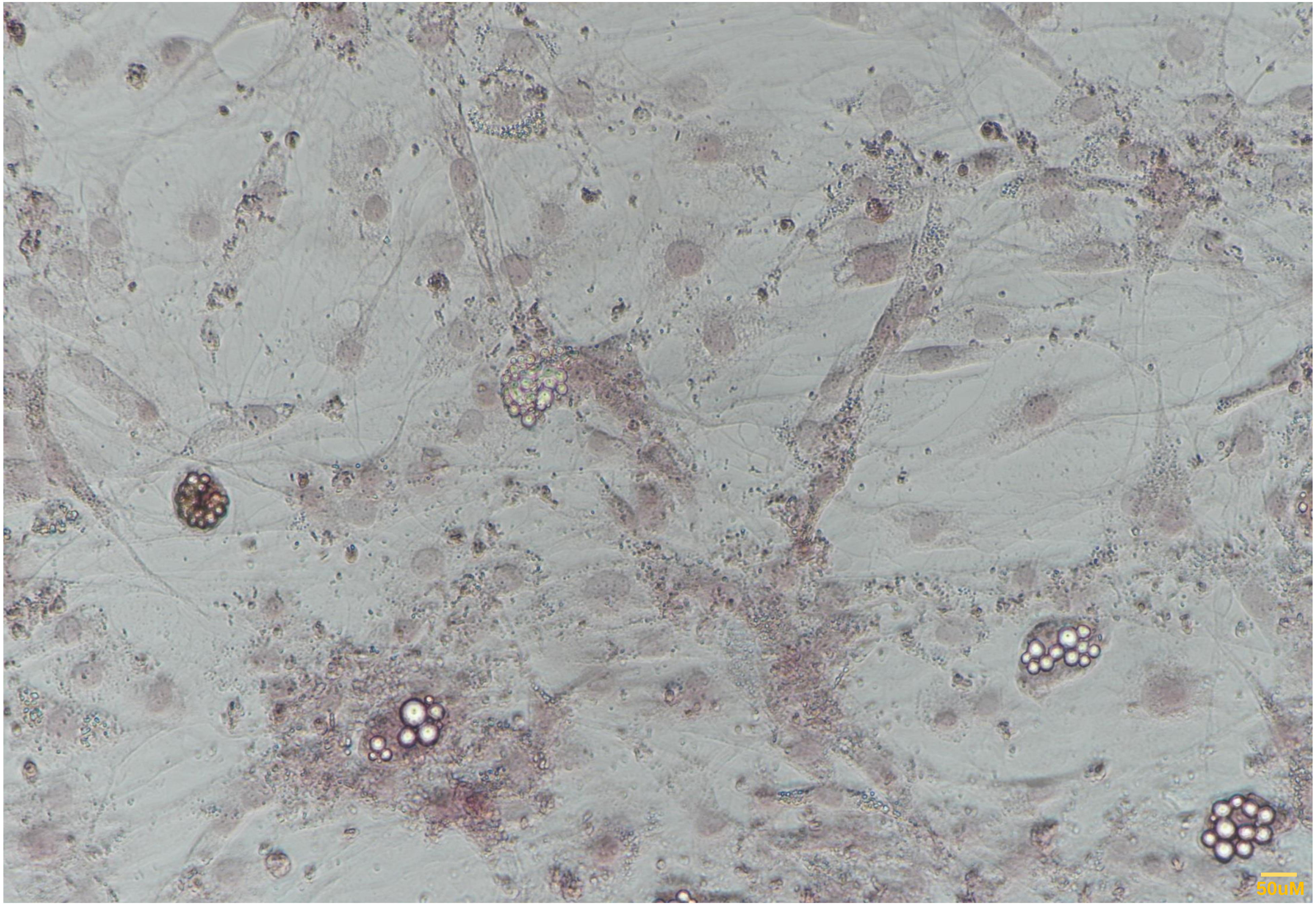

**D-0** **AM + RIC KD**

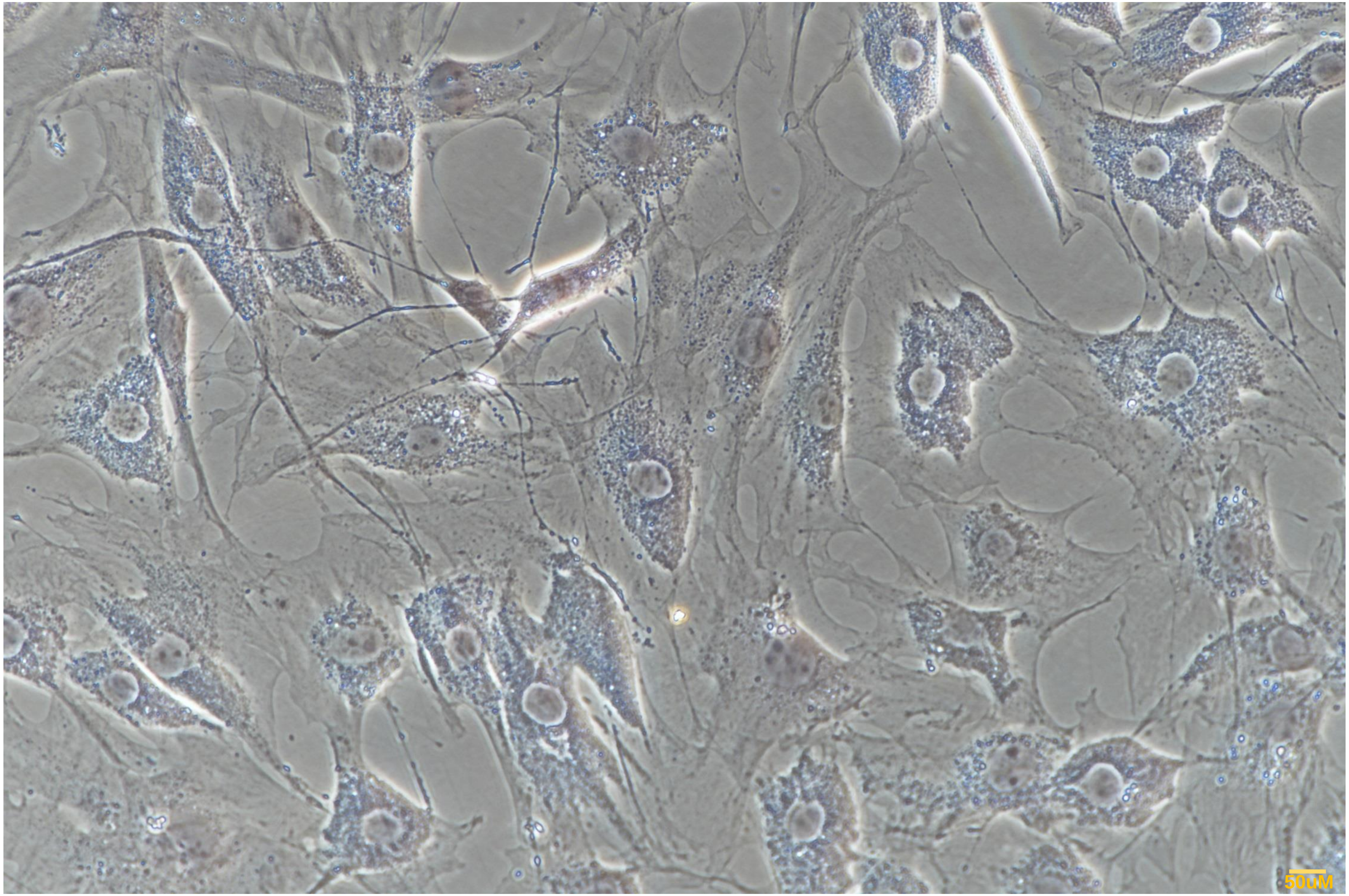

D-8 **AM + RIC KD**

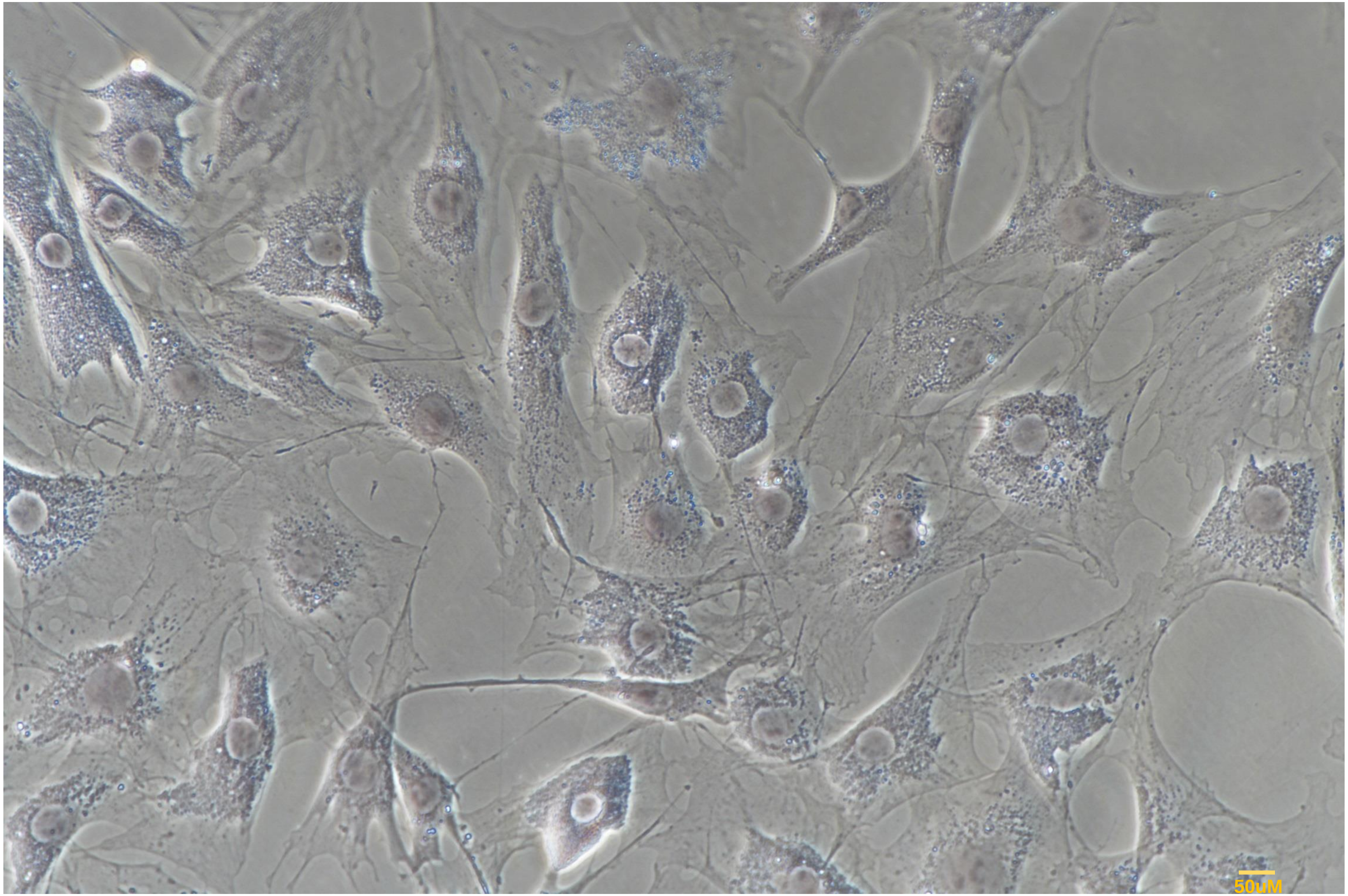

D-0

AM + RAP KD

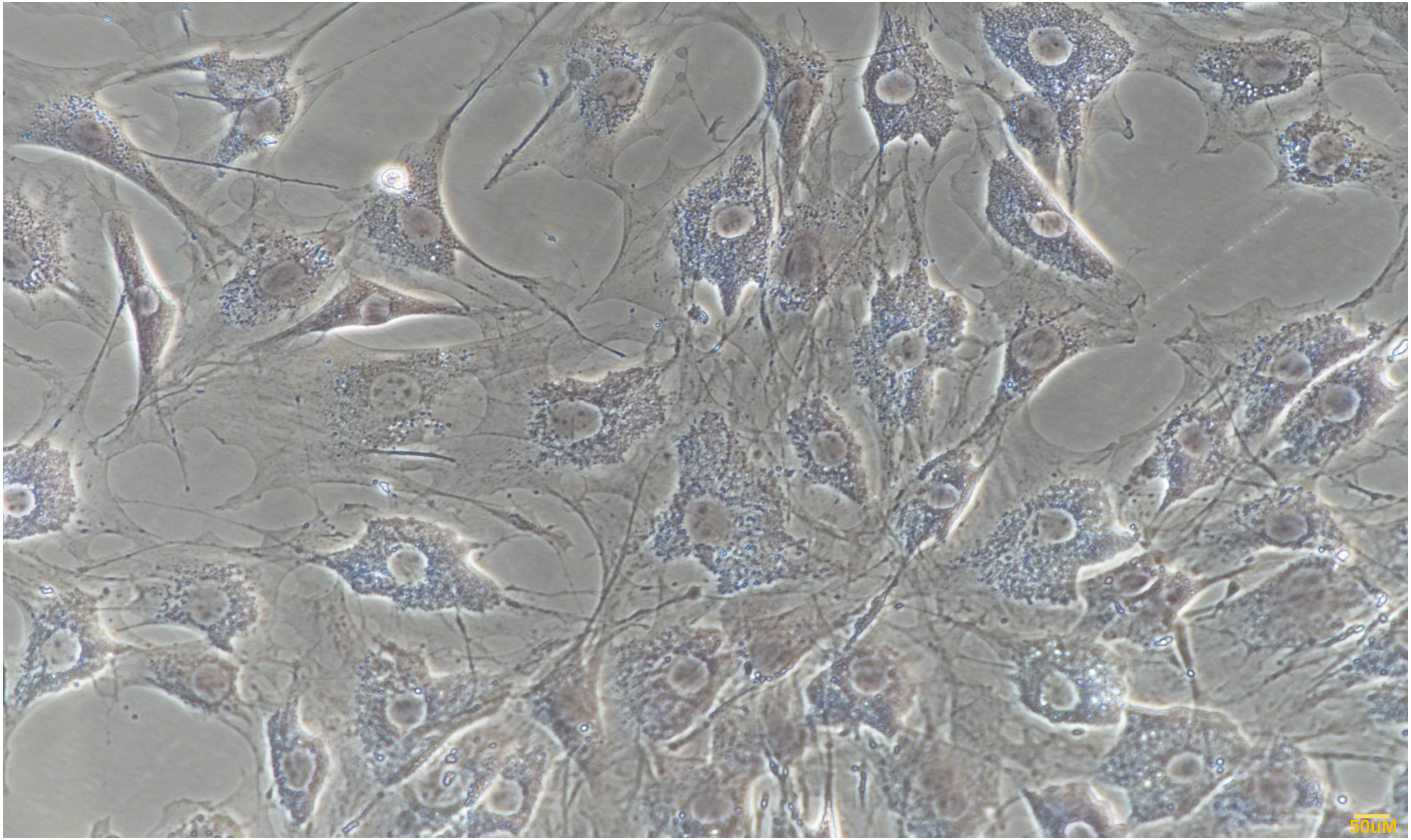

D-8

## AM + RAP KD

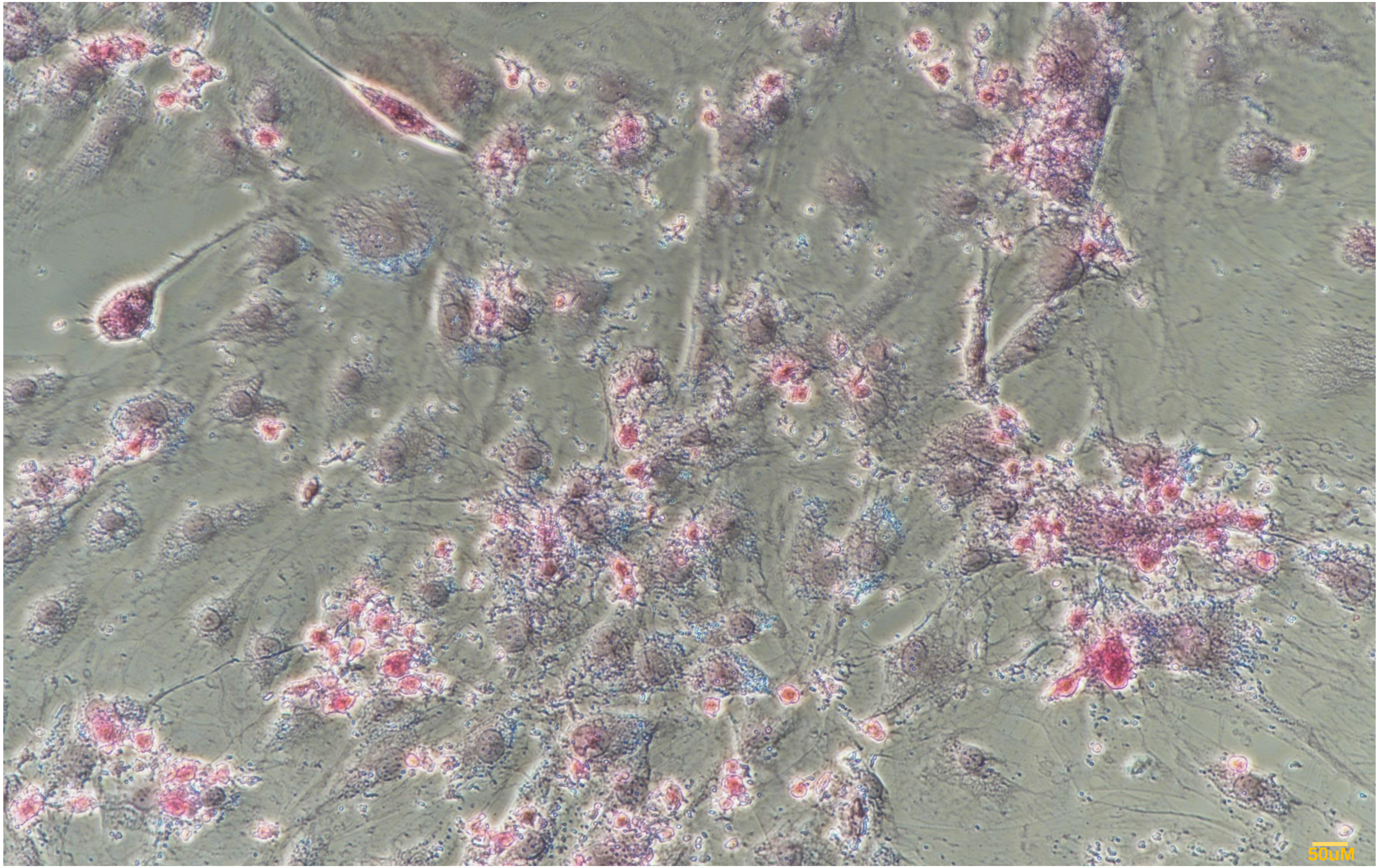

Fig.2 D)

D-0 AM + Scr

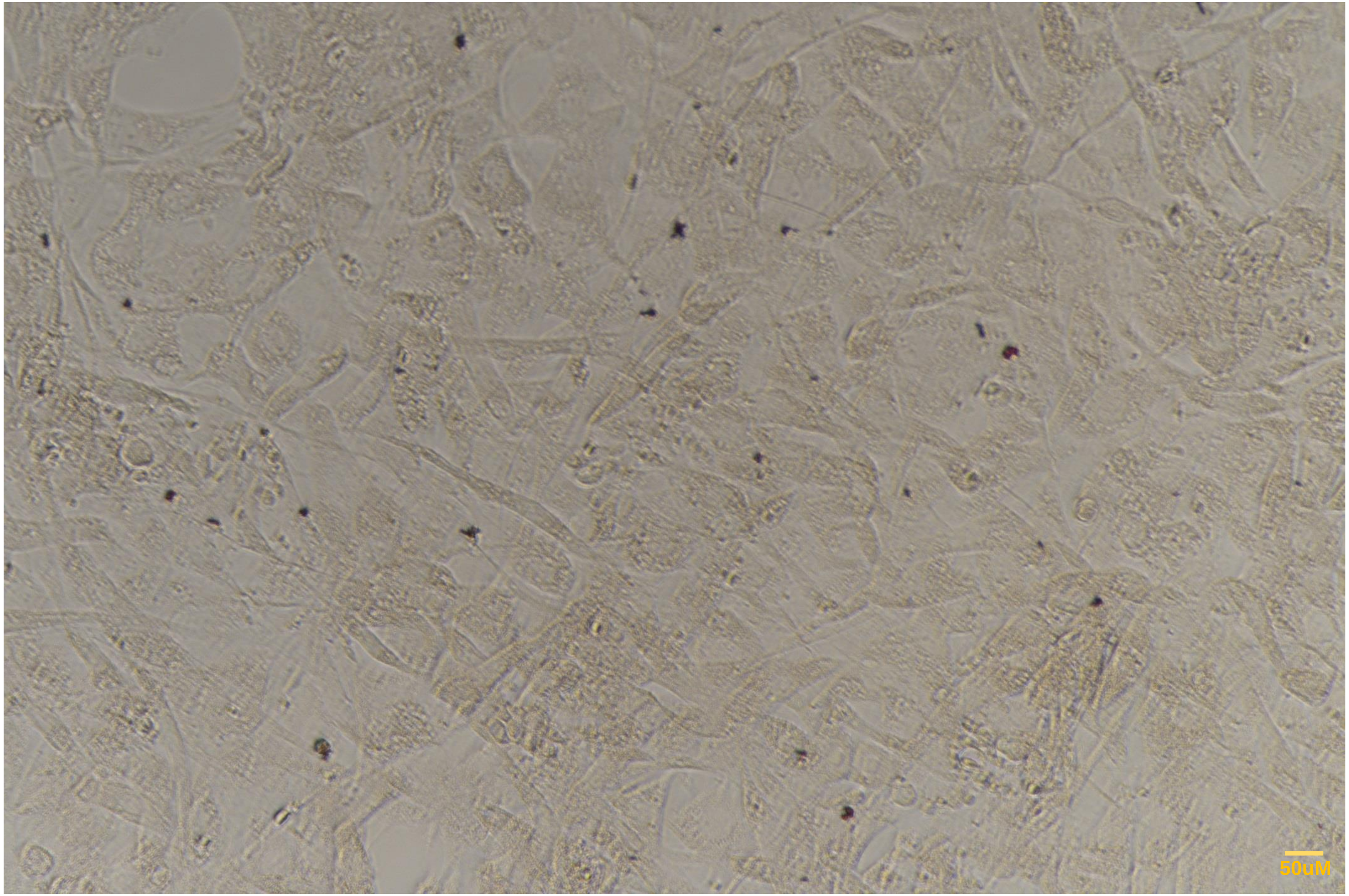

D-8 **AM + Scr**

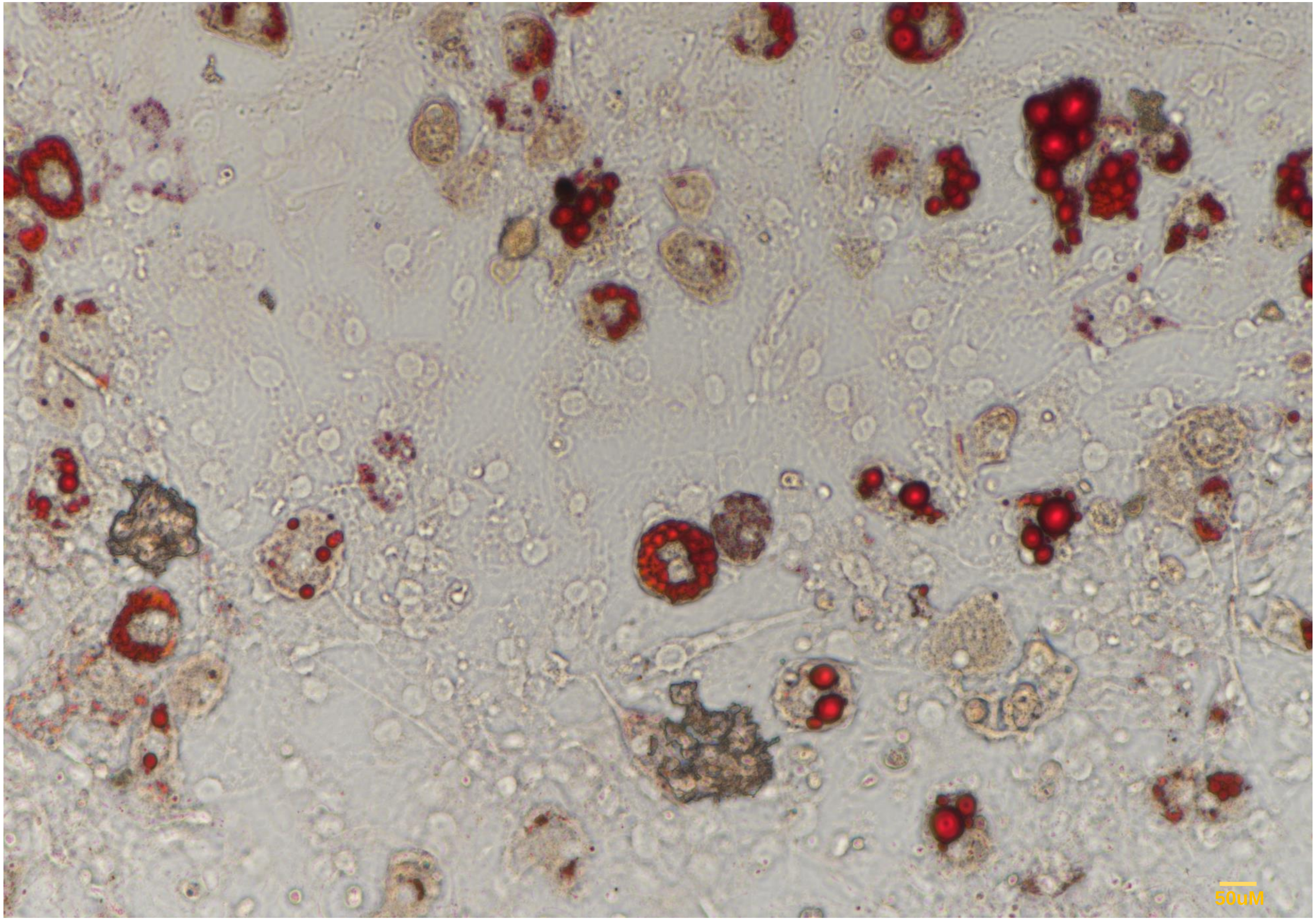

D-0 **AM + RIC KD**

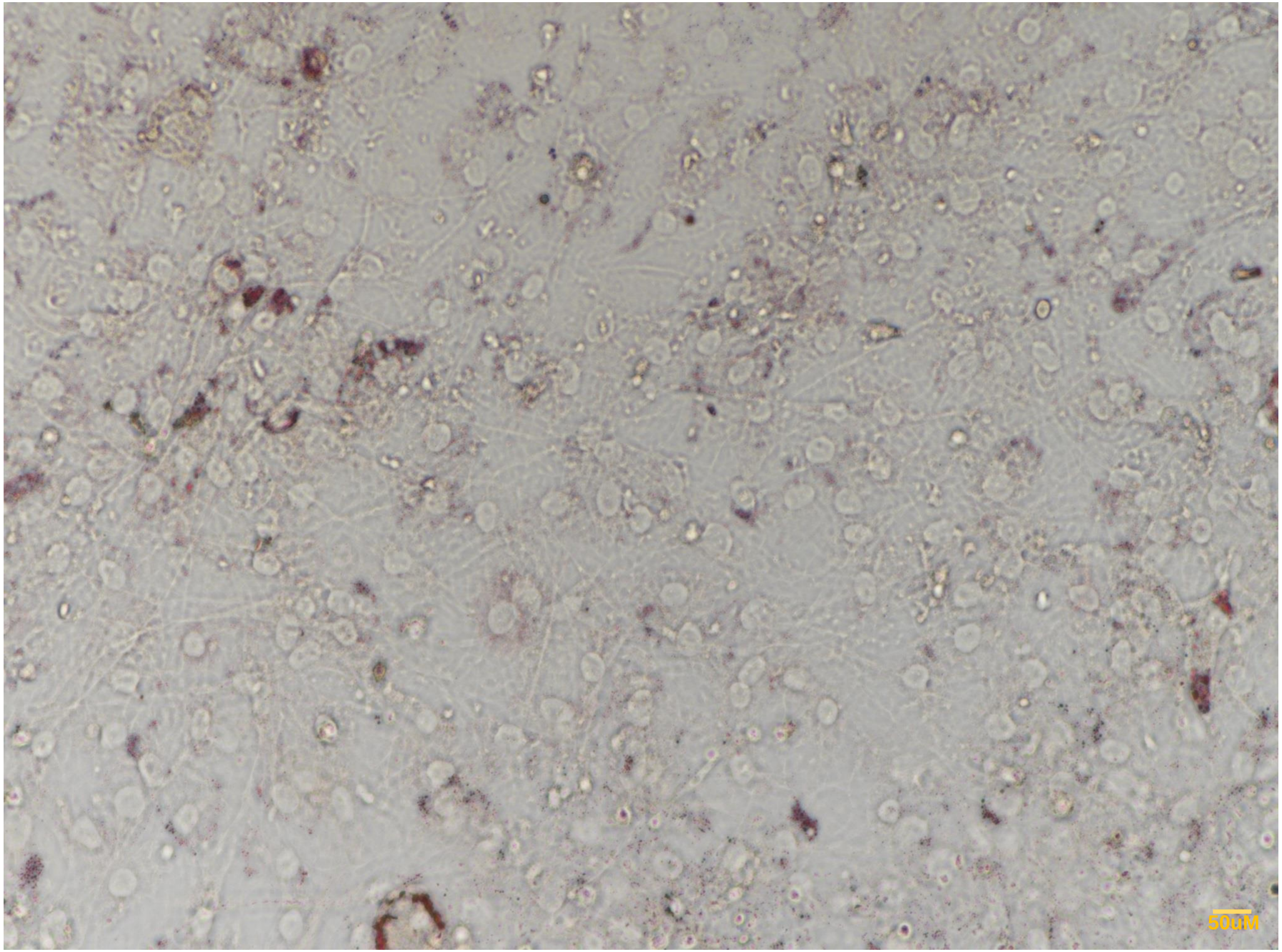

**D-8** **AM + RIC KD**

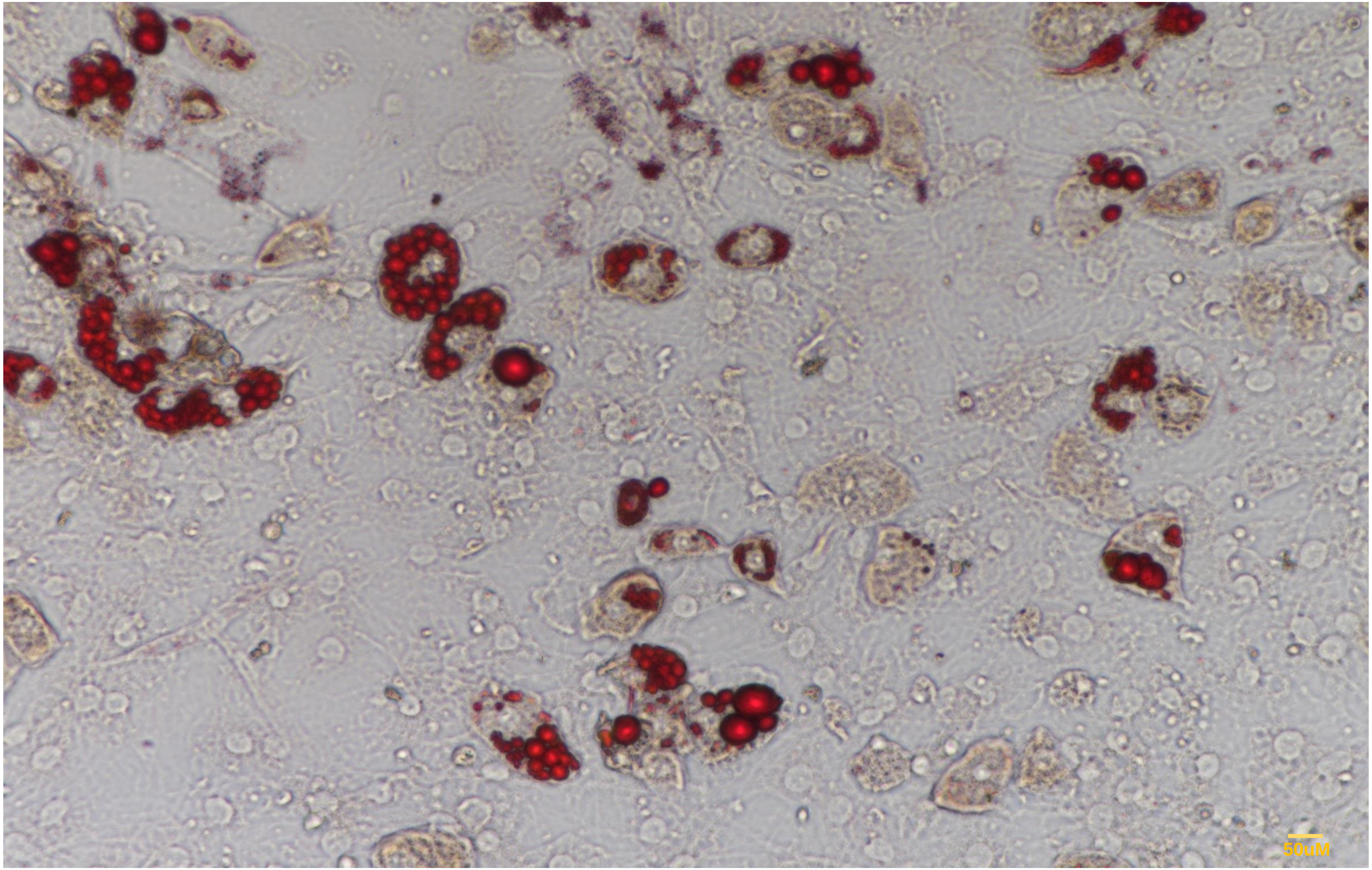

D-0 AM + RAP KD

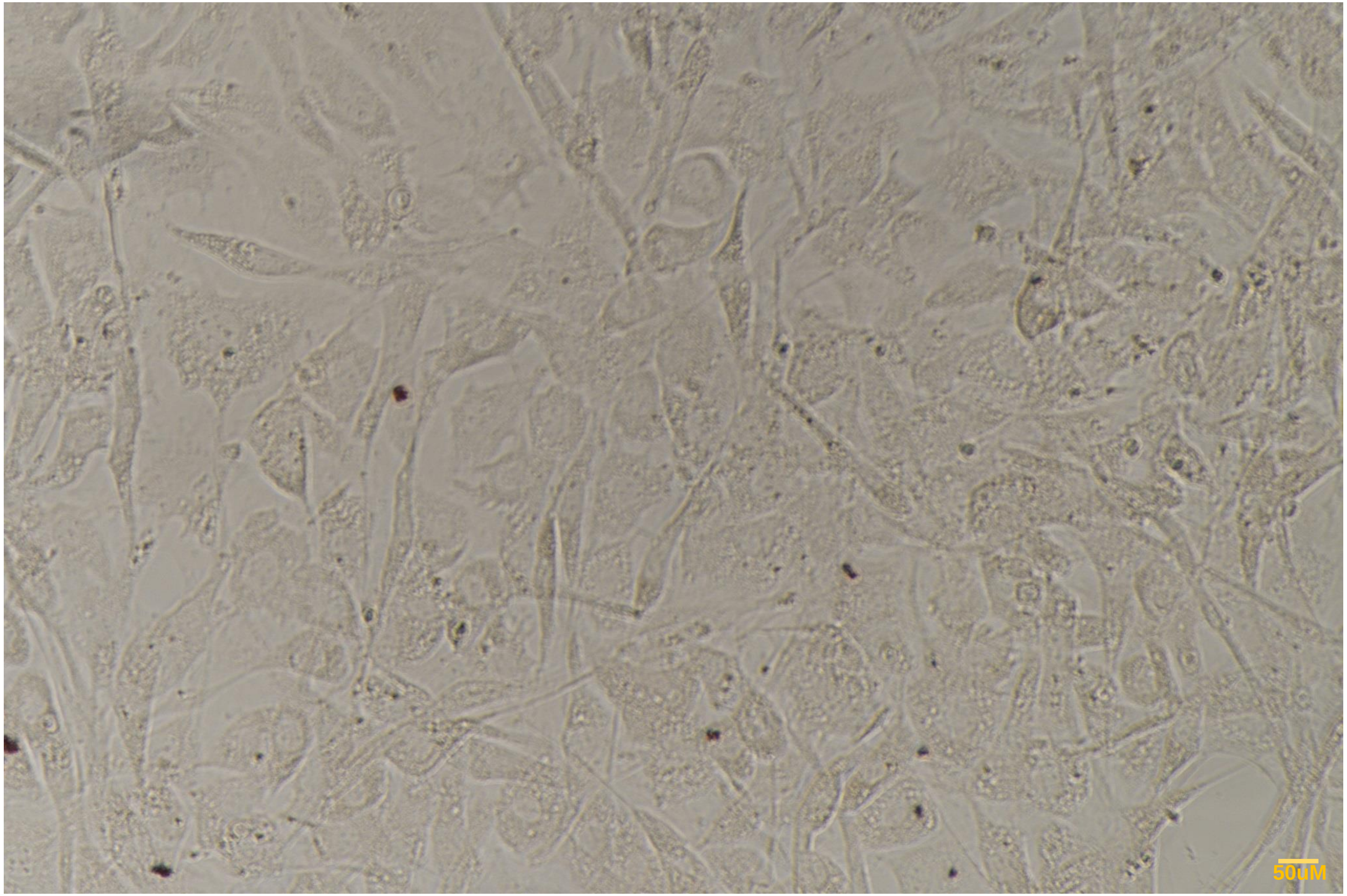

D-8

AM + RAP KD

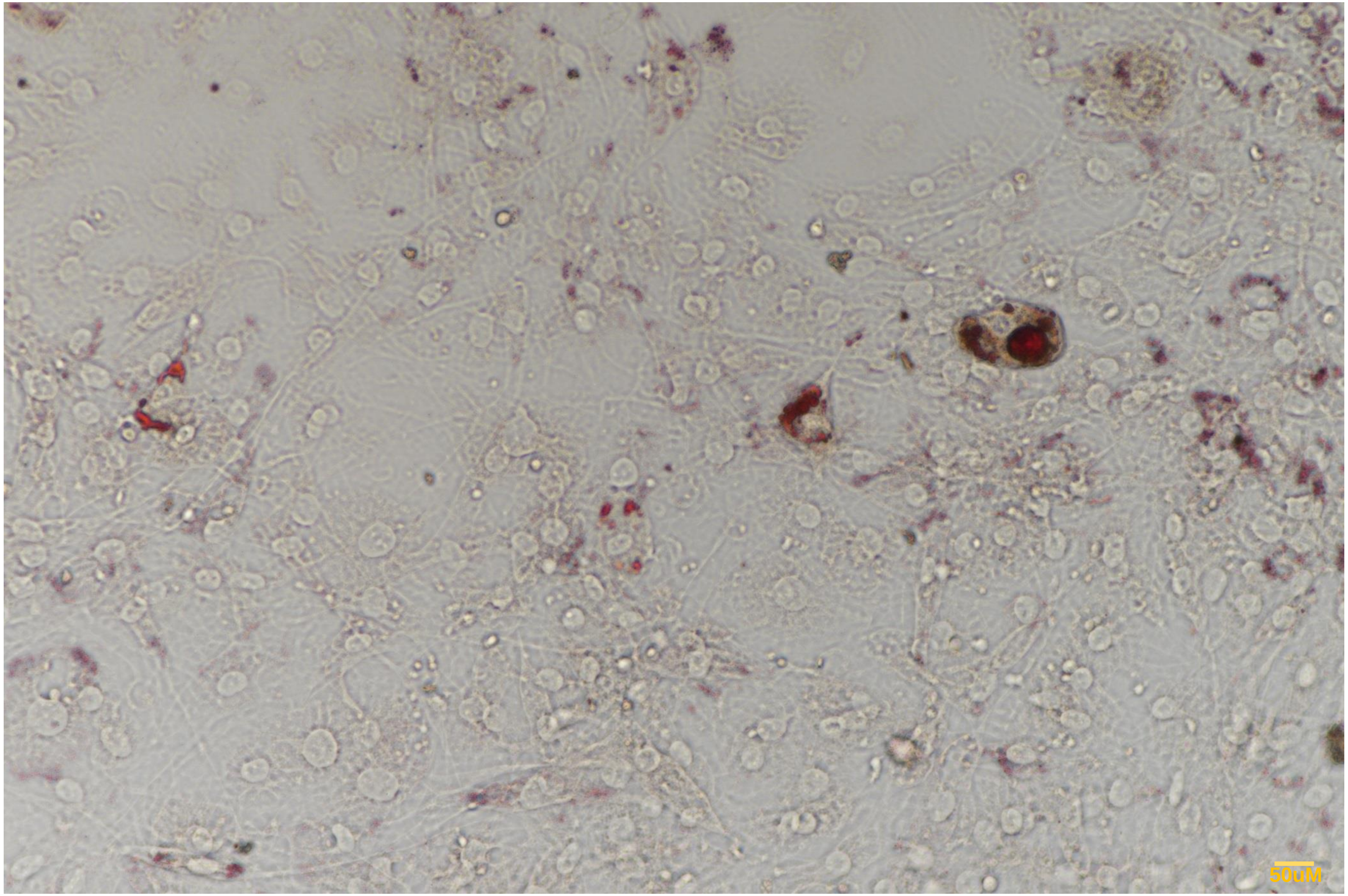

Fig.2 G)

UND RUNX2

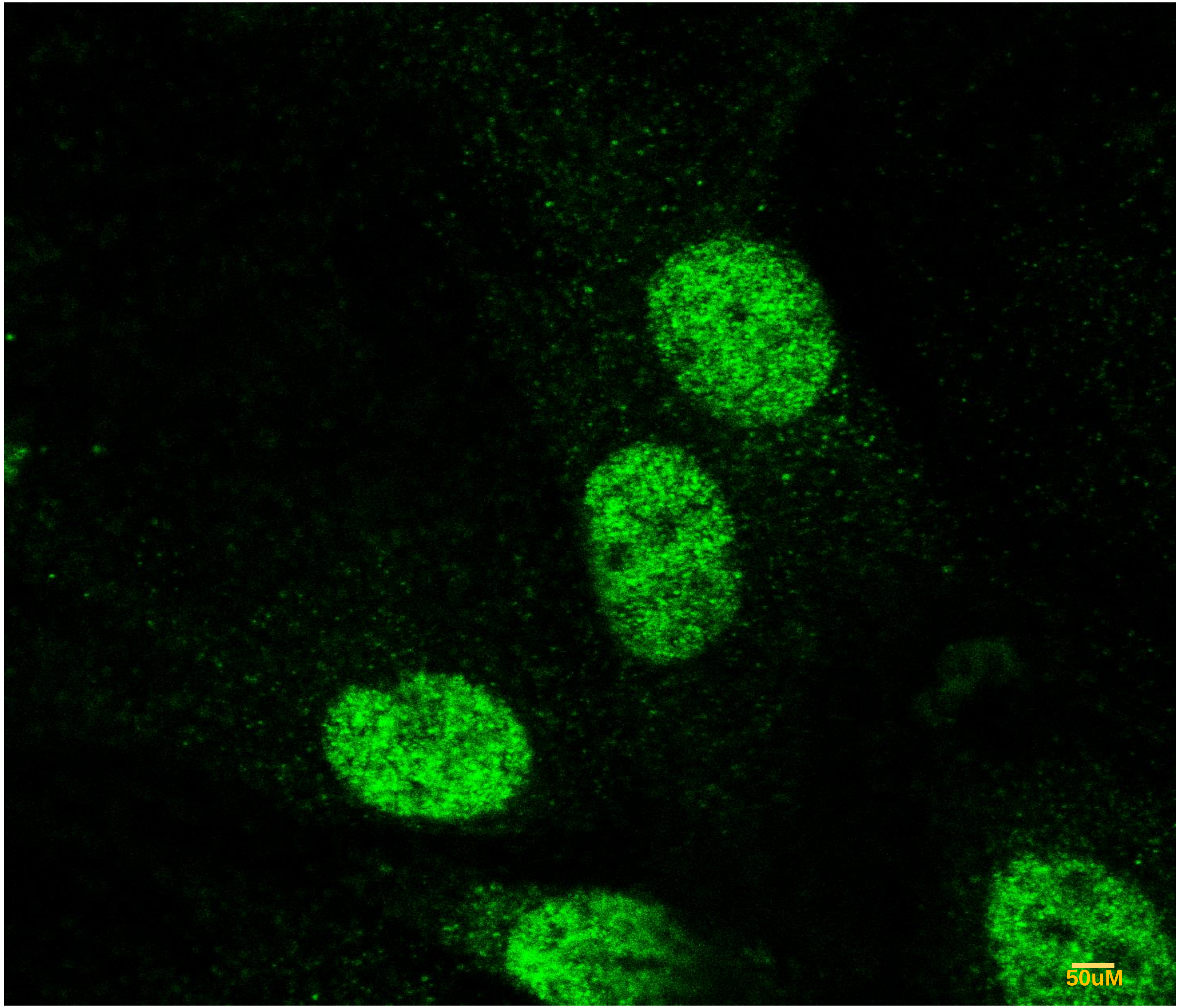

AM + Scr RUNX2

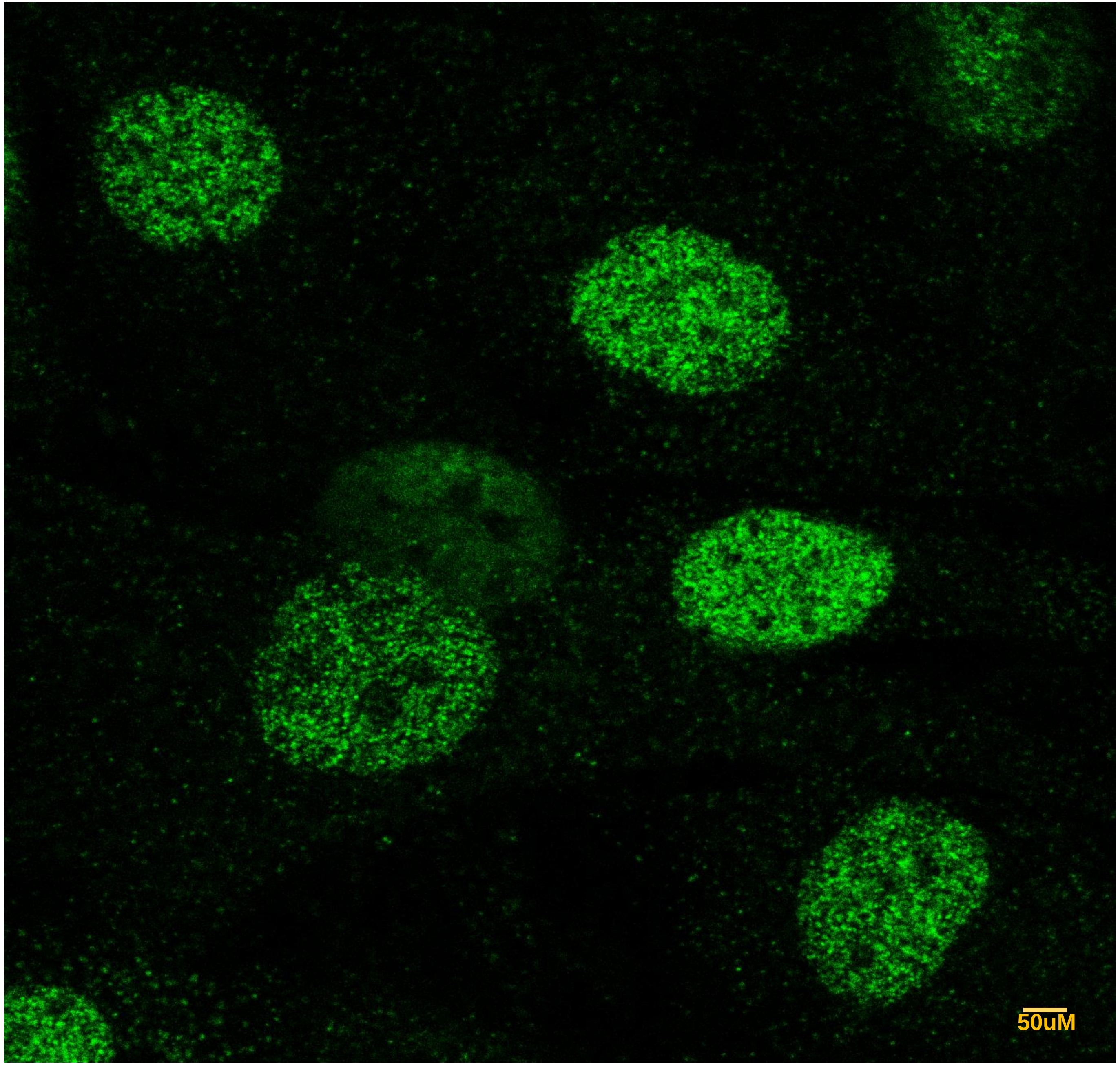

AM + RIC KD RUNX2

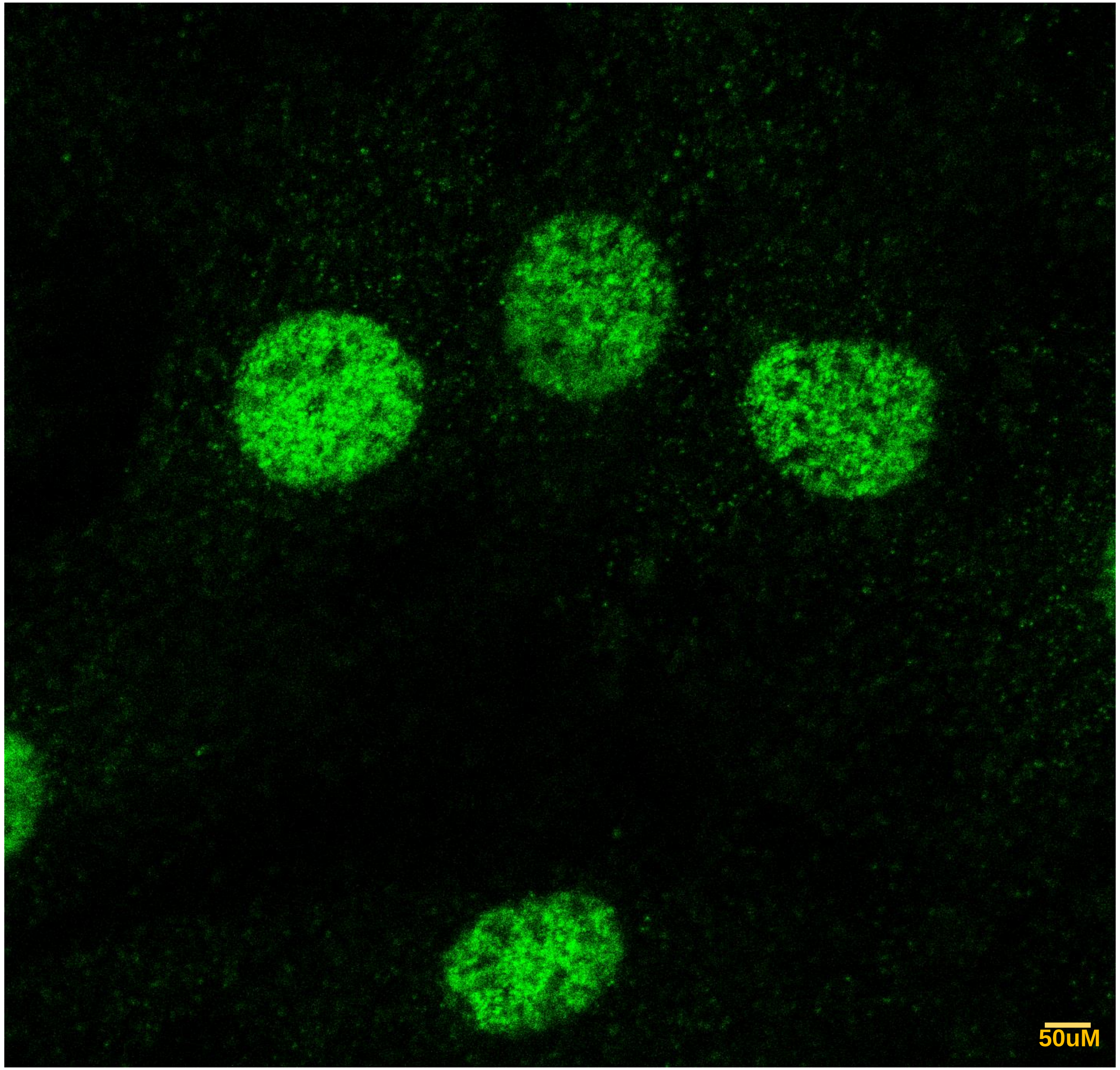

AM + RAP KD RUNX2

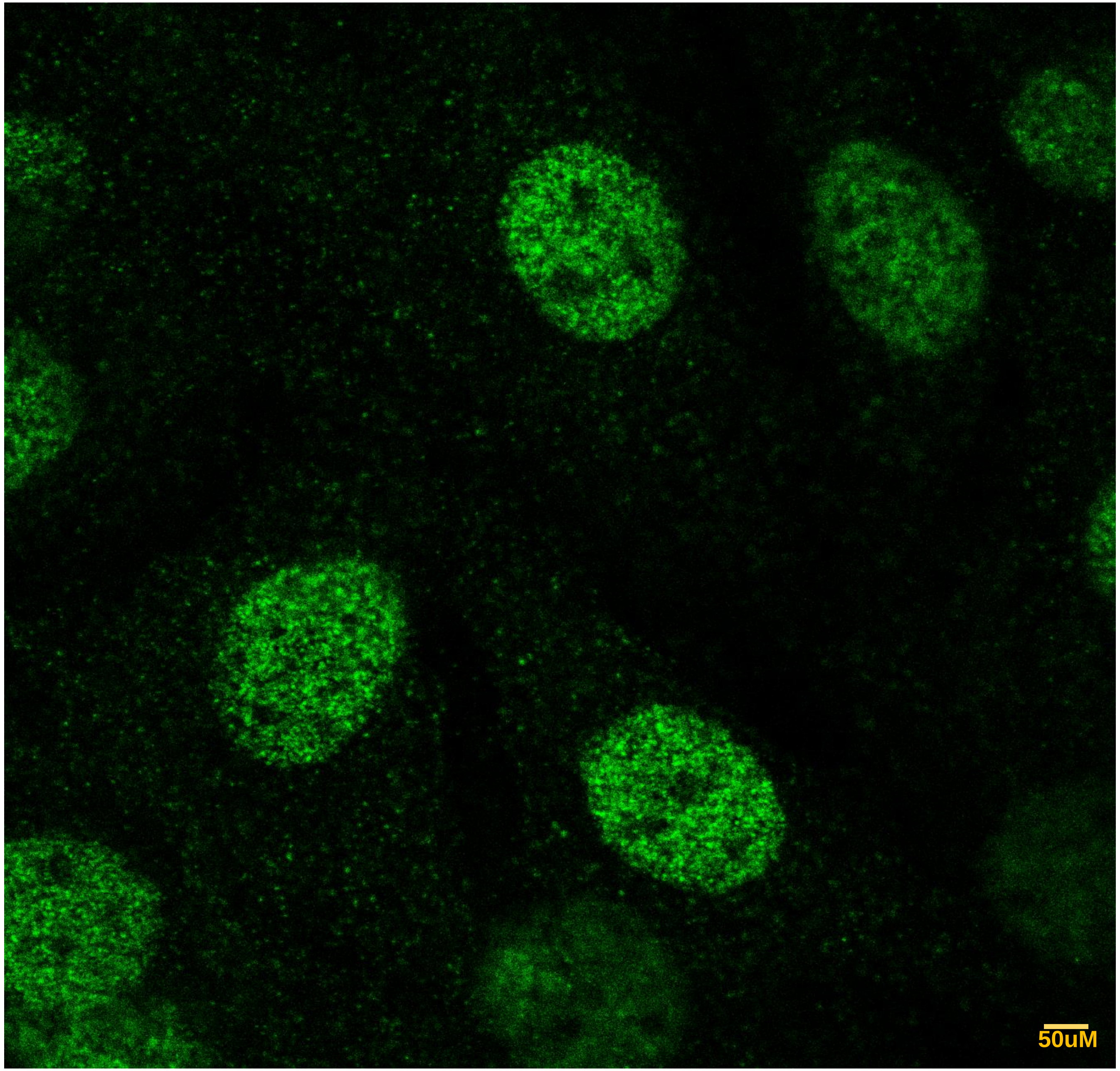

UND GSK3β

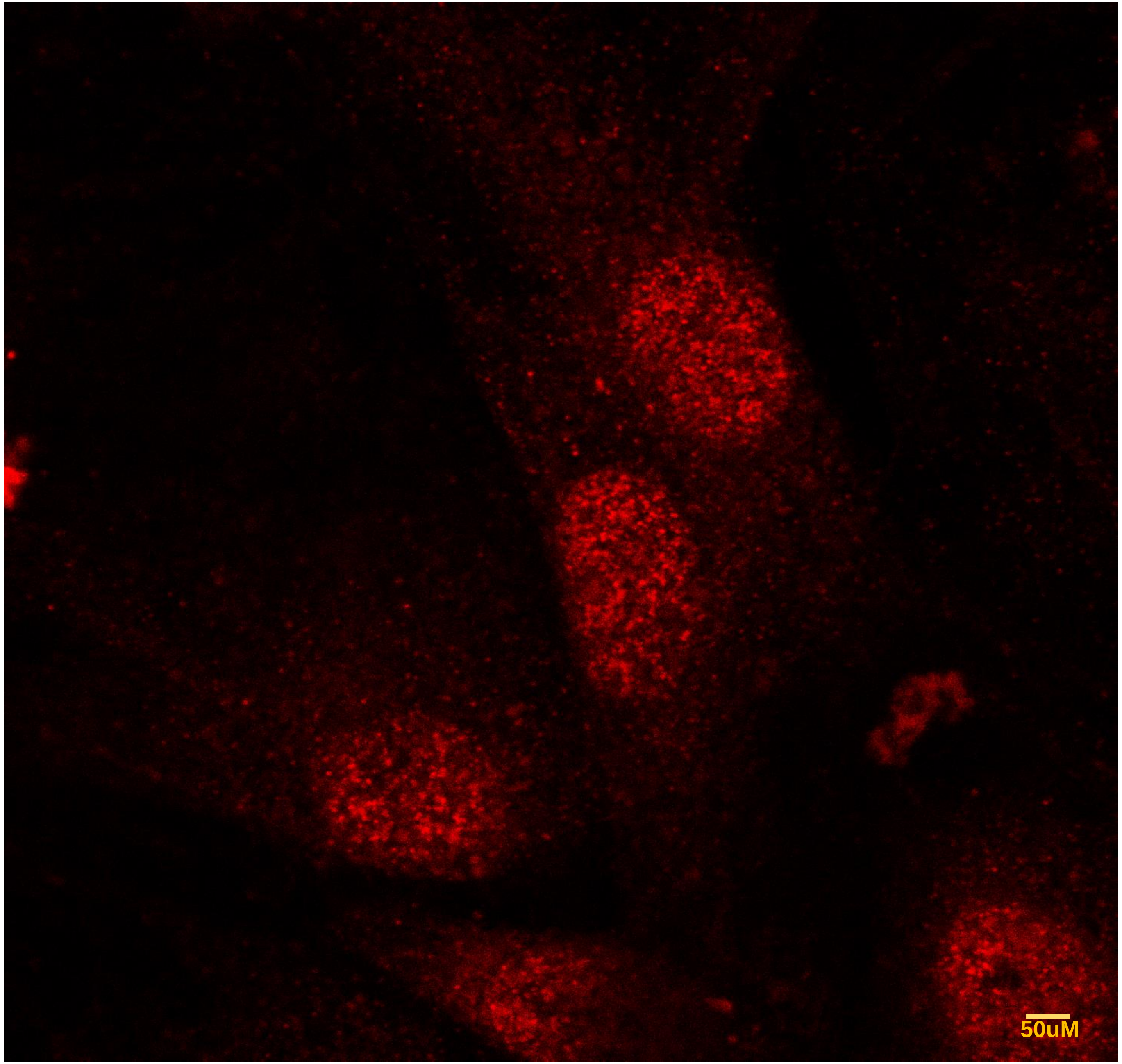

AM + Scr GSK3β

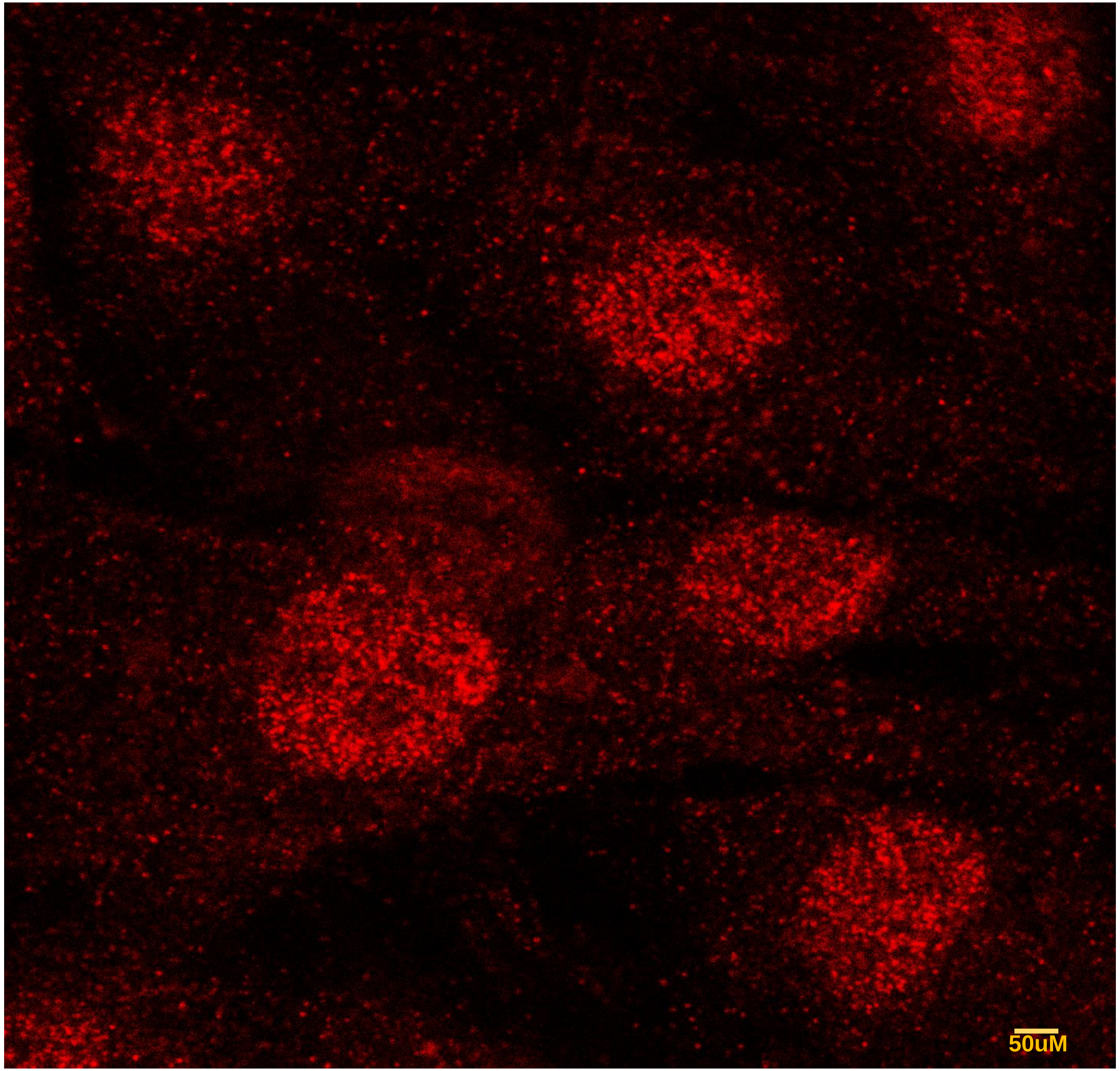

AM + RIC KD GSK3β

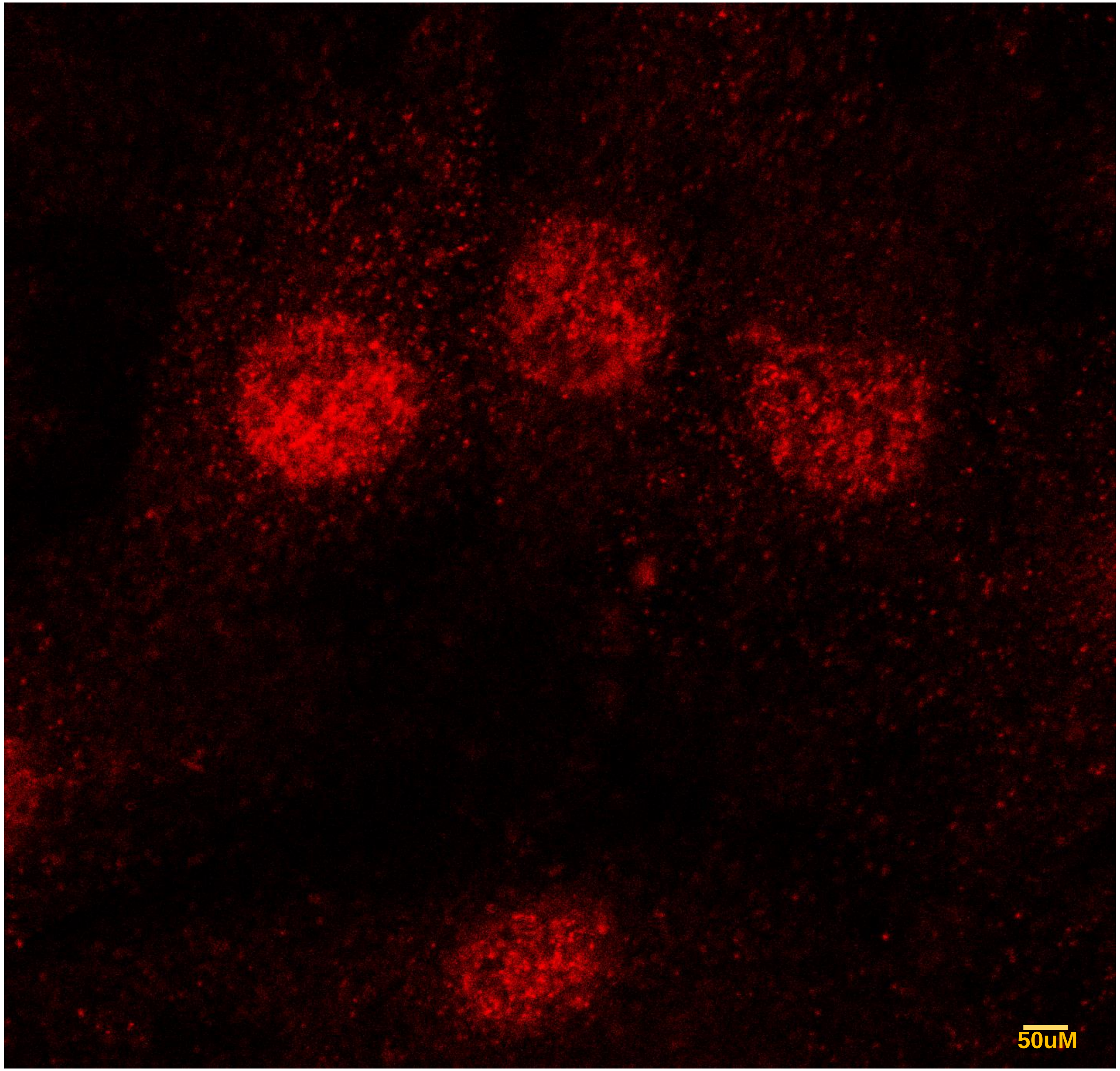

AM + RAP KD GSK3β

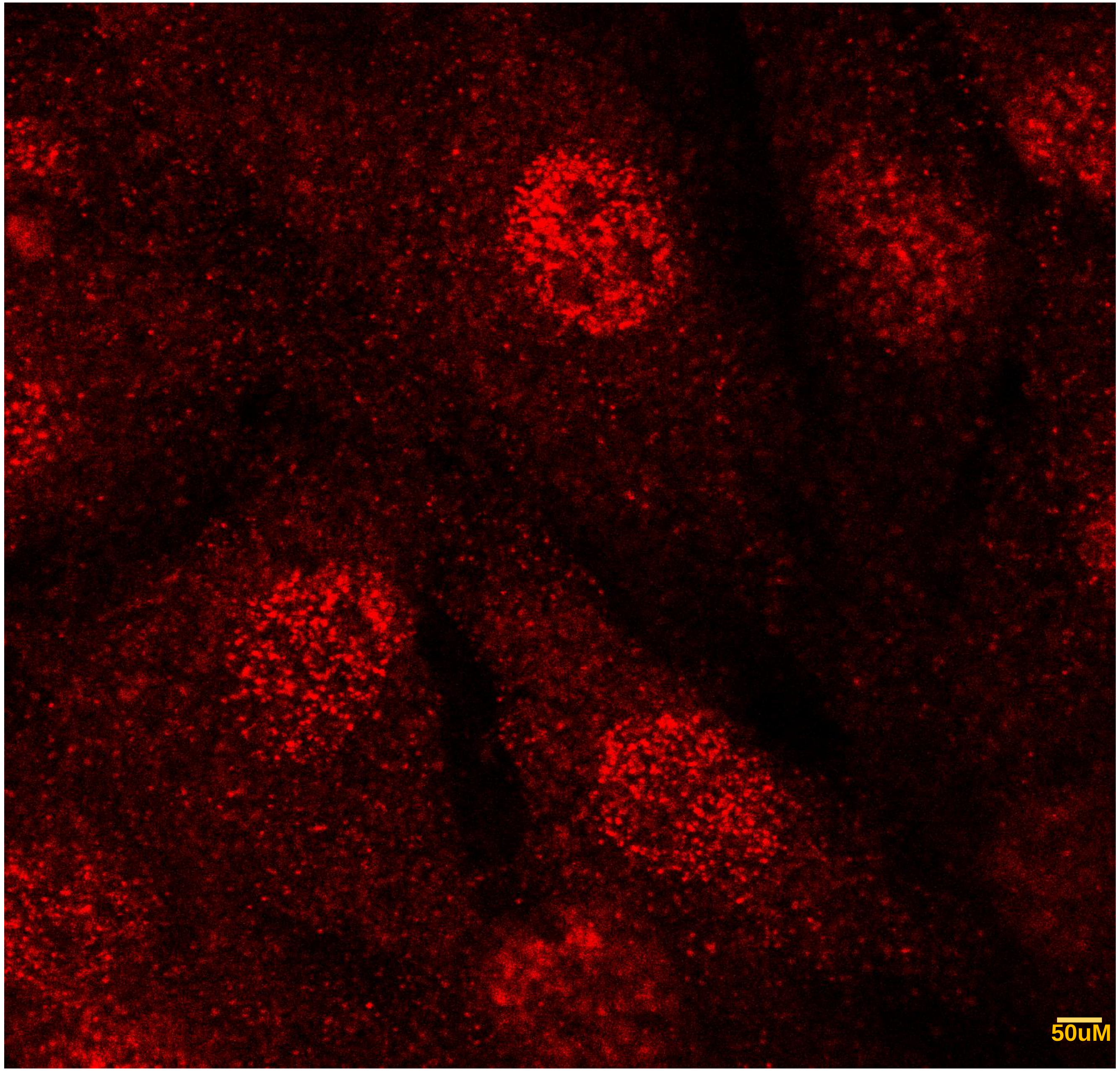

UND MERGED

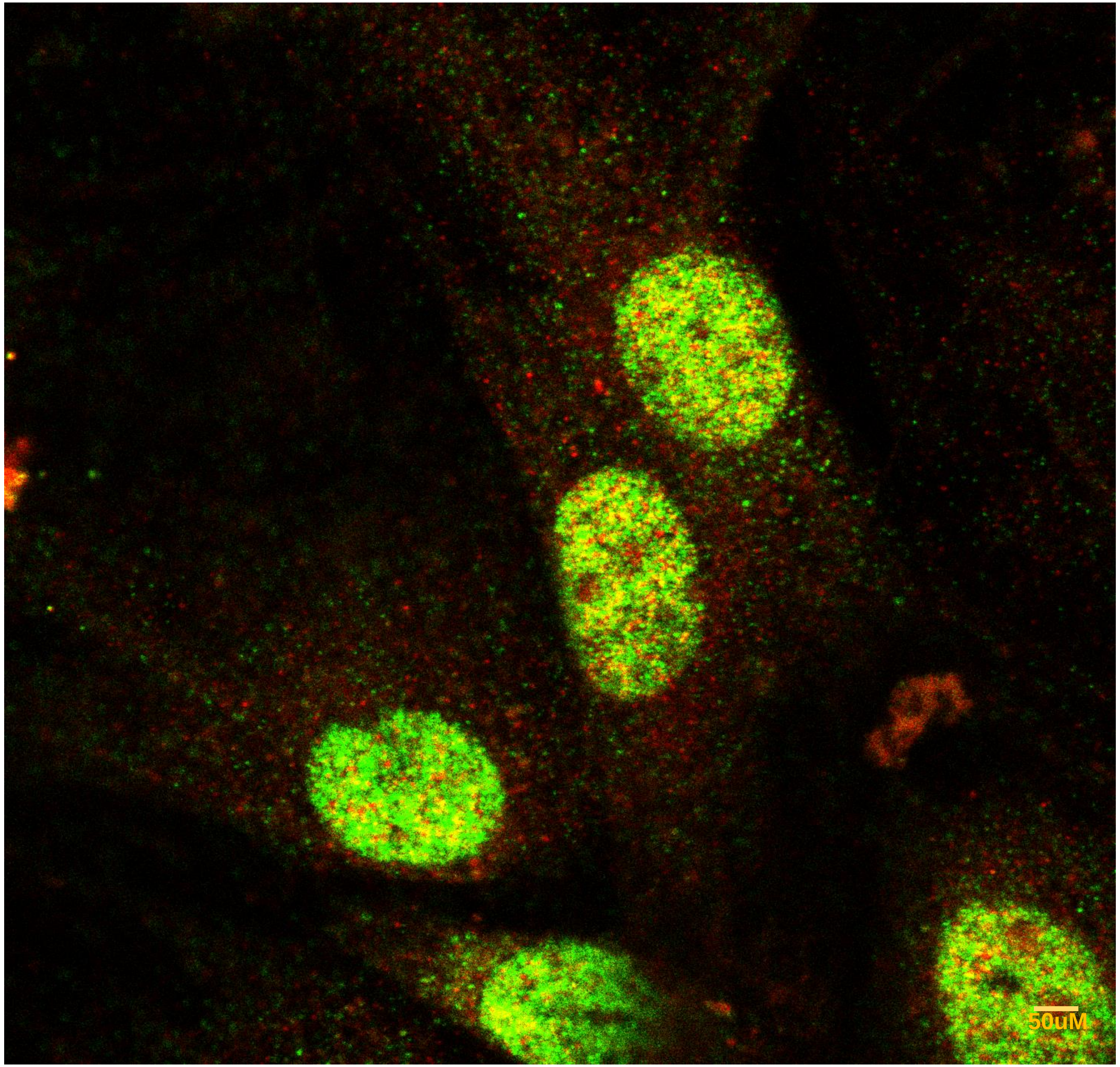

AM + Scr

MERGED

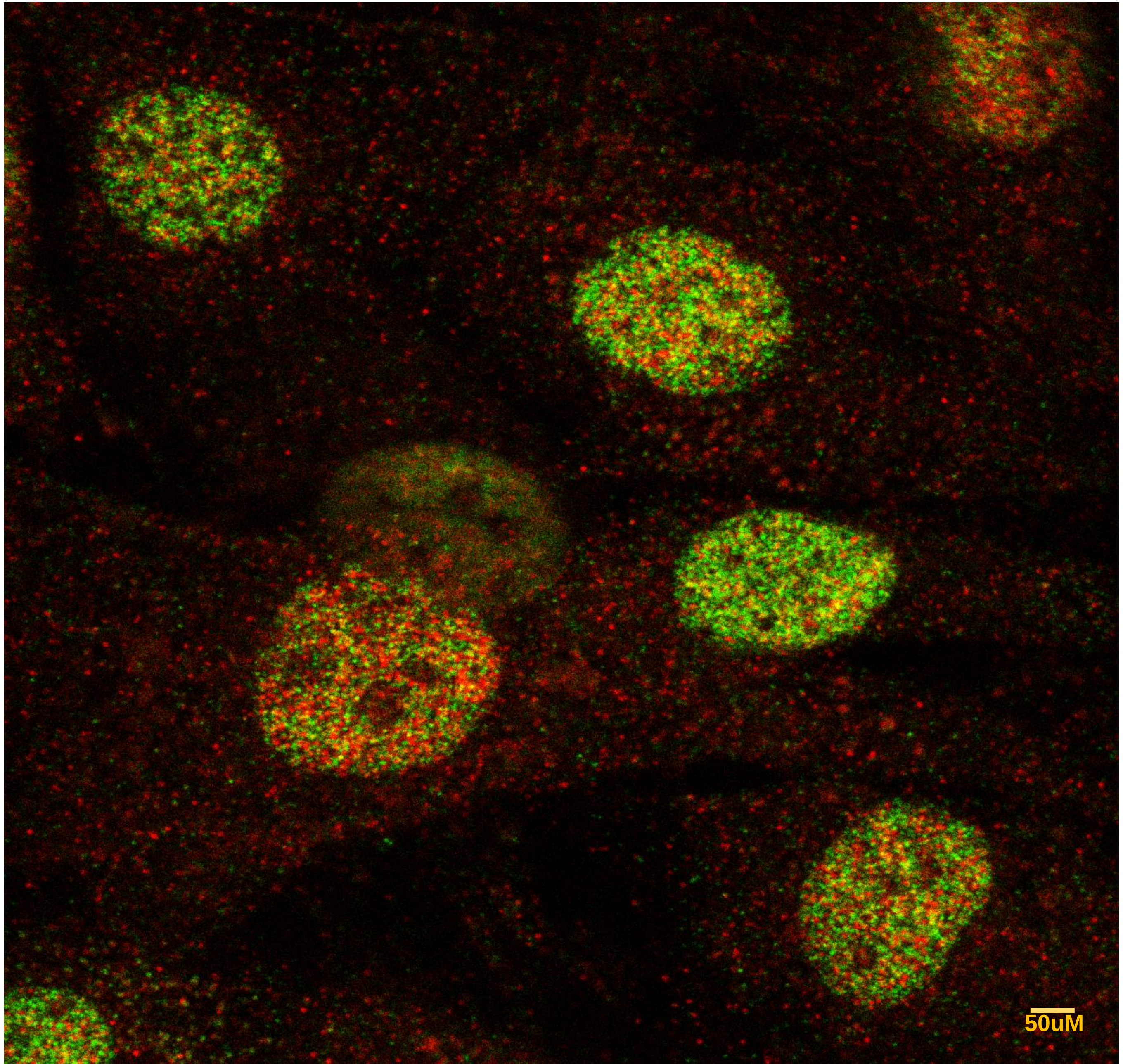

AM + RIC KD

MERGED

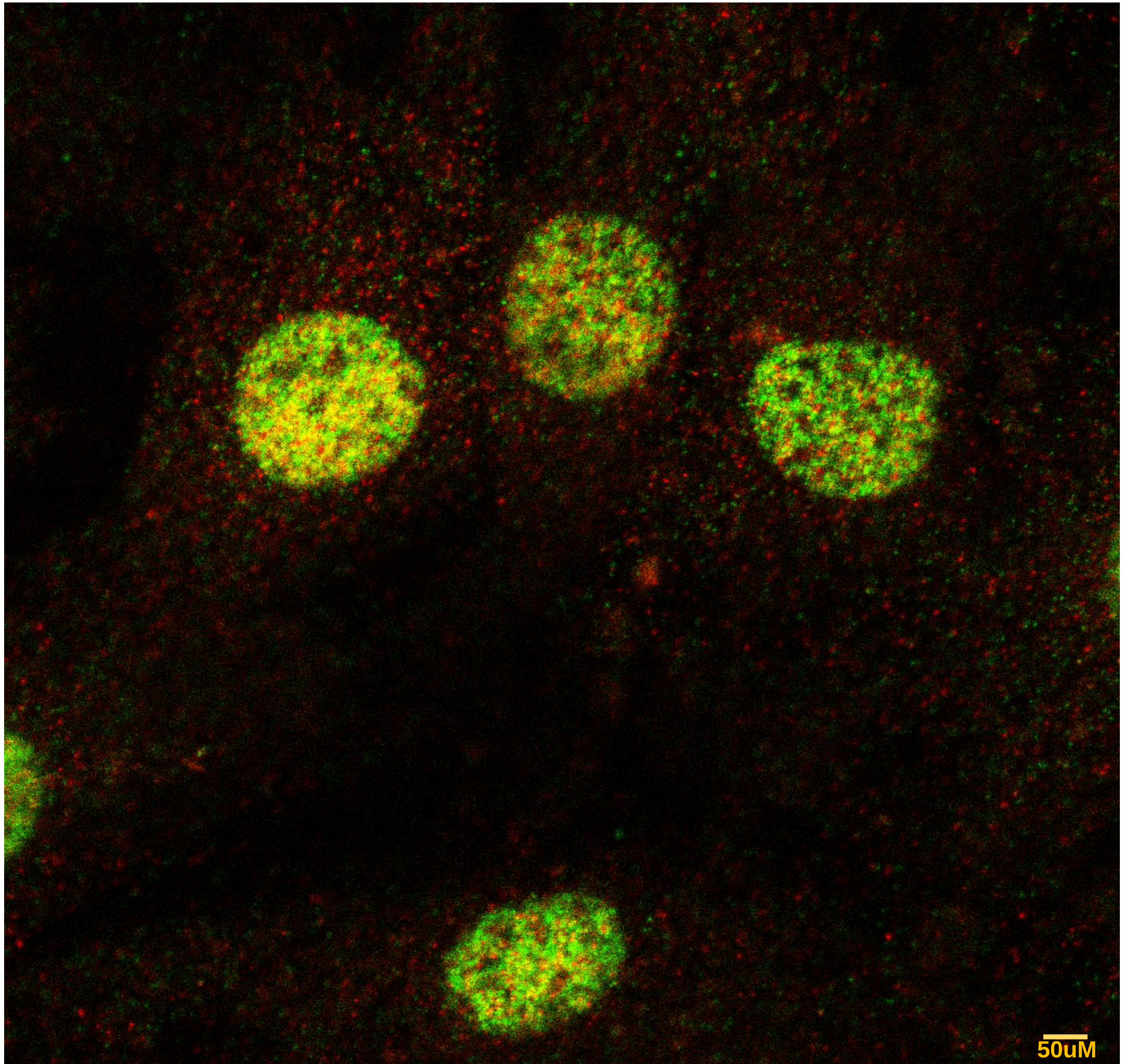

**AM + RAP KD**

**MERGED**

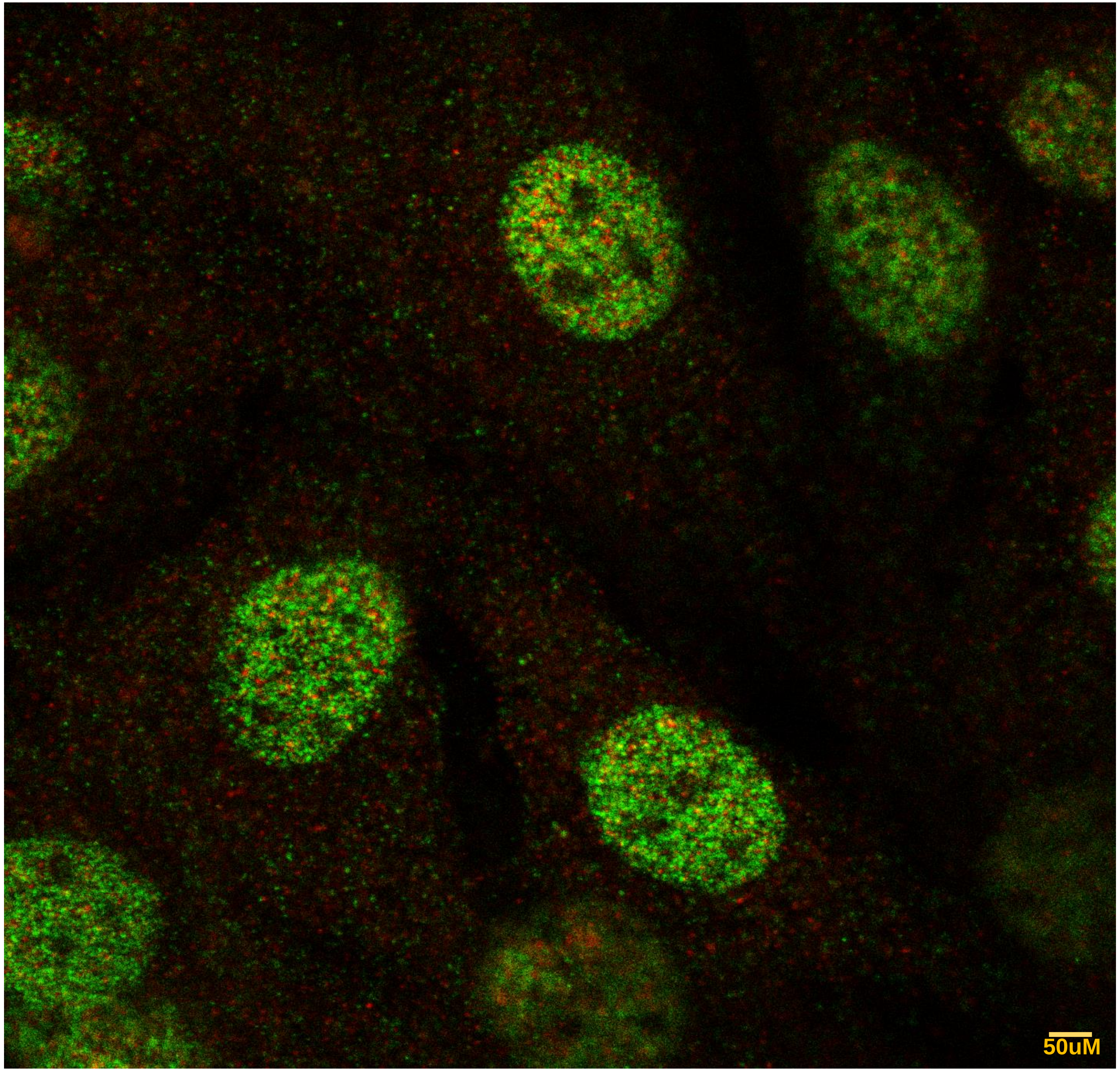

UND MERGED + DAPI

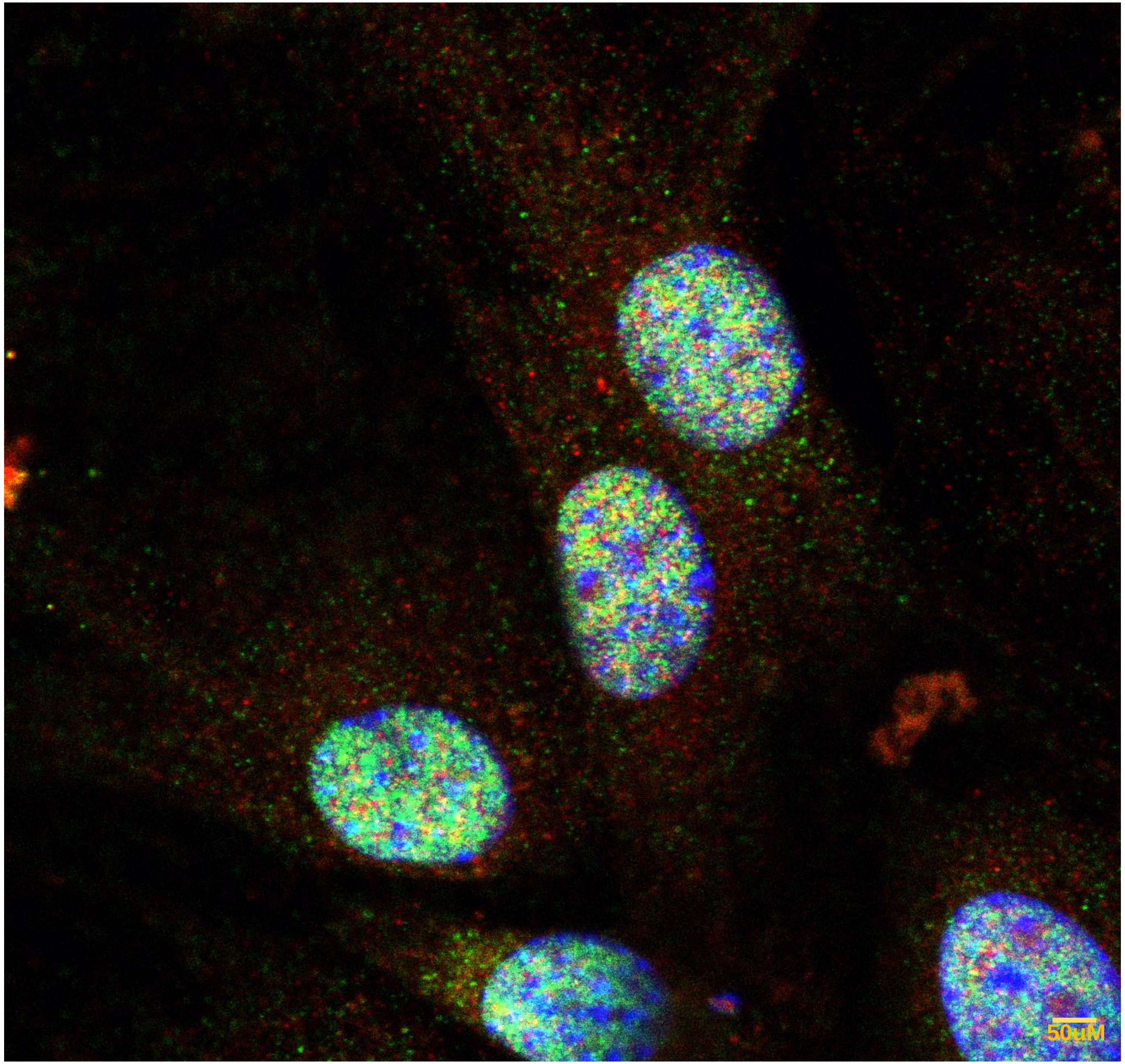

**AM + Scr** **MERGED + DAPI**

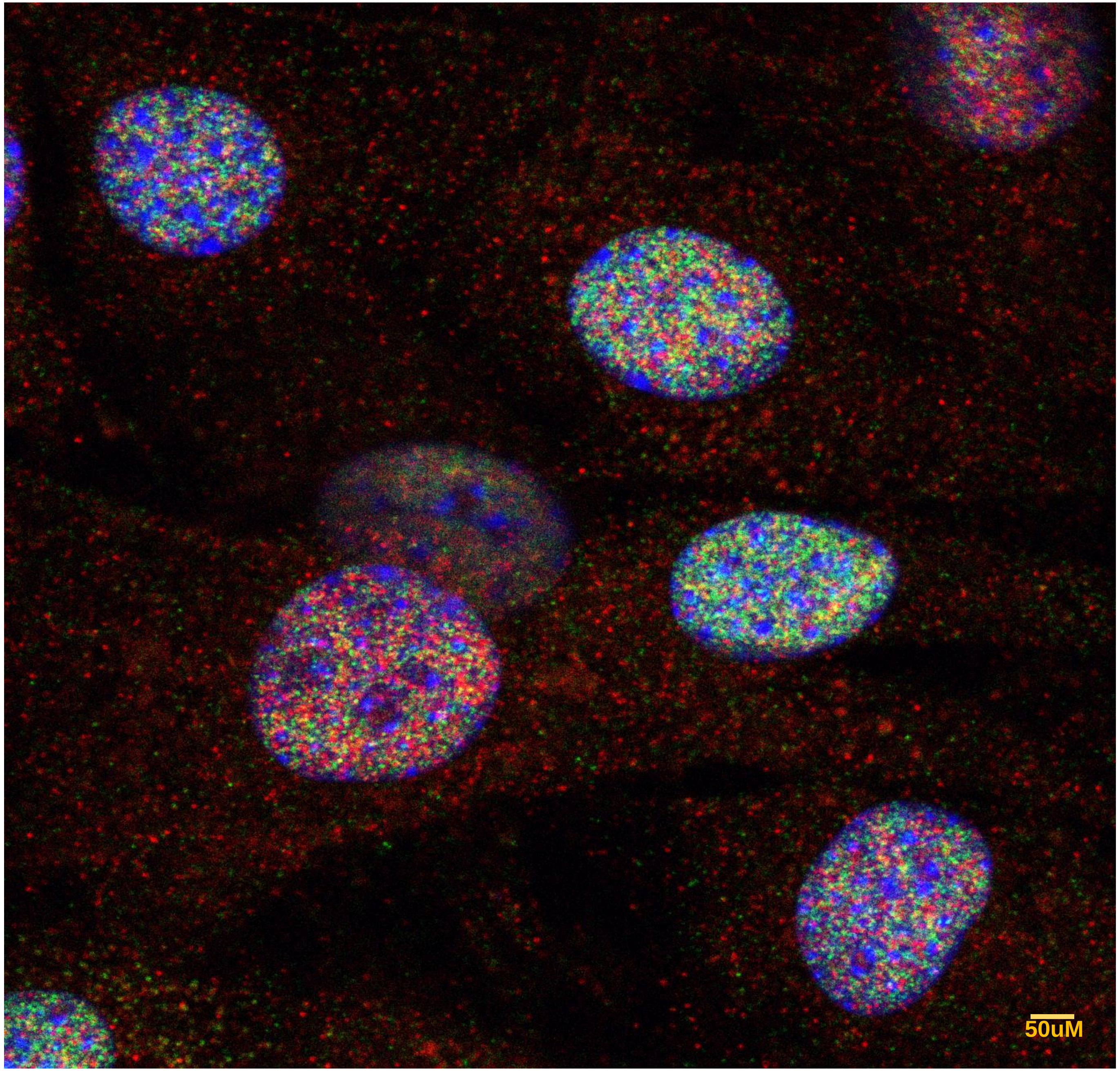

AM + RIC KD **MERGED + DAPI**

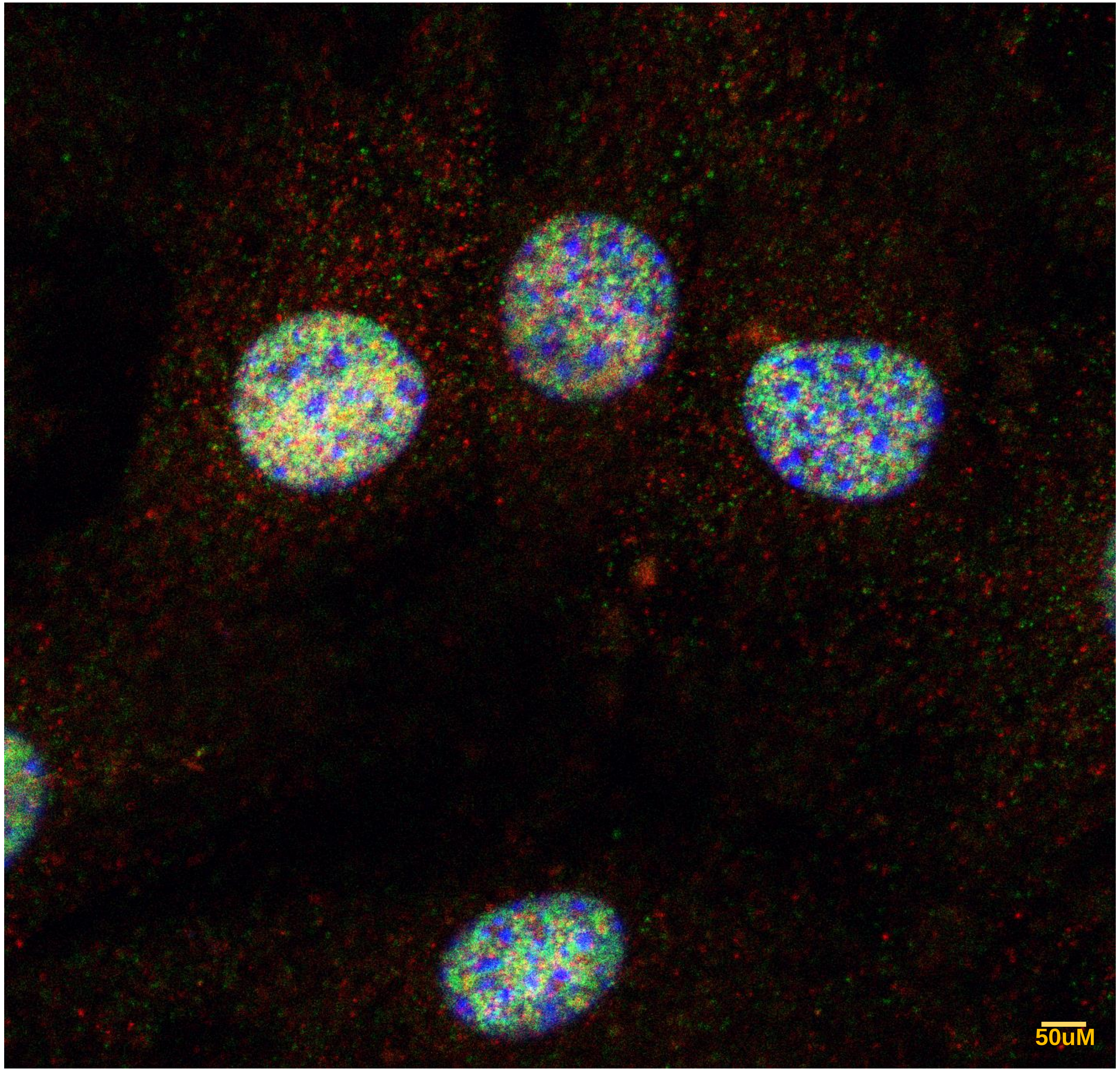

# AM + RAP KD MERGED + DAPI

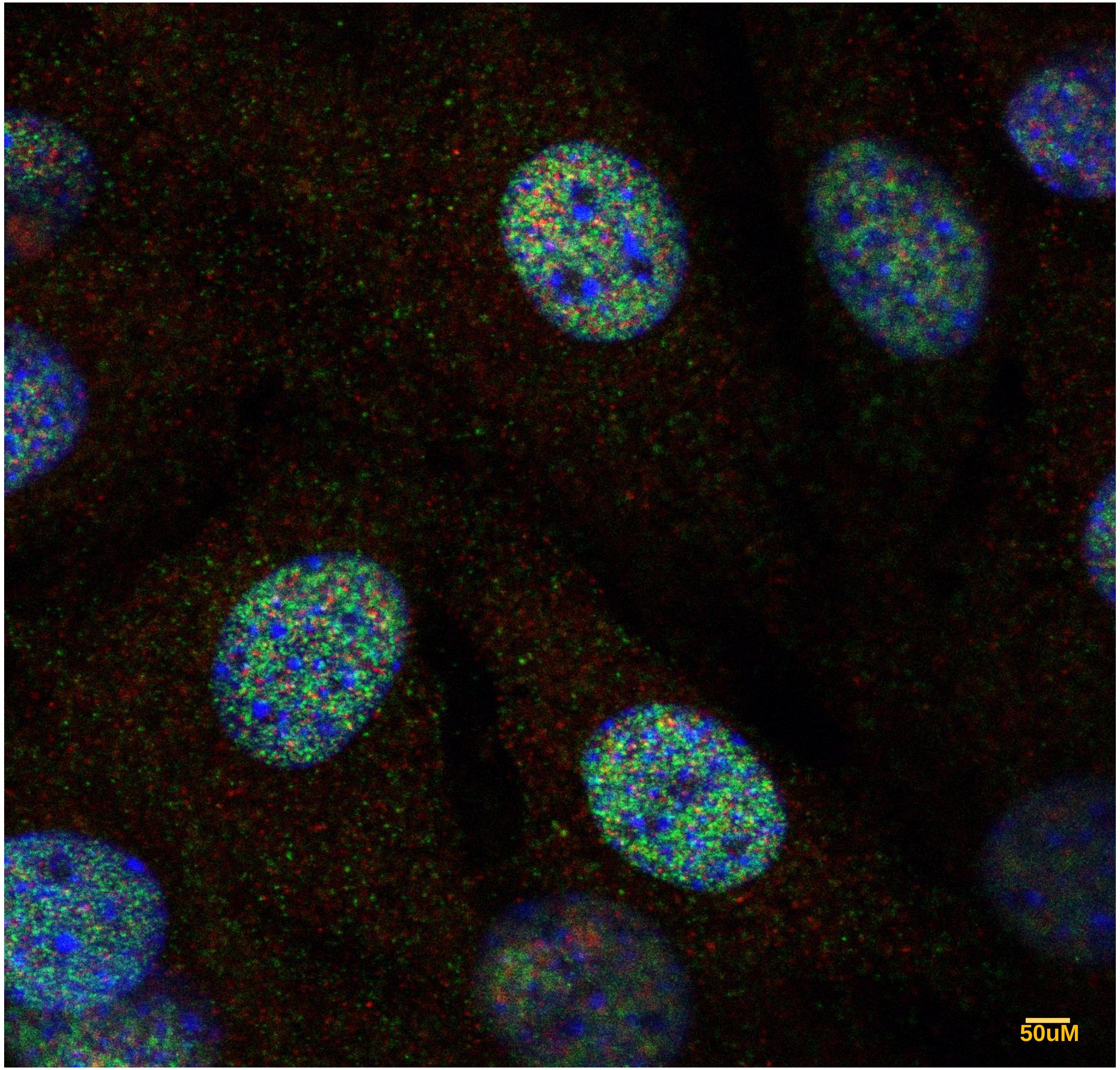

Fig.3 C)

D-0 AM + Scr

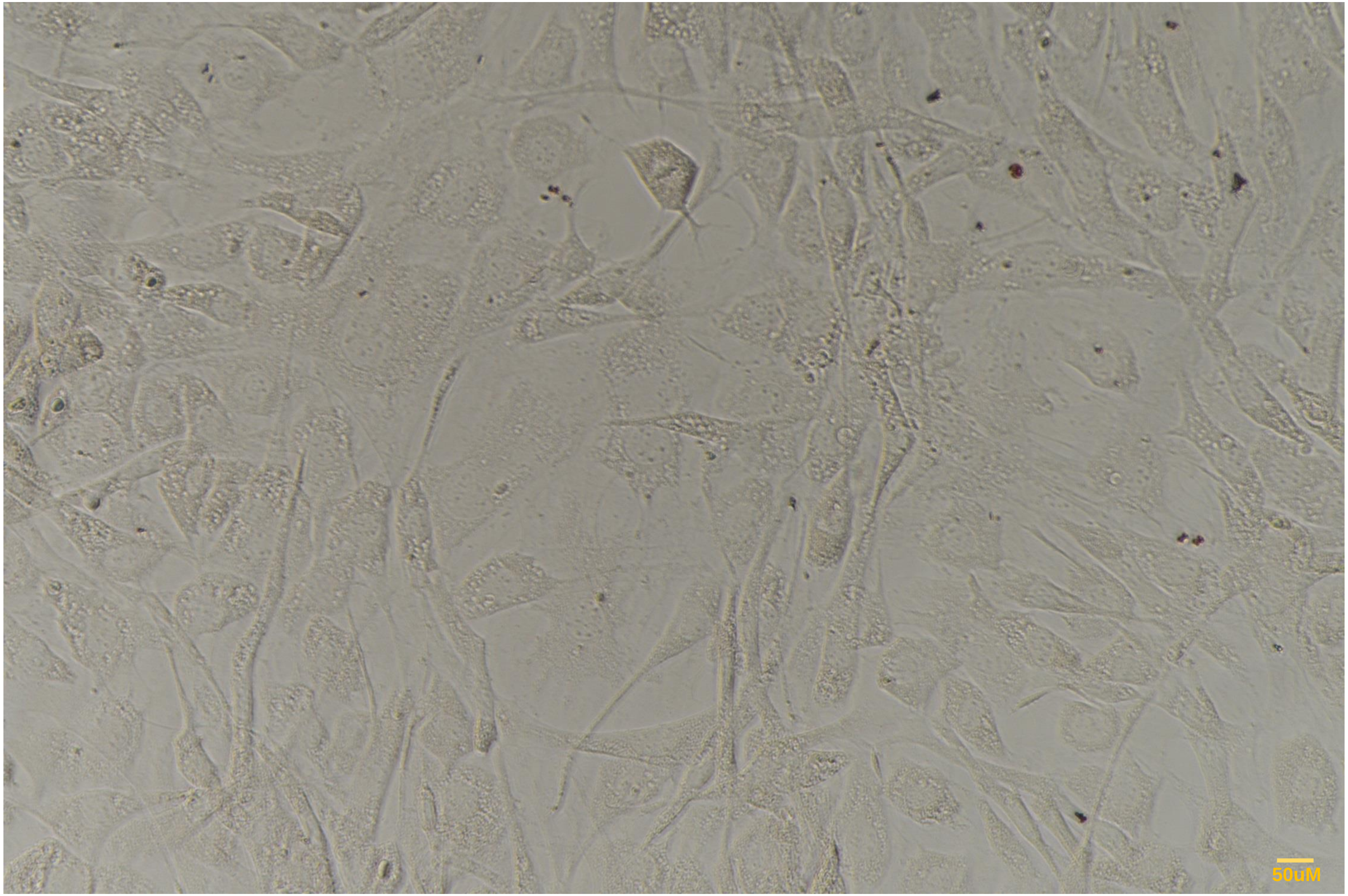

D-8

AM + Scr

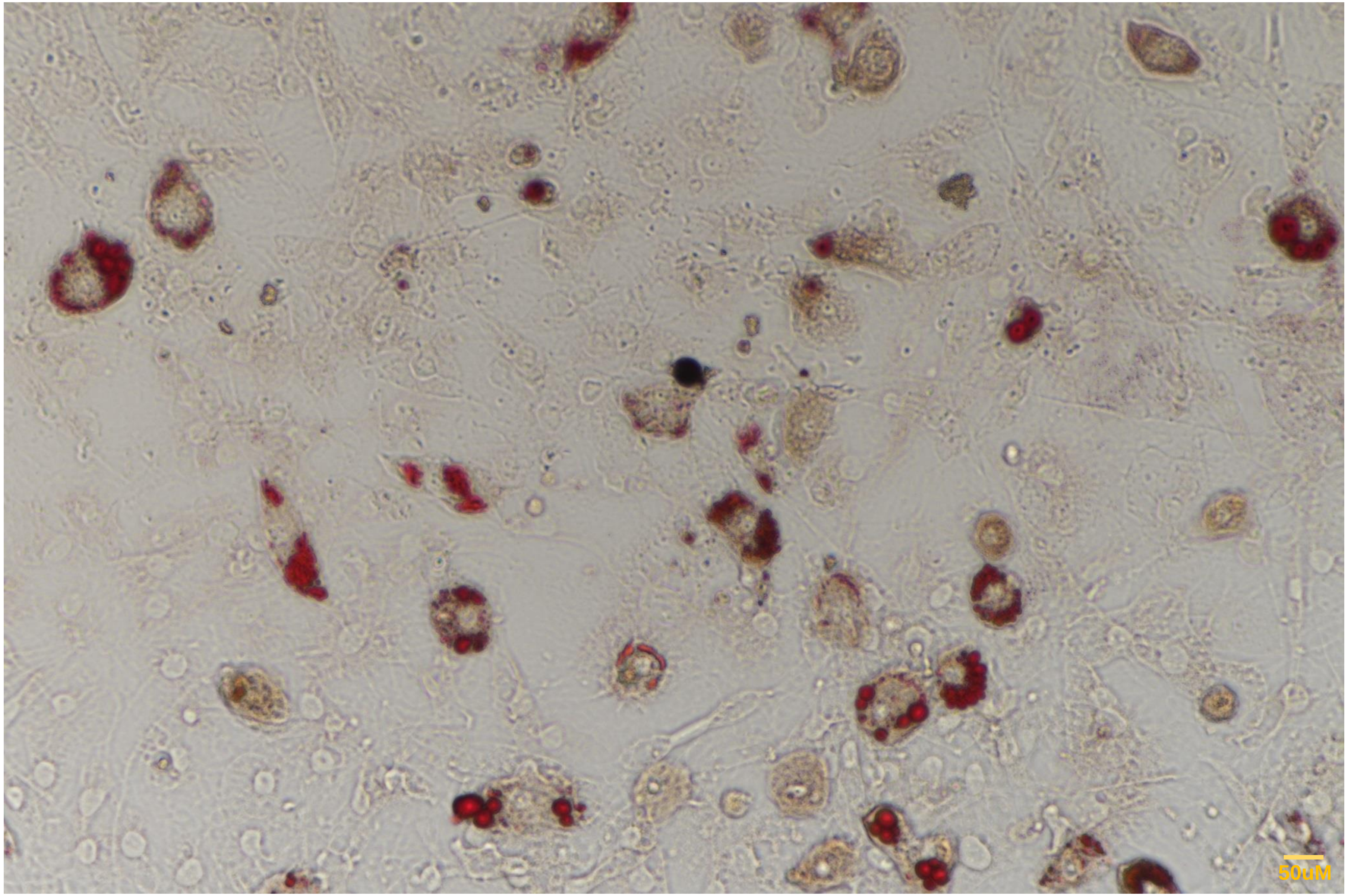

D-0 AM + Scr + LiCl

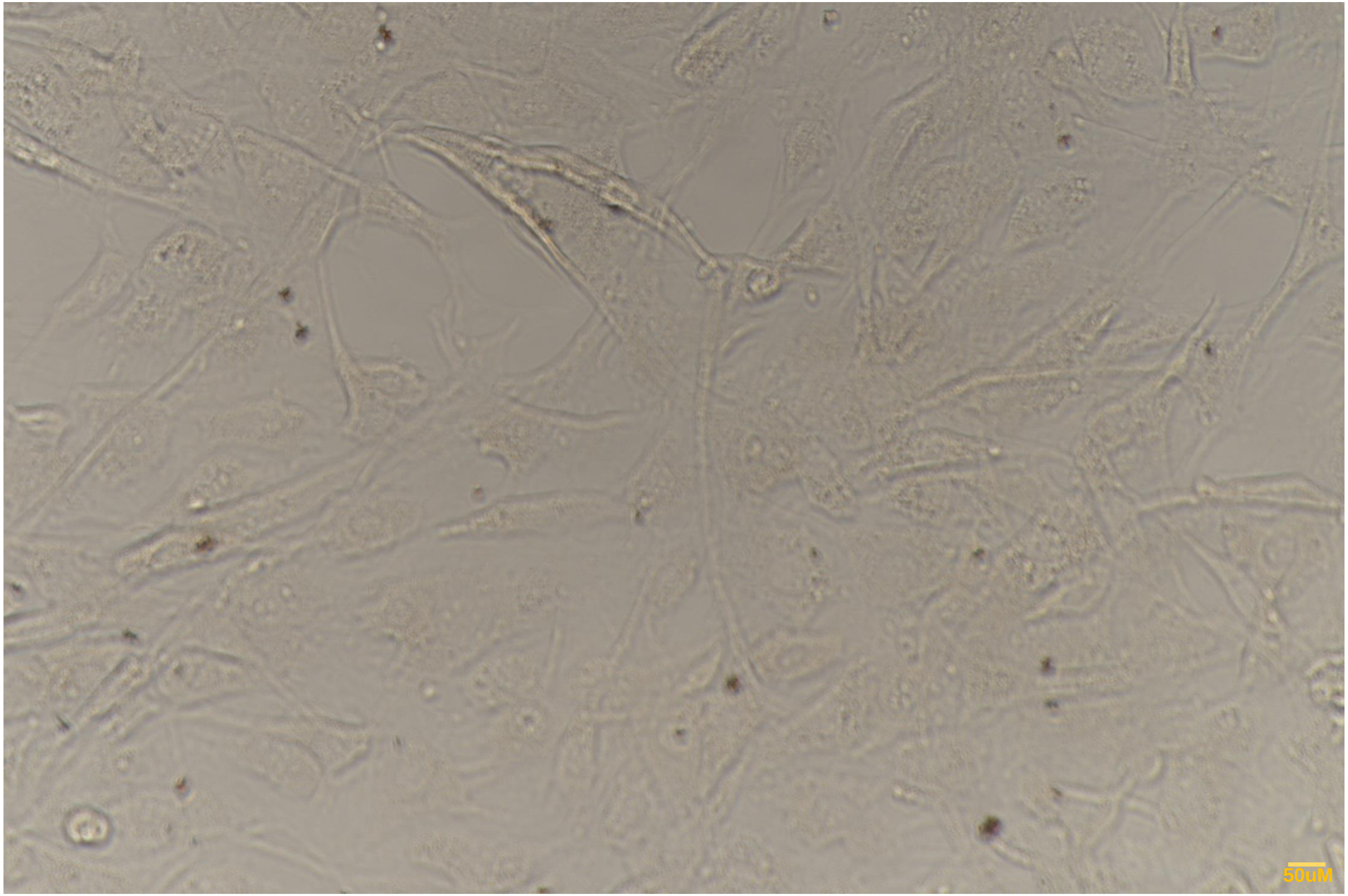

**D-8** **AM + Scr + LiCl**

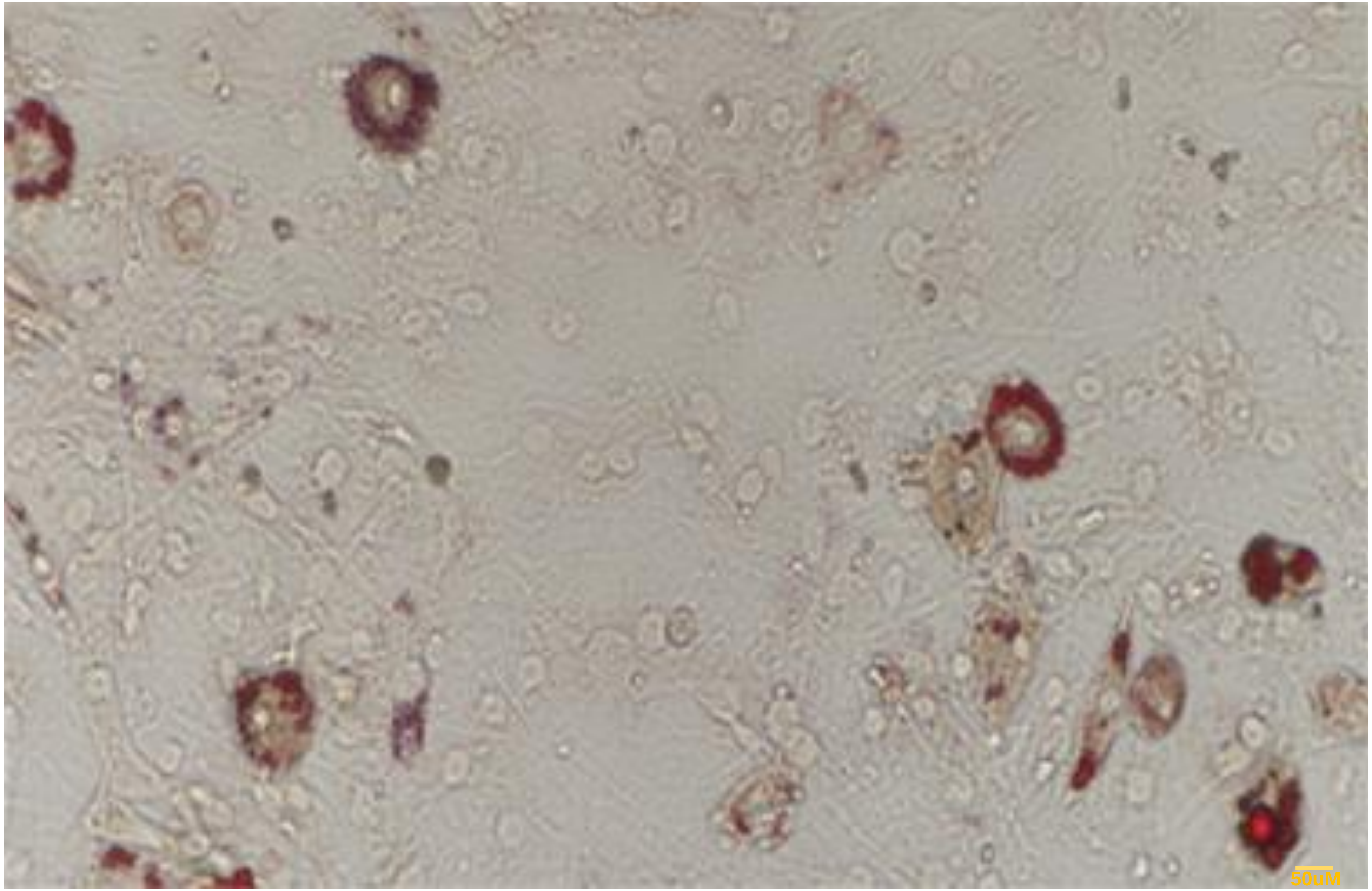

D-0 AM + RIC KD

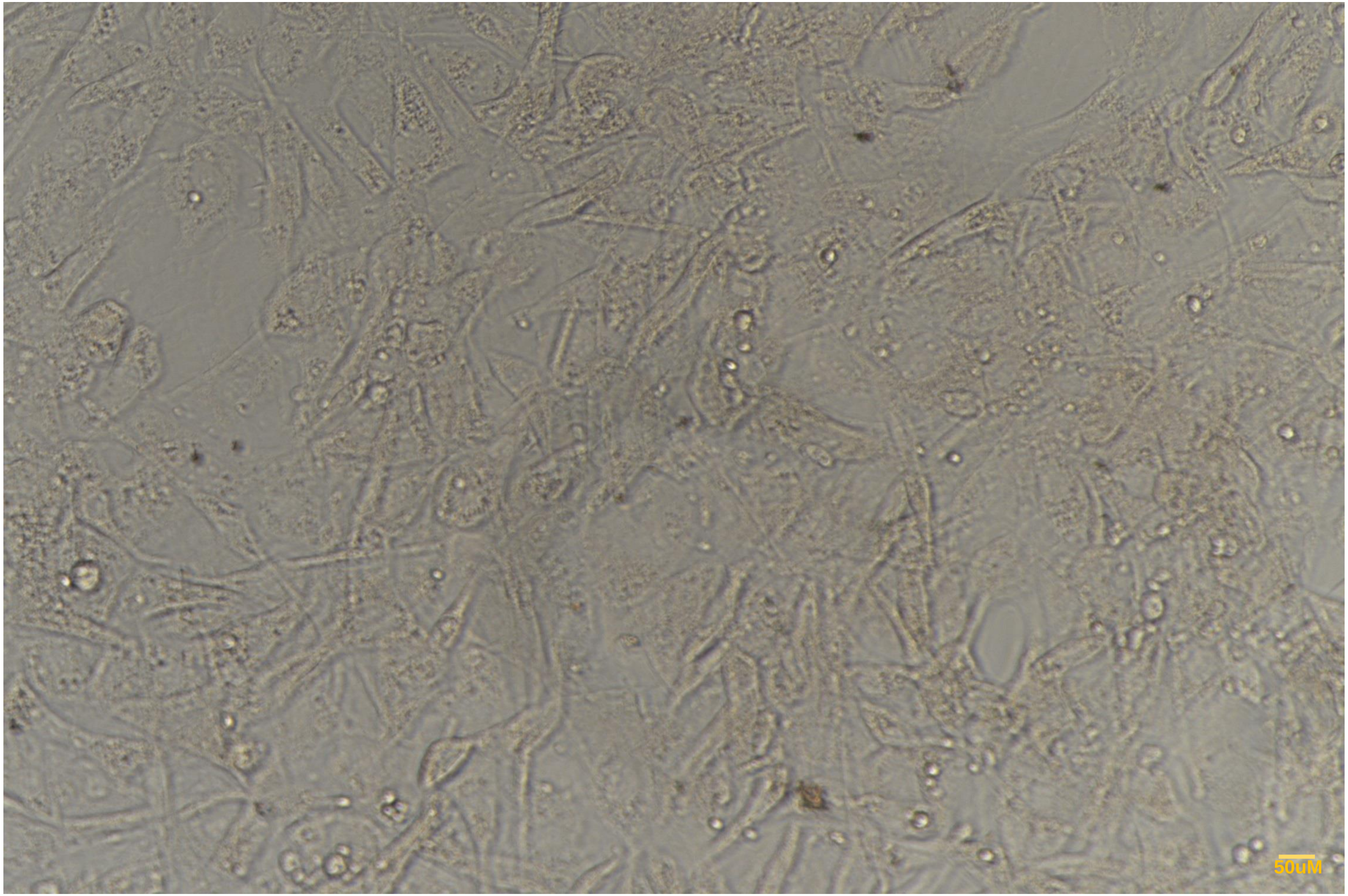

**D-8** **AM + RIC KD**

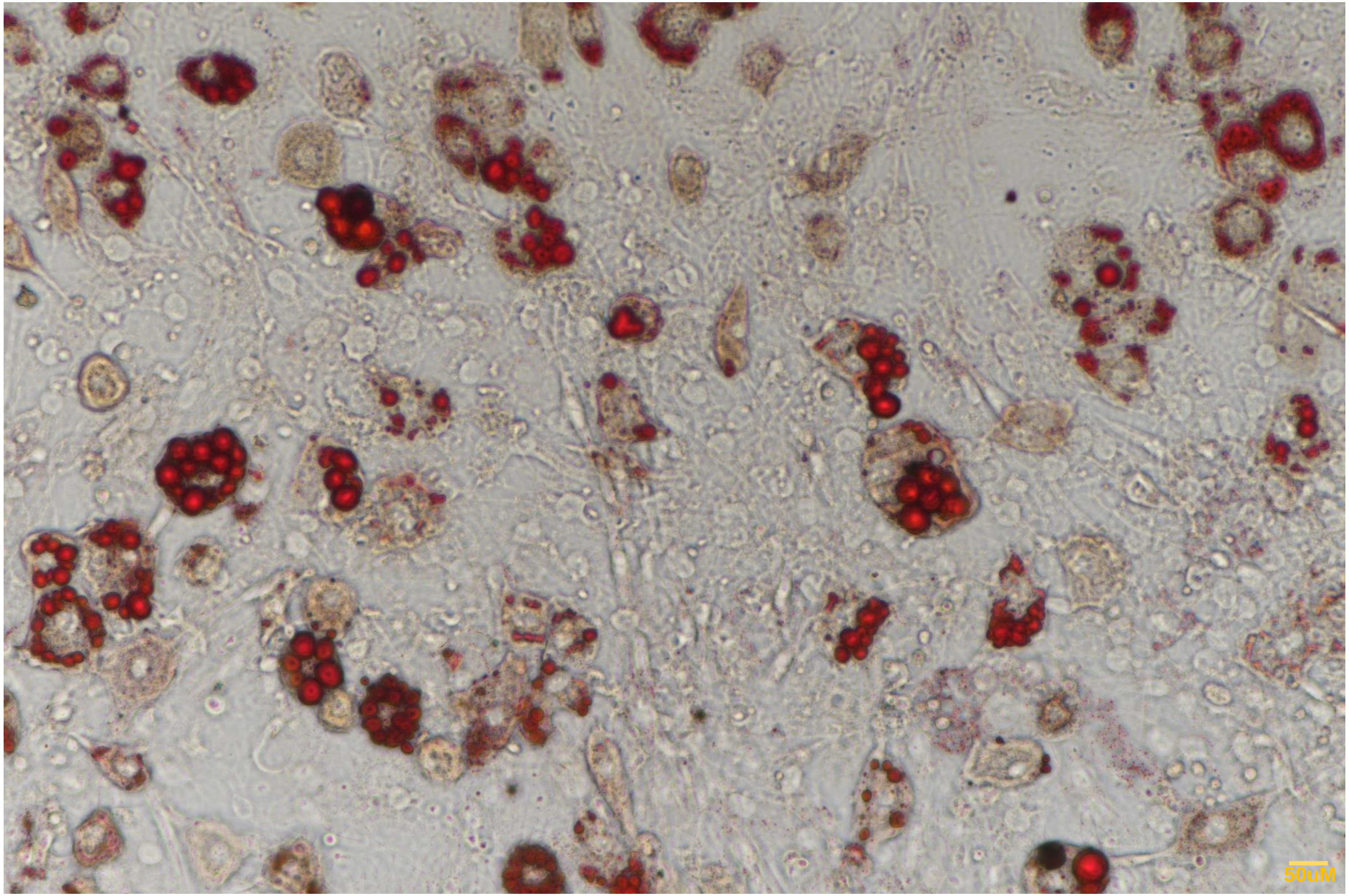

**D-0** **AM + RIC KD + LiCl**

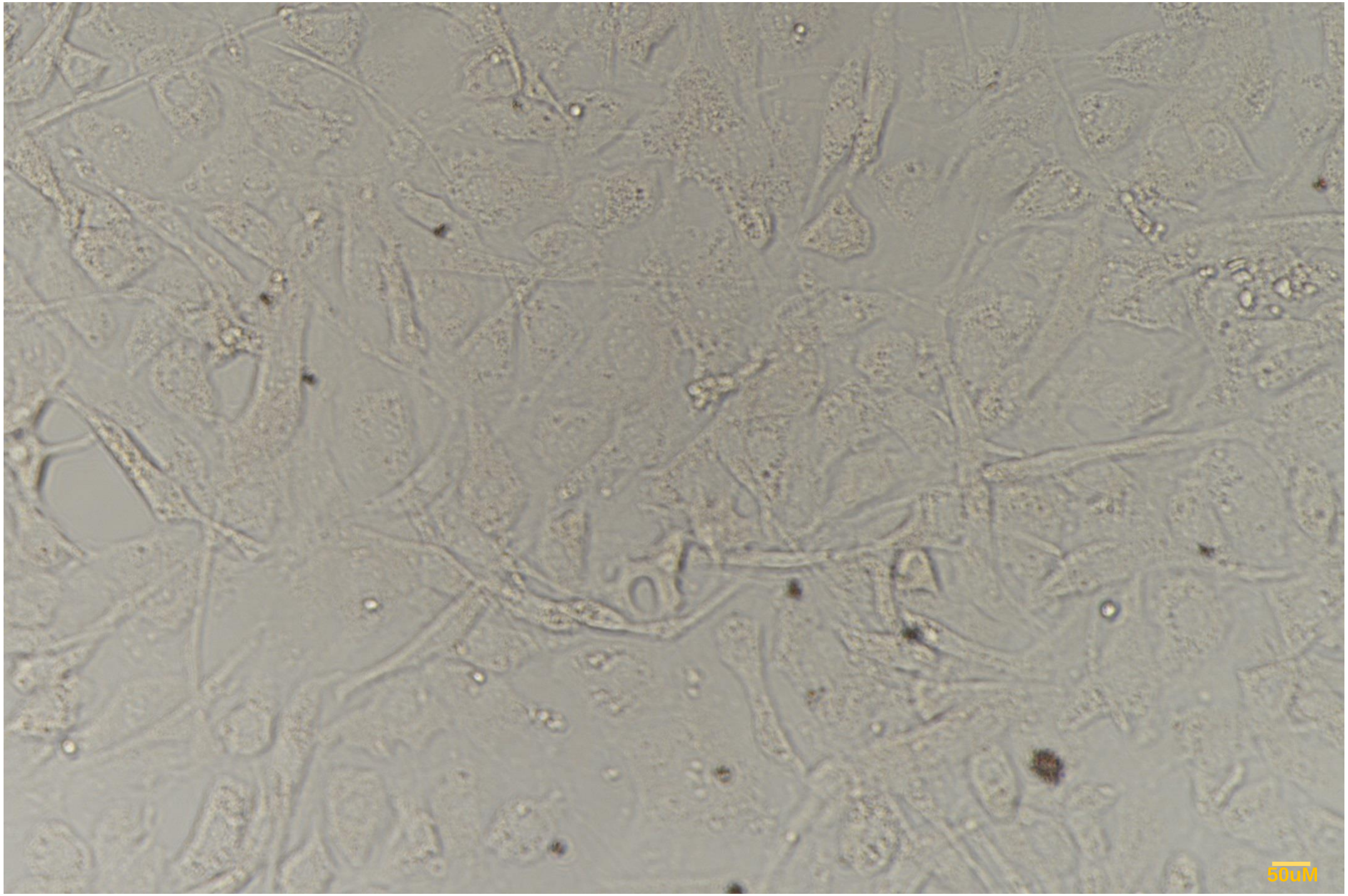

**D-8** **AM + RIC KD + LiCl**

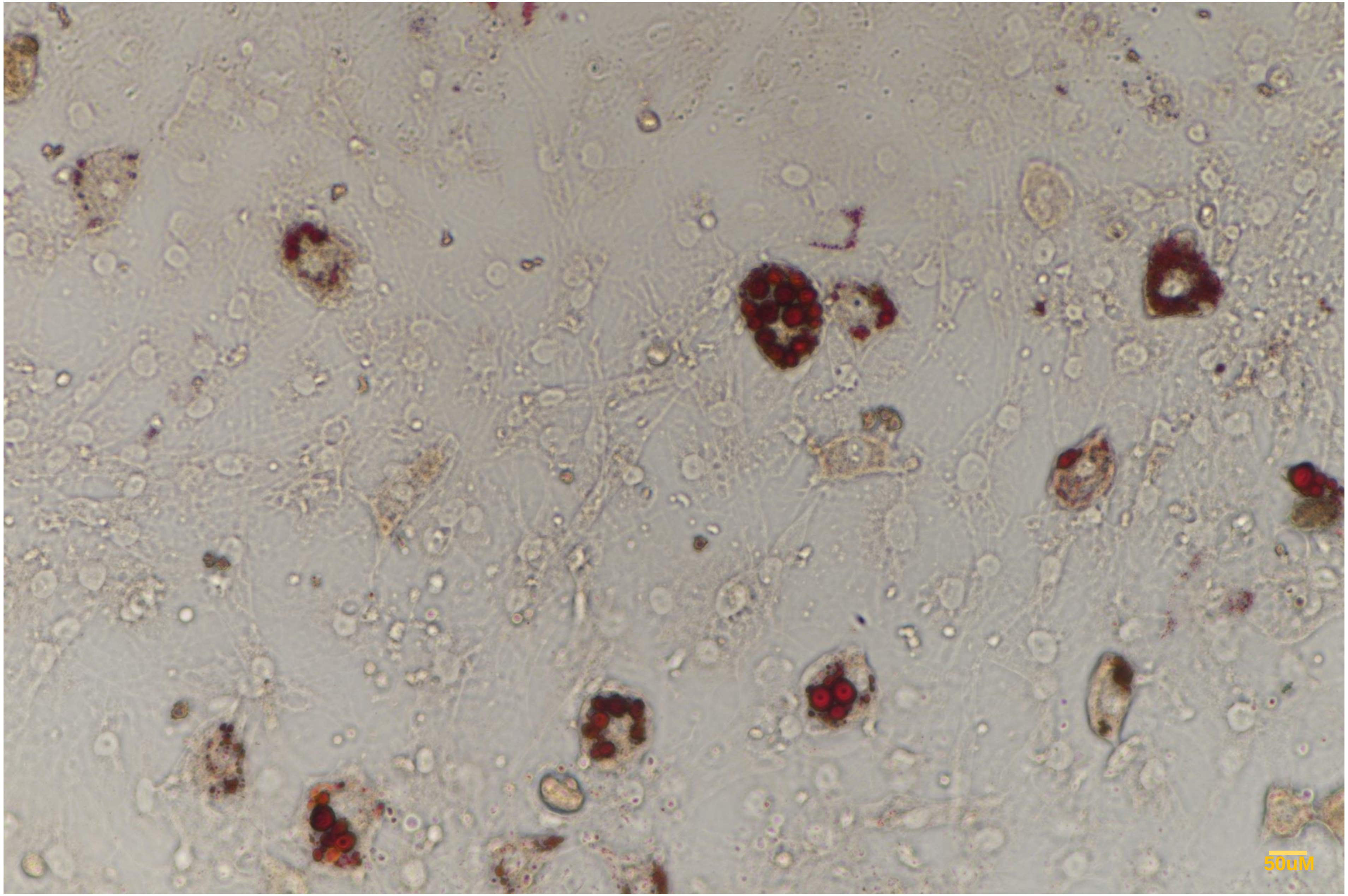

Fig.4 D)

D-0 AM

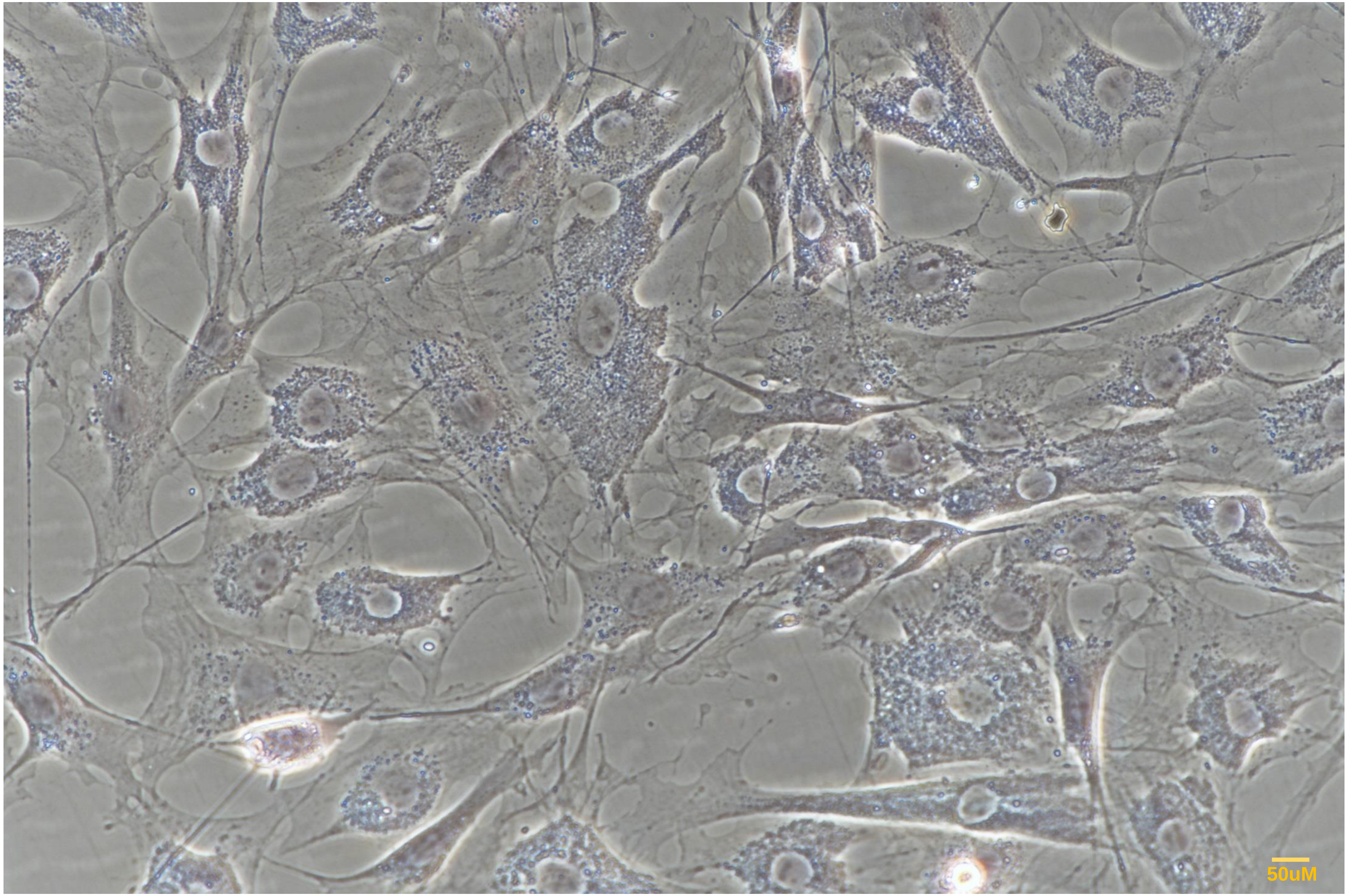

D-8 **AM**

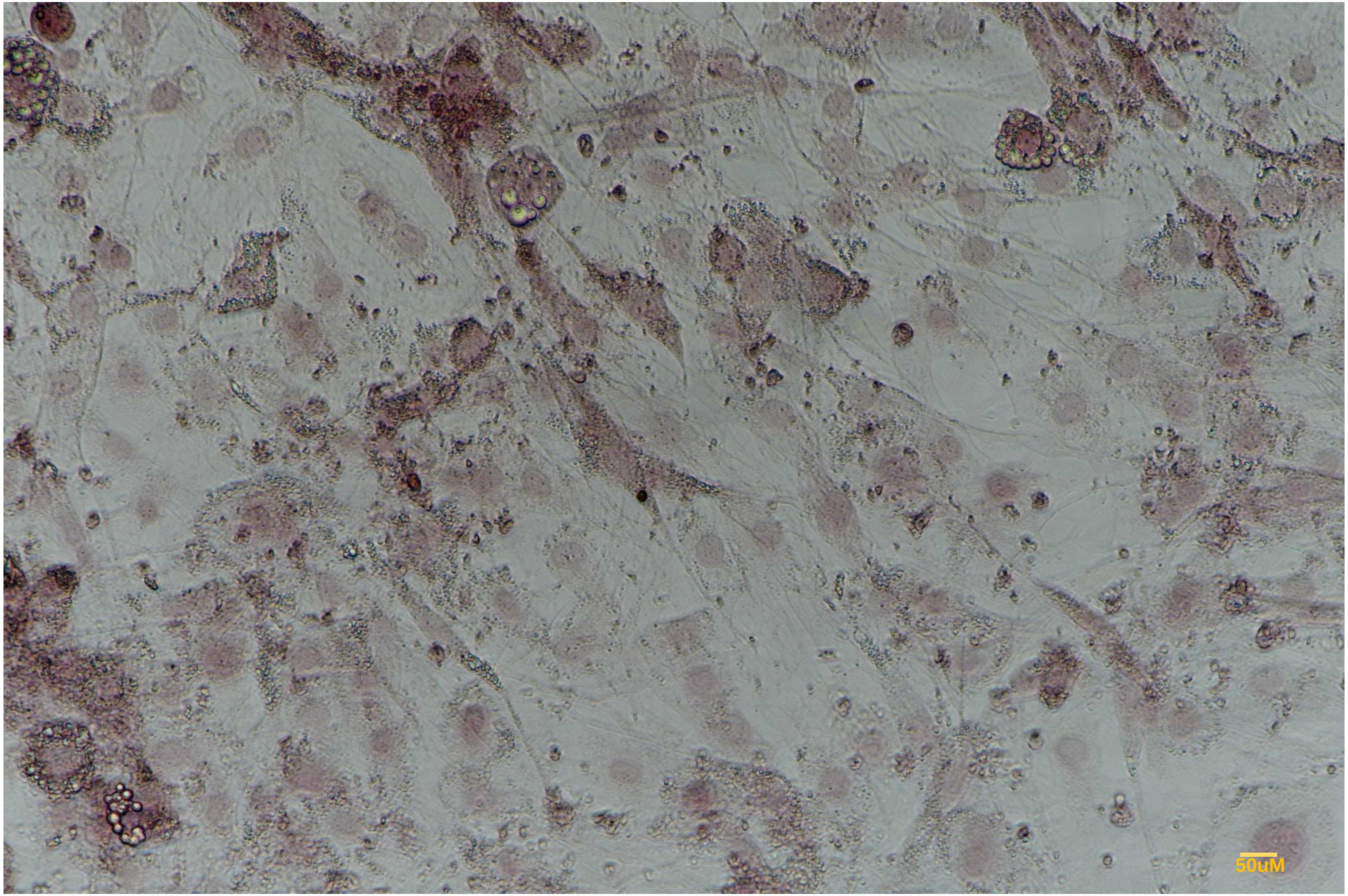

**D-0** **AM + LiCl**

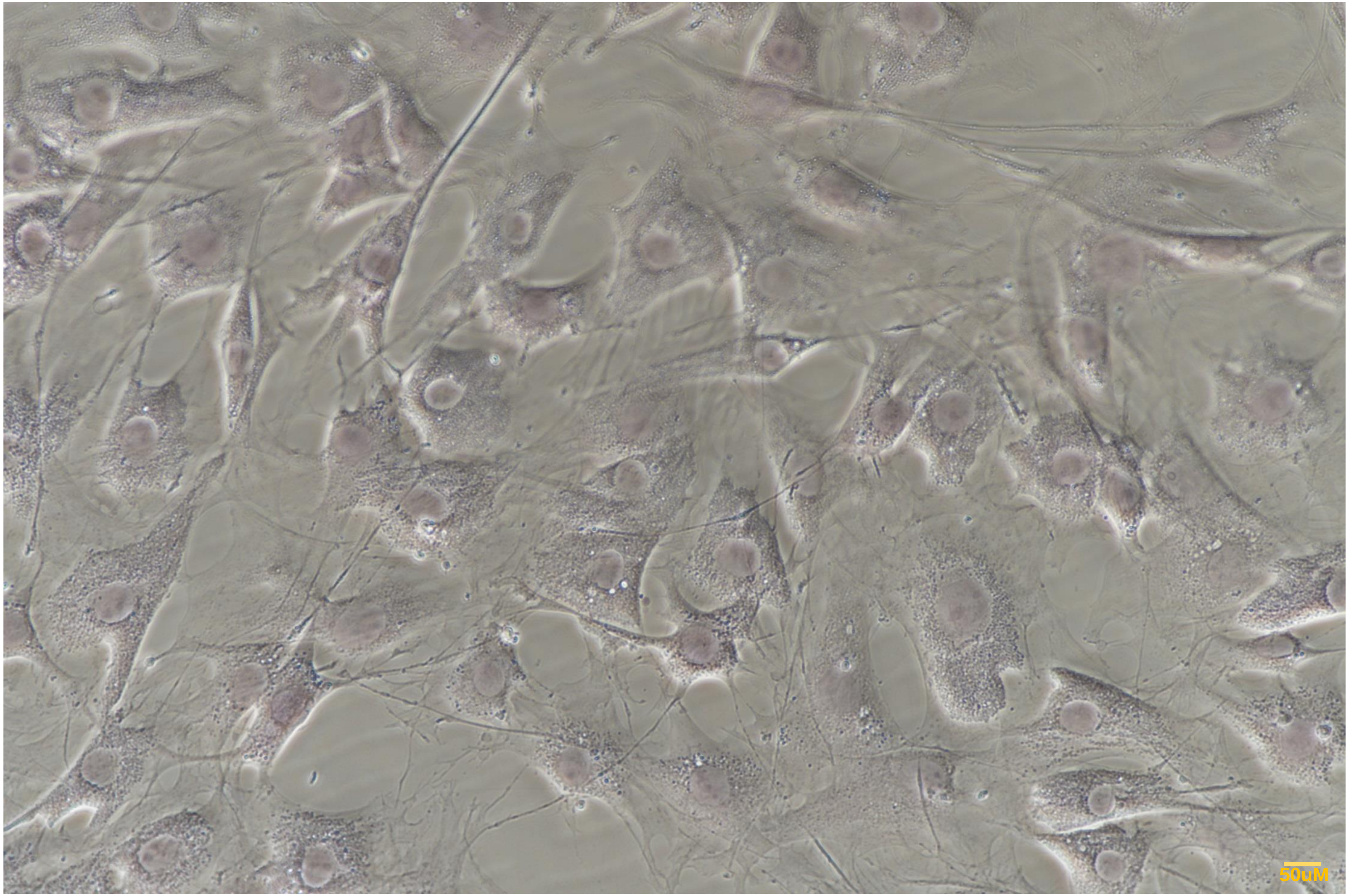

**D-8** **AM + LiCl**

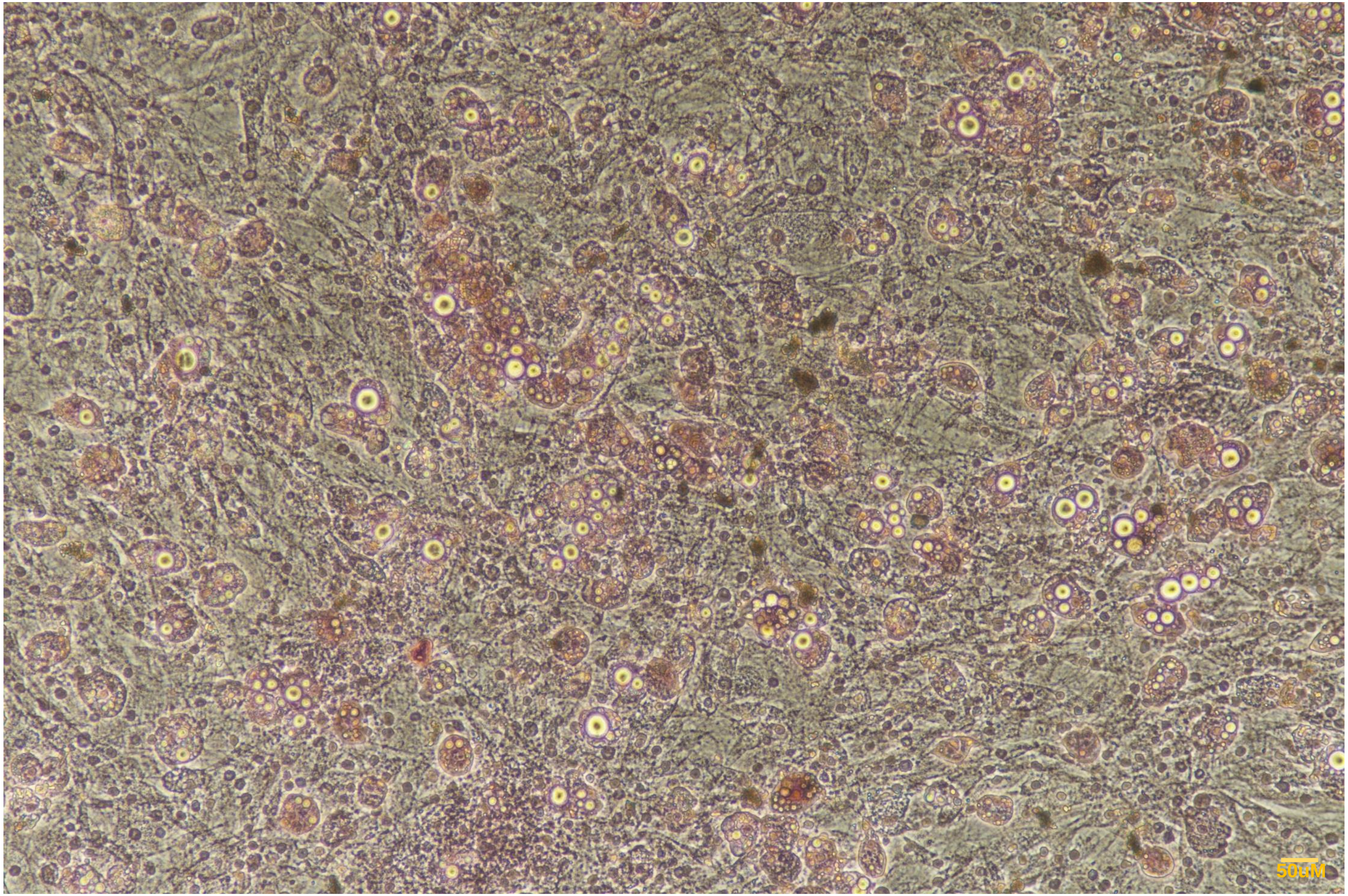

D-0 AM (LG)

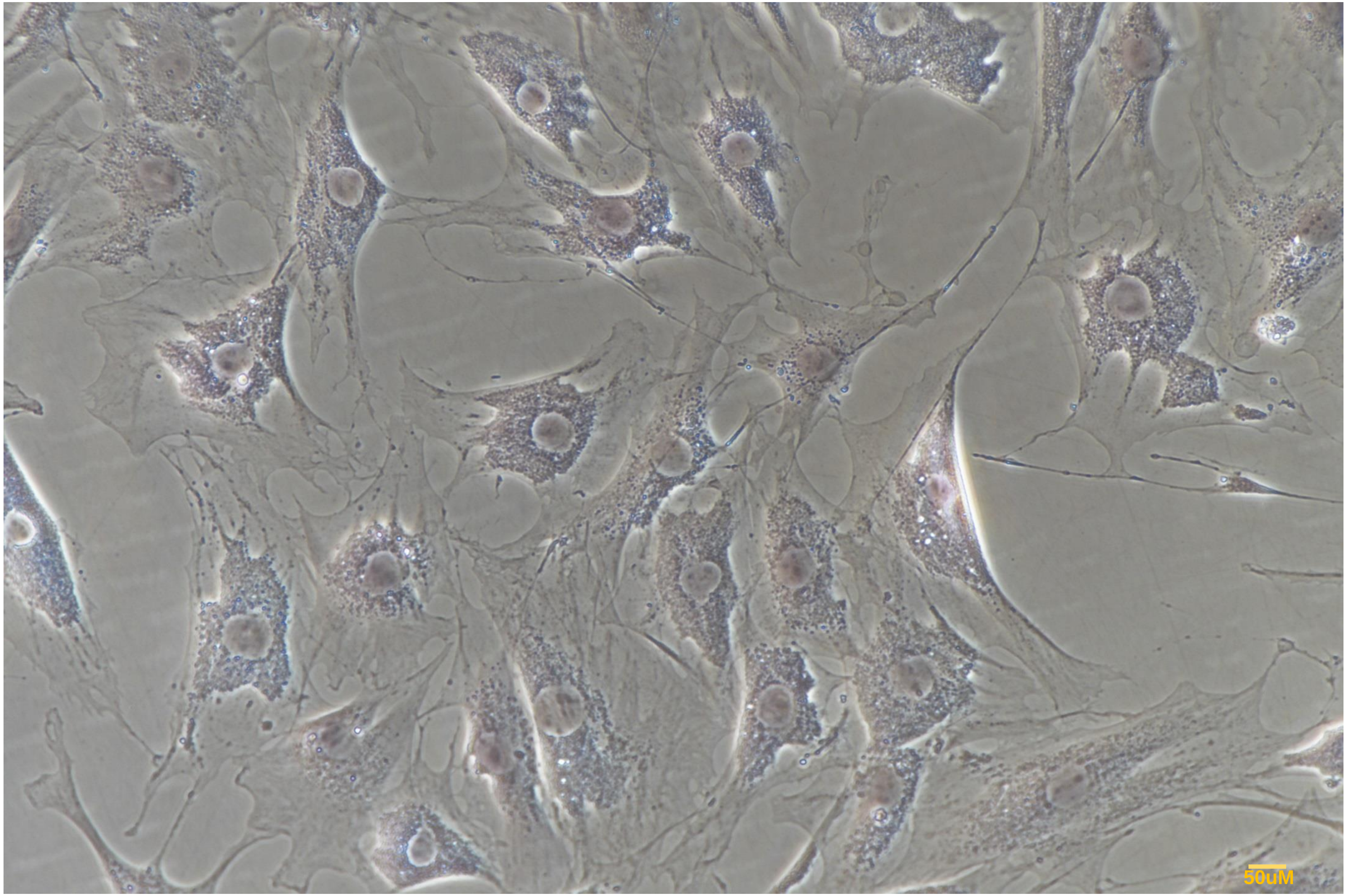

**D-8** **AM (LG)**

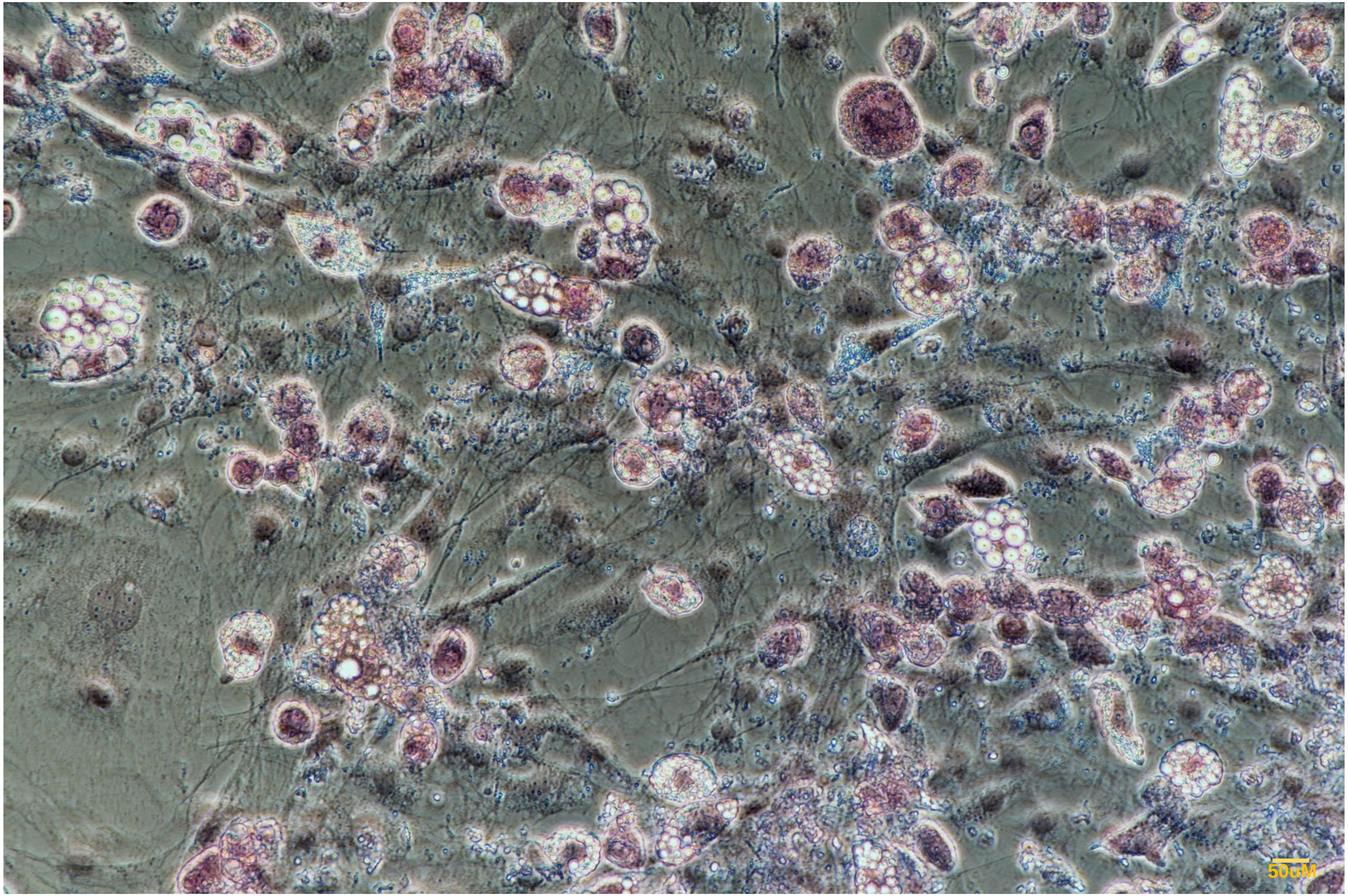

**D-0** **AM (HG)**

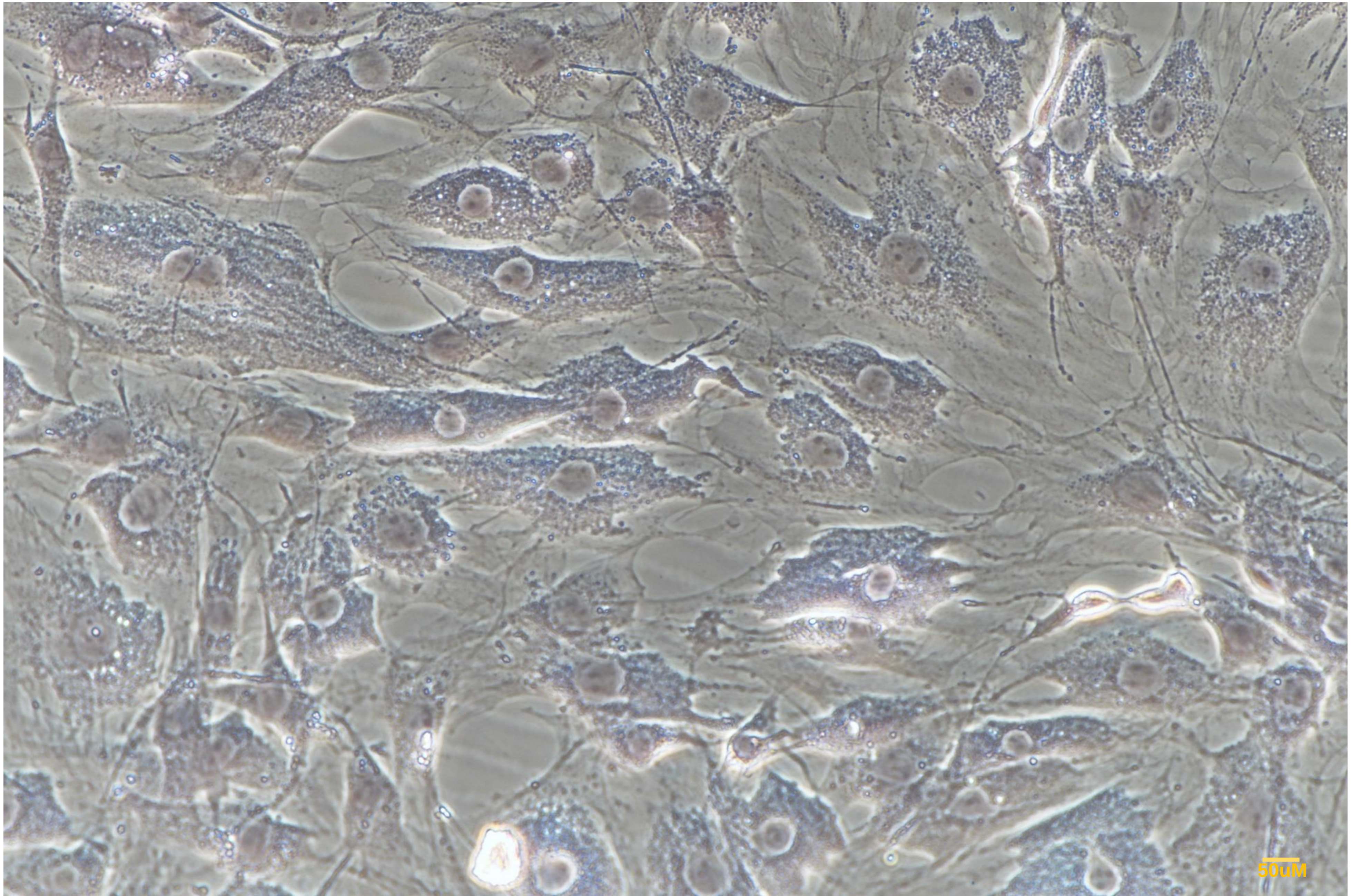

**D-8** **AM (HG)**

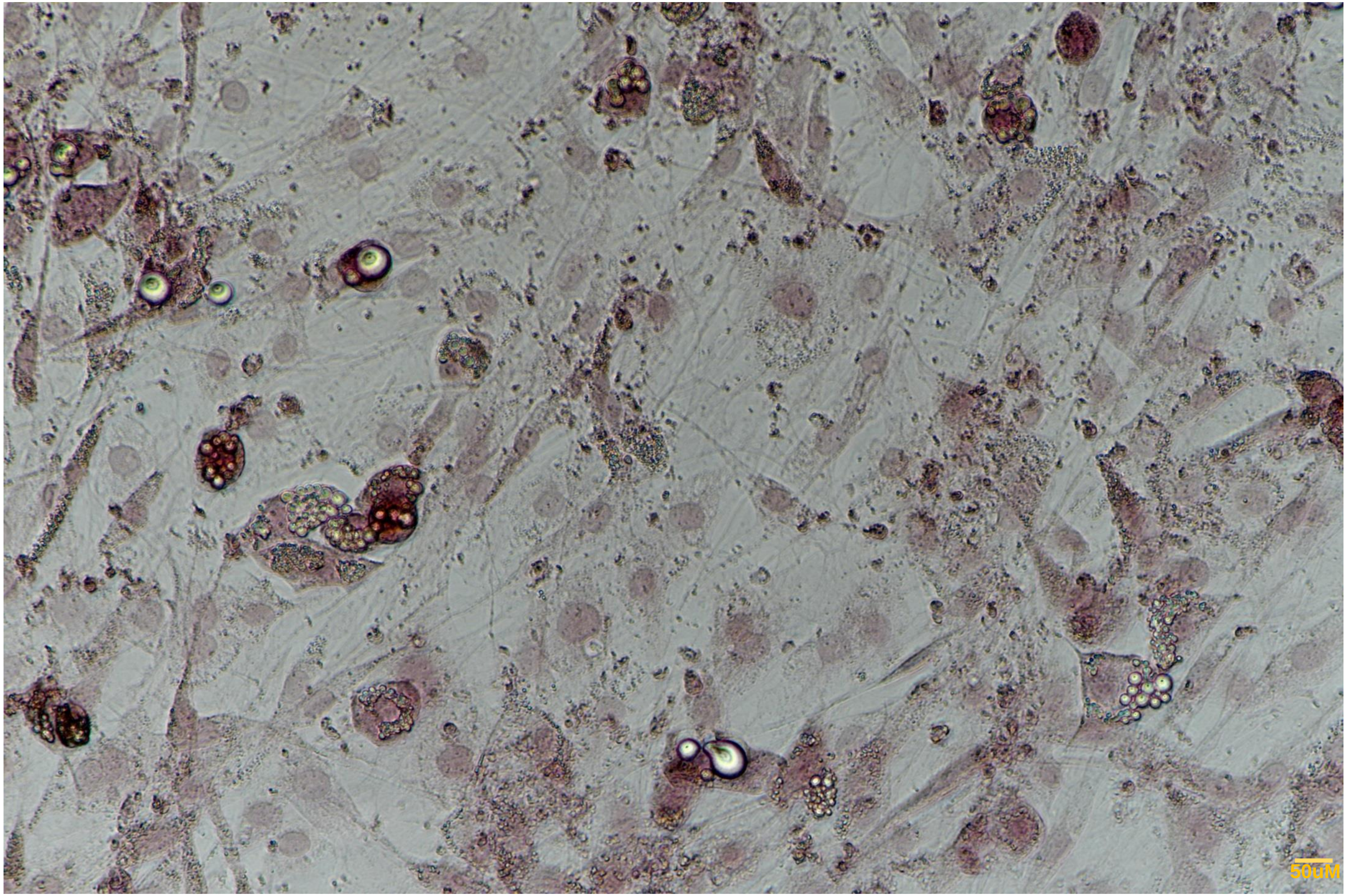

**D-0** **AM (HG + LiCl)**

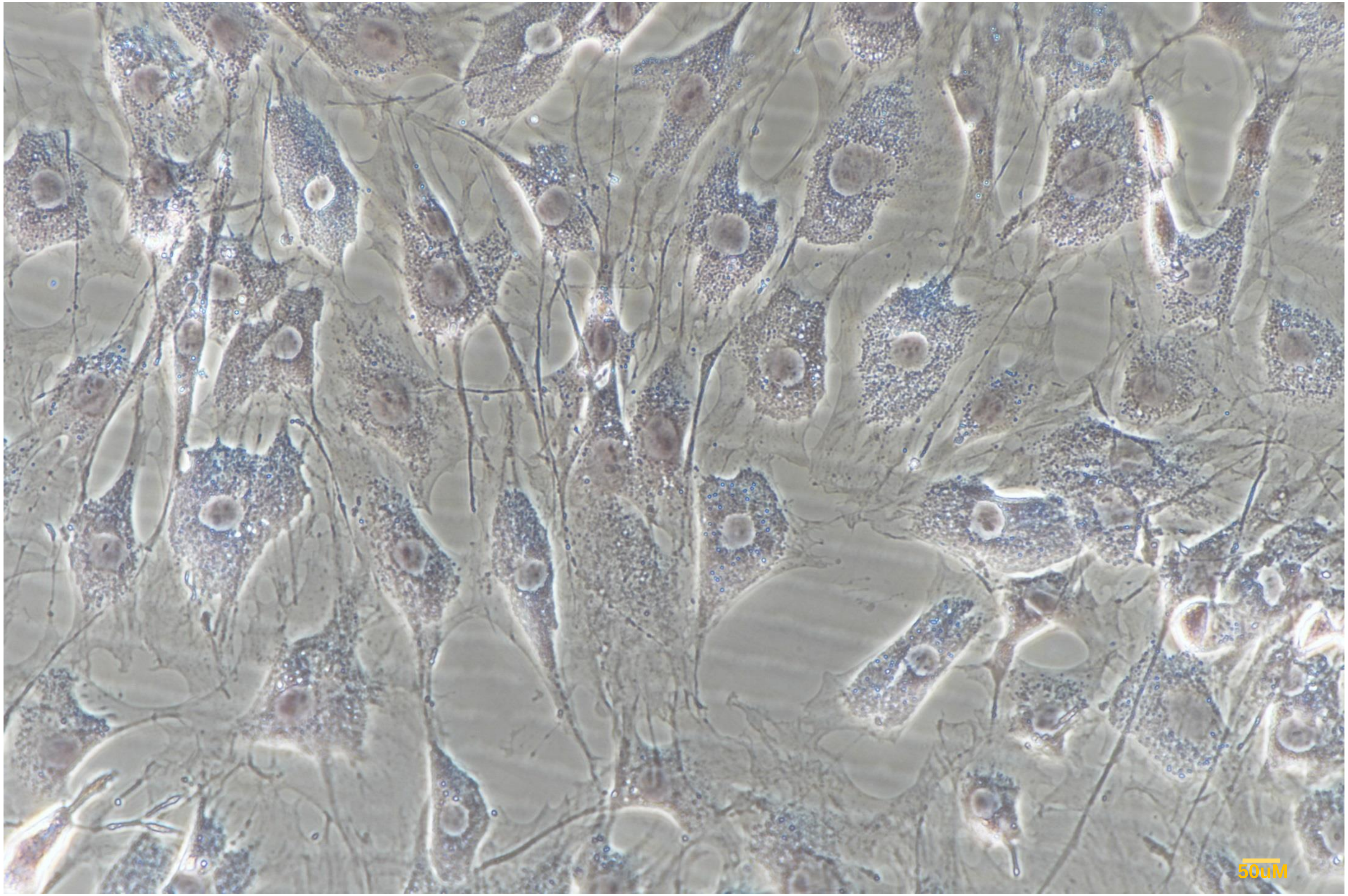

D-8 AM (HG + LiCl)

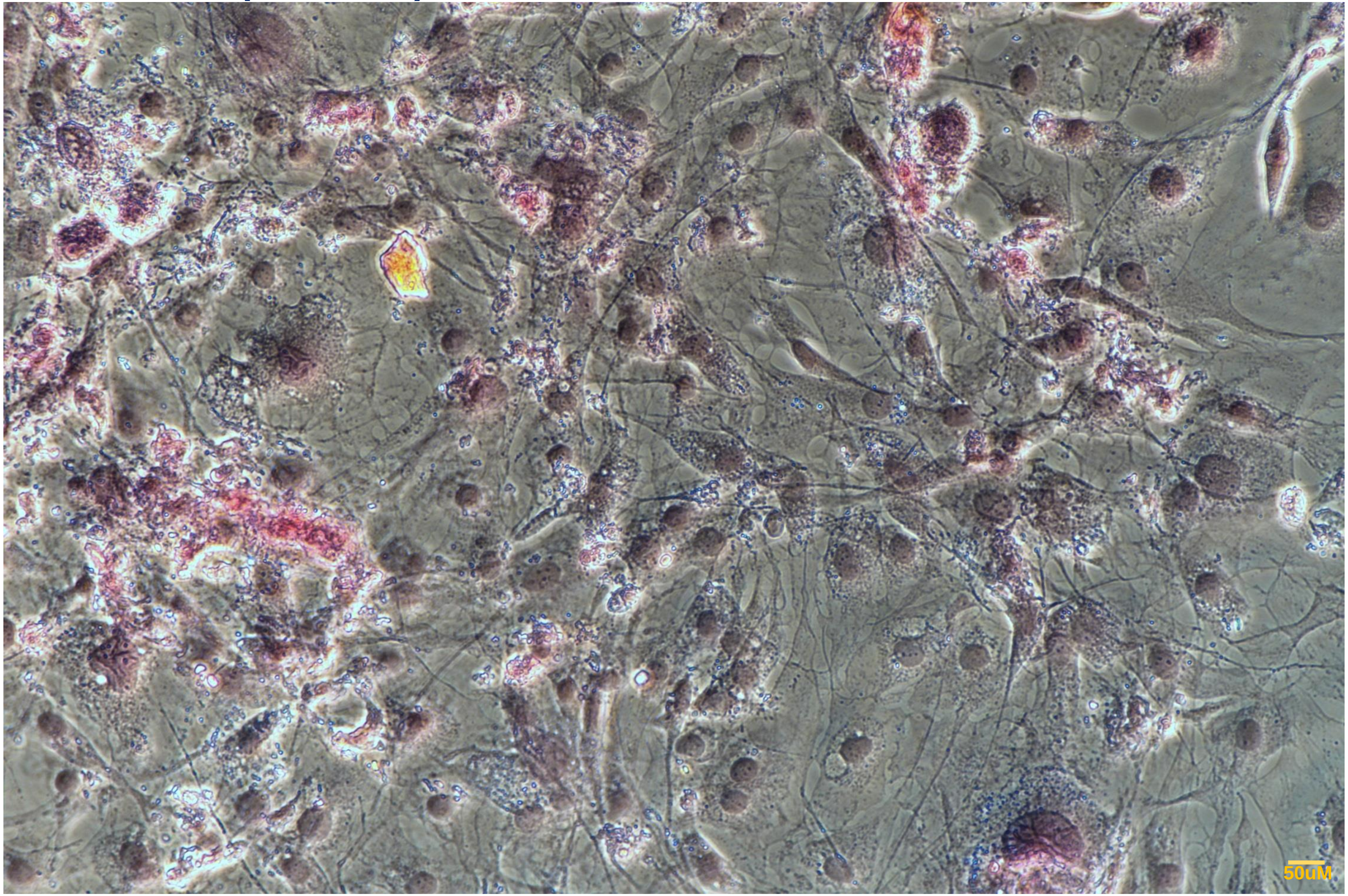

Fig.4 E)

D-8 AM

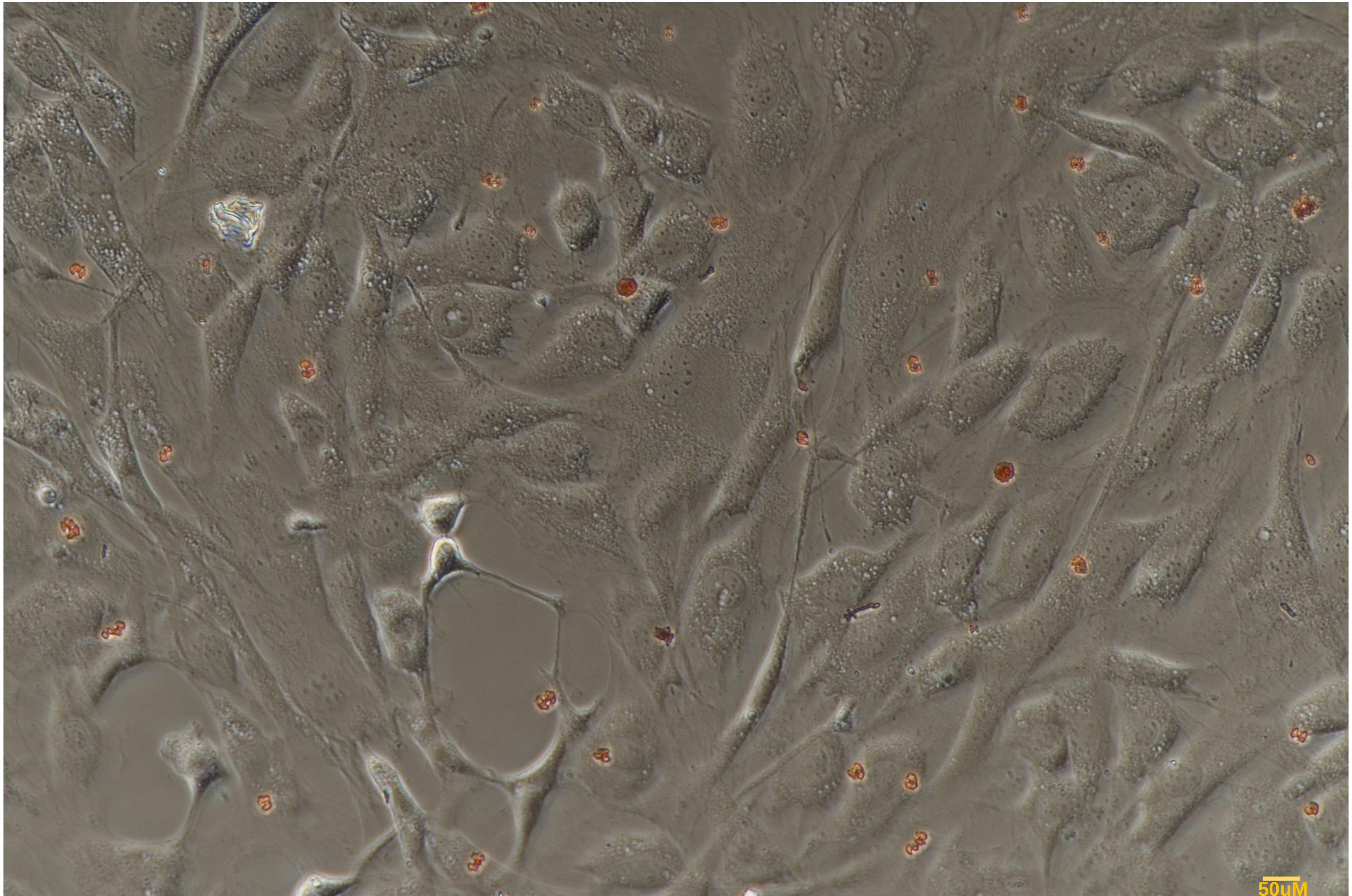

D-8 AM

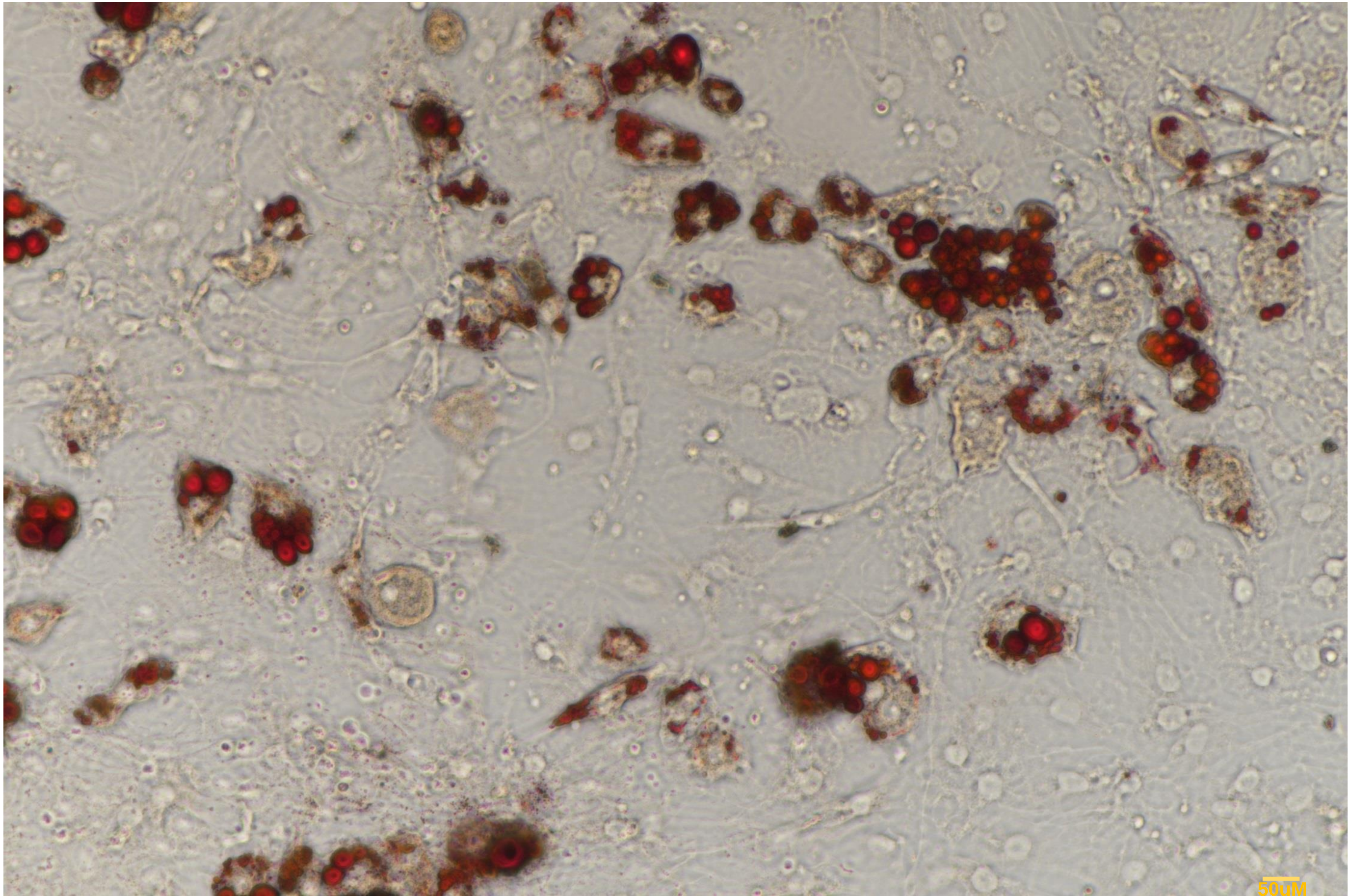

D-0 AM + LiCl

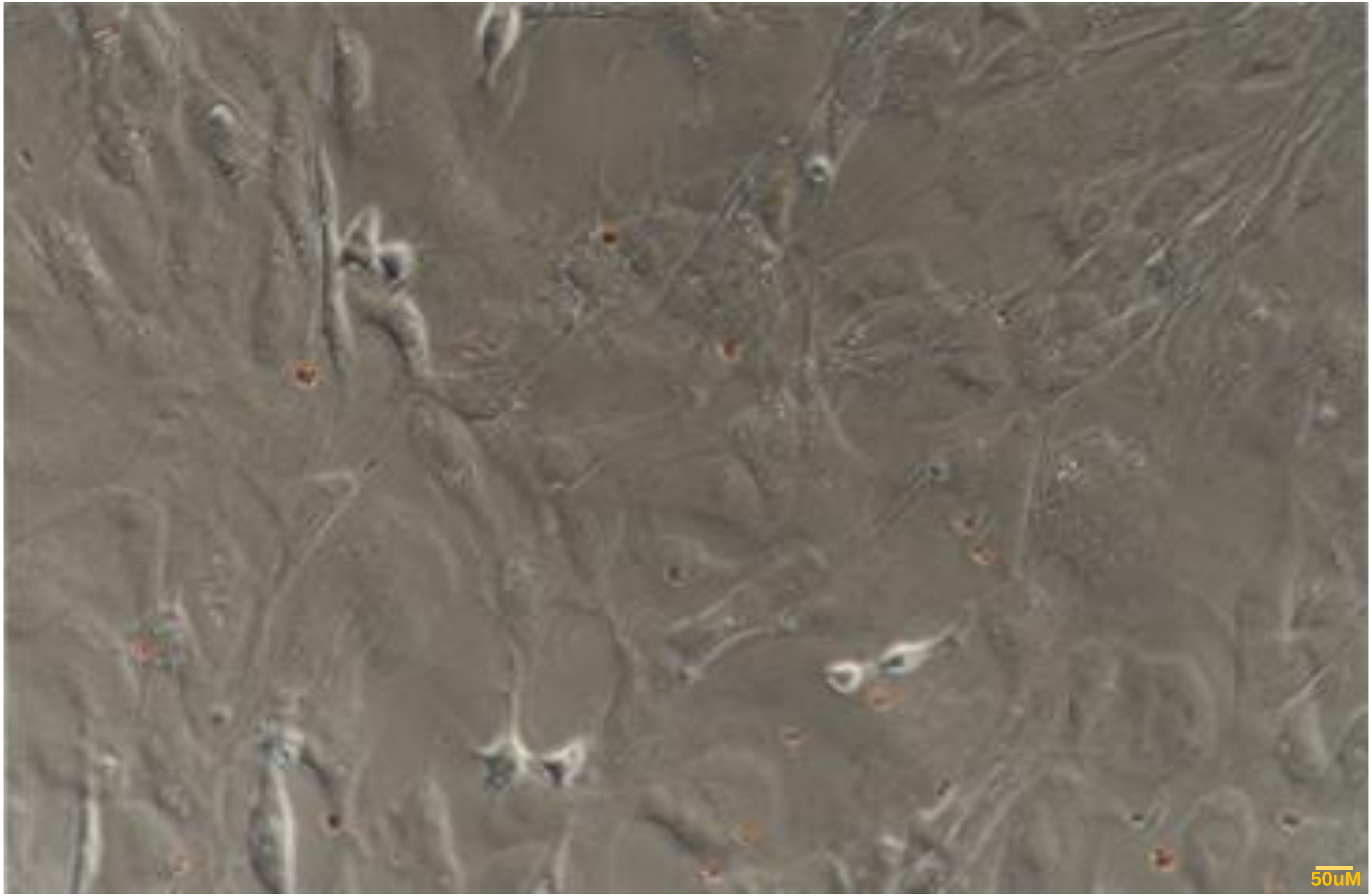

D-8 **AM + LiCl**

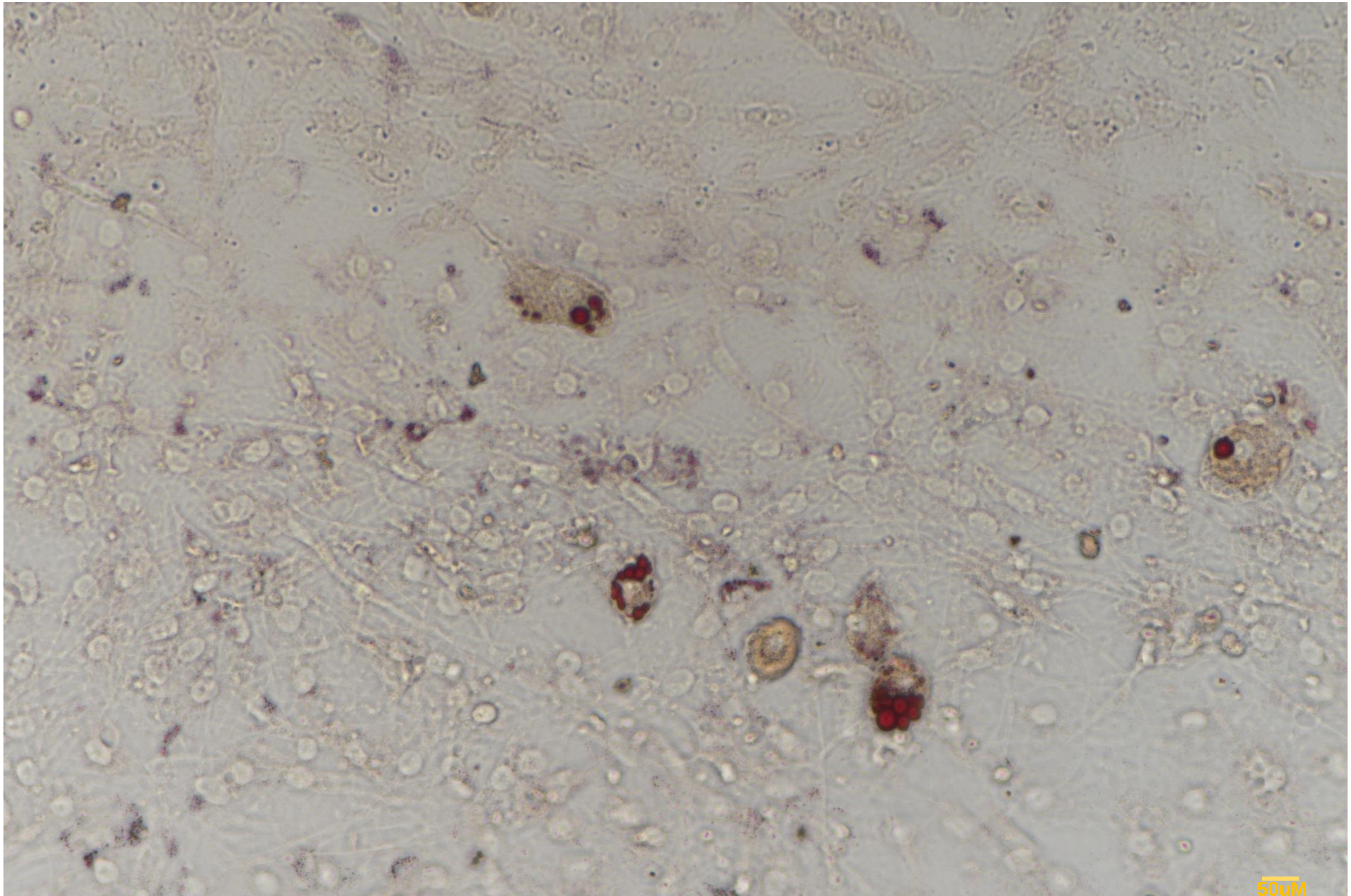

D-0 AM (LG)

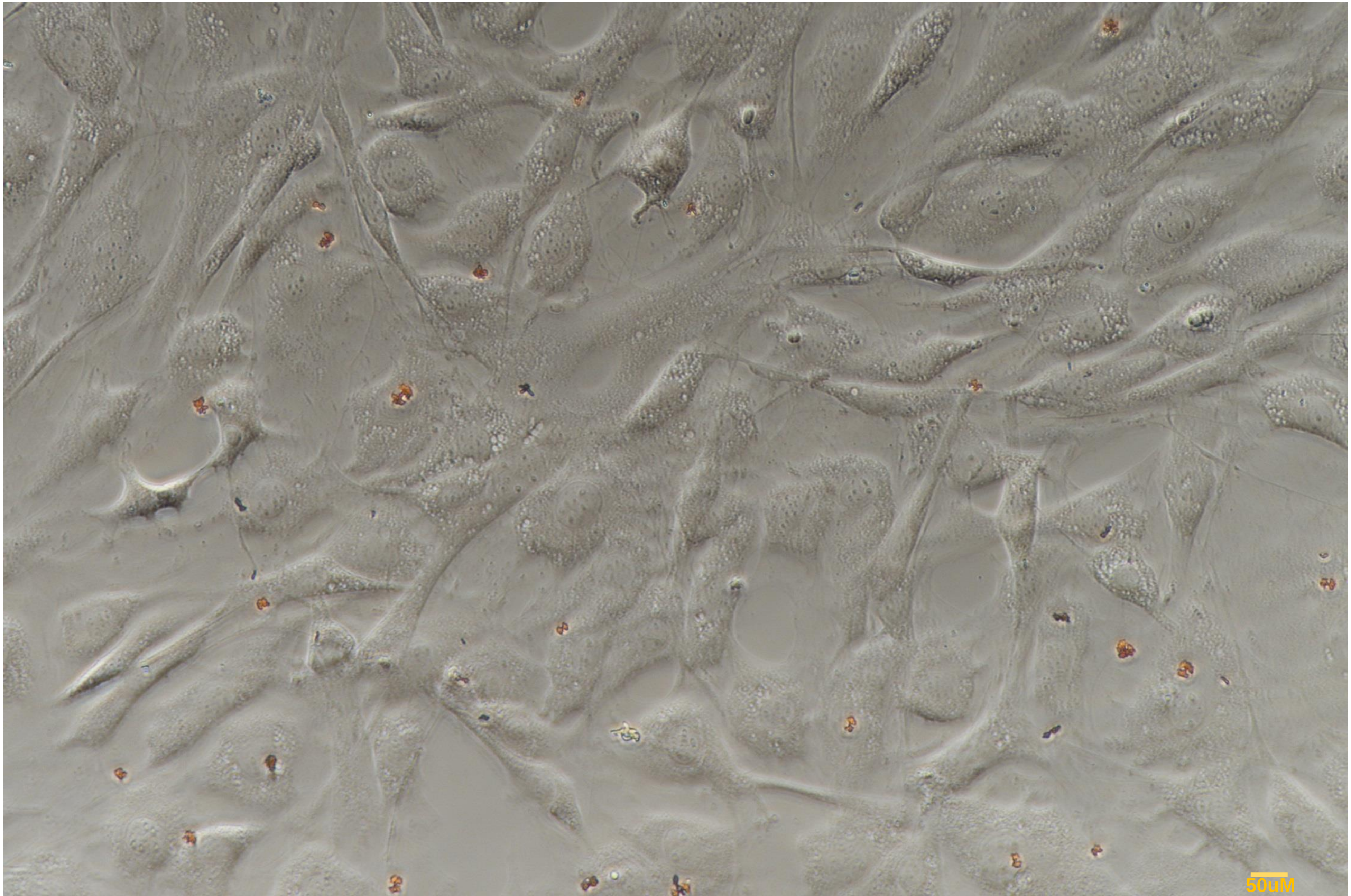

D-8 **AM (LG)**

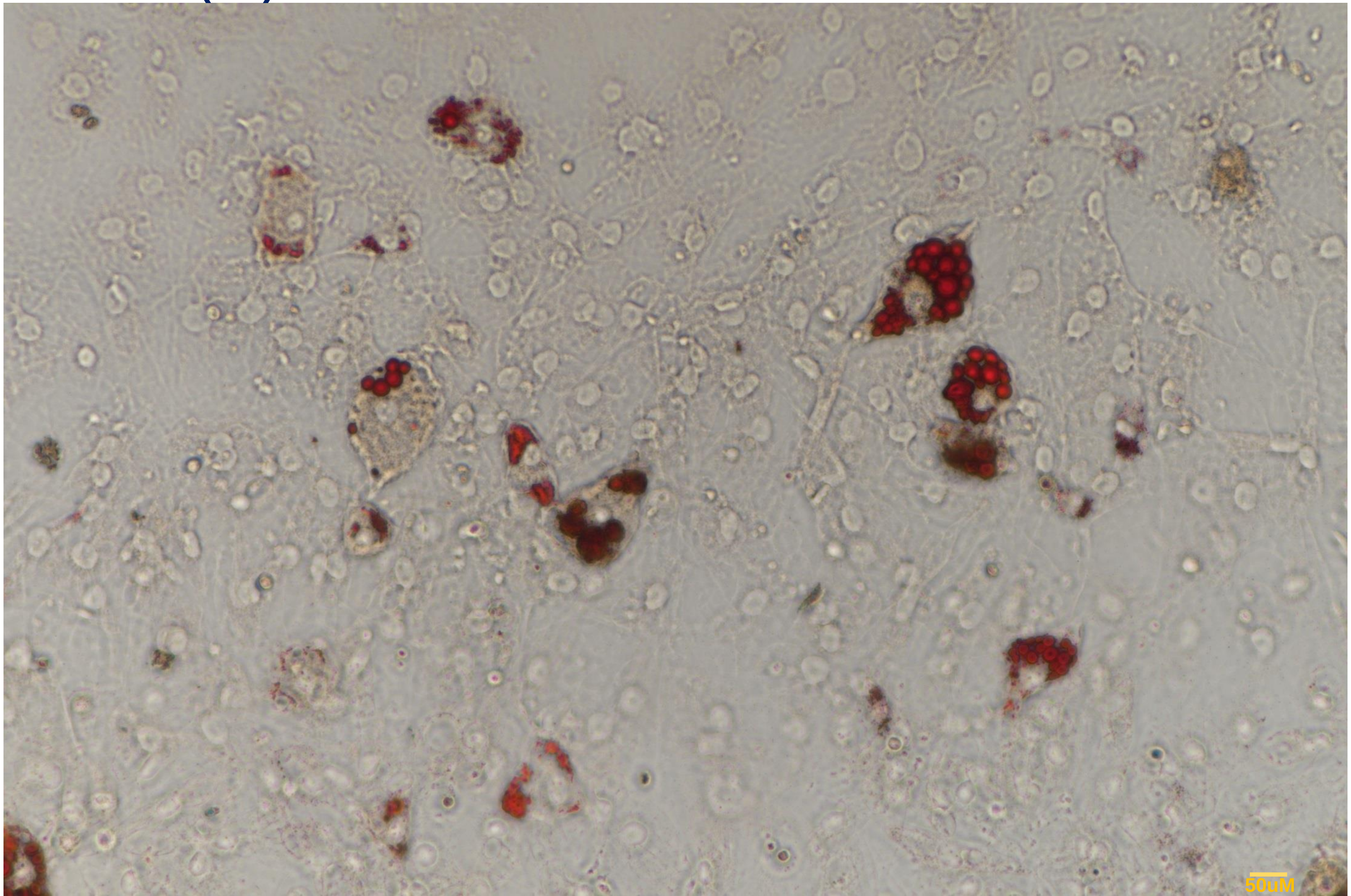

D-0 AM (HG)

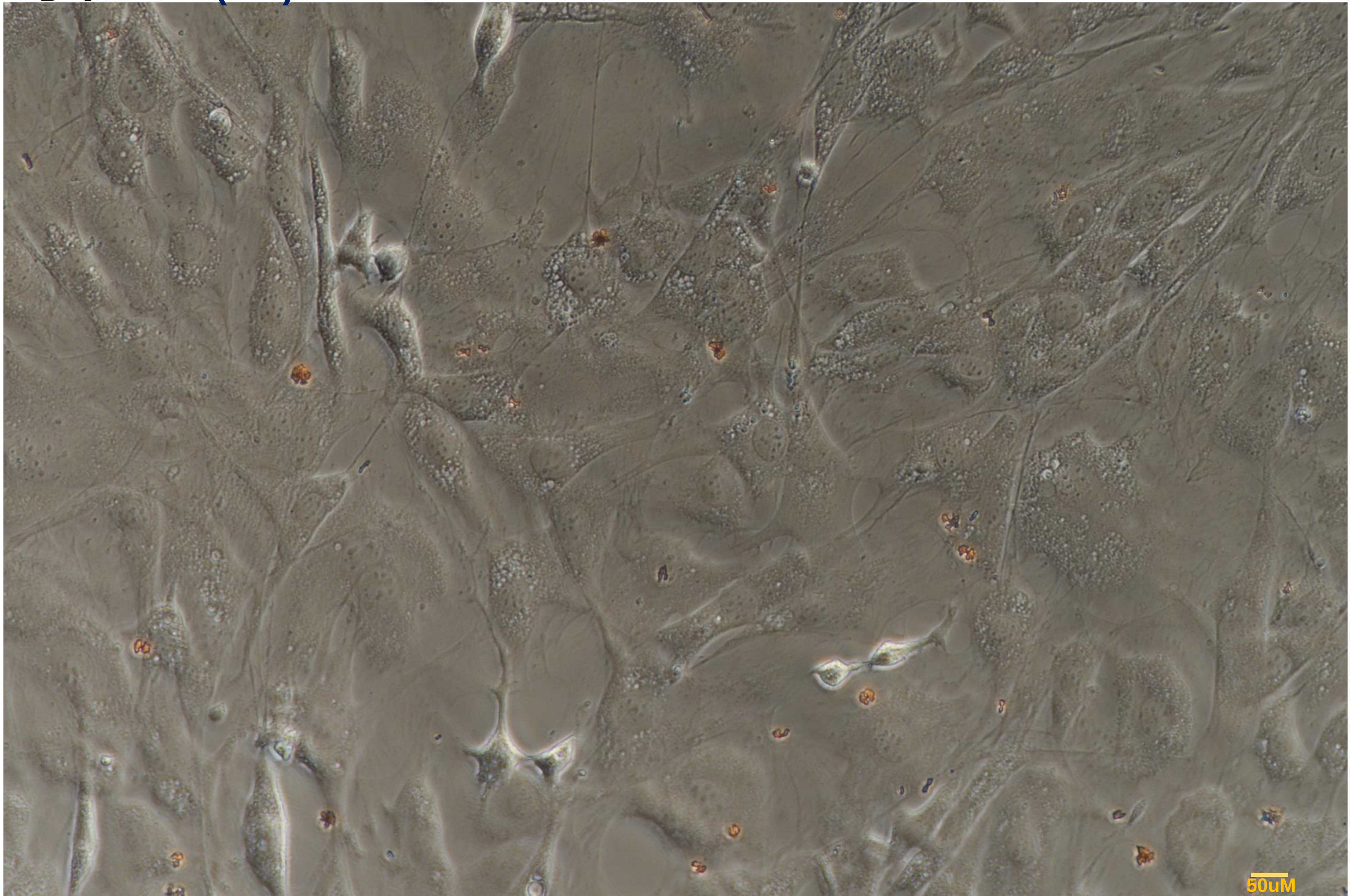

D-8 AM (HG)

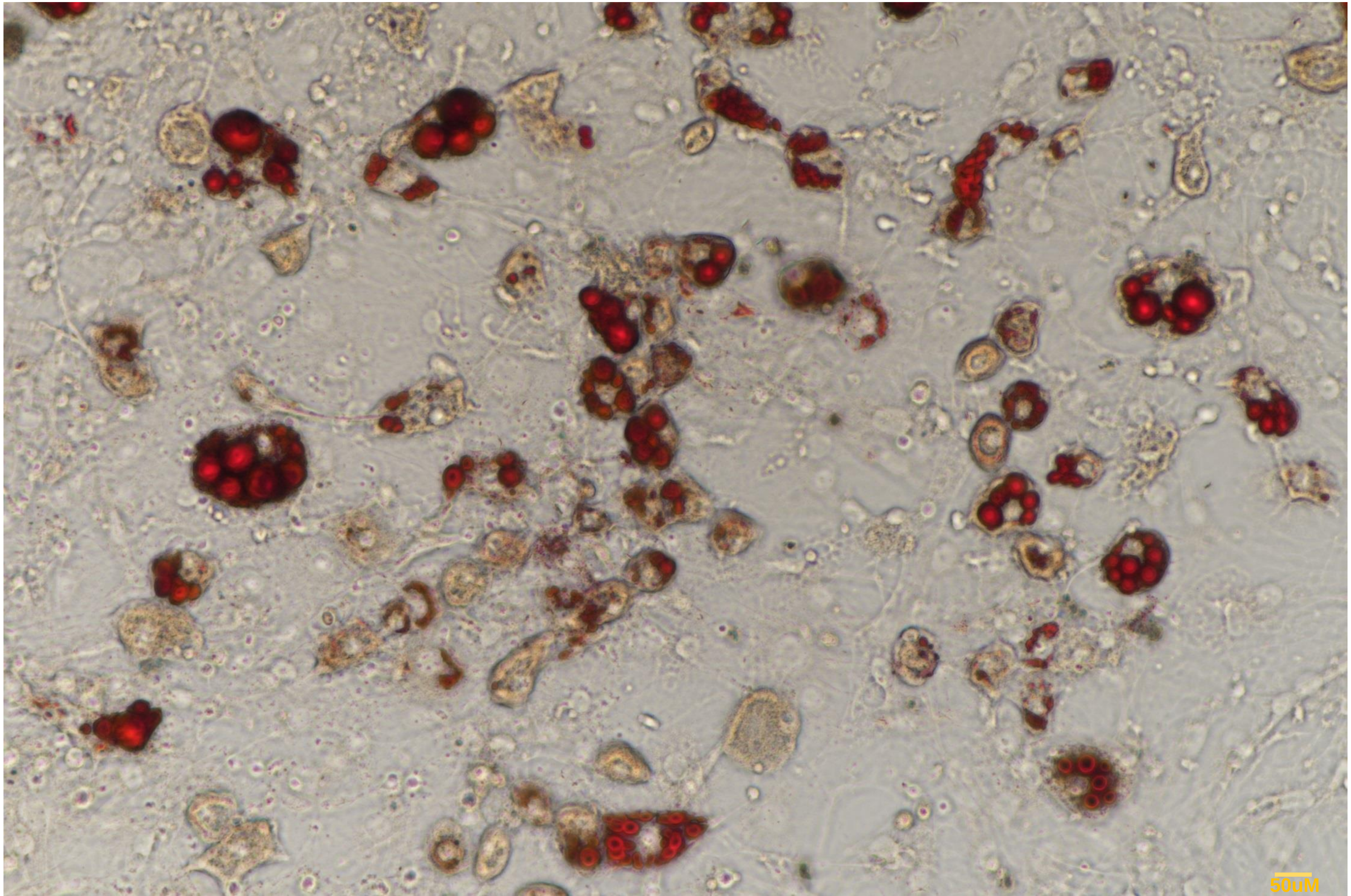

D-0 AM (HG + LiCl)

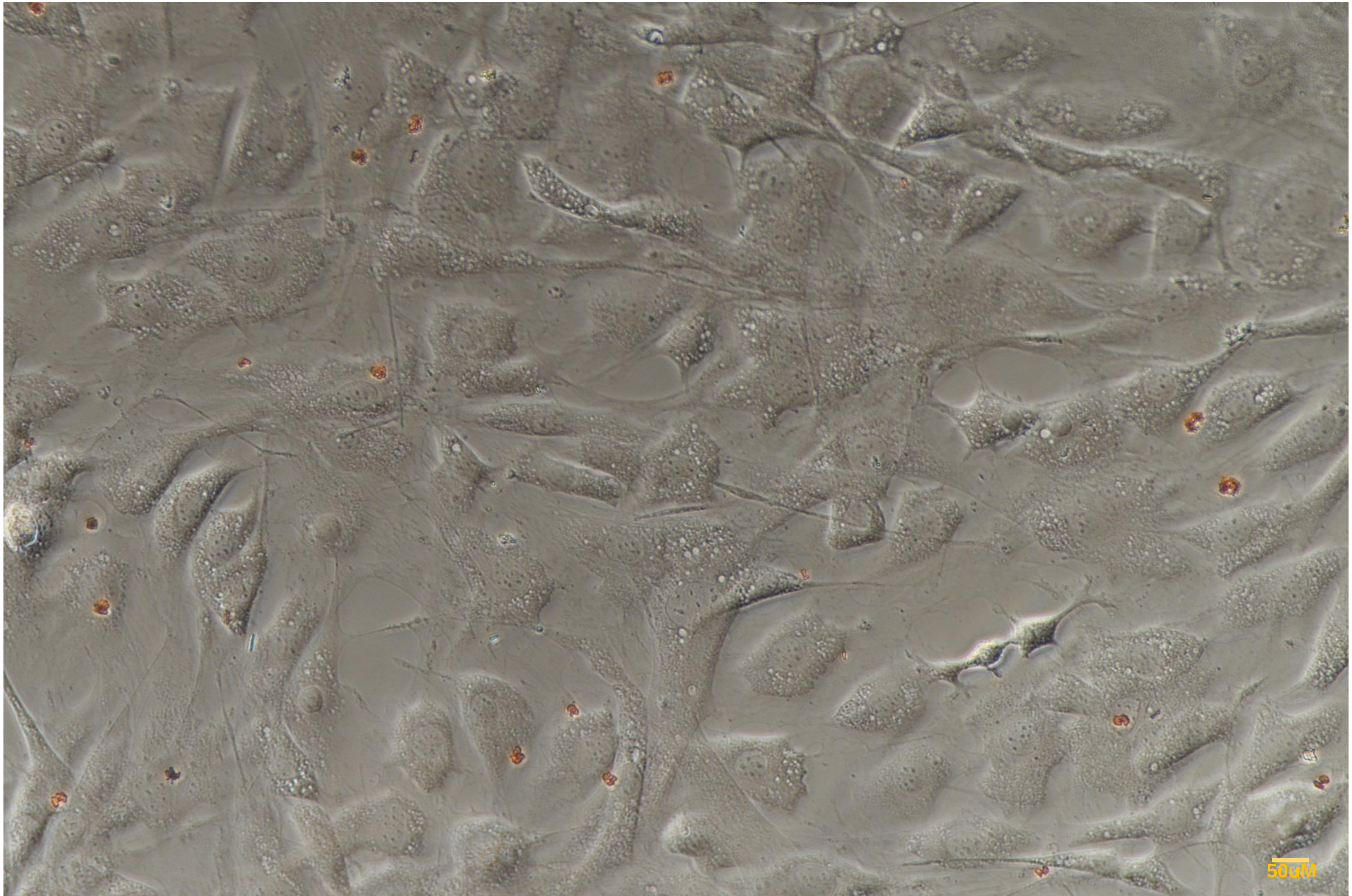

D-8 **AM (HG + LiCl)**

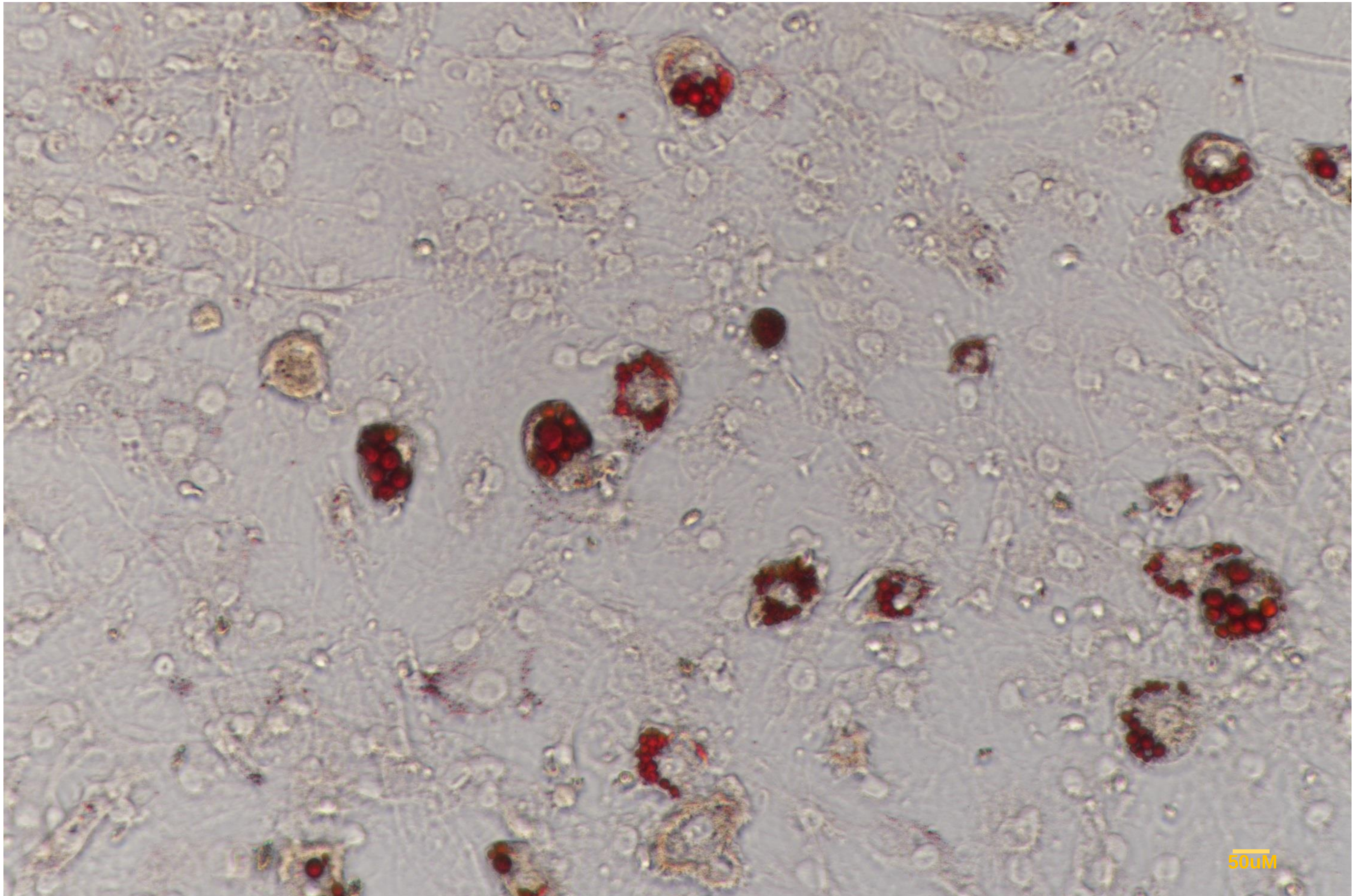

Fig.5 G)

AM RUNX2

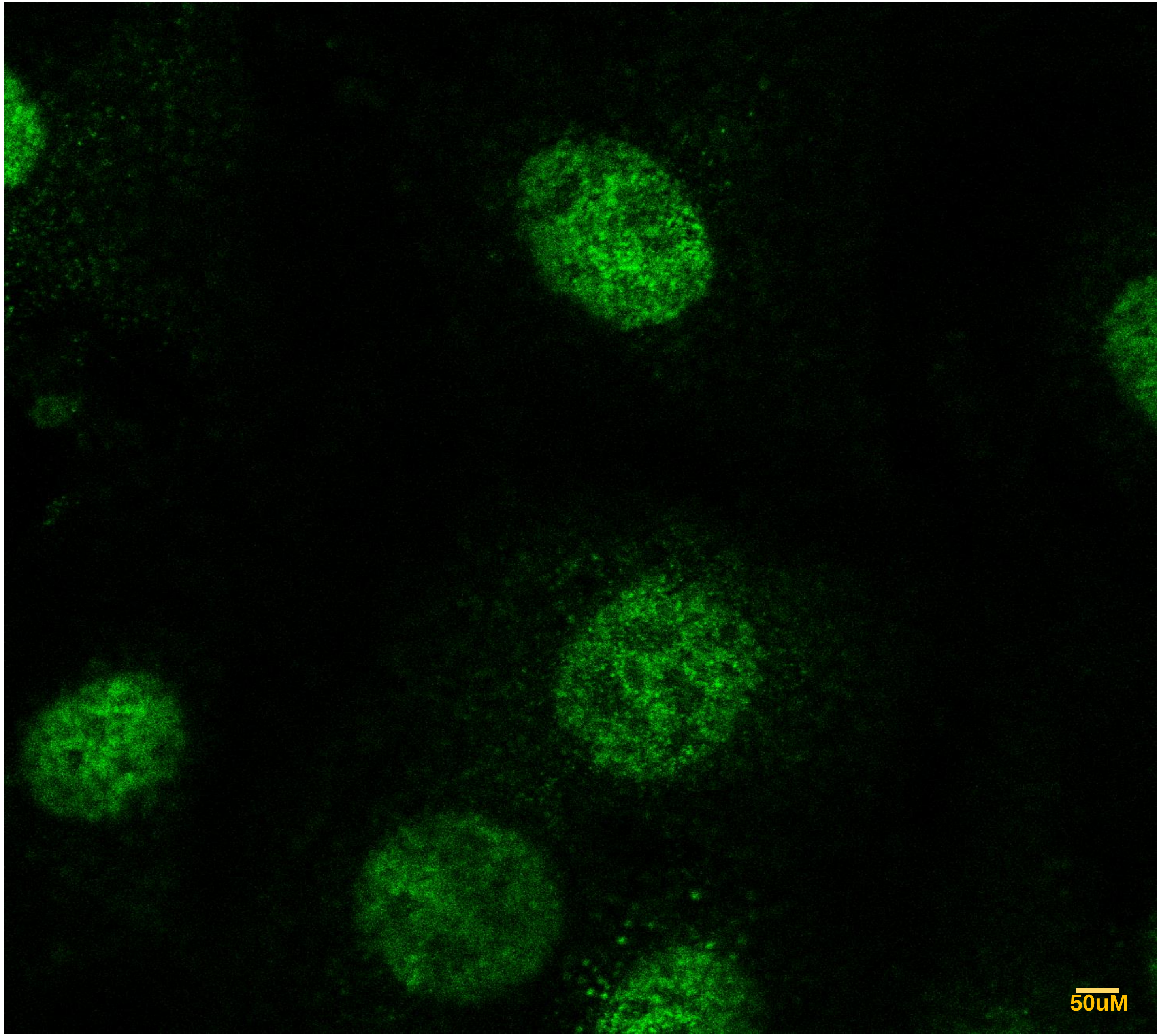

AM (HG) RUNX2

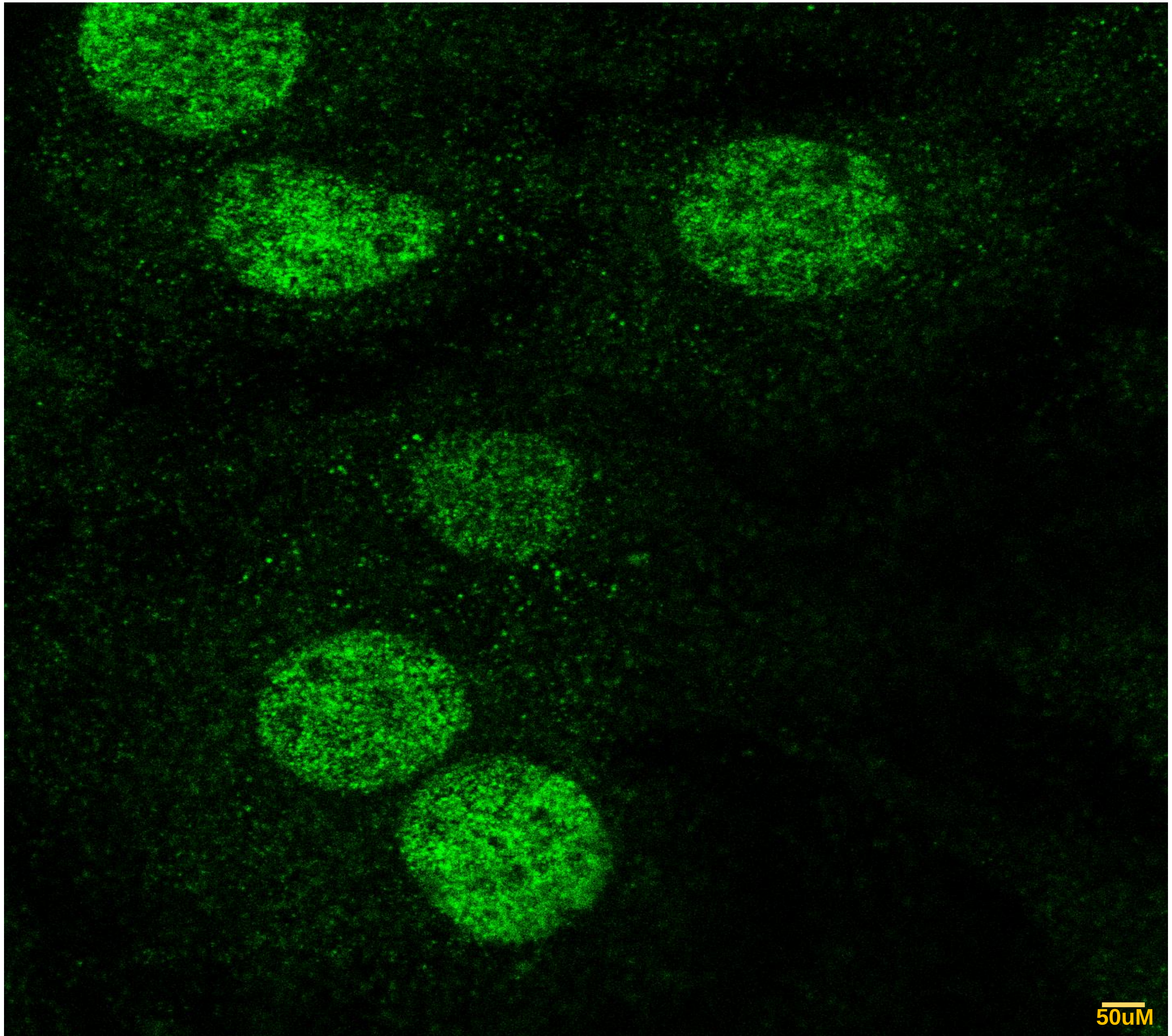

AM (LG) RUNX2

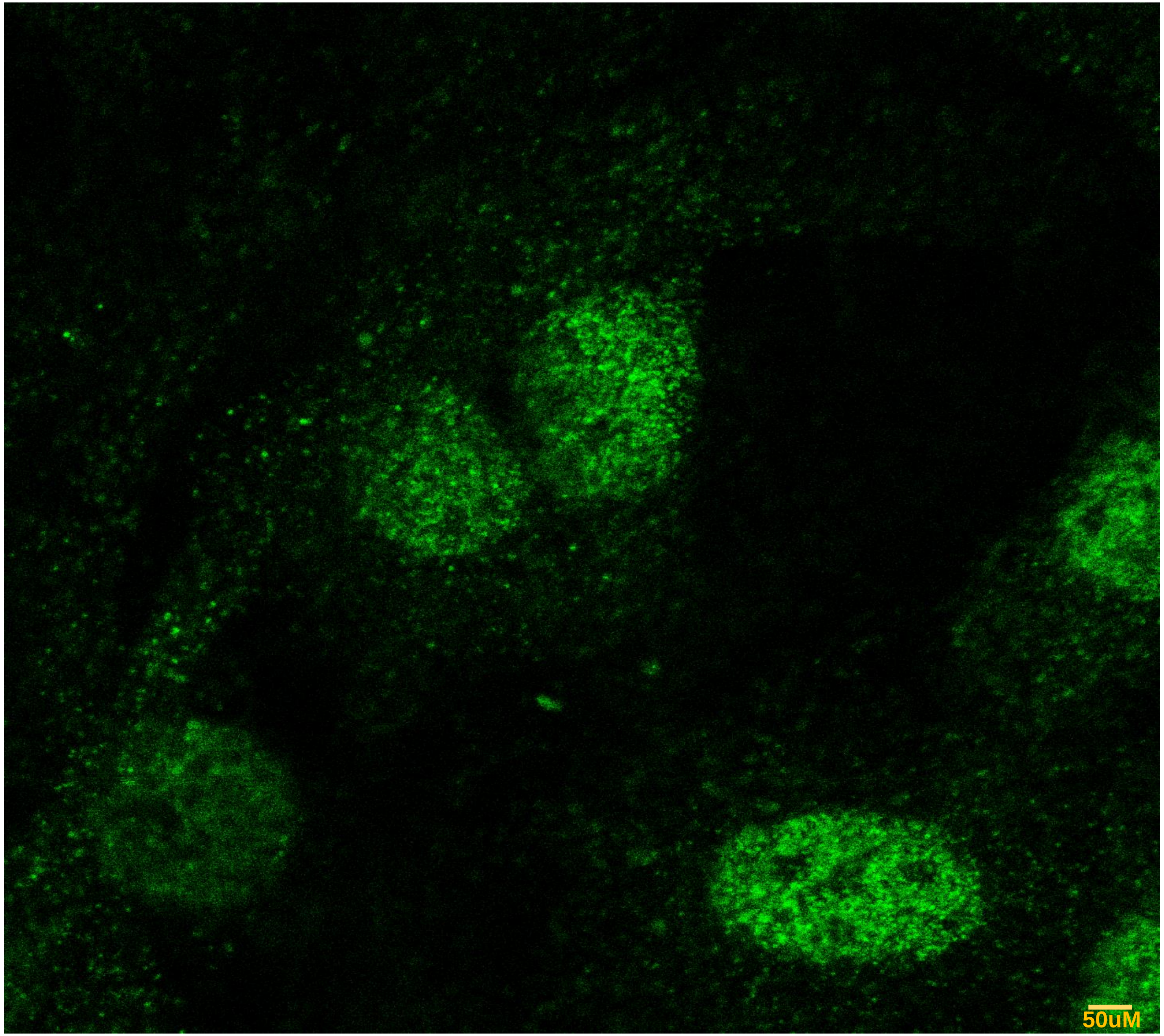

AM (HG, noGlut) RUNX2

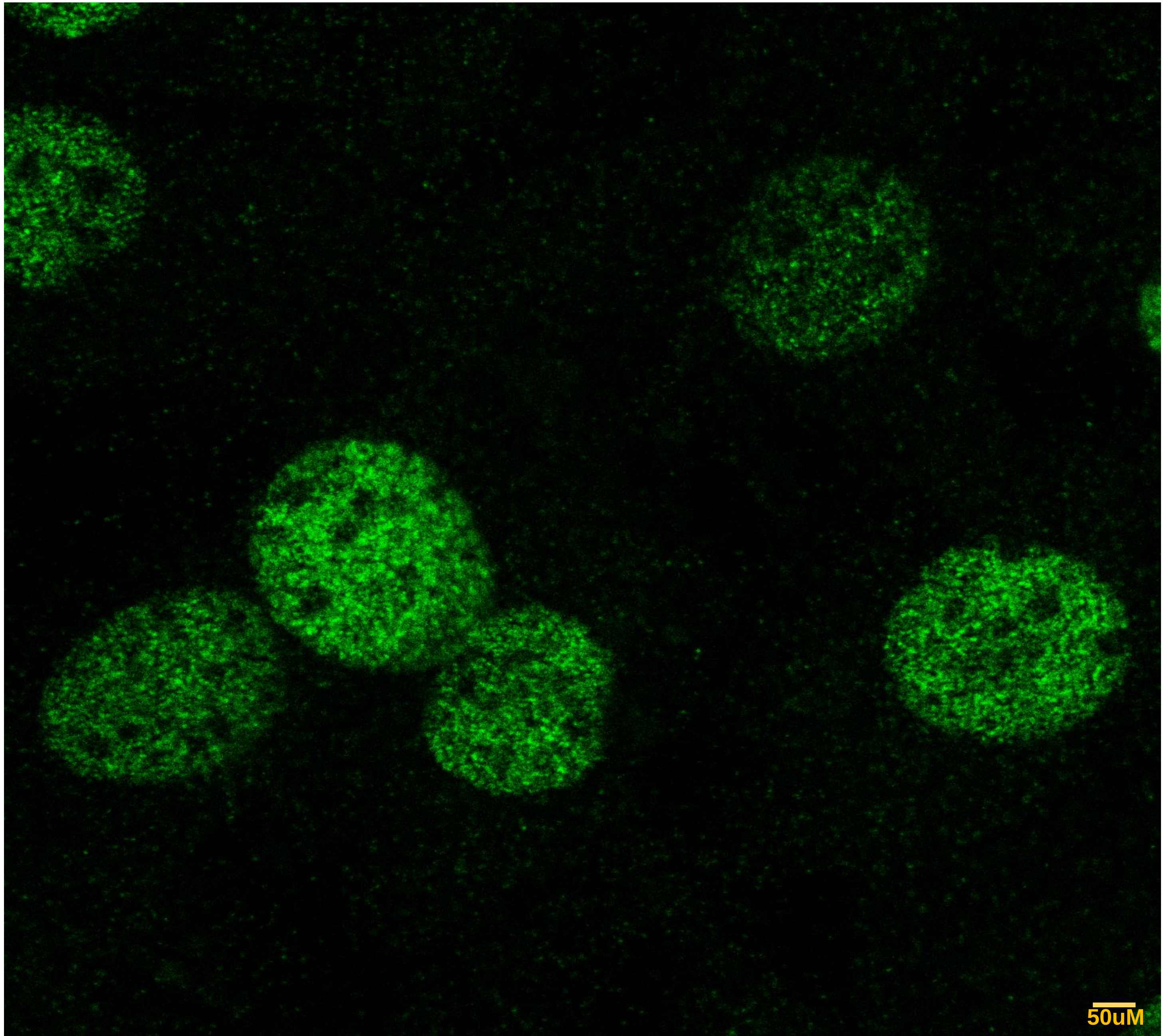

AM (LG, HGlut) RUNX2

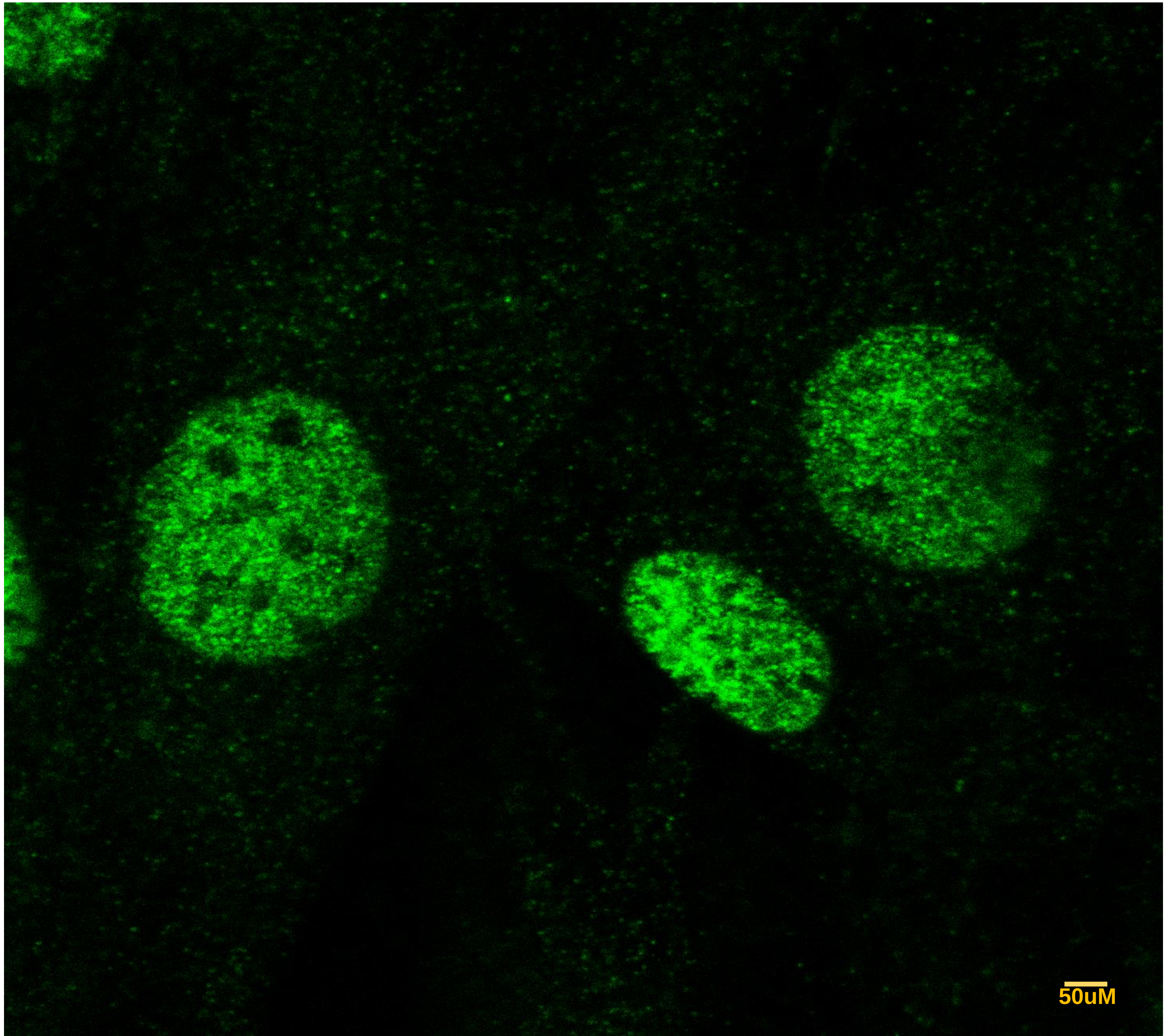

AM GSK3β

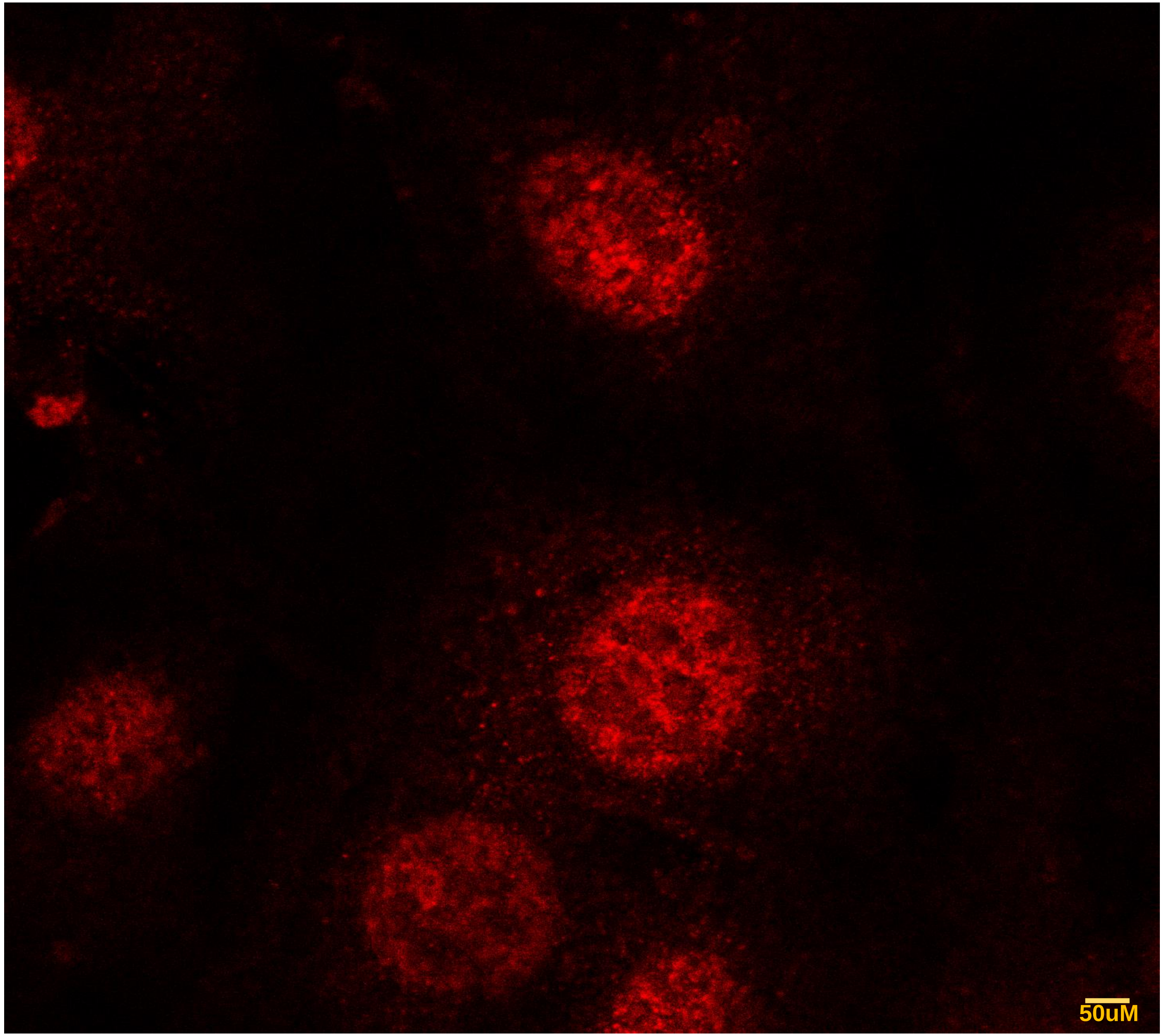

AM (HG) GSK3β

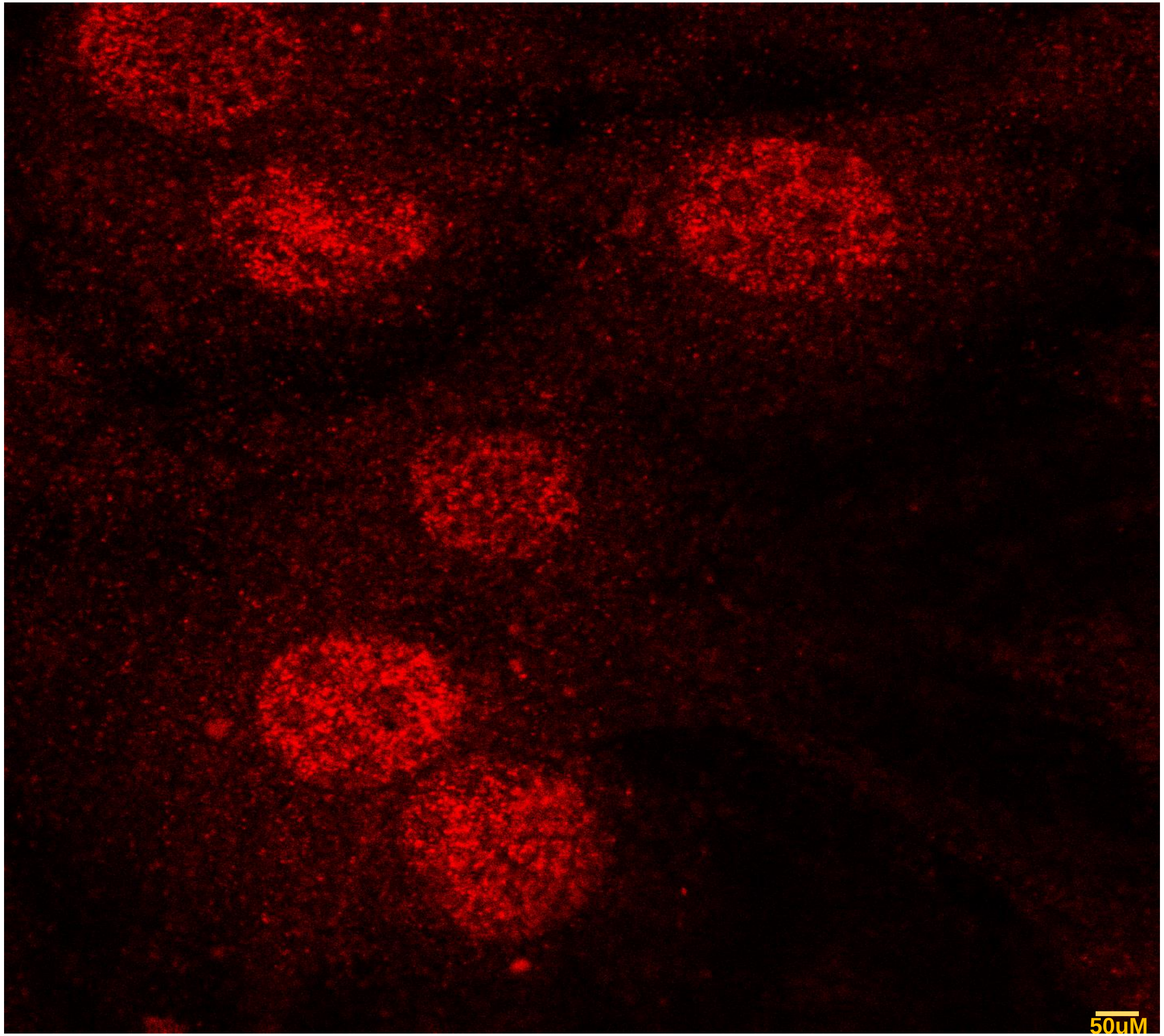

AM (LG)

GSK3β

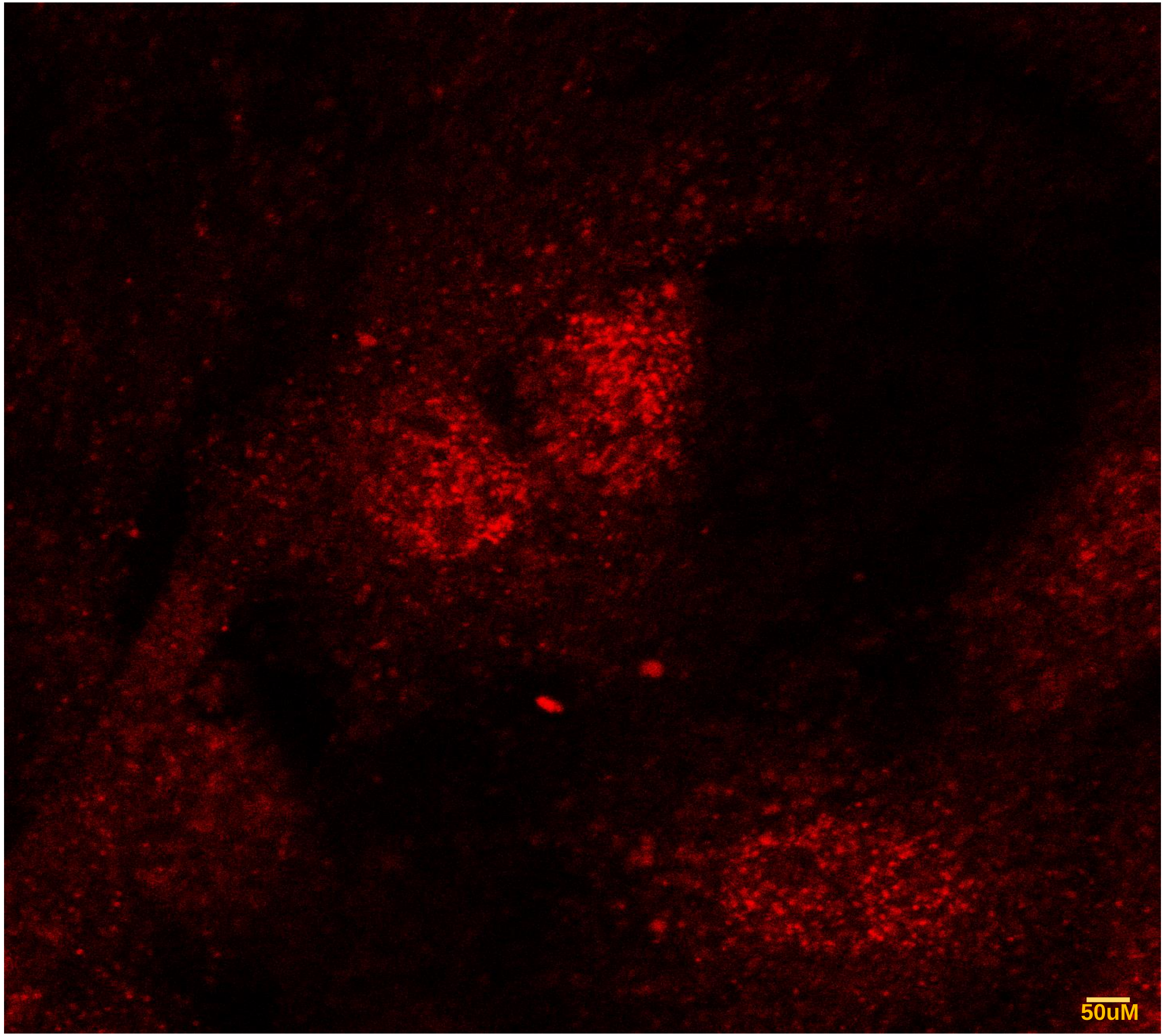

AM (HG, noGlut)

GSK3β

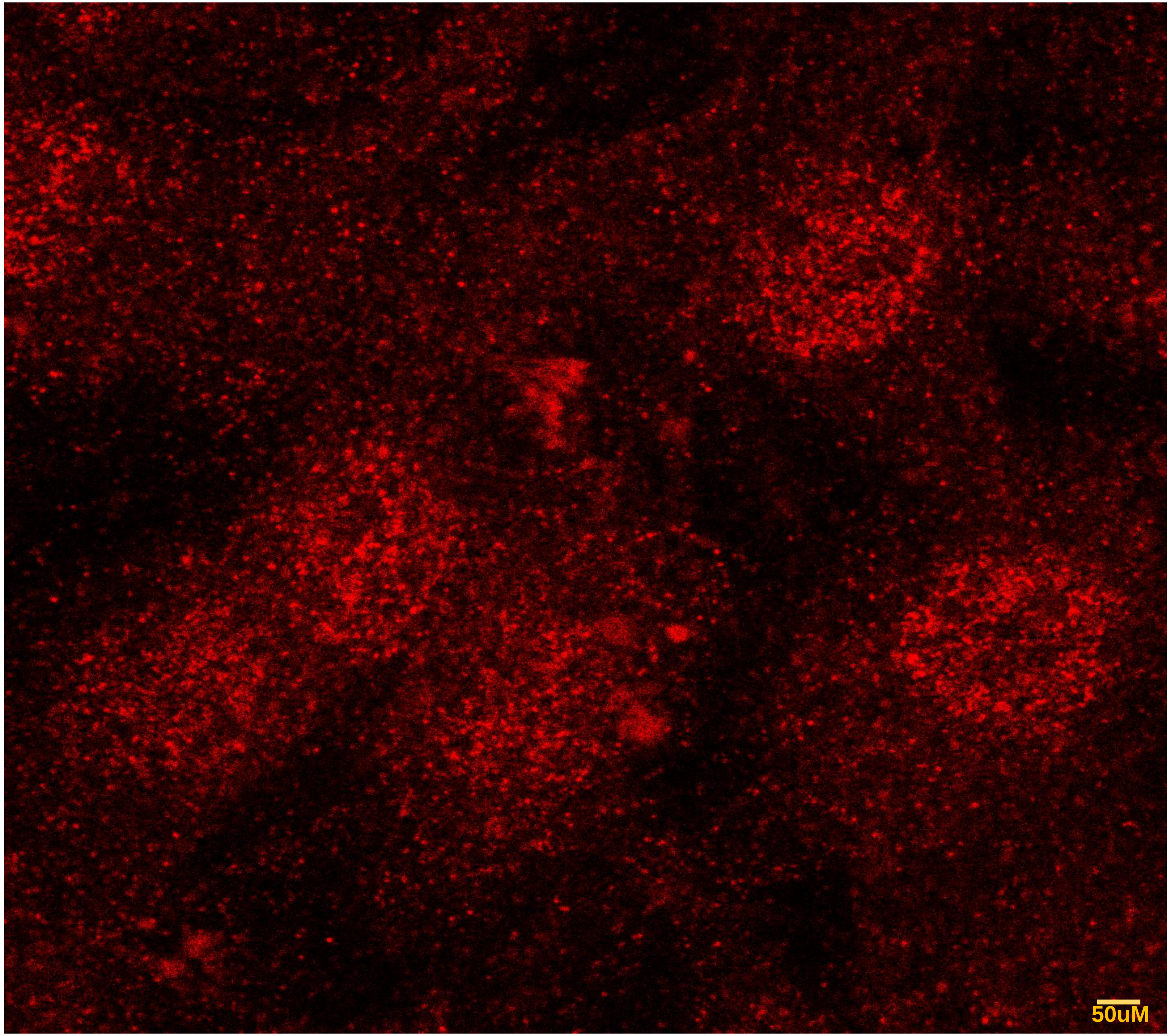

AM (LG, HGlut) GSK3β

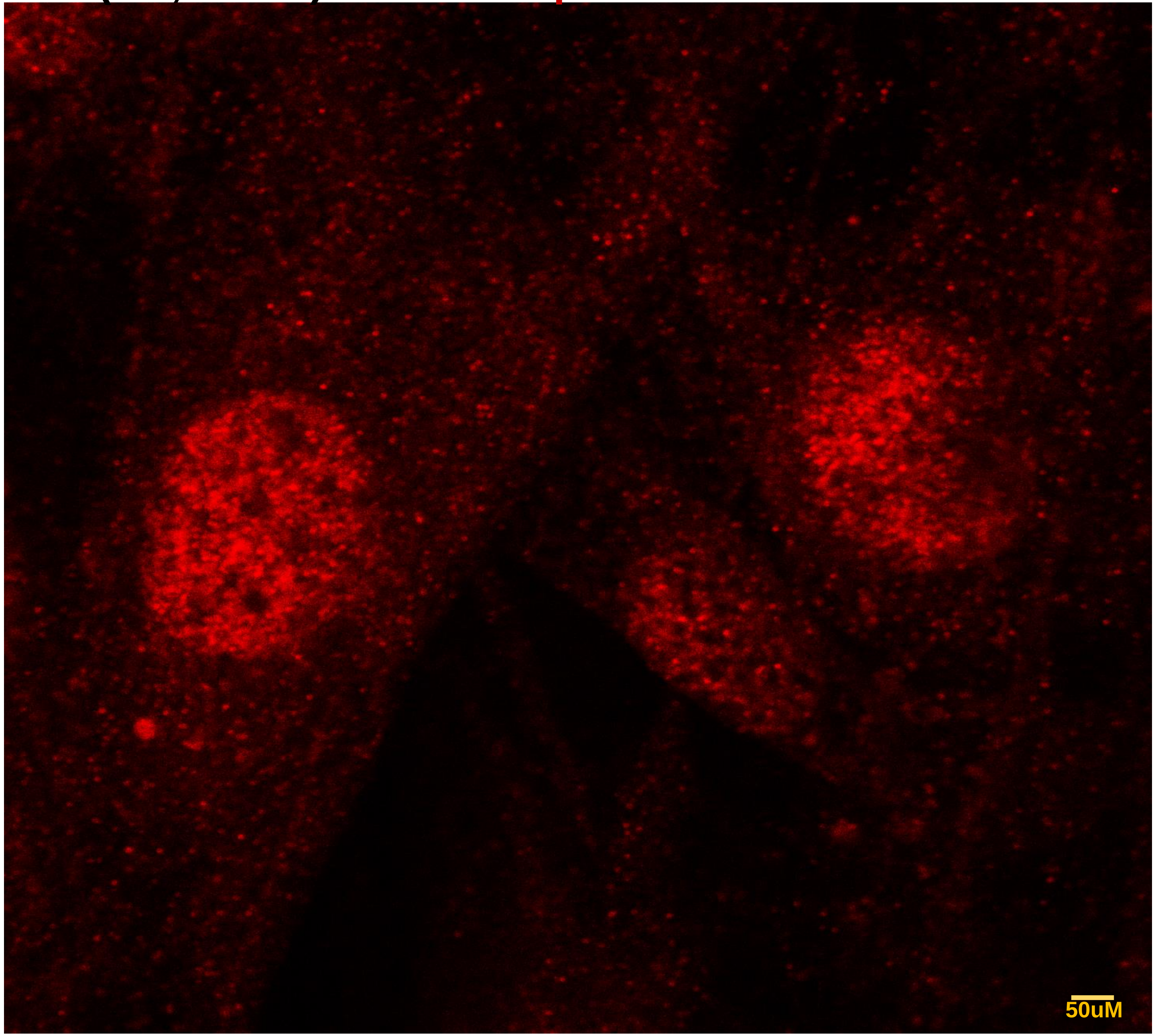

AM MERGED

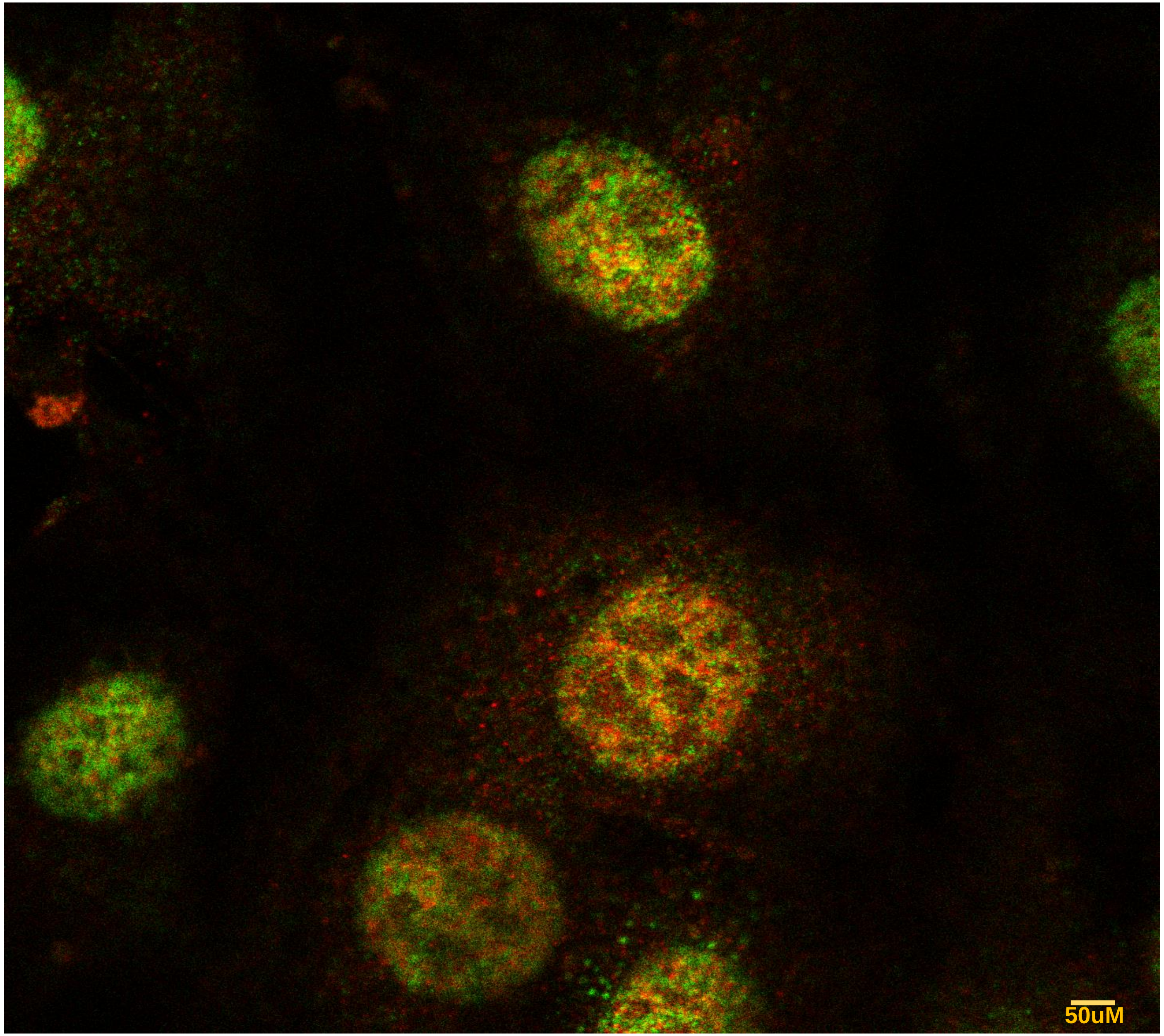

AM (HG) MERGED

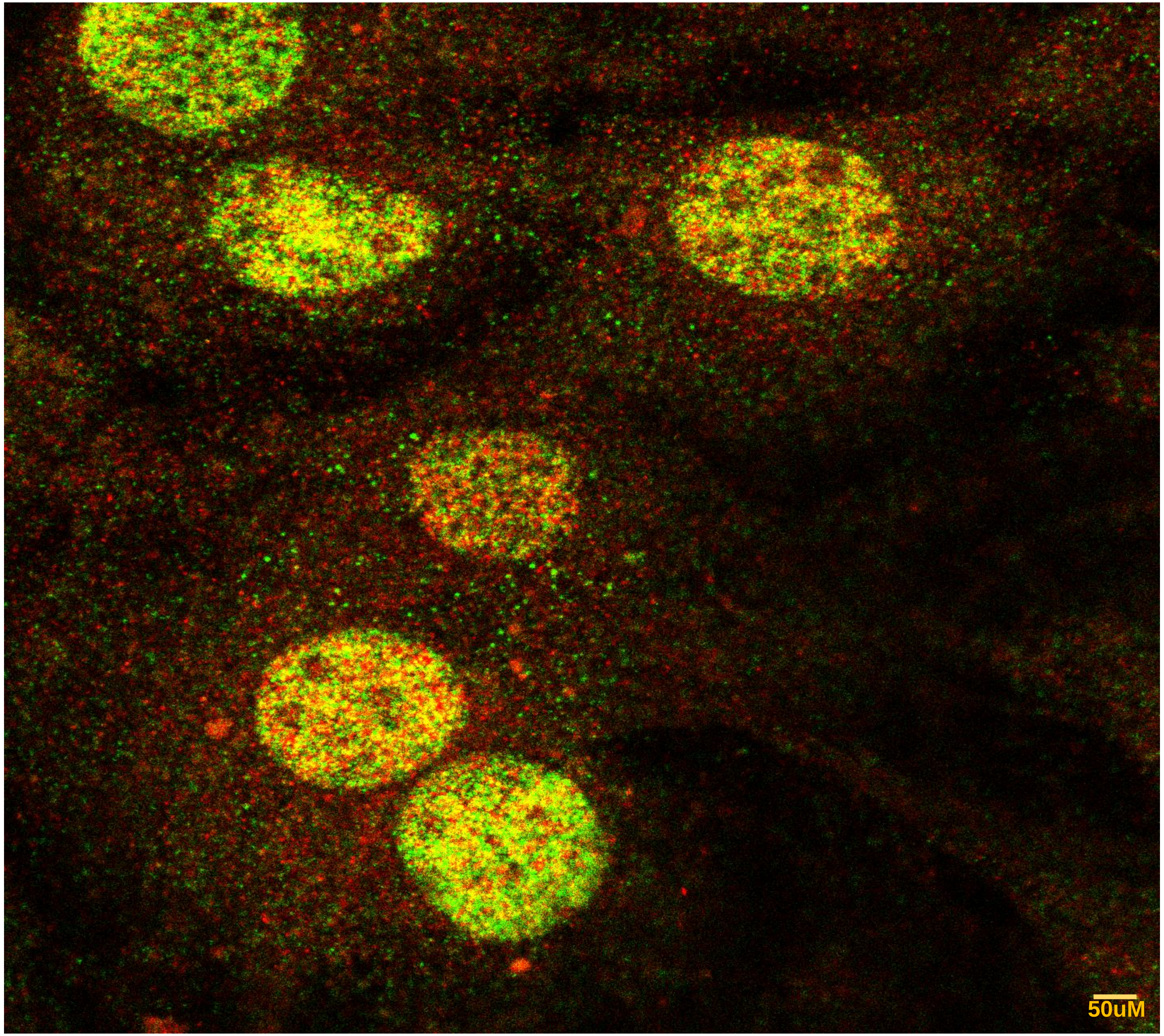

AM (LG) MERGED

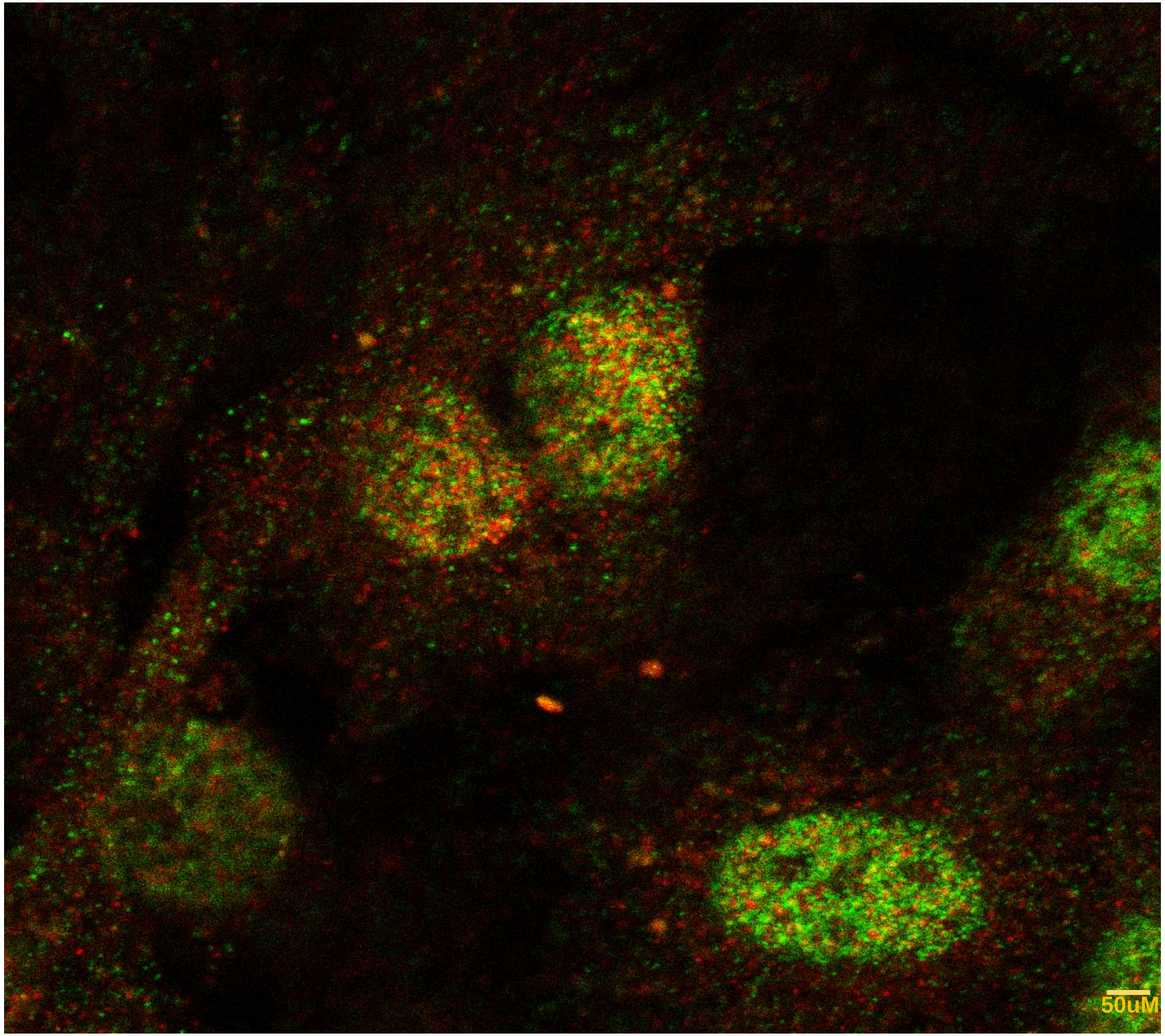

AM (HG, noGlut) MERGED

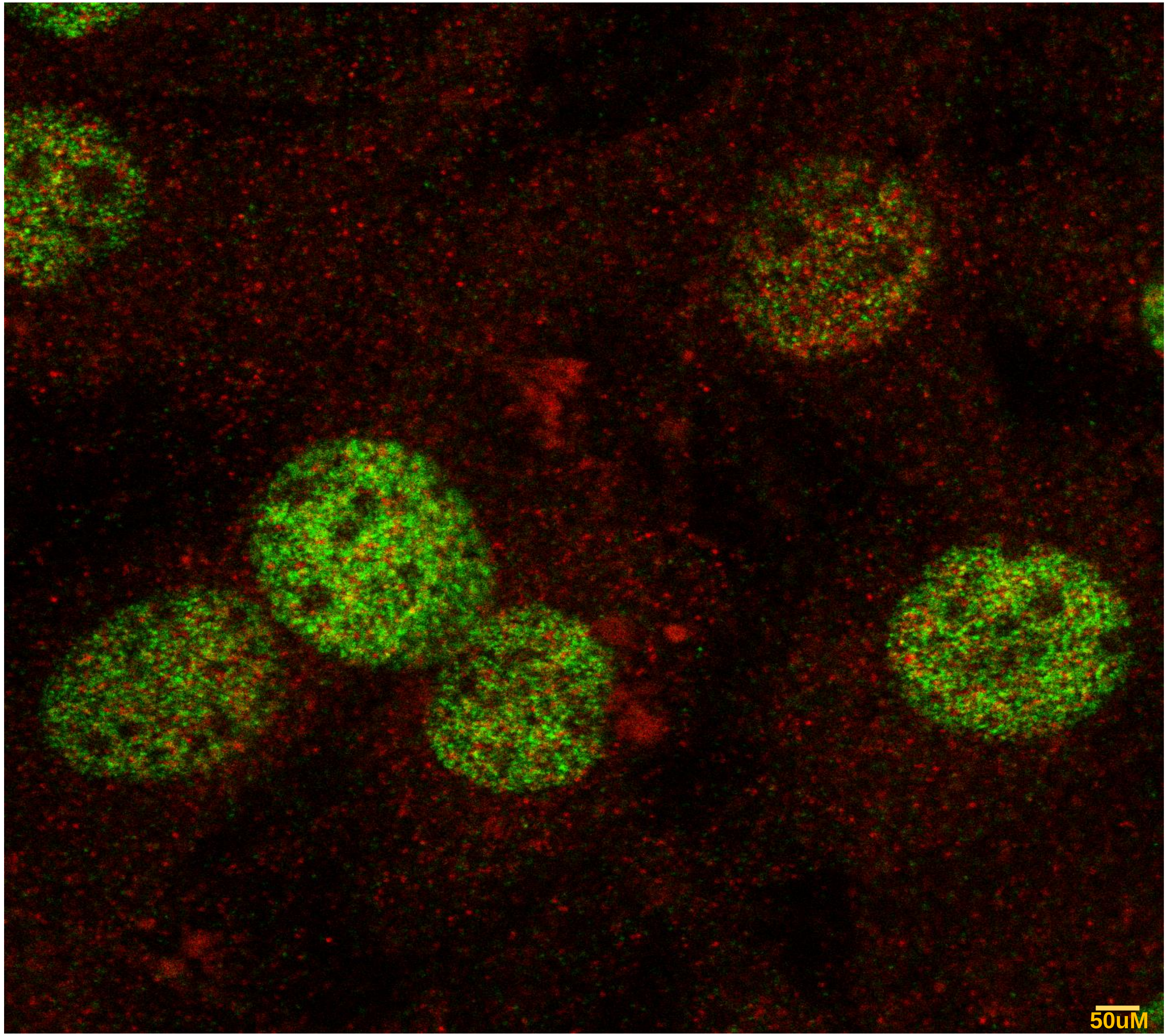

AM (LG, HGlut) MERGED

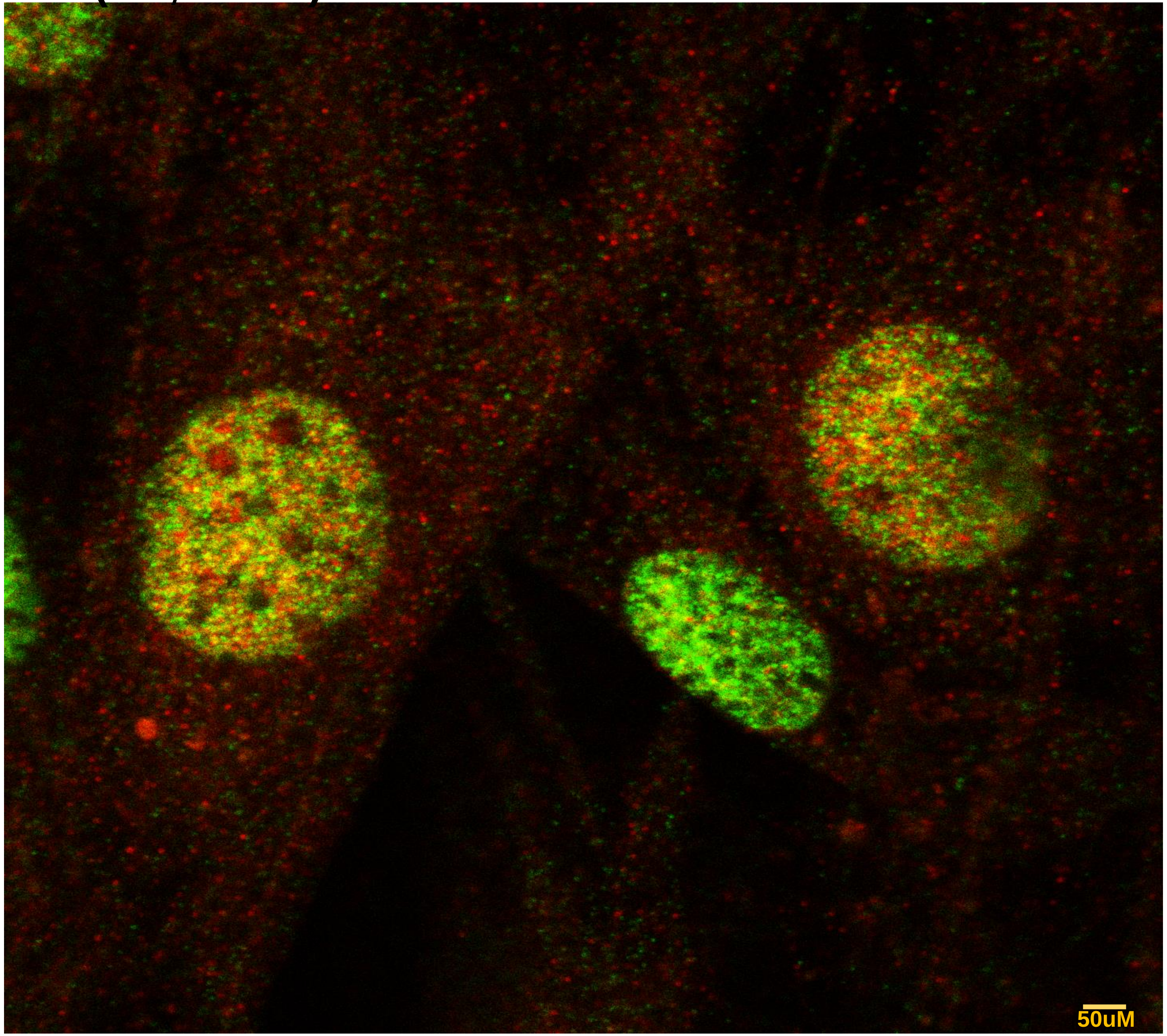

AM

MERGED + DAPI

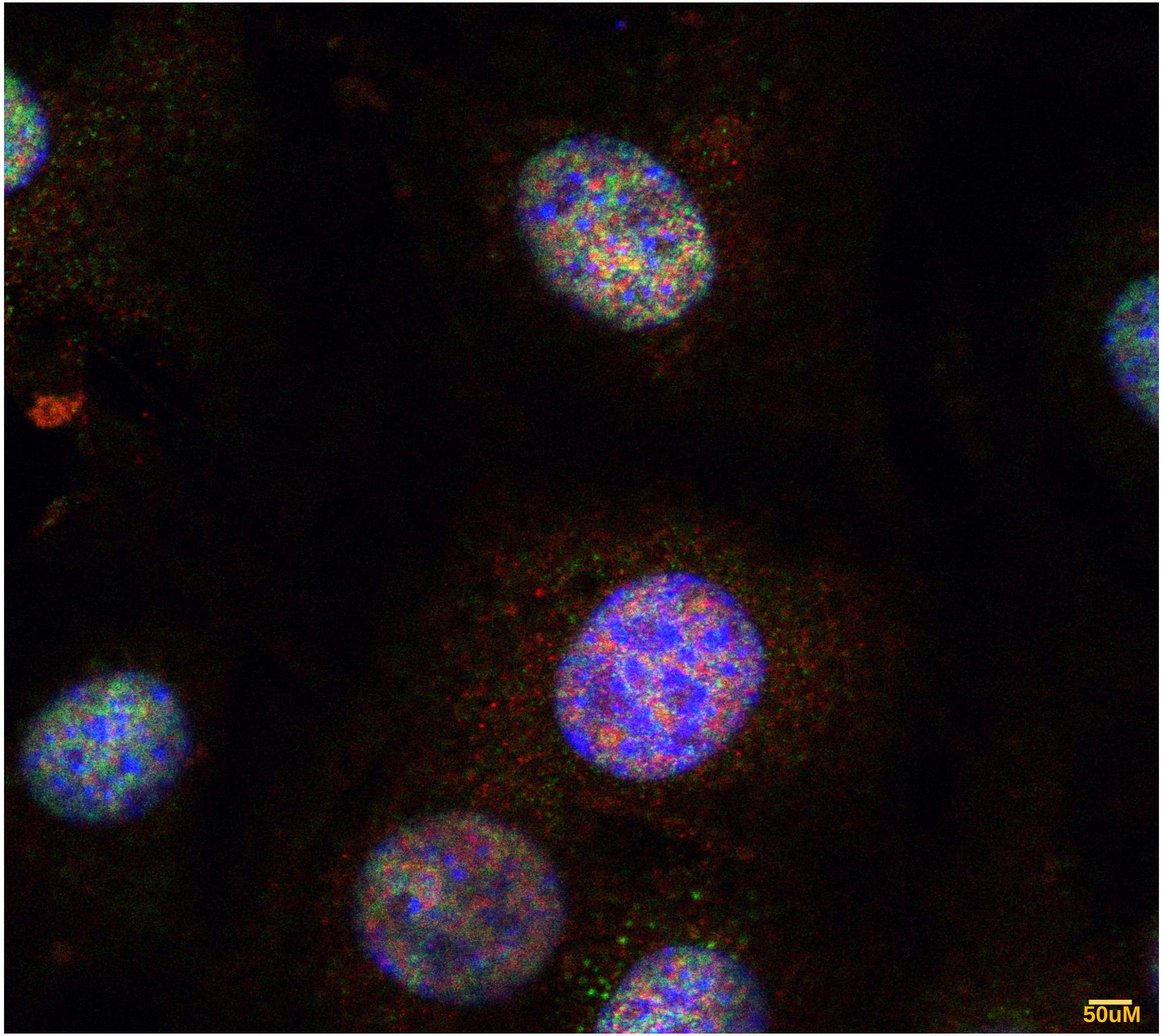

AM (HG)

MERGED + DAPI

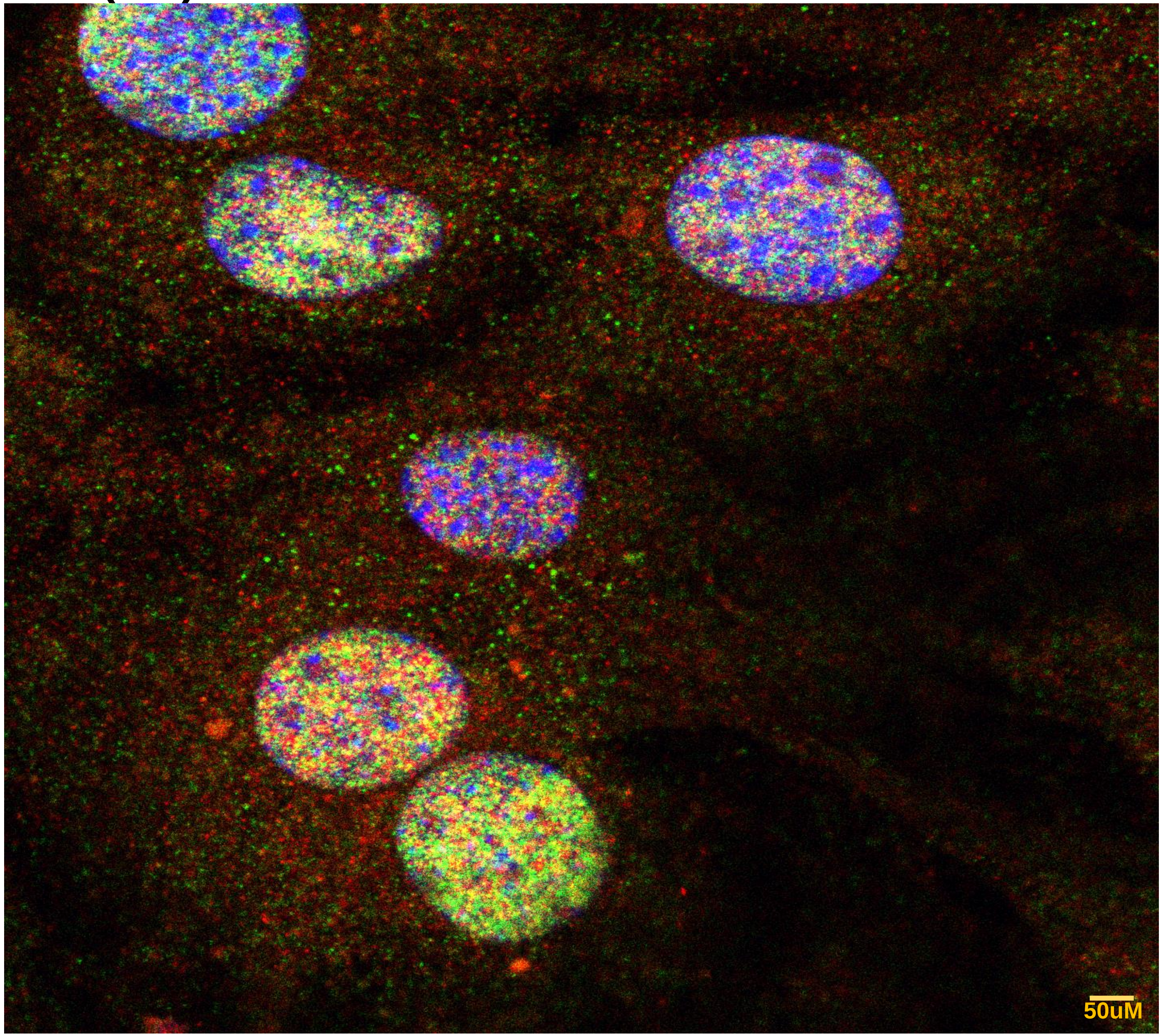

AM (LG)    MERGED + DAPI

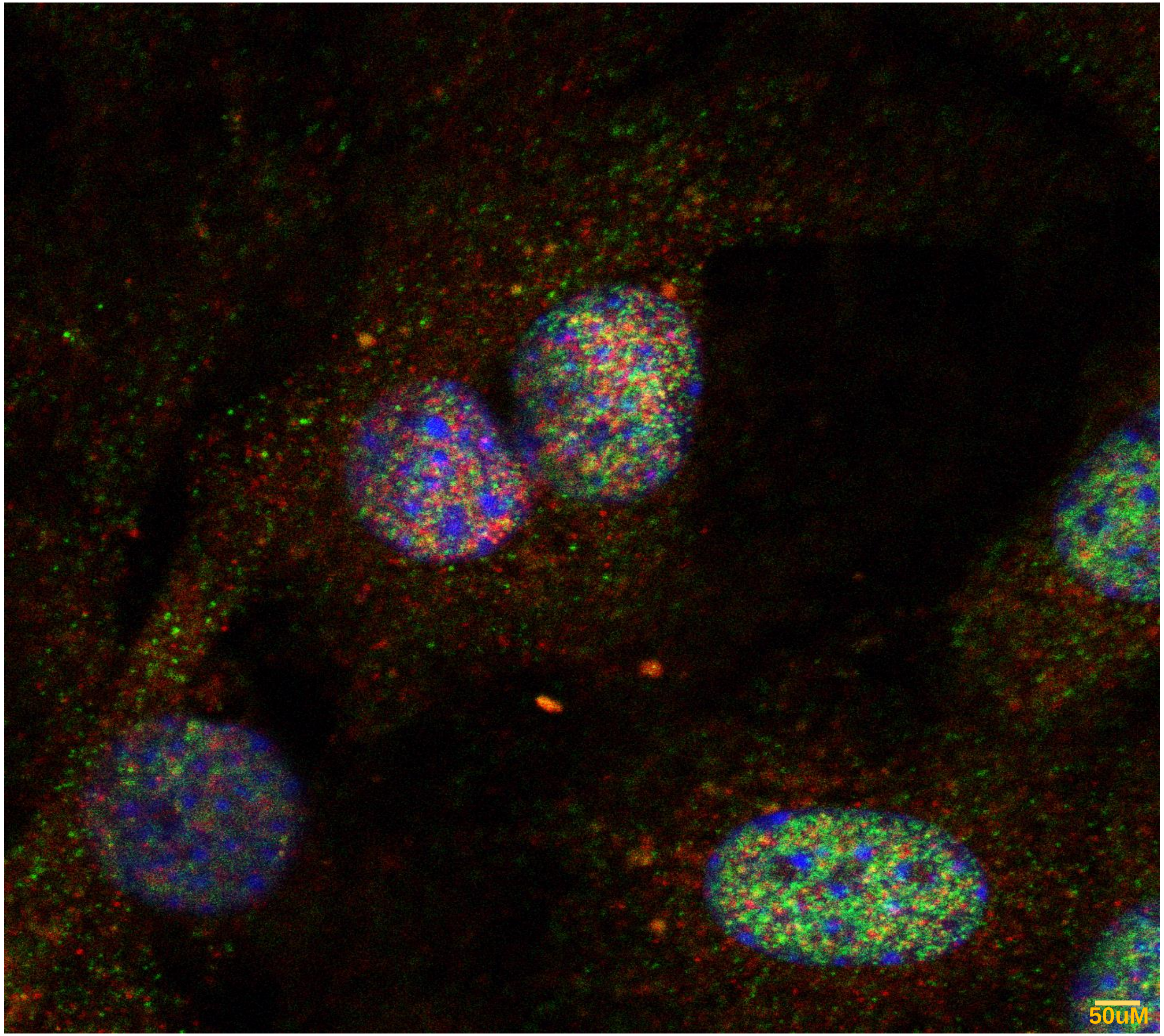

AM (HG, noGlut) MERGED + DAPI

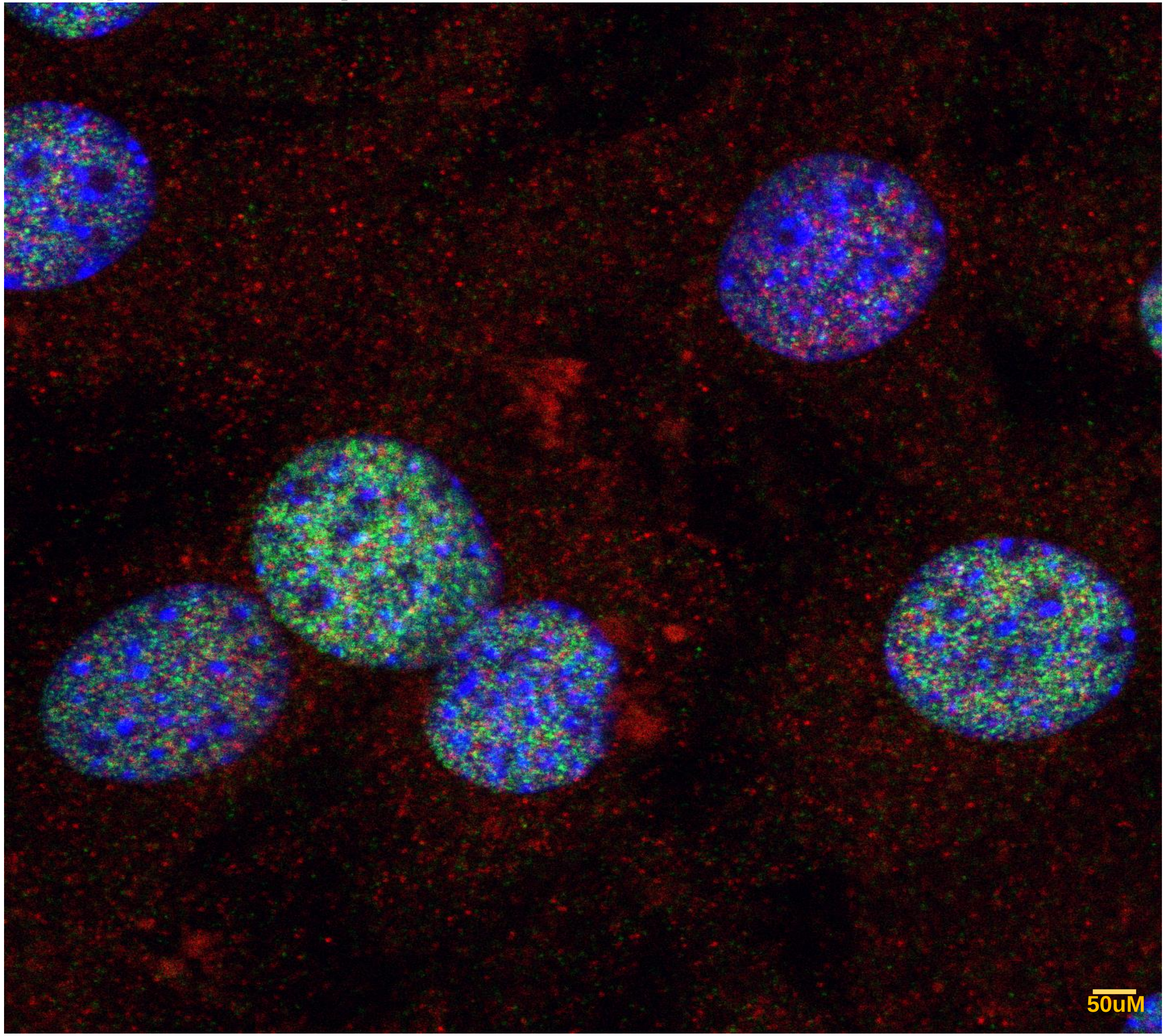

AM (LG, HGlut) MERGED + DAPI

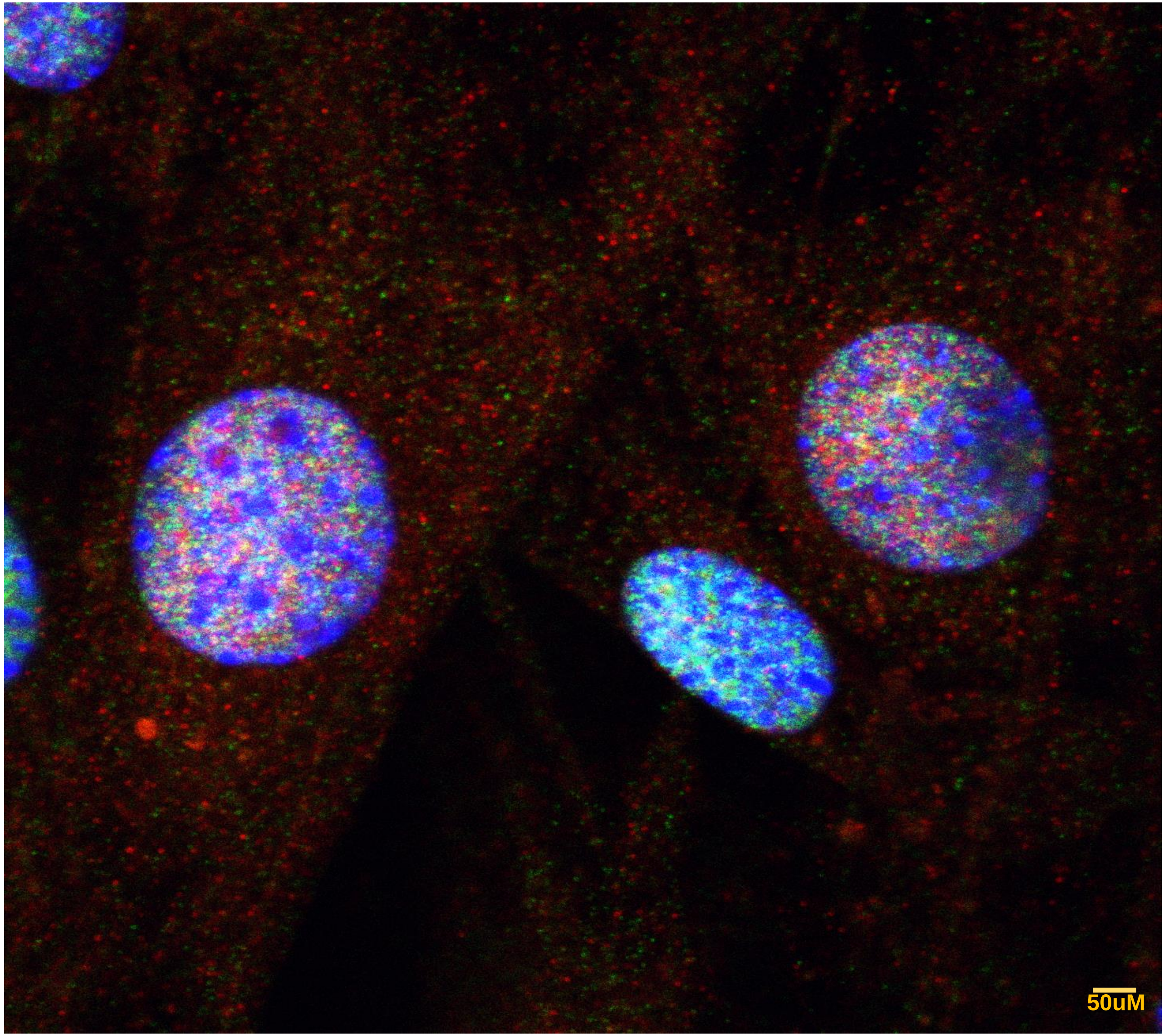

SF.2 B)

D-0 AM

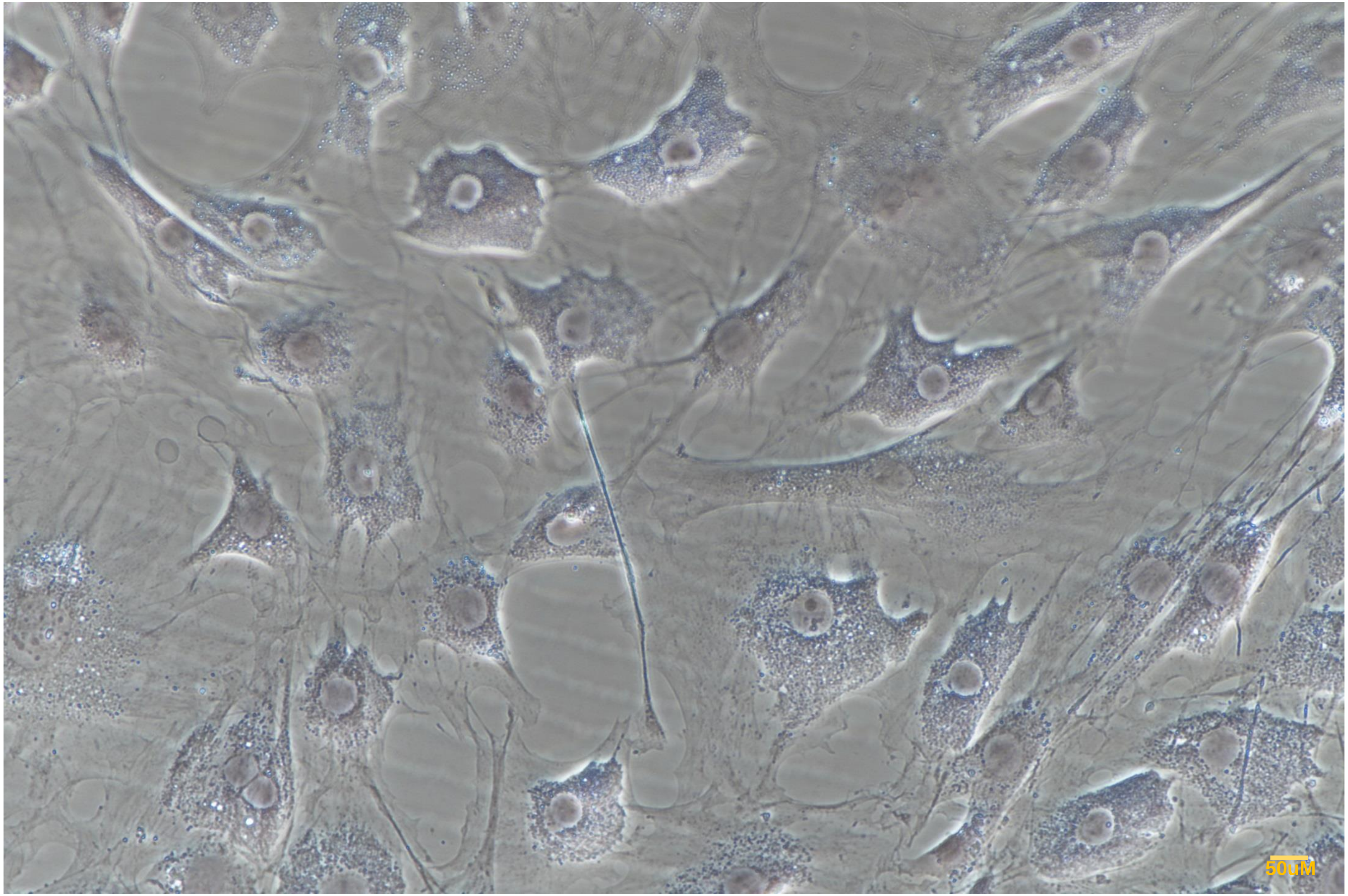

D-0 AM

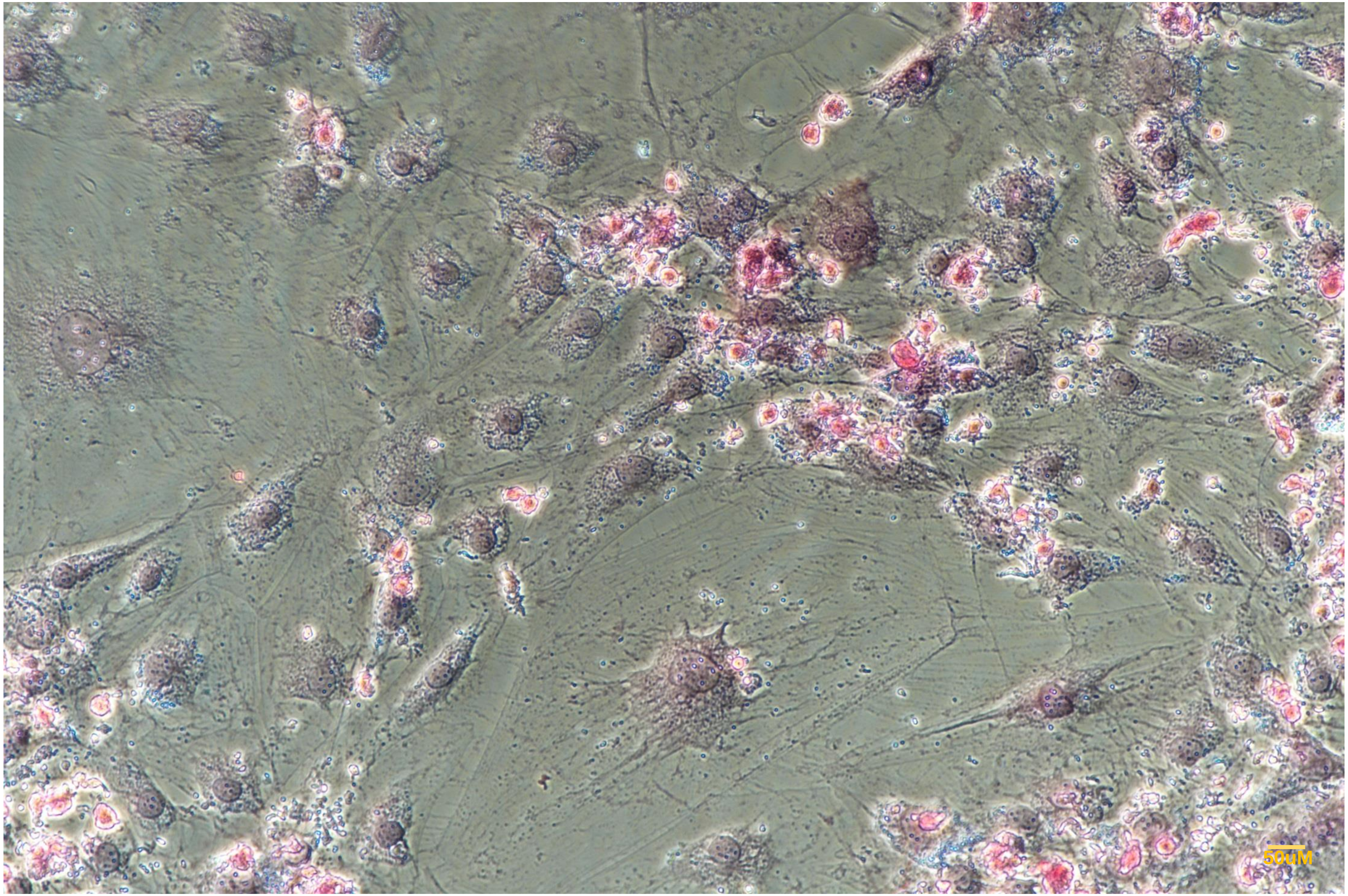

**D-0** **AM (HG)**

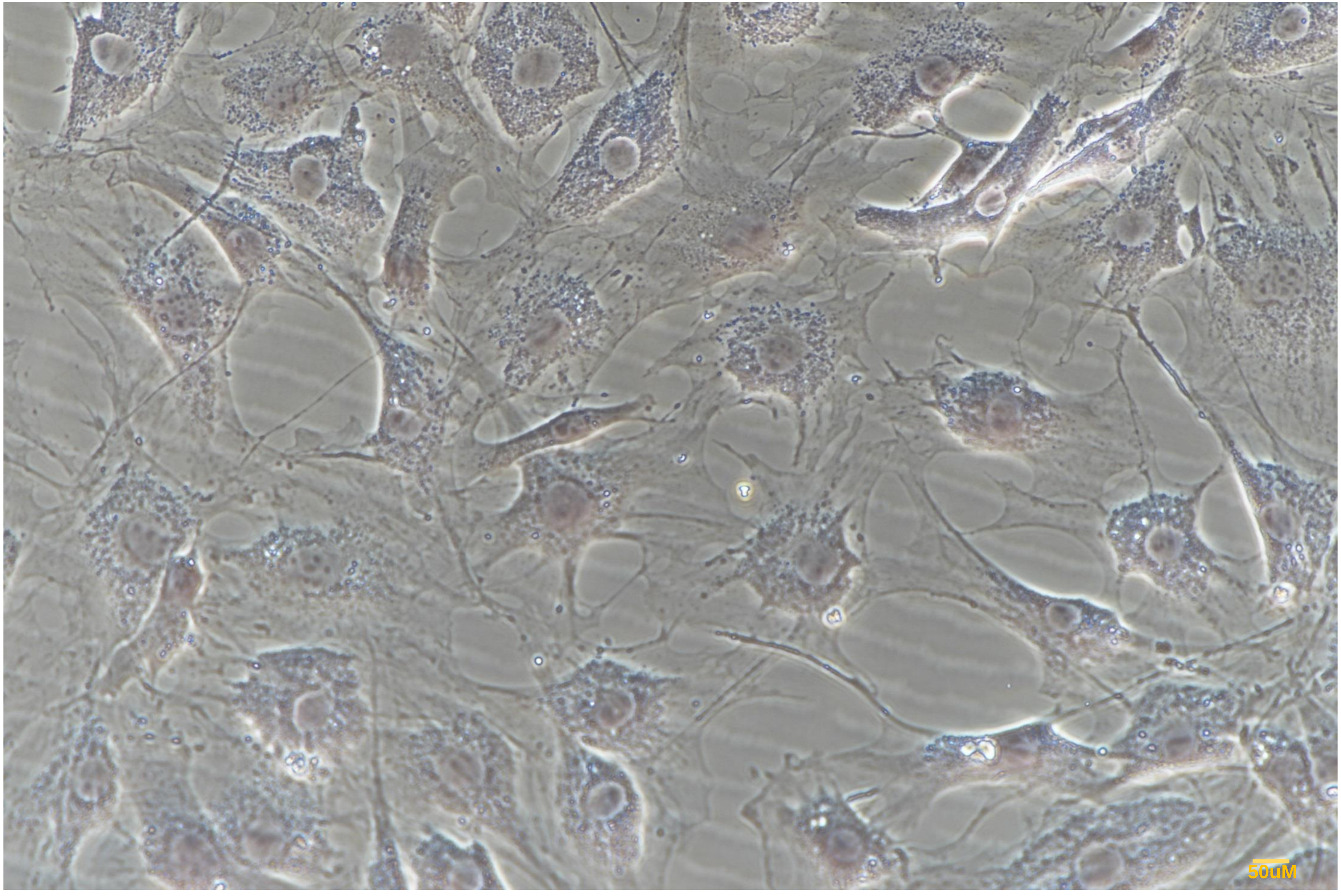

D-0 AM (HG)

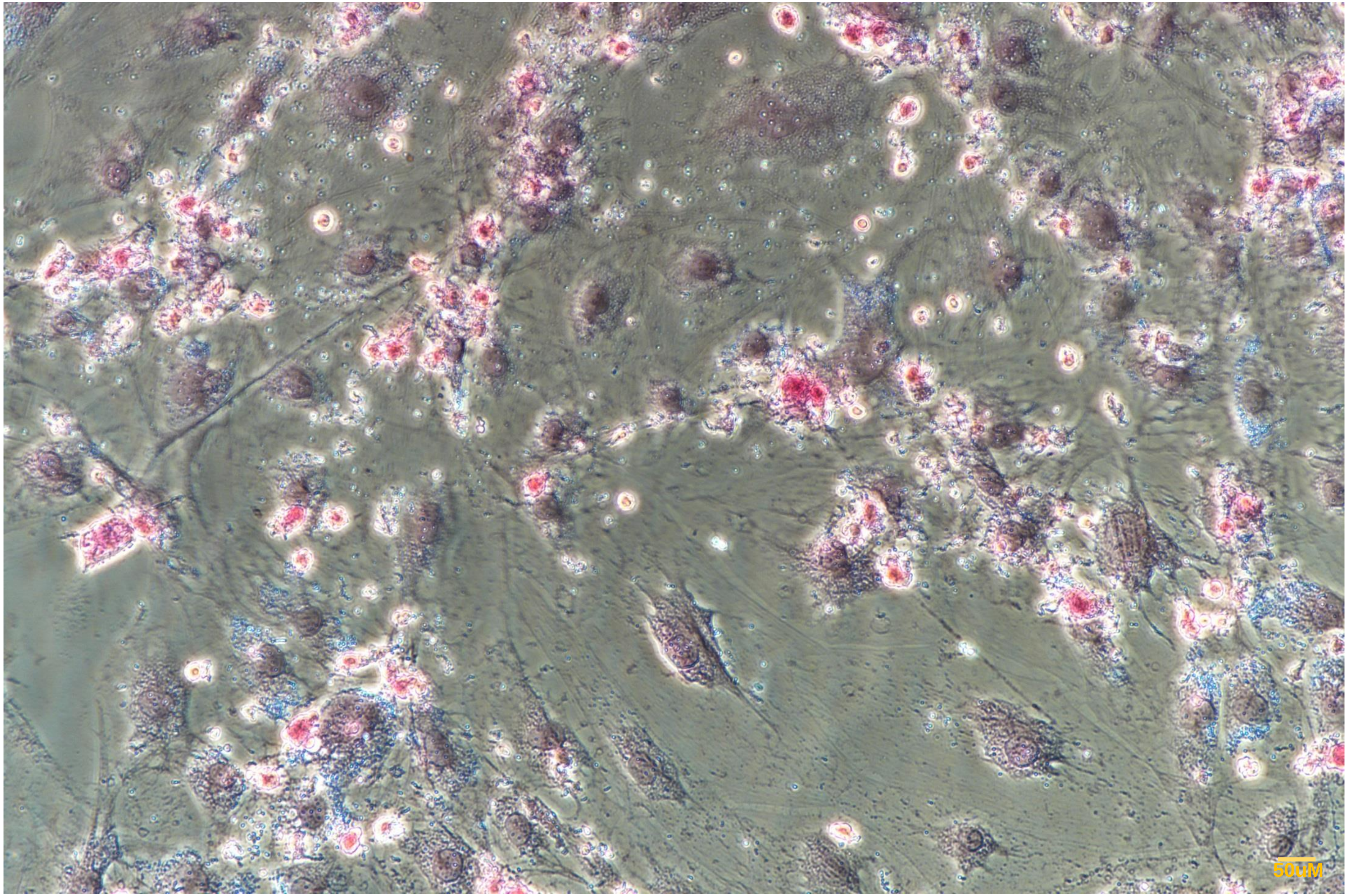

**D-0** **AM (HG, noGlut)**

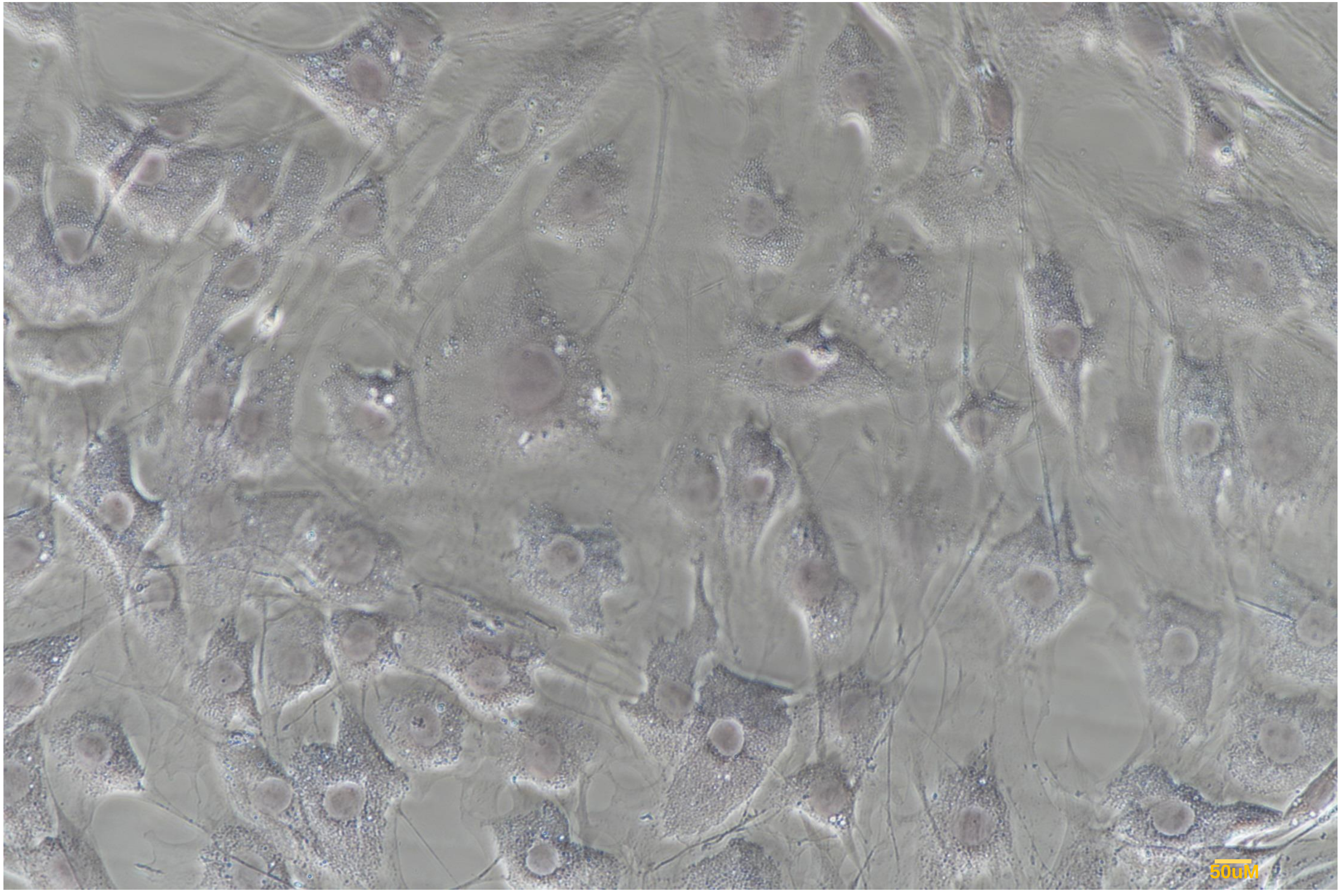

D-8 AM (HG, noGlut)

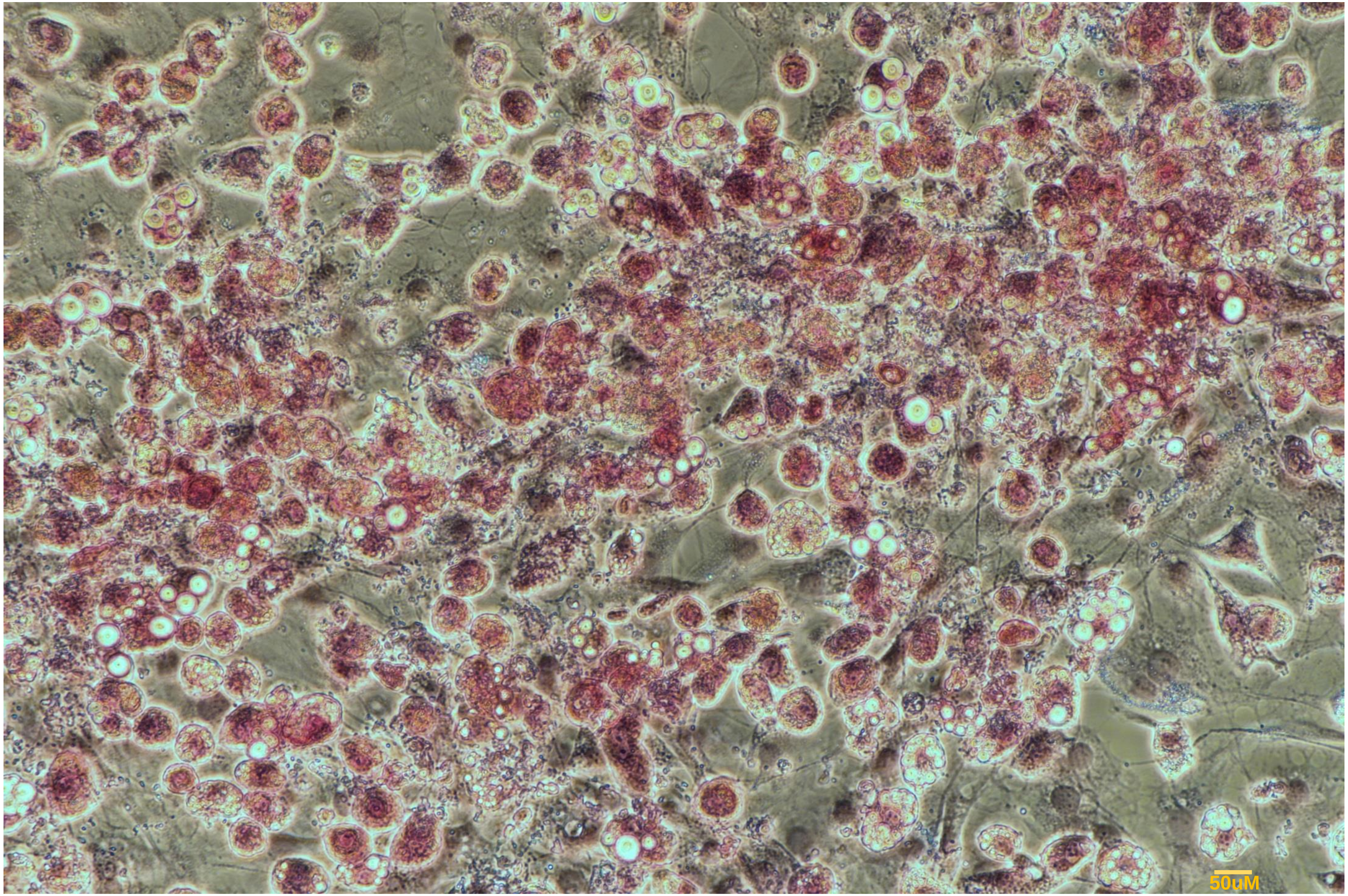

D-8 AM (LG)

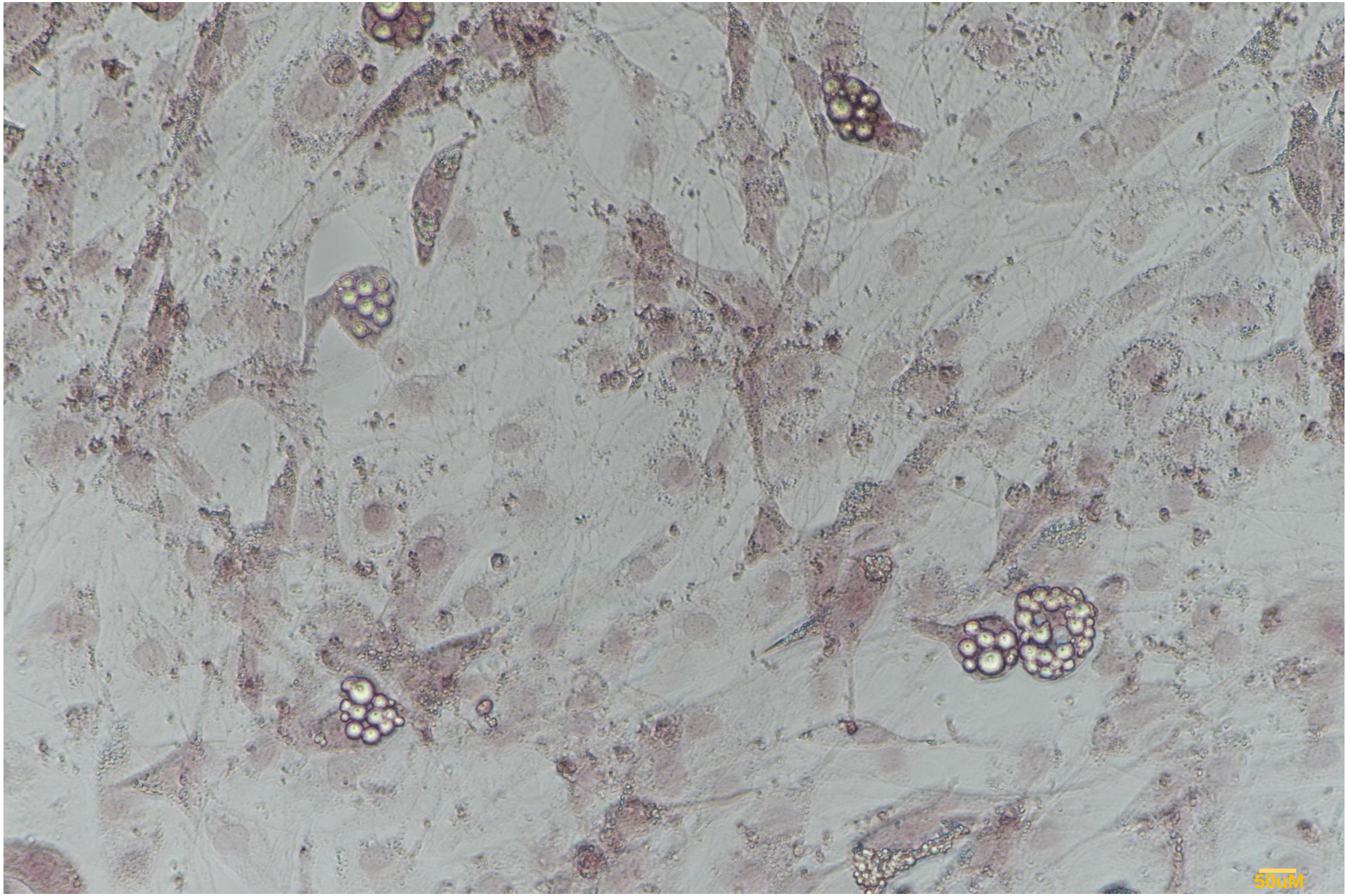

D-8 AM (LG)

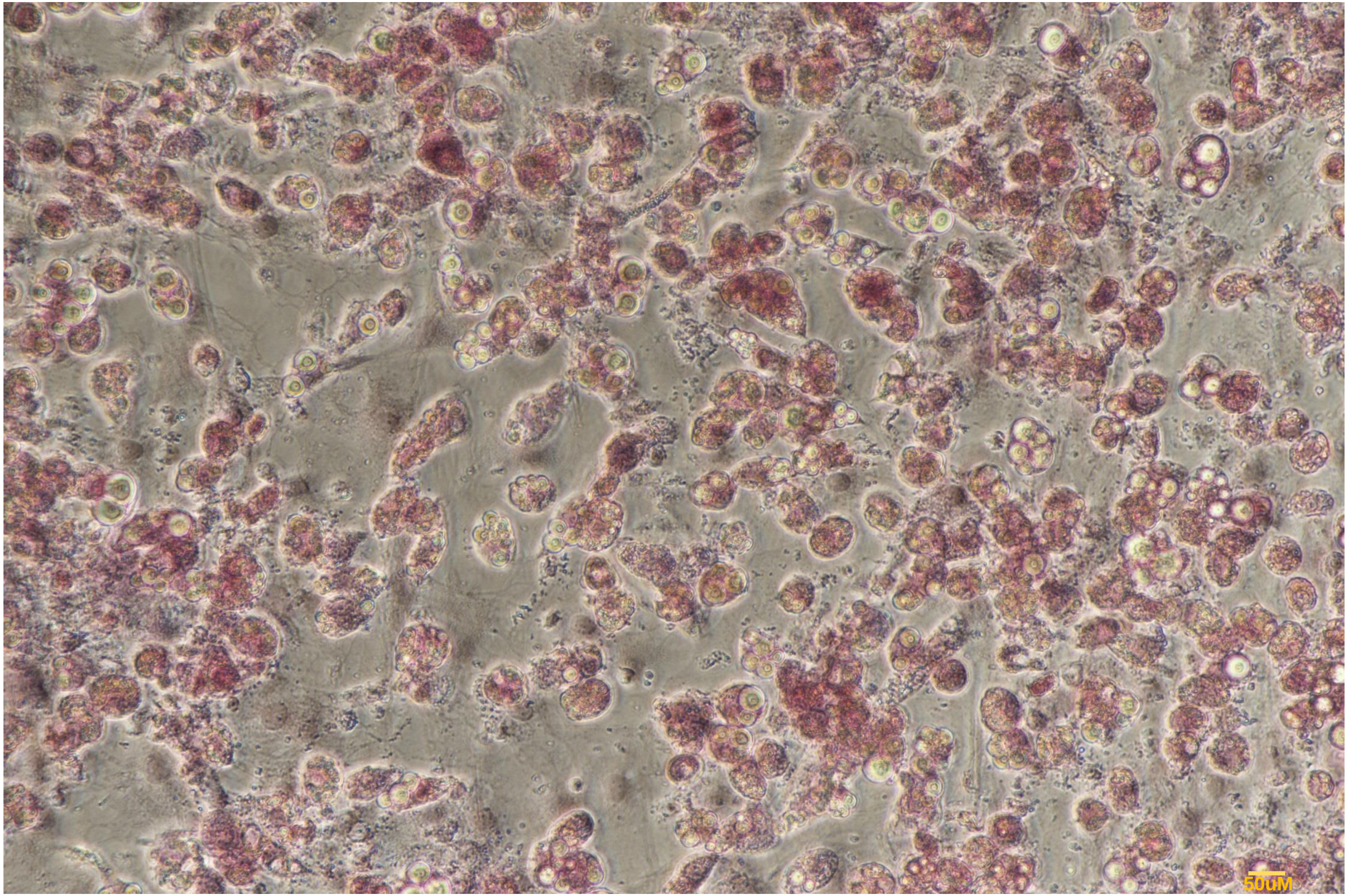

**D-8** **AM (HG, HGlut)**

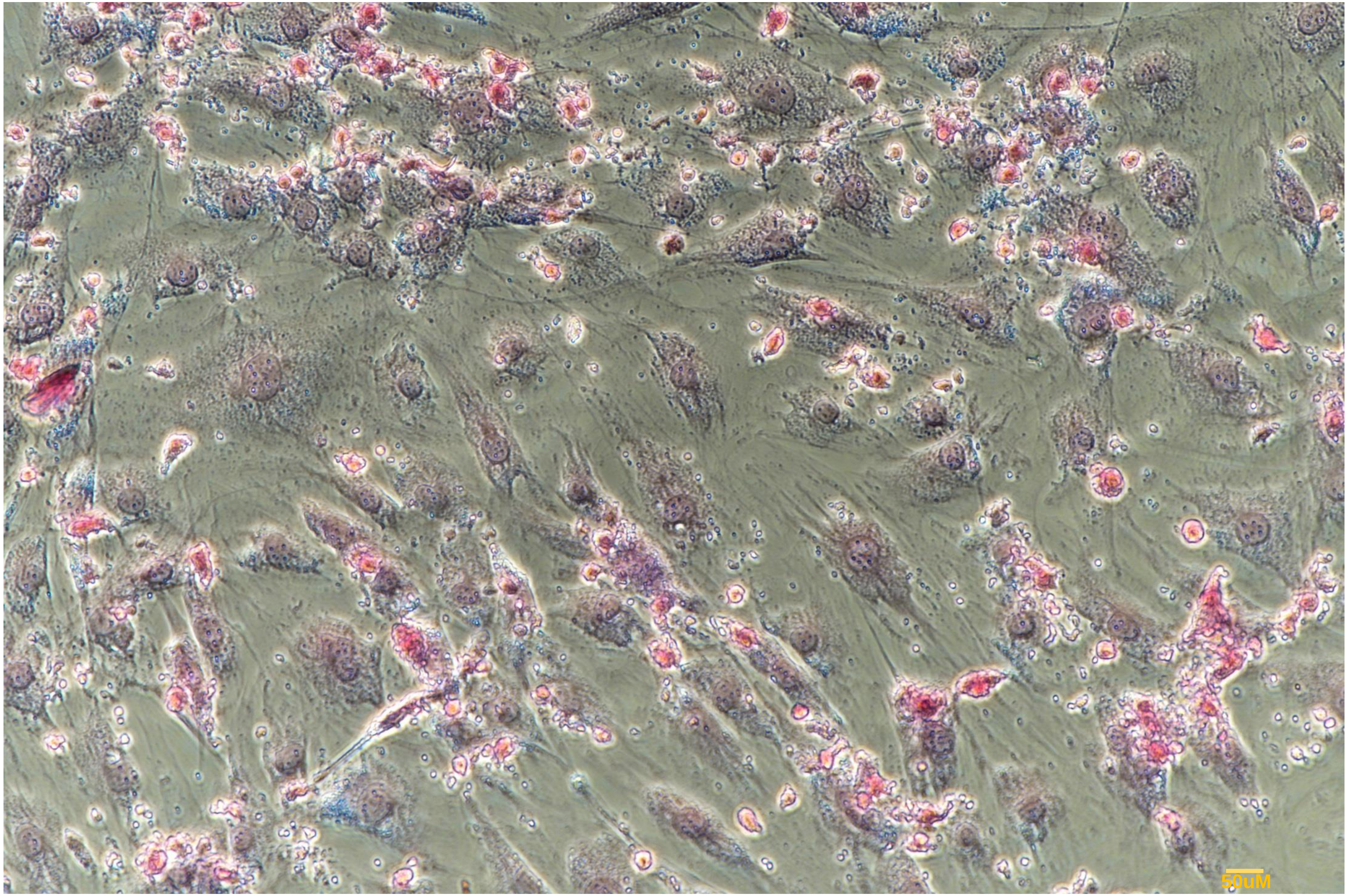

SF.2 C)

D-0 AM

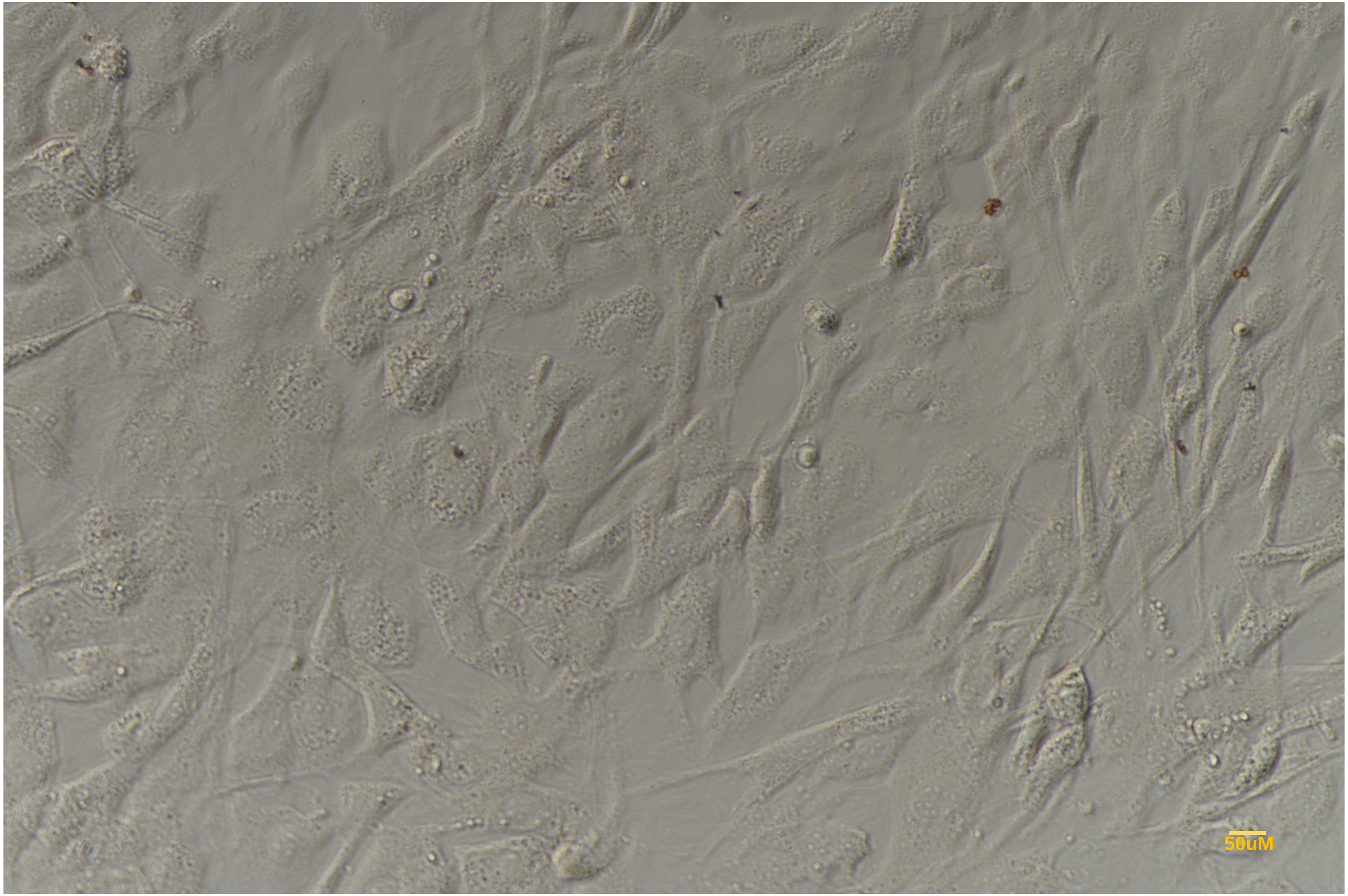

D-8 AM

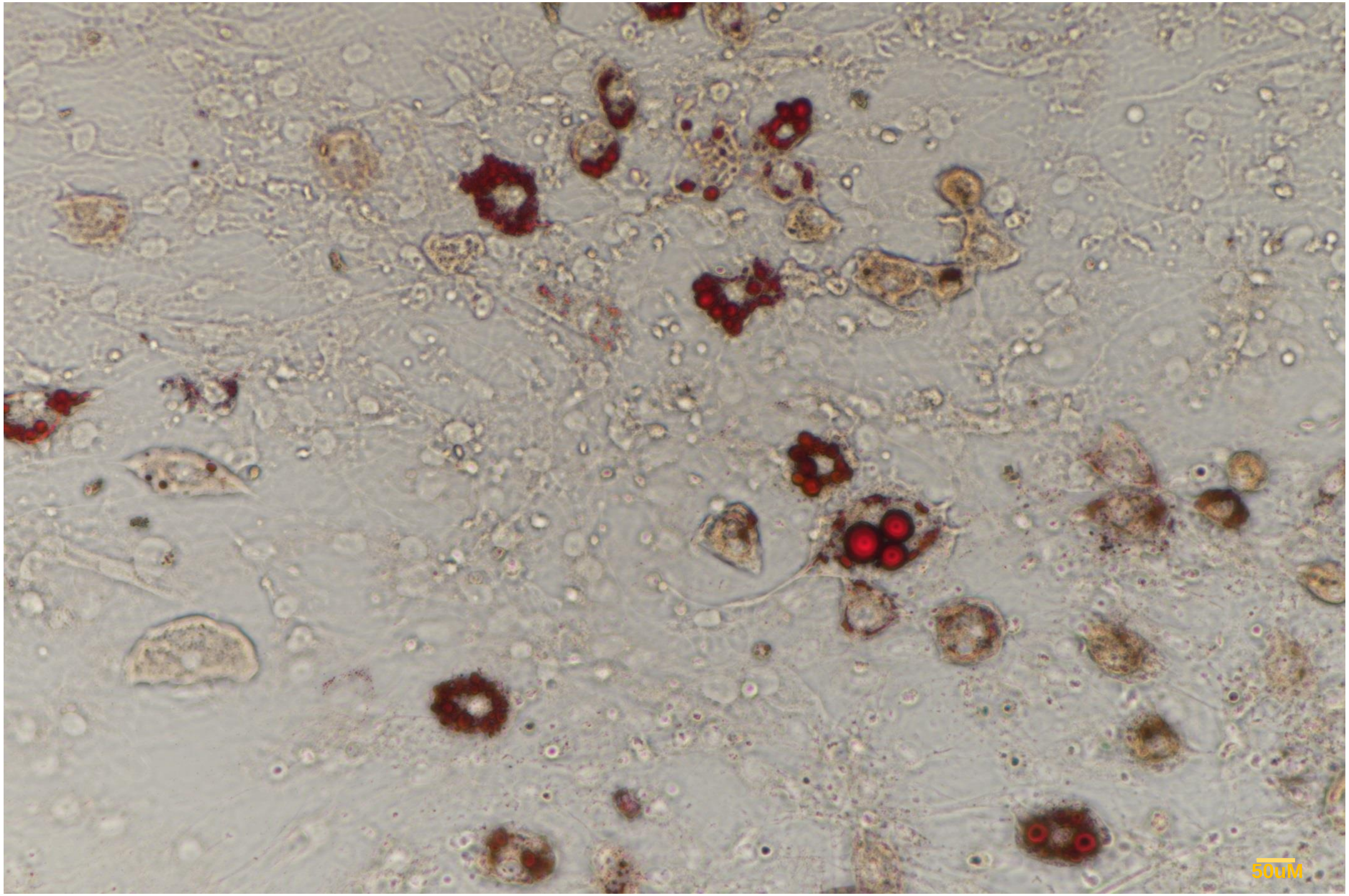

D-0 AM (HG)

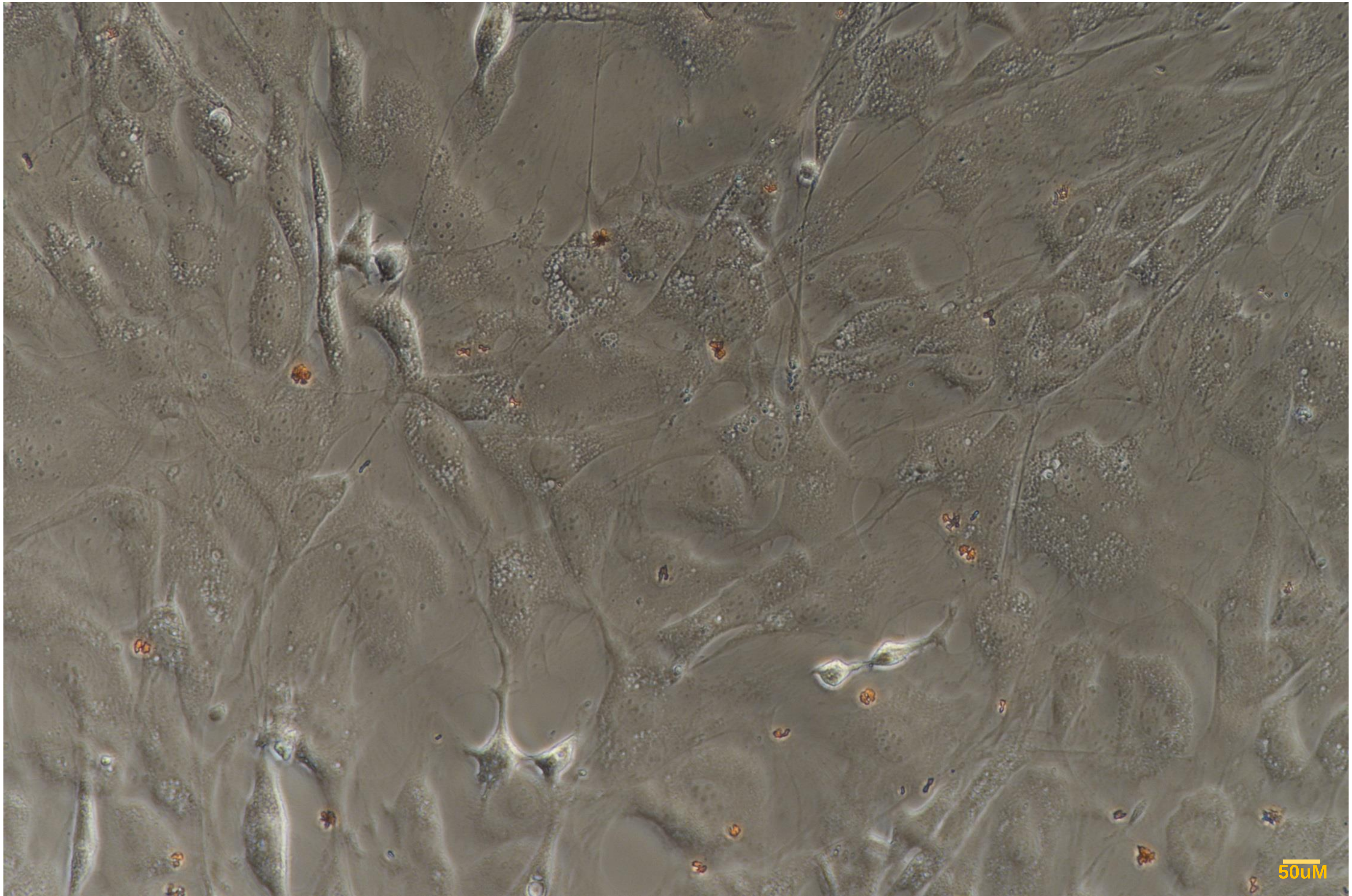

D-8 AM (HG)

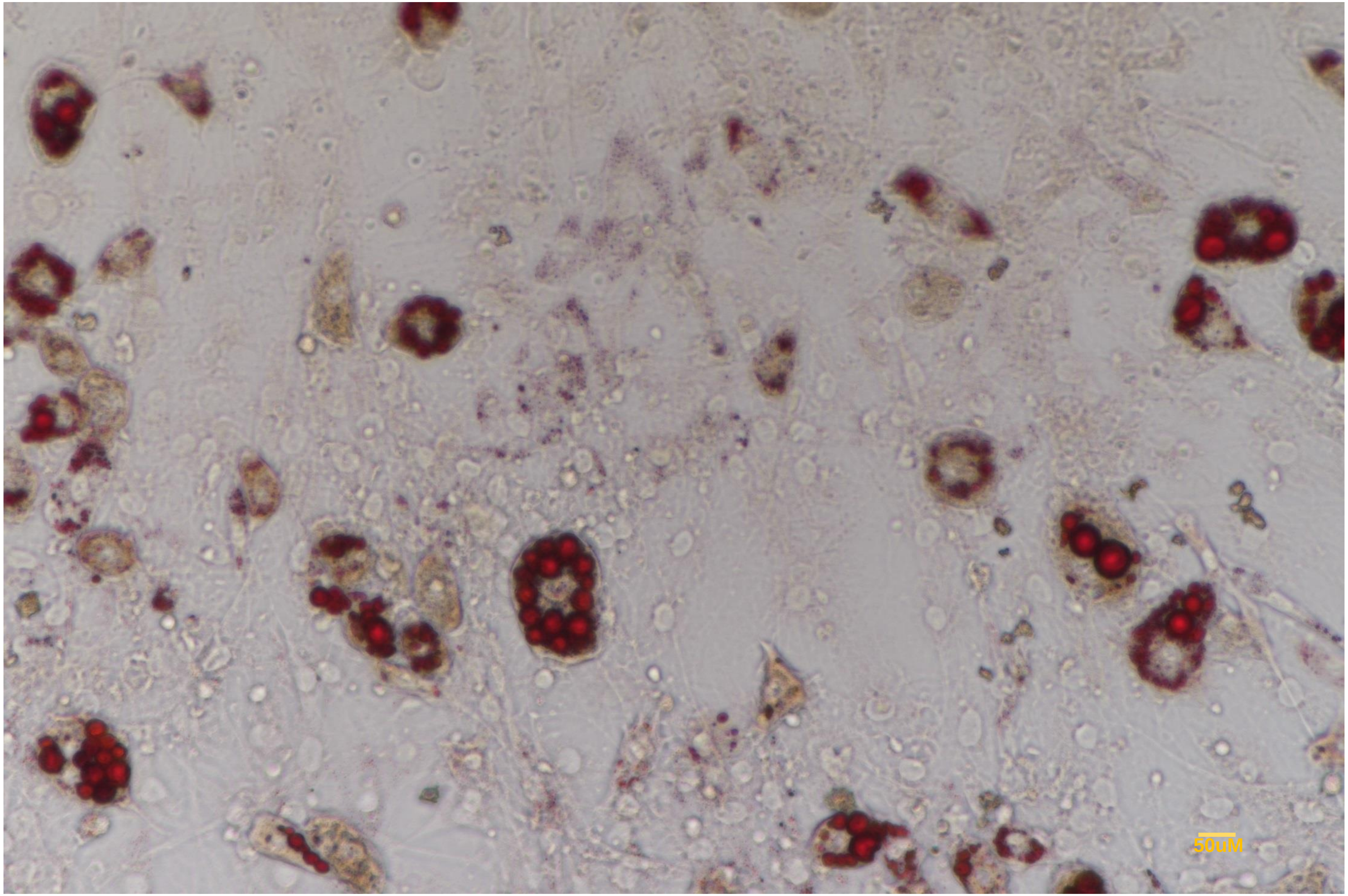

**D-0** **AM (HG, noGlut)**

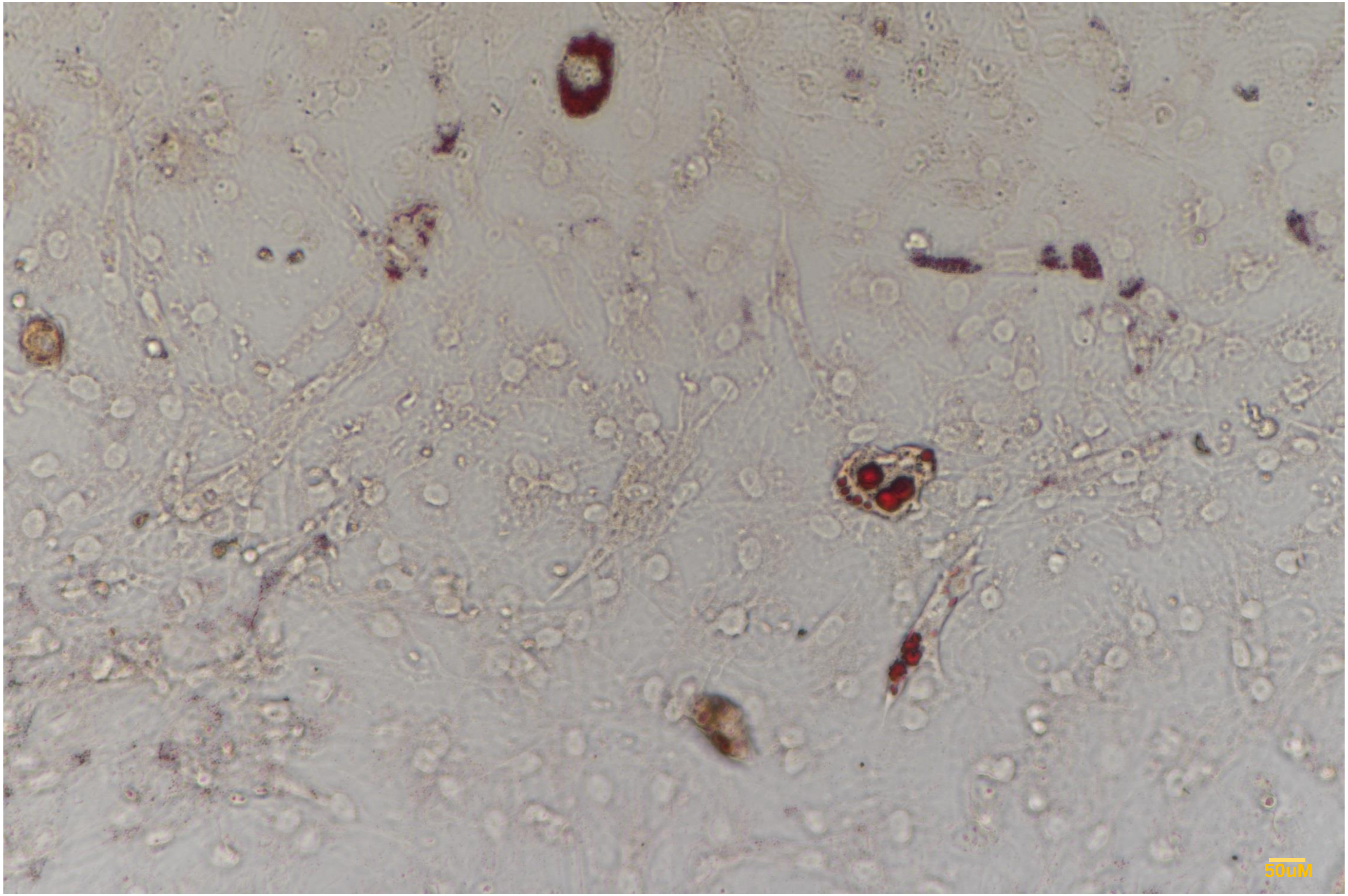

D-8 AM (HG, noGlut)

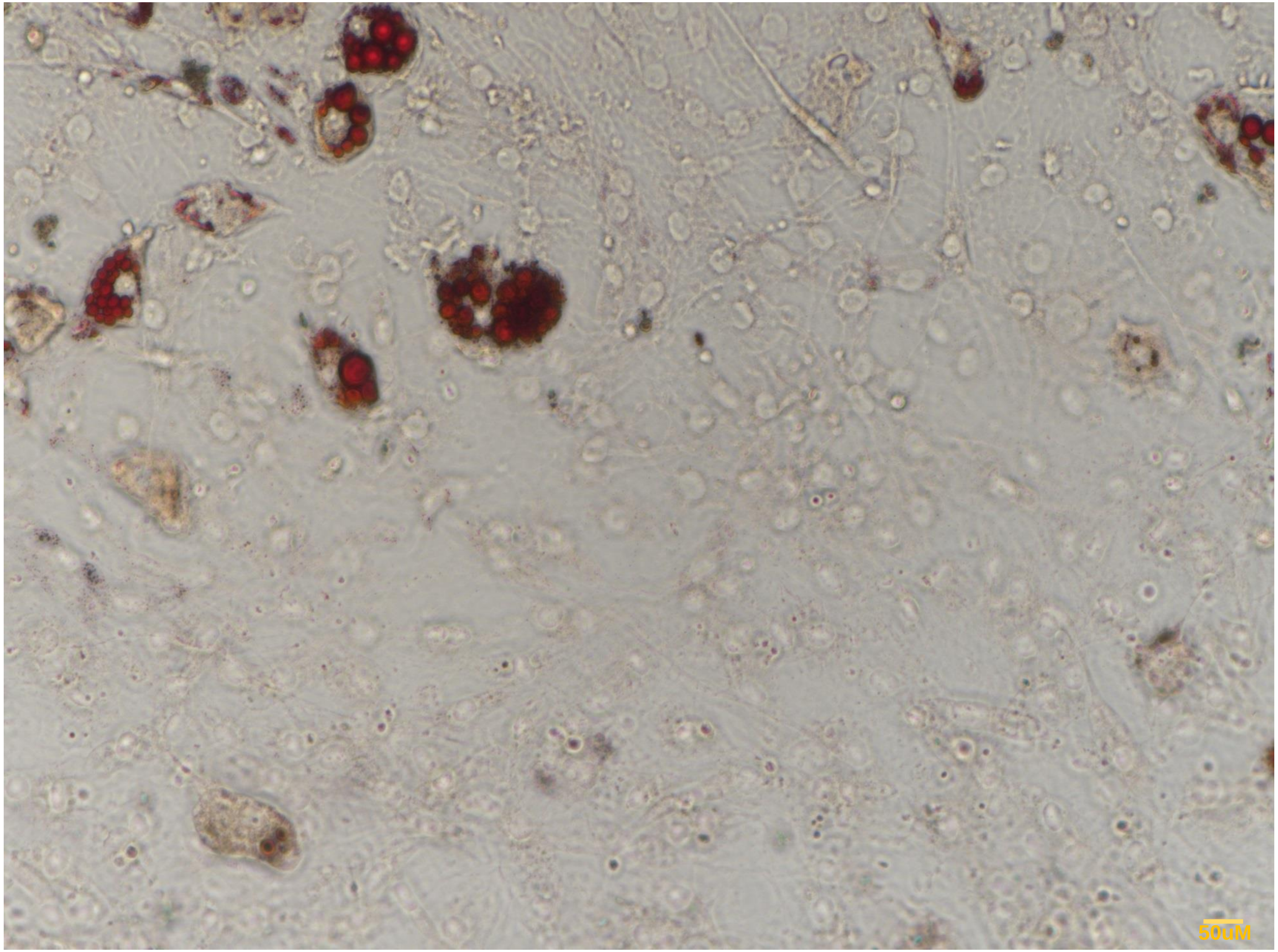

**D-0** **AM (LG)**

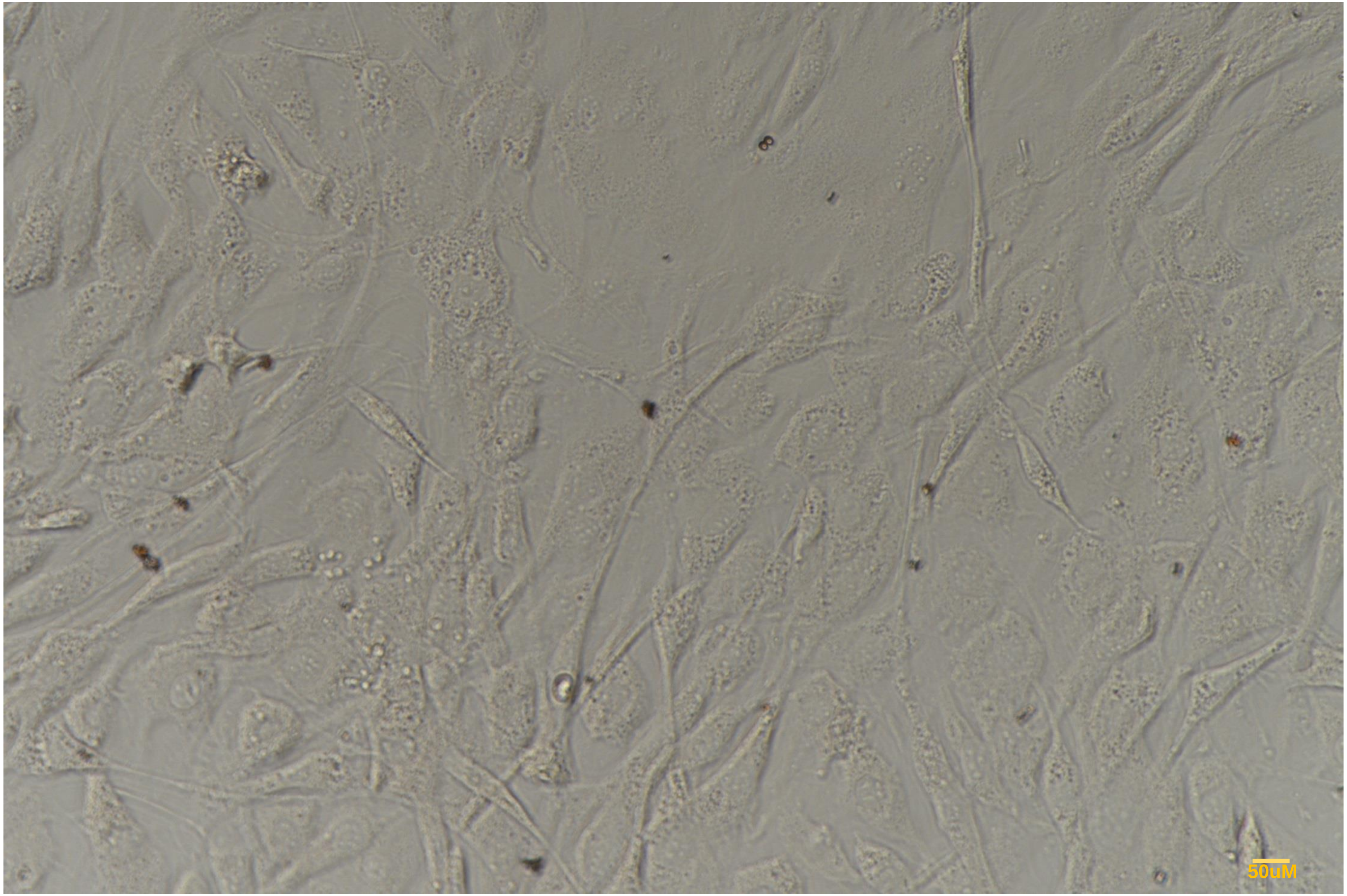

D-8 AM (LG)

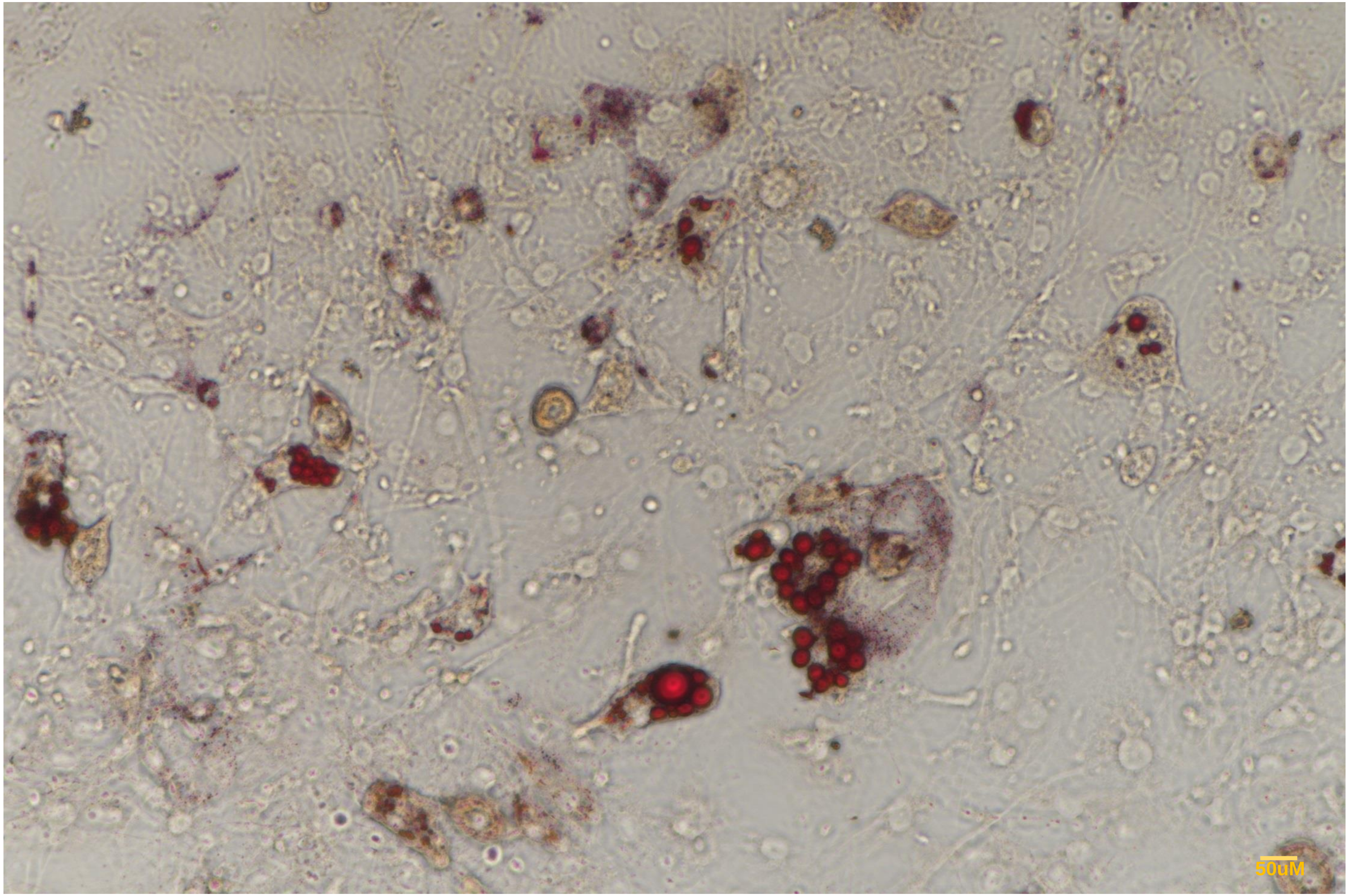

## D-0 AM (HG, HGlut)

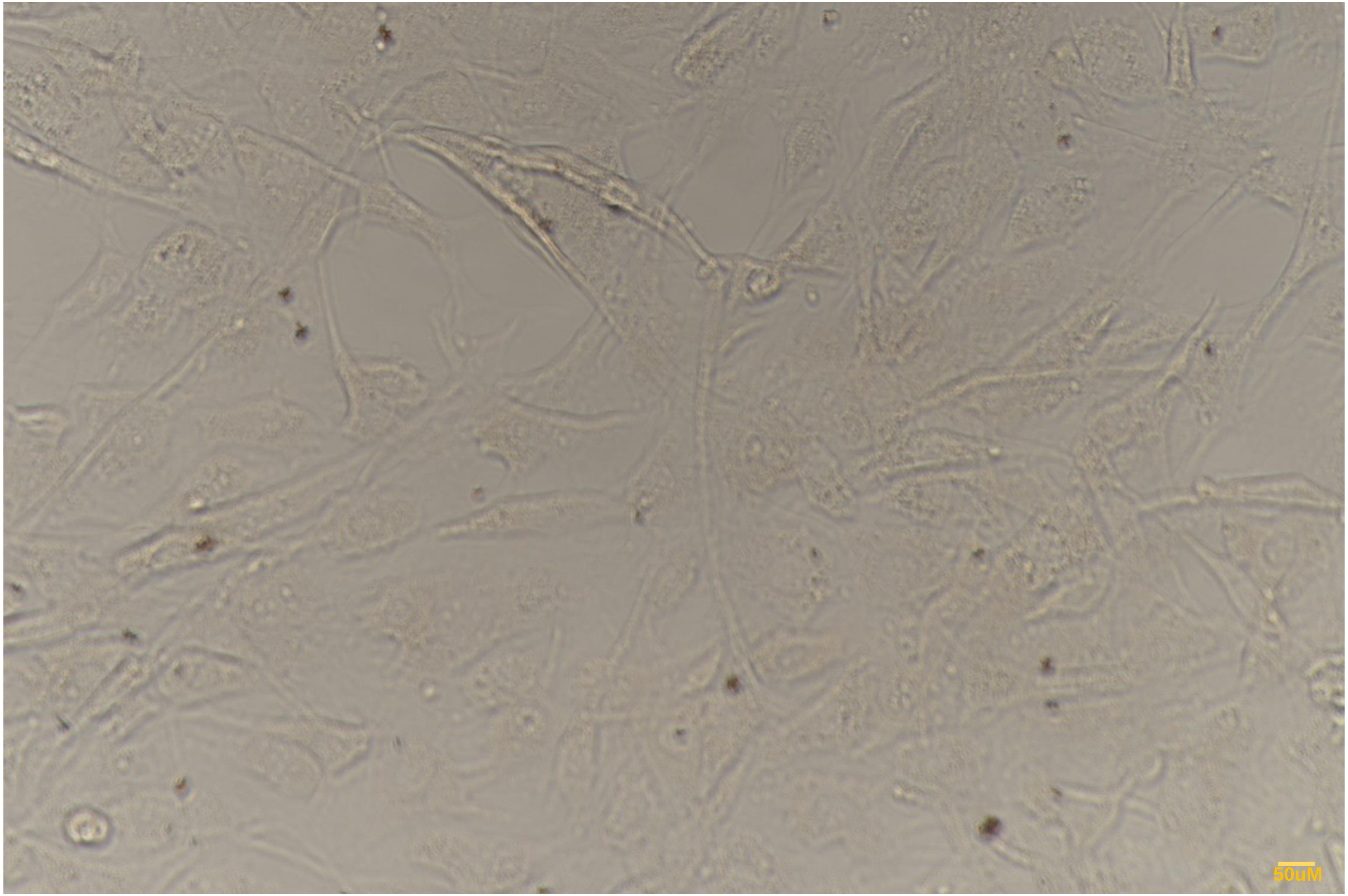

**D-8** **AM (HG, HGlut)**

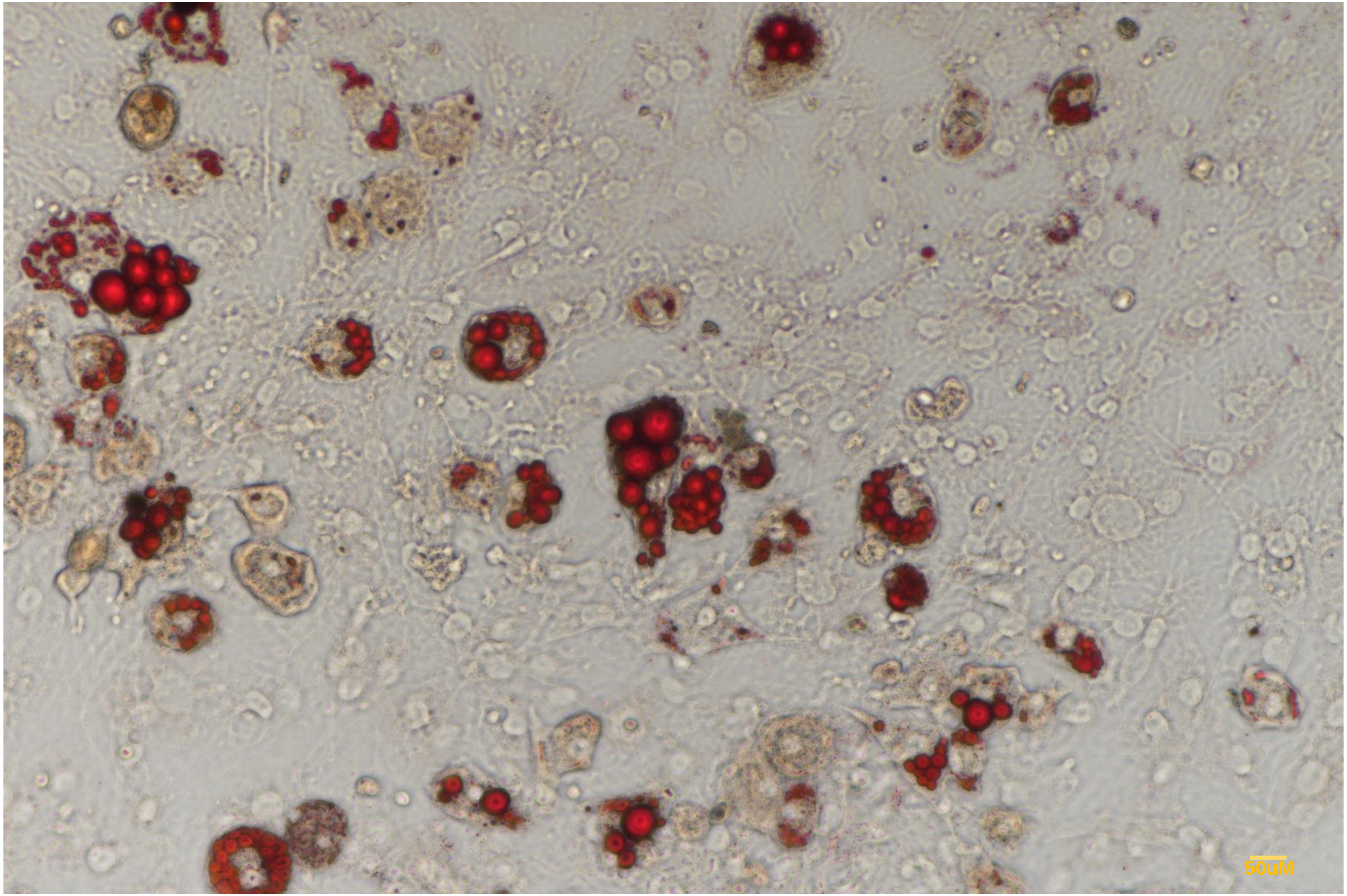

Supplement: Supplementary file 3 — Original Data File [file 41420_2022_1077_MOESM3_ESM.pdf]
